# A Study on Repositioning Nalidixic Acid via Lanthanide Complexation: Synthesis, Characterization, Cytotoxicity and DNA/Protein Binding Studies

**DOI:** 10.3390/ph15081010

**Published:** 2022-08-17

**Authors:** Ana-Madalina Maciuca, Alexandra-Cristina Munteanu, Mirela Mihaila, Mihaela Badea, Rodica Olar, George Mihai Nitulescu, Cristian V. A. Munteanu, Valentina Uivarosi

**Affiliations:** 1 Department of General and Inorganic Chemistry, Faculty of Pharmacy, Carol Davila University of Medicine and Pharmacy, 6 Traian Vuia Str., 020956 Bucharest, Romania; 2 Center of Immunology, Stefan S. Nicolau Institute of Virology, 285 Mihai Bravu Ave, 030304 Bucharest, Romania; 3 Department of Inorganic Chemistry, Faculty of Chemistry, University of Bucharest, 90-92 Panduri Str., 050663 Bucharest, Romania; 4 Department of Pharmaceutical Chemistry, Faculty of Pharmacy, Carol Davila University of Medicine and Pharmacy, 6 Traian Vuia Str., 020956 Bucharest, Romania; 5 Romanian Academy-Institute of Biochemistry (IBAR), 296 Splaiul. Independenţei, 060031 Bucharest, Romania

**Keywords:** drug repositioning, quinolones, nalidixic acid, lanthanide ions, anticancer, DNA binding, serum proteins binding

## Abstract

“Drug repositioning” is a modern strategy used to uncover new applications for out-of-date drugs. In this context, nalidixic acid, the first member of the quinolone class with limited use today, has been selected to obtain nine new metal complexes with lanthanide cations (La^3+^, Sm^3+^, Eu^3+^, Gd^3+^, Tb^3+^); the experimental data suggest that the quinolone acts as a bidentate ligand, binding to the metal ion via the keto and carboxylate oxygen atoms, findings that are supported by DFT calculations. The cytotoxic activity of the complexes has been studied using the tumoral cell lines, MDA-MB-231 and LoVo, and a normal cell line, HUVEC. The most active compounds of the series display selective activity against LoVo. Their affinity for DNA and the manner of binding have been tested using UV–Vis spectroscopy and competitive binding studies; our results indicate that major and minor groove binding play a significant role in these interactions. The affinity towards serum proteins has also been evaluated, the complexes displaying higher affinity towards albumin than apotransferrin.

## 1. Introduction

Despite the fact that research continues to uncover new chemotherapy drugs and new applications for old ones, and several new cancer drugs are introduced to the market each year, the demand for new active molecules remains high due to the emergence of drug resistance and the growing number of new cancer cases. Quinolones have been traditionally used in the treatment of bacterial infections. Recently, more interest has been given to other properties of quinolones, such as immunomodulation [1], pro-apoptotic and antiproliferative activity [2,3,4,5,6,7], anti-metastatic potential [8,9], and as human topoisomerase II α inhibitors [10]. All these aspects have highlighted their suitability for repositioning as anticancer agents with some quinolones having a number of advantages over other topoisomerase II inhibitors in terms of toxicity, development of drug resistance and drug-induced secondary cancers, better physicochemical qualities and improved pharmacokinetic profiles [11,12,13]. Furthermore, quinolone derivatives are distinguished by their versatility and ease of synthesis which can be accomplished using a variety of techniques and building blocks [14]. These considerations drove the development of a slew of derived chemical structures, which has culminated with vosaroxin (formerly voreloxin), the first-in-class anticancer quinolone approved by the FDA for the treatment of acute myeloid leukemia [15]. 

Some of the quinolone–metal complexes characterized so far are of interest because of their DNA-binding [16,17,18,19], antibacterial [20,21], antitumoral [22,23], antifungal [23], and antiparasitic [24] properties. Moreover, gold complexes of norfloxacin, levofloxacin and sparfloxacin present antimetastatic properties, and are non-toxic towards normal cells [25]. Copper(II) complexes of N-propyl-norfloxacin displayed excellent activity against leukemia cells [26], and complexes of moxifloxacin and sparfloxacin against breast cancer cell lines [22,27].

**Figure 1:**
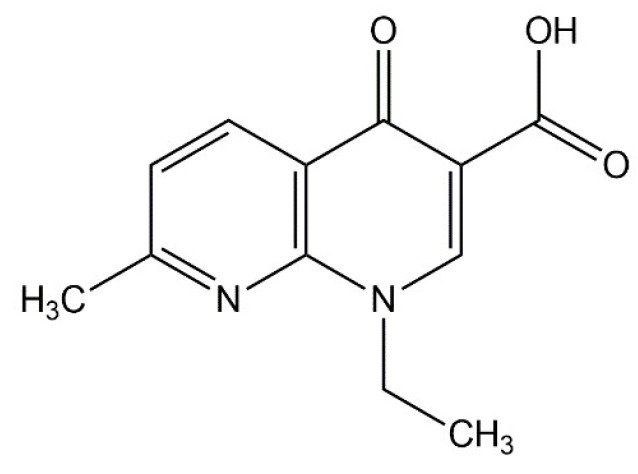
Chemical structure of nalidixic acid.

Nevertheless, research on metal complexes of quinolones with anticancer activity is still largely underexplored. With this in mind, we chose to investigate the potential of nalidixic acid metal complexes for cancer treatment. Nalidixic acid (2-ethyl-7-methyl-4-oxo-1,8-naphtyridine-3-carboxylic acid; Figure 1) was the first member of the quinolone drug class, originally synthesized in 1962 [28] by G.Y. Lesher et al. and introduced in therapy two years later for the treatment of uncomplicated urinary tract infections [29]. Its narrow-spectrum activity is based on its ability to block bacterial RNA and vital processes such as protein synthesis. The drug is, however, susceptible to the emergence of resistant mutants [30], which has limited its use in therapy. Several attempts have been made to reintroduce this molecule in therapy, including the synthesis of peptide conjugates with increased antibacterial activity [31], metal complexes [32,33,34,35,36,37,38] and hybrid nanocapsules containing nalidixic acid–vanadium complexes [39]. 

Due to the large ionic radii of the lanthanide ions, their complexes have high coordination numbers, usually ranging from 6 to 12 [40]. Furthermore, their coordination bonds lack directionality since they have a fundamentally electrostatic character, allowing organic ligands the opportunity to approach and accommodate themselves sterically around the metal center in a nearly random manner. As a result, their coordination polyhedra can take on a wide variety of different geometries [41]. Their versatility in shape and size, together with their magnetic and luminescent properties, allow for a wide range of applications, including development of luminescence sensors and probes for cellular imaging [42,43], contrast agents for magnetic resonance imaging [44], thermosensors [45] or drug development ([46,47] and citations therein). 

The biological activity of lanthanide cations (Ln^3+^) may arise from their ability to mimic physiological ions like Mg^2+^, Ca^2+^ or Zn^2+^ as co-factors in the structure of some metalloenzymes. As hard Lewis acids with fast ligand exchange rates, and no apparent redox chemistry, Ln^3+^ are well suited to mimic the reactivity of these biological metal cofactors [48,49,50,51,52]. As Mg^2+^ ions play a key role in the interaction of quinolones with both DNA and proteins, we expected lanthanide coordination to increase the ability of nalidixic acid to bind biomacromolecules in a similar manner to Mg^2+^ and improve its biological activity. Also, we expected that the presence of hydrophobic methyl and ethyl groups from multiple nalidixate moieties bound to the lanthanide ion would provide a hydrophobic recognition element that would interact with DNA and proteins in an effective manner [37].

To date, several metal complexes containing nalidixic acid or derivatives have presented promising antitumoral activity [32,33,34,35,36,37,38]. Of note, two seven-coordinate La(III) and Sm(III) complexes with nalidixic acid have been previously synthesized [53], yet their biological properties have not been assessed, so far. In this paper, we report the synthesis and characterization of nine complexes of La(III), Sm(III), Eu(III), Gd(III) and Tb(III) and nalidixic acid in 1:2 and 1:3 metal:ligand molar ratios. The geometries of the complexes were further optimized using the semiempirical Sparkle/PM7 method implemented in MOPAC software package, followed by TDDFT calculations carried out in the Orca program suite. The cytotoxic activity of the free ligand and its complexes was studied on human breast adenocarcinoma MDA-MB-231 and human colorectal adenocarcinoma LoVo cell lines, as well as on the healthy human umbilical vein endothelial HUVEC cell line. Their affinity for DNA, human serum albumin (HSA) and apotransferrin (apo-Tf) was also assessed.

## 2. Results

### 2.1. Synthesis

**Scheme 1:**
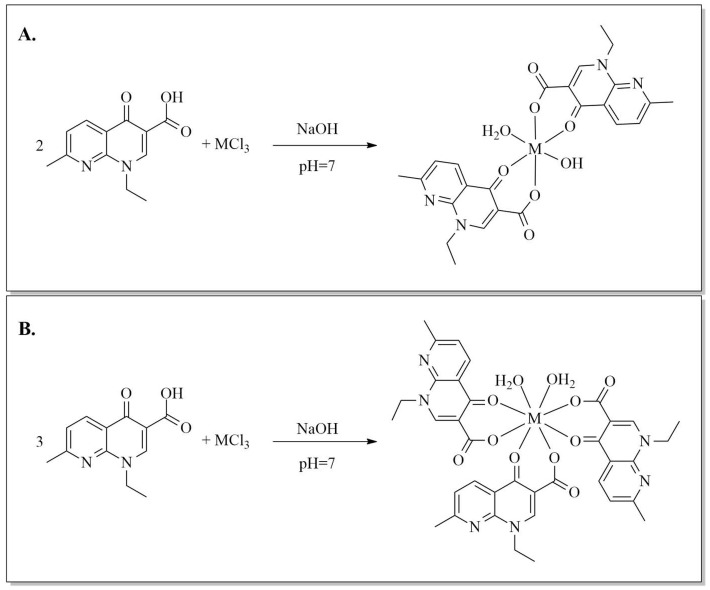
(**A**) Synthesis of the 1:2 metal complexes of nalidixic acid, where M = La^3+^, Sm^3+^, Eu^3+^, Gd^3+^, Tb^3+^. (**B**) Synthesis of the 1:3 metal complexes of nalidixic acid, where M = Sm^3+^, Eu^3+^, Gd^3+^, Tb^3+^.

Five novel M^3+^ complexes (M = La^3+^, Sm^3+^, Eu^3+^, Gd^3+^, Tb^3+^) in a 1:2 metal:ligand molar ratio and four M^3+^ complexes (M = La^3+^, Eu^3+^, Gd^3+^, Tb^3+^) in a 1:3 molar ratio were synthesized starting from nalidixic acid. Briefly, the metal salts were added to the sodium salt of nalidixic acid, and the mixtures were refluxed for 4.5 h (Scheme 1). The new compounds were isolated as off-white powders that dissolved in DMSO but not DMF and were insoluble in water or ethanol.

### 2.2. Structural Characterization

#### 2.2.1. UV–Vis–NIR Spectra

**Figure 2:**
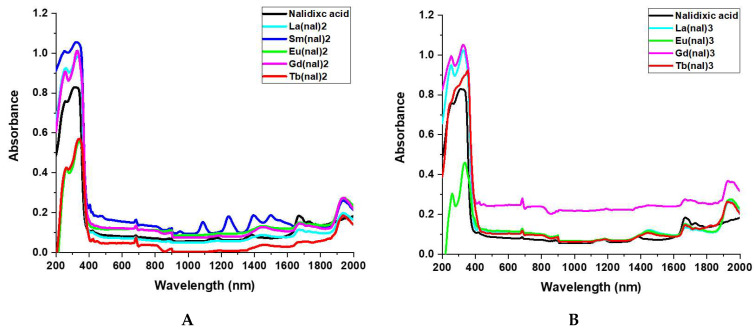
UV–Visible spectra of (**A**) nalidixic acid and M(nal)_2_, where M = La^3+^, Sm^3+^, Eu^3+^, Gd^3+^, Tb^3+^); (**B**) nalidixic acid and M(nal)_3_, where (M = La^3+^, Eu^3+^, Gd^3+^, Tb^3+^).

The UV–Vis–NIR spectra (Figure 2) of nalidixic acid presents two absorption peaks at 255 nm and 315 nm, respectively. These peaks are shifted in the spectra of the M(nal)_2_ (Figure 2A) and M(nal)_3_ (Figure 2B) complexes. The absorbances and related wavelengths are presented in Table S1. In addition, Sm(nal)_2_ presents specific absorption bands in the NIR region due to f-f transitions from the ground state ^6^H_5/2_ to ^6^F multiplet of Sm^3+^ ion (4f^5^) (Figure 2A).

#### 2.2.2. FT-IR Spectra

Band assignments are described in Tables S2 and S3, and the Fourier-transform infrared spectra are shown in Figure S1. In the spectrum of the free ligand, the stretching vibrations corresponding to the carboxylic acid arm (υC=Oc) can be seen at 1701 cm^−1^, and to the C=O pyridonic bond (υC=Op) at 1613 cm^−1^. The latter appears shifted to 1567–1570 cm^−1^ in the spectra of M(nal)_2_ complexes and 1557–1559 cm^−1^ for M(nal)_3_. At the same time, the former band is absent in the spectra of the metal complexes, whereas two additional bands emerge at 1614–1618 cm^−1^ and 1439–1443 cm^−1^. These new bands were assigned to the O-C-O asymmetric (O-C-Oas) and O-C-O symmetric (O-C-Os) stretching vibrations. In order to assess the binding mode, Δ values (υO-C-Oas - υO-C-Os) for the complexes (Δ = 173–176 cm^−1^) were compared with that of the sodium salt of the ligand (Δ = 194 cm^−1^). 

#### 2.2.3. Mass Spectra

Under the working conditions of the mass spectra analysis, DMSO molecules have replaced the coordinated water molecules and hydroxide ions, resulting in the generation of peaks corresponding to the following formulas: [M(nal)_2_(DMSO)_2_]^+^, [M(nal)_2_(DMSO)]^+^. Peaks of the type [M(nal)_2_]^+^ for the 1:2 series and [[M(nal)_3_]_2_+2H]^2+^ for the 1:3 series are also present (Figure S2). The spectra for all 1:2 and 1:3 complexes matched the natural isotopic abundances of the metals involved (for peak assignments see Materials and Methods). Of note, the mass spectra indicate that the complexes have polymeric structures, however, in the absence of crystallographic data, the monomeric forms of the complexes will be used in this work.

#### 2.2.4. Thermal Behavior

The simultaneous thermogravimetric (TG) and differential thermal analysis (DTA) curves of the trivalent lanthanide complexes heated in the 20–1000 °C temperature range show mass losses in three or four consecutive or overlapping steps (Figures S3 and S4). Thermal decomposition data are summarized in Tables S4 and S5. The first step between 50–235 °C (50–150 °C for Eu(nal)_2_ and Tb(nal)_2_) consists of endothermic elimination of the lattice water molecules. The anhydrous compounds are stable up to ~200 °C, with the exceptions of La(nal)_2_ and La(nal)_3_, for which the following step begins at 190 °C and 175 °C, respectively. The next steps are consistent with exothermic loss of water molecules and stepwise oxidative degradation of the organic frame. The loss of H_2_O molecules consists of an overlap of at least two processes as indicated by both TG and DTA curves, namely coordinated water and exothermic decomposition of the hydroxide groups. The degradation of the organic moiety most likely starts with the loss of the 3-methylpyridine moieties, followed by stepwise decomposition of the rest of the organic frame [54]. The residual masses found after thermal decomposition of the complexes are consistent with the formation of the most stable oxide [55]. For most complexes, the overall mass reduction is consistent with the formation of M_2_O_3_ as the final residue. For both Tb(nal)_2_ and Tb(nal)_3_, Tb_4_O_7_ is produced as a result of the partial oxidation of Tb(III) to Tb(IV).

##### H NMR Spectra

**Figure 3:**
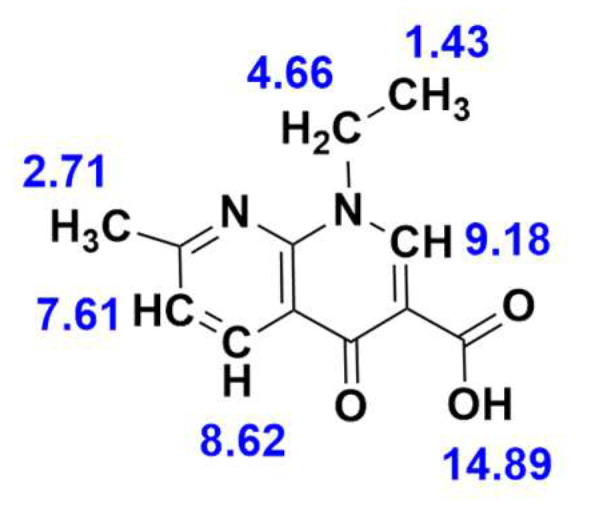
^1^H NMR *chemical shift* (δ) values *in ppm* for nalidixic acid in DMSO-*d_6_*.

The ^1^H NMR spectra for nalidixic acid and La(nal)_3_ (Figures S5 and S6) were recorded in DMSO-*d_6_* relative to the internal standard, tetramethylsilane (TMS). Chemical shifts in ppm for nalidixic acid are depicted in Figure 3. The singlet at 2.71 ppm in the ^1^H NMR spectrum of free nalidixic acid can be assigned to the three equivalent -CH_3_ protons of the 2-methyl pyridine moiety, and appears shifted to 2.57 ppm in the spectrum of La(nal)_3_, Furthermore, the -CH_3_ and -CH_2_ protons of the 1-ethyl-7-methyl-4-oxo-[1,8]naphthyridine moiety of the free ligand were observed at 1.43 (triplet) and 4.66 (quartet) ppm, respectively. In the spectrum of the complex, these peaks are shifted to 1.23 and 4.40 ppm (for La(nal)_3_). The three aromatic protons of the 4-oxo-[1,8]naphthyridine moiety appear in the spectrum of nalidixic acid at 7.61, 8.62 (as doublets) and 9.18 (singlet) ppm. Upon coordination, these peaks appear shifted to 7.28 ppm, 8.16 ppm and 8.83 ppm, respectively. Moreover, the characteristic signal for the proton of -COOH can be seen at δ = 14.89 ppm in the spectrum of the free ligand. This peak disappears entirely in the ^1^H NMR spectrum of La(nal)_3_, which indicates that the coordination of nalidixic acid to La(III) occurs via the deprotonated carboxylic group [33,56]. It should be noted that, due to the limited solubility of the complex, the ^1^H NMR spectrum of La(nal)_3_ shows rather broad signals, for which the multiplicity could not be assigned.

### 2.3. Computational Studies

**Figure 4:**
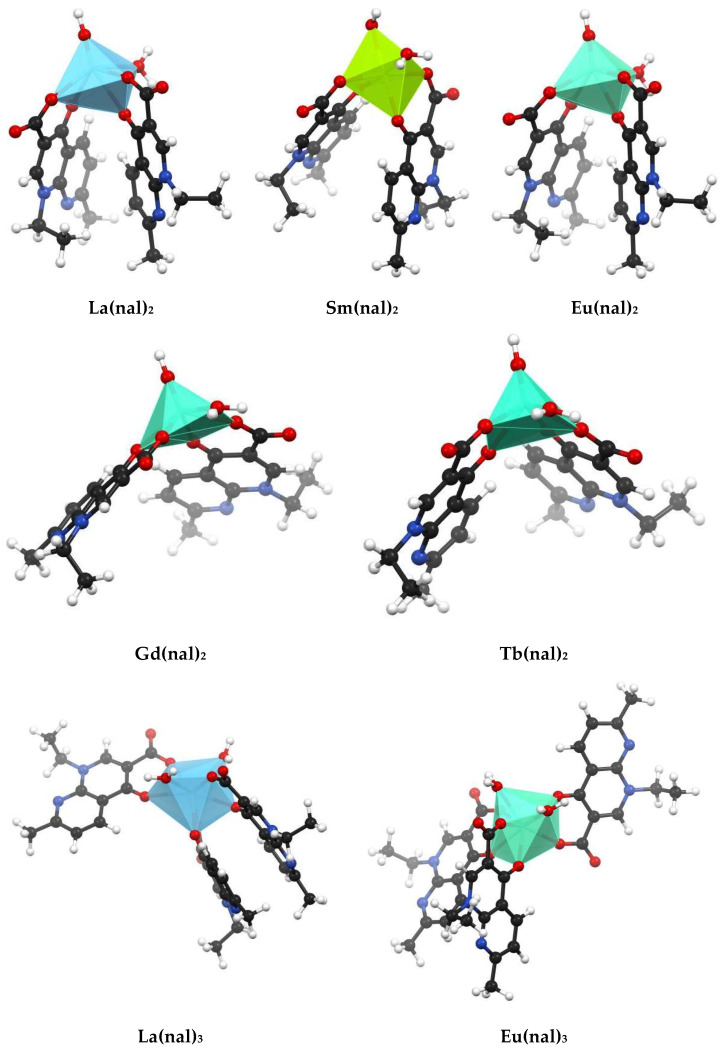

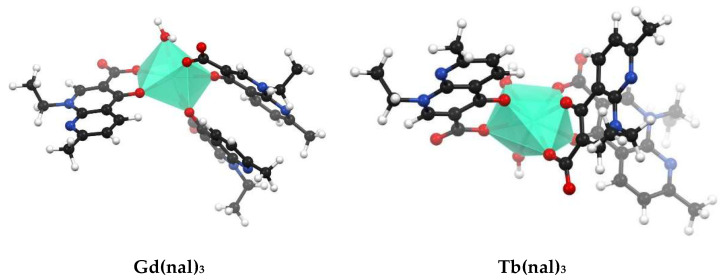
Optimized molecular geometries of M(nal)_2_ and M(nal)_3_ computed using the semiempirical method PM7 in MOPAC; the images were obtained using Mercury^®^ 2020.2 CSD Release (Cambridge Crystallographic Data Centre, Cambridge, UK).

The fully optimized geometries of the complexes were obtained using the semiempirical method PM7 in MOPAC and are shown in Figure 4. Table S6 presents the values of the selected bond lengths, angles and charge densities. For all complexes the M-O(nal) and M-O(H_2_) bond lengths vary in the range of 2.256 to 2.625 Å. The polyhedra around the metal center corresponds to a distorted octahedral geometry for La(nal)_2_, Sm(nal)_2_ and Eu(nal)_2_, and distorted pentagonal pyramid for Gd(nal)_2_ and Tb(nal)_2_. The difference in geometries stems from the larger values computed for Gd(nal)_2_ and Tb(nal)_2_ for the O(H)-M-O(nal) angles and the dihedral angles between two nalidixate moieties (Table S6). In the M(nal)_3_ complexes, the metal center is eightfold coordinated in a square antiprismatic geometry (Figure 4).

Selected data and assignments resulting from the computed vibrational frequencies for Eu(nal)_2_ and Eu(nal)_3_ are detailed in Table S7. The O-H stretches give weak, broad bands around 3395 cm^−1^ in the experimental IR spectra of the complexes that are underestimated in the predicted spectra in the range of 2700–2900 cm^−1^ (Figure S1), which has been previously reported for frequencies above 2500 cm^−1^ [57]. For Eu(nal)_2_, the bands corresponding to the asymmetric and symmetric stretching vibrations of the carboxylate moiety coordinated to the metal centers are present in the experimental spectrum at 1615 and 1442 cm^−1^, respectively, and predicted at 1888 and 1388 cm^−1^, respectively. The stretching vibration of the pyridonic group is predicted at 1757 cm^−1^ and can be correlated to the experimental band at 1567 cm^−1^. Similarly, in the case of Eu(nal)_3_, the antisymmetric stretching of the carboxylate moiety is overestimated by MOPAC at 1882 and 1862 cm^−1^ vs. the experimental position at 1615 cm^−1^_,_ and the symmetric stretching of the same group predicted at 1381 cm^−1^ can be found at 1441 cm^−1^ in the experimental spectrum. Moreover, the band corresponding to the stretching of the pyridonic group was predicted at 1768 cm^−1^ and observed at 1558 cm^−1^.

The electronic spectra of nalidixic acid and its metal complexes in DMSO, as well as the frontier molecular orbitals (MO), were computed for the optimized ground-state geometries using TD-DFT calculations in the Orca program suite using the PBE0 functional with the CPCM solvation model and the RIJCOSX approximation.

Figure S7 and Table S8 compile the results of the simulations in comparison with the experimental spectra. The UV spectrum of nalidixic acid presents a long-wavelength, broad band (maximum at 335 nm experimental vs. 291 nm predicted), and an intense absorption band observed at 253 nm and computed at 270 nm. The short-wavelength bands deviate from experiment by 14–49 nm to higher wavelengths, whereas the long-wavelength bands are underestimated by 6–36 nm.

**Figure 5:**
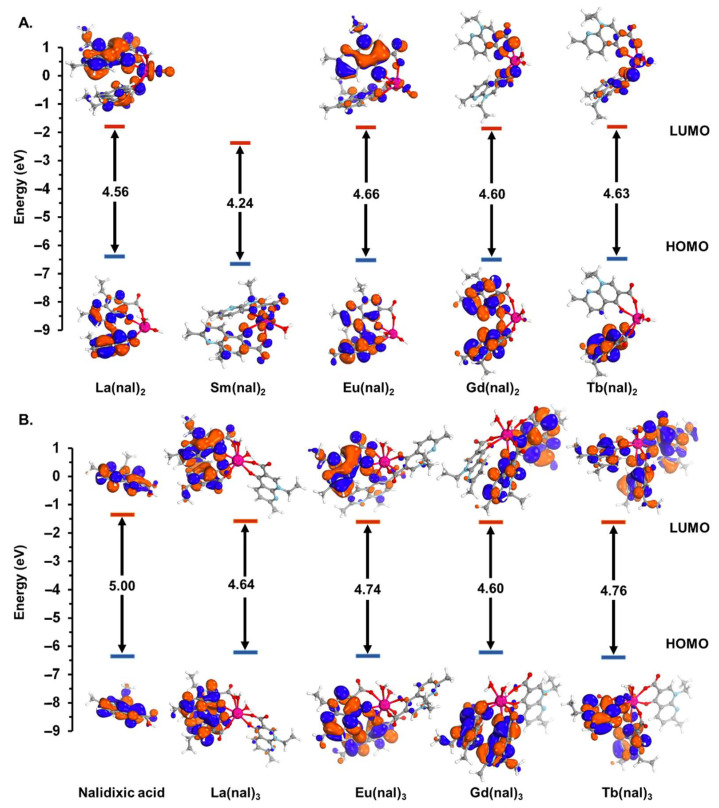
Plotted spatial distributions of the HOMO and LUMO and the HOMO–LUMO energy gaps computed for isolated molecules belonging to the M(nal)_2_ (**A**) and the M(nal)_3_ (**B**) series in DMSO using Orca program suite with PBE0 functional, with the RIJCOSX approximation and the CPCM solvation model. Red and blue isosurface colors denote + and − nodes, respectively.

The frontier MO surface plots for nalidixic acid (Figure 5B) show that electron densities are widely dispersed around the heteroatoms and the unsaturated carbons. The highest occupied molecular orbital (HOMO) energy value calculated for nalidixic acid (−6.724 eV) is lower than its lanthanide complexes (−6.712–−6.385 eV), with the exception of Tb(nal)_3_ (−6.765 eV). At the same time, the energy calculated for the lowest unoccupied molecular orbital (LUMO) is larger for nalidixic acid (−1.720 eV) than its metal complexes (−1.816–−2.400 eV) (Figure 5). The HOMO–LUMO energy gap (ΔE) was calculated as ΔE = E_LUMO_ − E_HOMO_ [58]. The value computed for the free ligand is greater than the metal complexes, indicating that complexation decreases system stability. The ΔE values are similar for all complexes, with generally higher values for M(nal)_3_ in comparison with their M(nal)_2_ correspondents.

### 2.4. Cytotoxicity Studies

Two cancer cell lines, namely MDA-MB-231 (human breast adenocarcinoma) and LoVo (human colorectal adenocarcinoma), and one normal cell line (HUVEC, human umbilical vein endothelial cells) were used to evaluate the cytotoxic effects of the complexes and the free ligand; cisplatin (Cis-Pt) and adriamycin (ADR) were used as positive controls. Cytotoxic activities for all complexes are presented in Figure S8, the calculated IC_50_ values in Table 1 and the corresponding plots in Figure S9. It should be noted that for the LoVo cell line the cytotoxic effects are more pronounced after 48 h in comparison with 24 h post treatment. Therefore, IC_50_ values were calculated taking into the account the results after 48 h. For HUVEC, however, the cytotoxic effects were more pronounced after 24 h (Figure S8).

All compounds had low activity against MDA-MB-231 cells (Figure S8), with IC_50_ values higher than 200 μM. However, against the human colon cancer cell line LoVo, La(nal)_2_, Tb(nal)_3_, Eu(nal)_3_ and Gd(nal)_3_ displayed good activity, with lower IC_50_ values than Cis-Pt (IC_50_ = 38.55 ± 4.63 μM). Importantly, most complexes were shown to have stronger activity than nalidixic acid alone on this cell line. The exceptions are Eu(nal)_2_, Gd(nal)_2_ and Tb(nal)_2_. Moreover, apart from Gd(nal)_3_, all compounds displayed lower toxicity against the normal cell line than cisplatin.

### 2.5. DNA Binding Studies

#### 2.5.1. Stability Studies

The stability of the compounds was assessed in the same working conditions as for the CT-DNA and protein interaction studies (Tris-HCl buffer, pH = 7.4, the complexes were dissolved in DMSO and diluted with Tris-HCl buffer). The recorded spectra for the highest tested concentration (20 µM) are shown in Figure S10 and corresponding data are presented in Table S9. No significant changes in peak absorption and position can be observed. Therefore, we can consider the complexes to be stable in the tested conditions and time frame.

#### 2.5.2. UV–Vis Spectroscopy

**Figure 6:**
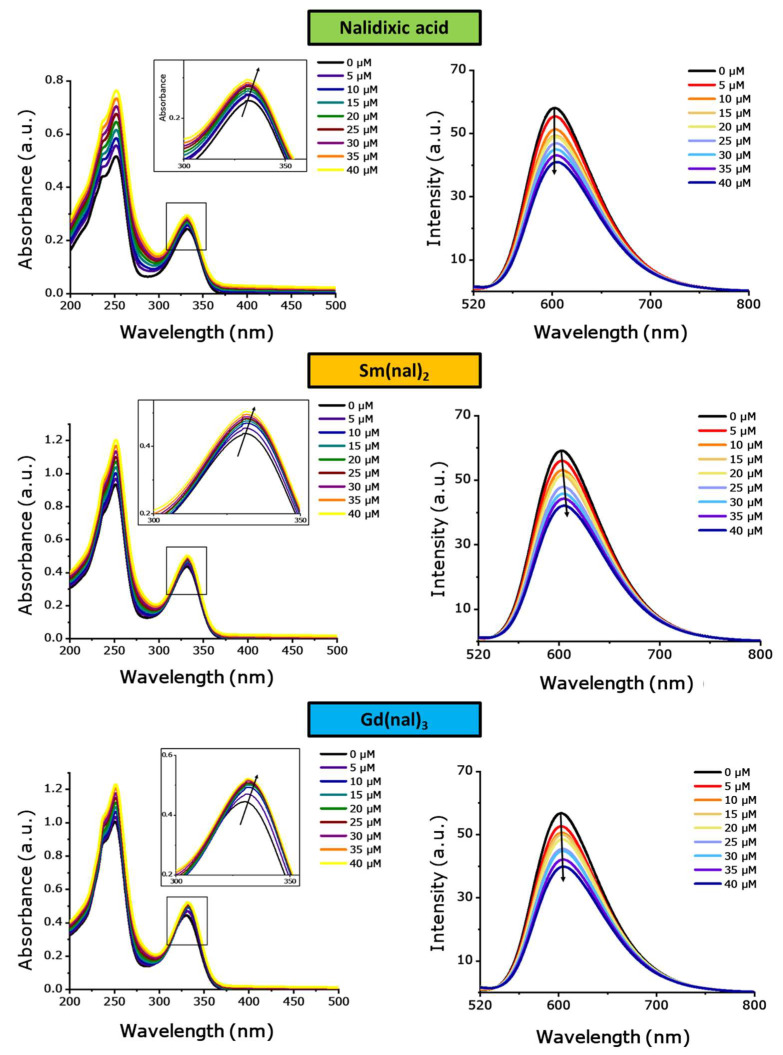
(**left**) Absorption spectra of nalidixic acid, Sm(nal)_2_ and Gd(nal)_3_ in the presence and absence of increasing amounts of DNA: [compound] = 20 μM; [DNA] = 0, 5, 10, 15, 20, 25, 30, 35, 40 μM. The arrow indicates the change in absorption upon increasing the DNA concentration. (**right**) Fluorescence spectra of the EB–DNA system in the absence and presence of increasing amounts of the tested compound. λ_ex_ = 500 nm, [EB] = 2 μM, [DNA] = 10 μM, [compound] = 0, 5, 10, 15, 20, 25, 30, 35, 40 μM. The arrows indicate the changes in fluorescence intensities upon increasing the concentration of the tested compounds.

Changes in the UV–Vis spectra of the compounds (tested at 20 µM concentrations) were monitored as CT-DNA was added stepwise (in concentrations of 5–40 µM) (Figure 6 and Figure S11). For all complexes, the spectra show small shifts (2–3 nm) of the absorption bands, as well as significant increase in absorption.

The values of the binding constants, *K_b_*, were calculated (Equation (2), Table 2 and Figure S12) and used to evaluate the strength of the interaction between the studied compound and double-stranded DNA. In the 1:2 series of compounds, the maximum value corresponds to Sm(nal)_2_ ((2.18 ± 0.76) × 10^5^ L∙mol^−1^) and decreases in the following order: Sm(nal)_2_ > Gd(nal)_2_ > La(nal)_2_ > Eu(nal)_2_ > Tb(nal)_2_, to a minimum of (0.37 ± 0.04) × 10^5^ L∙mol^−1^. The *K_b_* values observed amongst the 1:3 series are similar, with a maximum registered for Gd(nal)_3_, (4.17 ± 1.72) × 10^5^ L∙mol^−1^, decreasing in the order Gd(nal)_3_ > Eu(nal)_3_ > Tb(nal)_3_, with a drop observed for La(nal)_3_ ((0.75 ± 0.17) × 10^5^ L∙mol^−1^). Notably, the free ligand displayed higher binding affinity, with *K_b_* = (4.30 ± 2.22) × 10^5^ L∙mol^−1^ in comparison with the complexes.

#### 2.5.3. Fluorescence Spectroscopy

For these studies, ethidium bromide (EB, 3,8-diamino-5-ethyl-6-phenylphenanthridinium bromide) was used as a fluorescent dye for DNA. The emission spectra of the EB–DNA system recorded in the absence and presence of the studied complexes are presented in Figure S13. The quenching effect observed for all compounds suggests EB displacement from the EB–DNA system. The data was used to calculate the Stern–Volmer constant (*K_SV_*, Equation (3)) and the half maximum binding constant (*K_50_*, Equation (4))—data summarized in Table 2 and graphically represented in Figures S14 and S15, respectively.

The *K_SV_* values for the two series of complexes are similar, the Gd(III) complexes presenting the highest values in both series: (10.47 ± 0.13) × 10^3^ for Gd(nal)_2_ and (9.86 ± 0.19) × 10^3^ for Gd(nal)_3_. In the 1:2 complex series, the *K_SV_* value decreases in the order Gd(nal)_2_ > Sm(nal)_2_ > Tb(nal)_2_ > La(nal)_2_ > Eu(nal)_2_ to a minimum of (8.35 ± 0.13) × 10^3^, whereas in the 1:3 complex series the order is Gd(nal)_3_ > Eu(nal)_3_ > Tb(nal)_3_ > La(nal)_3_ to a minimum of (2.48 ± 0.44) × 10^3^. The free ligand is characterized by a *K_SV_* value close to the ones corresponding to the complexes.

The half maximum binding constant (*K_50_*) was found to be highest for nalidixic acid, 50.41 ± 9.60 µM. In the 1:2 series the affinity of the tested compounds for the EB–DNA complex follows the order Sm(nal)_2_ (31.35 ± 4.21 µM) > La(nal)_2_ > Gd(nal)_2_ > Tb(nal)_2_ > Eu(nal)_2_ (34.75 ± 3.69 µM), whereas in the 1:3 series the order is Eu(nal)_3_ (30.96 ± 10.85 µM) > Gd(nal)_3_ > Tb(nal)_3_ > La(nal)_3_ (36.45 ± 16.53 µM).

### 2.6. HSA and Apo-Tf Binding Studies

#### 2.6.1. Studies Regarding the Fluorescence Quenching Mechanism

**Figure 7:**
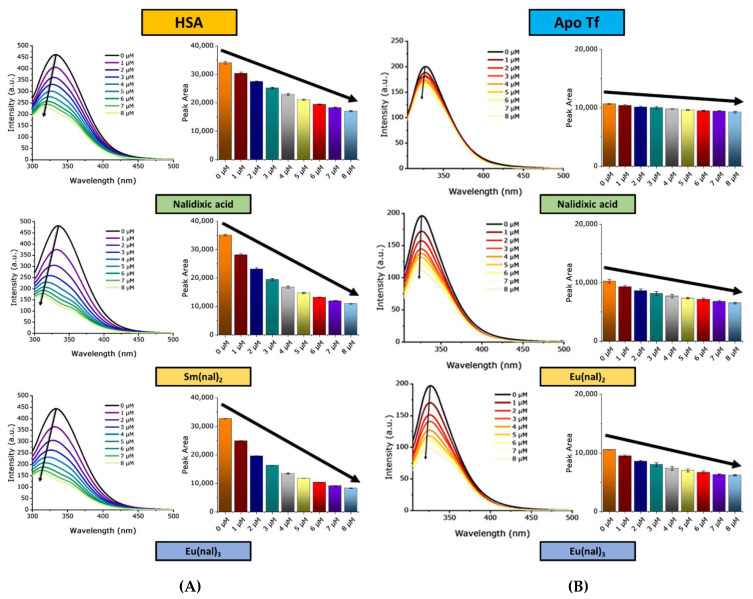
The changes observed in the fluorescence spectra of the free proteins (HSA and apo-Tf) upon addition of increasing amounts of complexes: (**A**) [HSA] = 2.5 μM, [complex] = 0, 1, 2, 3, 4, 5, 6, 7, 8 μM; (**B**) [apo-Tf] = 1 μM, [HSA] = 2.5 μM, [complex] = 0, 1, 2, 3, 4, 5, 6, 7, 8 μM. The black arrows indicate the decrease of the peak with increasing concentrations of complex.

The HSA peak showed considerable shifts (Figure 7 and Figure S16) from 334 nm to 309–310 nm due to the interaction with the M(nal)_3_ complexes and a significant quenching effect (>75%). Likewise, the M(nal)_2_ series displayed a similar effect, causing a shift of the maxima from 334 nm to 310–312 nm; the quenching effect is largest for Sm(nal)_2_ (~73%) and decreases within the series to 67 percent, the lowest value corresponding to Eu(nal)_2_.

For apo-Tf (Figure 7 and Figure S17), the quenching effect and the shift of the peak recorded at 327 nm are less pronounced. For both series, the maximum effect corresponds to the Eu(III) complex, dropping in the following order: Eu(nal)_3_ (48%) > Tb(nal)_3_ > Gd(nal)_3_ > La(nal)_3_ (31%), and Eu(nal)_2_ (41%) > Tb(nal)_2_ > La(nal)_2_ > Gd(nal)_2_ > Sm(nal)_2_ (35%), respectively. Variations in peak areas follow the same trend as the peak intensities for both proteins (Figure 6, Figure 7, Figures S18 and S19). 

The Stern–Volmer plots (F_0_/F vs. [Q]) and modified Stern–Volmer plots (F_0_/(F_0_-F) vs. [Q]) for the studied interactions were used to calculate the Stern–Volmer quenching constant (K_SV_*) and the bimolecular quenching constant (*K_q_*) from Equations (5) and (6), respectively, and are presented in Table 3 and Figures S20 and S21. For apo-Tf, the free ligand and complexes present similar values for K_SV_*, in the range of 10^5^, which drop in the following order for the 1:3 series: Tb(nal)_3_((2.70 ± 0.11) × 10^5^ M^−1^) > Eu(nal)_3_ > Gd(nal)_3_ > La(nal)_3_ ((1.04 ± 0.13) × 10^5^ M^−1^). A similar trend can be noticed for the 1:2 series, with values decreasing as follows: Tb(nal)_2_ ((2.23 ± 0.15) × 10^5^ M^−1^) > Eu(nal)_2_ > Sm(nal)_2_ > Gd(nal)_2_ > La(nal)_2_((0.44 ± 0.23) × 10^5^ M^−1^). The *K_q_* values are analogous for both series and vary in the same manner as the K_SV_* constant. 

K_SV_* values calculated for the compounds from the experiment involving HSA are similar to those corresponding to apo-Tf. The minimum value amongst the 1:3 series corresponds to Tb(nal)_3_ (2.39 ± 0.15) × 10^5^ M^−1^) and decreases as such: Eu(nal)_3_((2.89 ± 0.11) × 10^5^ M^−1^) > La(nal)_3_ > Gd(nal)_3_ > Tb(nal)_3_; K_SV_* values are also very close within the 1:2 series: Sm(nal)_2_ ((2.50 ± 0.12) × 10^5^ M^−1^) > Tb(nal)_2_ > Eu(nal)_2_ > Gd(nal)_2_ > La(nal)_2_((1.09 ± 0.02) × 10^5^ M^−1^).

The association binding constant (*K_a_*, M^−1^) and number of binding sites (n) were also calculated using Equation (7) and are presented in Table 3. The complexes with the highest affinities for apo-Tf are Gd(nal)_3_ ((2.63 ± 0.39) × 10^4^ M^−1^) and Sm(nal)_2_ ((10.17 ± 6.14) × 10^4^ M^−1^). In turn, Tb(nal)_3_ ((31.26 ± 9.66) × 10^5^ M^−1^) and Gd(nal)_2_ ((15.52 ± 2.93) × 10^5^) present the highest affinities for HSA. The lowest *K_a_* values registered correspond to the free ligand, indicating an increase in affinity after coordination. The number of binding sites (n) fluctuates around 1 for all complexes and both proteins.

The values obtained for *K_SV_* in the experiment involving HSA are similar to those corresponding to apo-Tf. The minimum value amongst the 1:3 series corresponds to Tb(nal)_3_ (2.39 ± 0.15) × 10^5^ M^−1^) and decreases as such: Eu(nal)_3_((2.89 ± 0.11) × 10^5^ M^−1^) > La(nal)_3_ > Gd(nal)_3_ > Tb(nal)_3_; *K_a_* values are also very close within the 1:2 series: Sm(nal)_2_ ((2.50 ± 0.12) × 10^5^ M^−1^) > Tb(nal)_2_ > Eu(nal)_2_ > Gd(nal)_2_ > La(nal)_2_((1.09 ± 0.02) × 10^5^ M^−1^).

The association binding constant (*K_a_*, M^−1^) and number of binding sites (n) were also calculated using Equation (7). *K_a_* values (Table 3, Figures S22 and S23) indicate good protein binding, but a slightly higher affinity towards HSA. The complexes with the highest affinities for apo-Tf are Gd(nal)_3_ ((2.63 ± 0.39) × 10^4^ M^−1^) and Sm(nal)_2_ ((10.17 ± 6.14) × 10^4^ M^−1^). In turn, Tb(nal)_3_ ((31.26 ± 9.66) × 10^5^ M^−1^) and Gd(nal)_2_ ((15.52 ± 2.93) × 10^5^) present the highest affinities for HSA. The lowest *K_a_* values registered correspond to the free ligand, indicating an increase in affinity after coordination. The number of binding sites (n) fluctuates around 1 for all complexes interacting with both proteins.

Equation (8) was used to determine the dissociation constant (*K_d_*) (Figures S22 and S23). All of the obtained values are below 9 µM for both HSA and apo-Tf, which indicates an optimal interaction between the complexes and the proteins. Most values calculated for the Hill coefficient are higher than one, indicating a positively cooperative binding [59]; the exceptions are La(nal)_3_ with a value of 1.04 ± 0.19, indicating a noncooperative binding to apo-Tf, and Eu(nal)_2_, Tb(nal)_2_ and Tb(nal)_3_ with values lower than the unity, indicative of negatively cooperative binding with apo-Tf [59].

#### 2.6.2. Studies on Conformational Changes of HSA and apo-Tf Due to Interaction with the Tested Compounds

Synchronous fluorescence spectra were collected in the range 250–400 nm with wavelength intervals of Δλ = 15 nm and Δλ = 60 nm, where Δλ represents the difference between the emission wavelength (λ_em_) and the excitation wavelength (λ_ex_) and are presented in Figures S24–S27. Changes in the vicinity of the tyrosine and tryptophan residues were monitored_._ Bathochromic shifts are observed for the examined compounds upon interaction with apo-Tf when using Δλ = 60 nm. No changes could be observed in the other synchronous spectra.

## 3. Discussion

### 3.1. Structural Characterization

Five novel M^3+^ complexes (M = La^3+^, Sm^3+^, Eu^3+^, Gd^3+^, Tb^3+^) in a 1:2 metal:ligand molar ratio (M(nal)_2_) and four M^3+^ complexes (M = La^3+^, Eu^3+^, Gd^3+^, Tb^3+^) in a 1:3 molar ratio (M(nal)_3_) were synthesized starting from nalidixic acid. The complexes were isolated as off-white powders and their structure was characterized using elemental analysis, spectroscopic studies (UV–VIS–NIR, FT-IR, high-resolution mass spectrometry, 1H-NMR), conductivity measurements, and thermal analysis.

The UV–Vis–NIR spectra provide evidence of metal binding to nalidixic acid (Figure 2). The peaks at 255 nm and 315 nm in the spectrum of the free ligand can be assigned to the absorption of the aromatic ring, and the n→π* (HOMO–LUMO) transitions, respectively [60]. Upon coordination, due to metal quinolone interaction associated with intra-ligand transitions, these peaks suffer shifts [60], in the range of 5–10 nm for the short-wavelength band and 15–40 nm for the long-wavelength band relative to the free ligand. The larger shift of the long-wavelength band is consistent with the involvement of the C=O carboxylic and C=O pyridonic groups in coordination [37,56]. The bands at 685 nm in the spectra of the complexes can be assigned to a metal to ligand charge transfer (MLCT) transition [37]. Furthermore, in the spectrum of Sm(nal)_2_, specific absorption bands in the NIR region can be observed, which can be assigned to f-f transitions from the ground state ^6^H_5/2_ to ^6^F_5/2_ (1395 nm) and ^6^H_5/2_ to ^6^F*_3/2_ (1500 nm) of the Sm^3+^ ion (4f^5^) [61,62] (Figure 2A). For all complexes, the weak bands between 1400–2000 nm can be attributed to the presence of coordinated or lattice H_2_O molecules in their structure [63].

Fourier transform infrared spectroscopy was used to further assess the binding mode of nalidixic acid and the lanthanide cations (Figure S1). In the spectrum of the free ligand, two sharp bands appear at 1701 cm^−^^1^ and 1613 cm^−1^ corresponding to υC=O carboxylic and υC=O pyridonic, respectively. Deprotonation of the carboxylic acid moiety followed by coordination of the carboxylate group to the lanthanide cation can explain the absence of the former band and the emergence of two additional bands in the spectra of the complexes; these new bands can be assigned to the O-C-O asymmetric (O-C-Oas) and O-C-O symmetric (O-C-Os) stretching vibrations. In addition, the binding mode of the carboxylate group to the metal center was determined by comparing the calculated Δ values (υO-C-Oas - υO-C-Os) for the complexes (Δ = 173–176) with that of the sodium salt of the ligand (Δ = 194). Our results indicate that nalidixic acid most likely acts as a bidentate ligand in all complexes, via two oxo groups corresponding to one carboxylate and one pyridonic oxygen atom [64].

The decomposition patterns of M(nal)_2_ (Figure S3) and M(nal)_3_ (Figure S4) show mass losses in three (most complexes), or four (La(nal)_2_) steps (summarized in Tables S4 and S5). The first two steps involve endothermic elimination of the lattice water molecules, followed by exothermic loss of coordinated water and exothermic decomposition of the hydroxide groups resulting in further elimination of water molecules. Notably, the loss of H_2_O molecules in distinct steps at different temperatures suggests that the water molecules exist in the complexes both in the lattice structure and to complete the coordination sphere of the metal ion. The last step involves oxidative degradation of the organic frame with the formation of the most stable oxide [55]. For most complexes, the overall mass reduction is consistent with the formation of M_2_O_3_ as the final residue, whereas for both Tb(nal)_2_ and Tb(nal)_3_, Tb_4_O_7_ is produced.

The ^1^H NMR spectrum of La(nal)_3_ supports the results presented so far (see Section 2. Results, for peak positions and assignments). The characteristic signal for the proton of -COOH can be seen at δ = 14.89 ppm in the spectrum of the free ligand (Figure S5). Conversely, the -COOH proton disappears entirely in the ^1^H NMR spectrum of La(nal)_3_ (Figure S5), which indicates that the coordination of nalidixic acid to La(III) occurs via the deprotonated carboxylic group. It should be noted that due to the presence of uncoupled f electrons, the Sm(III), Eu(III), Gd(III) and Tb(III) complexes are paramagnetic and their NMR spectra display signals that are too broad to be interpreted.

Based on all experimental data, the following formulas have been attributed to the complexes: [M(nal)_2_(OH)(H_2_O)], where M = La^3+^, Sm^3+^, Eu^3+^, Gd^3+^, Tb^3+^, for the 1:2 molar ratio complexes, and [M(nal)_3_(H_2_O)_2_]·nH_2_O for complexes with a 1:3 molar ratio, where M = La^3+^, Eu^3+^, Gd^3+^, Tb^3+^. Moreover, the molar conductance values for the lanthanide complexes of nalidixic acid in DMSO (1.00 × 10^−3^ M) were found to be in the range of 2.2–9.5 Ω^−1^·cm^2^·mol^−1^ at 25 °C, suggesting them to be non-electrolytes (see Section 4. Materials and Methods), in agreement with the proposed structures. These formulas were then matched with various possible starting conformations, for which geometry optimizations were carried out.

### 3.2. Computational Studies

For all complexes the M-O(nal) and M-OH_2_ bond lengths are similar to those reported for other metal–quinolone complexes [38,65,66] and vary in the range 2.256–2.625 Å. Generally, predicted M-O(nal) bond lengths drop with decreasing lanthanide ion size, from La(III) to Tb(III). The shorter (roughly 1.5 Å) M-O(H) bonds in M(nal)_2_ are likely due to the smaller size of the ligand. The optimized geometries of the complexes belonging to the M(nal)_2_ series correspond to a distorted octahedral geometry for La(nal)_2_, Sm(nal)_2_ and Eu(nal)_2_. For Gd(nal)_2_ and Tb(nal)_2_, however, the larger values predicted for the O(H)-M-O(nal) angles and the dihedral angles between two nalidixate moieties indicate a distorted pentagonal pyramid coordination environment (Table S6). In the M(nal)_3_ complexes, on the other hand, the metal center is octacoordinated in a square antiprismatic geometry (Figure 4). Predicted vibrational spectra are given in Figure S1 and selected data and assignments for Eu(nal)_2_ and Eu(nal)_3_ are detailed in Table S7. All bands from the experimental spectra are reproduced in the predictions. However, the broad bands around 3395 cm^−1^ corresponding to O-H stretches in the experimental IR spectra of the complexes are underestimated in the predicted spectra (Figure S1). Of note, previous findings also showed that PM7 systematically underestimates frequencies of vibration above 2500 cm^−1^ [57]. Stretching vibrations over 1500 cm^−1^ are overestimated in the predicted spectra, whereas the symmetric stretching vibrations of the carboxylate moiety are slightly underestimated (see Section 2.3 Computational Studies for more details).

TD-DFT calculations were used to predict the electronic spectra of nalidixic acid and its metal complexes in DMSO (Figure S7 and Table S8), as well as the frontier molecular orbitals. In the electronic spectra, the long-wavelength, broad band of nalidixic acid (maximum at 335 nm experimental vs. 291 nm predicted) results from the n→π* electronic transition, whereas the intense absorption band observed at 253 nm and computed at 270 nm is attributed to the intraligand π→π* transition [67]. Of note, the experimental UV spectra of the complexes in DMSO are nearly identical to that of nalidixic acid, with only small band shifts, which is expected for coordination environments involving the pyridonic O and one carboxylic O atom [68]. The experimental bands are generally reproduced by our predictions, with deviations from experiment of 6–49 nm, which are within the expected accuracy.

The frontier MO surface plots (Figure 5) show that electron densities are widely dispersed around the heteroatoms and the unsaturated carbons, as a result of n→π* and π→π* electronic transitions. The HOMO energy value calculated for nalidixic acid is lower than its lanthanide complexes. In agreement with the literature data [69], complexes in which the metal center is represented by elements with an empty (La) or half-occupied (Gd) f shell have lower HOMO energies and are thus predicted to be more stable. At the same time, the energy calculated for the LUMO is larger for nalidixic acid than its metal complexes. The low-lying LUMO levels of the metal complexes indicate higher affinity for electrons and better π–π stacking ability. Additionally, the HOMO–LUMO energy gap, calculated as E_LUMO_ − E_HOMO_, was used to provide information on the stability and reactivity of the chemical systems [58]. The value computed for the free ligand is greater than the metal complexes, suggesting that complexation decreases system stability. Furthermore, the calculated HOMO–LUMO energy gaps for all complexes are similar, with generally greater values for the M(nal)_3_ series, which are therefore predicted to be slightly more stable and thus less reactive than their 1:2 correspondents.

### 3.3. Cytotoxicity Studies

The cytotoxic effects of the complexes and the free ligand were tested on two cancer cell lines, MDA-MB-231 (human breast adenocarcinoma) and LoVo (human colorectal adenocarcinoma), and one normal cell line (HUVEC, human umbilical vein endothelial cells); cisplatin (Cis-Pt) and adriamycin (ADR) were used as positive controls (Figure S8). Whereas the cytotoxic activity on the MDA-MB-231 cells was low (Figure S8), several complexes showed good activity against the LoVo cell line. The most active compounds against the latter, with lower IC_50_ values than cisplatin, are La(nal)_2_ (IC_50_ = 25.08 ± 9.11 μM), Tb(nal)_3_ (IC_50_ = 25.55 ± 6.97 μM), Eu(nal)_3_ (IC_50_ = 26.88 ± 5.72 μM) and Gd(nal)_3_ (IC_50_ = 28.36 ± 7.95 μM) (Table 1). Calculated IC_50_ values for La(nal)_3_ and Sm(nal)_2_ are 49.43 ± 7.92 μM and 92.39 ± 15.97 μM, respectively, whereas nalidixic acid, Eu(nal)_2_, Gd(nal)_2_ and Tb(nal)_2_ were not toxic. It is evident that the M(nal)_3_ series were generally more active than their M(nal)_2_ counterparts, except for the La(III) complexes. Moreover, most complexes were essentially non-toxic to the normal HUVEC cell line, which is a significant advantage over Cis-Pt. Gd(nal)_3_ was the only compound of the series which displayed higher toxicity against HUVEC when compared with Cis-Pt.

The most active complexes reported in this work generally display more potent activity in comparison with other metal complexes of nalidixic acid reported in the literature. For instance, Cu(II) complexes displayed an IC_50_ value > 200 μM on A549 non-small lung carcinoma cells [70], and an IC_50_ = 192.4 μM on MCF-7 human breast cancer cells [38]. Additionally, an Ru-(arene) complex was essentially non-toxic to human A549, CH1 (ovarian carcinoma) and SW480 (colon carcinoma) cells [35]. However, heteroleptic Cu(II) complexes bearing nalidixic acid and diimine ligands displayed significantly lower IC_50_ values of 3.8–7.5 μM on MCF-7 cells [37]. Nevertheless, the toxicity of these complexes on normal cells was not assessed, therefore the increased activity might be associated with an increase in toxicity to healthy cells.

**Figure 8:**
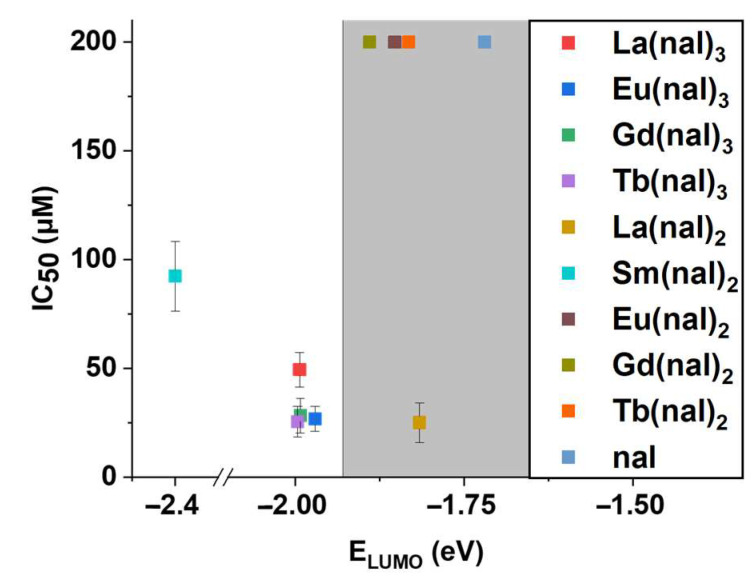
Graphical representation of IC_50_ (µM) vs. E_LUMO_ (eV); IC_50_ values were calculated for the LoVo cell line, and results > 200 µM were attributed the value 200 on the graph; grey area shows compounds with low activity (note that La(nal)_2_ represents the exception).

Interestingly, we noticed that compounds with higher LUMO energy values generally displayed low activity on the LoVo cell line (IC_50_ > 200 μM) (Figure 8). This observation agrees well with the fact that lower LUMO levels indicate higher affinity for electrons and thus higher reactivity. It should be noted that correlations between electronic properties including E_LUMO_, E_HOMO_, or ΔE and IC_50_ values have been previously reported by various groups (for instance [71,72,73]). Note that La(nal)_2_, with an IC_50_ value of 25.08 ± 9.11 represents the exception (Figure 8). Moreover, Sm(nal)_2_, with the lowest LUMO energy of the series, displays a relatively low activity (IC_50_ = 92.39 ± 15.97). In an attempt to further investigate this and given that we started from the assumption that DNA is the intracellular target for these complexes, we investigated their abilities to bind to DNA in order to formulate a putative mechanism of action.

### 3.4. DNA Binding Studies

Quinolones have been reported to inhibit DNA synthesis via interfering with the normal activity of DNA topoisomerases II (DNA gyrase) [74,75] and IV [76,77], a process that induces bacteriostasis [78], double-strand breaks, chromosome fragmentation, formation of reactive oxygen species (ROS) [78,79] and eventually, cell death. Therefore, assessing the ability of the complexes to interact with DNA can provide preliminary information regarding their mechanism of action, since quinolone metal complexes have been previously shown to possess DNA-binding properties [37,38,70,80].

Three major modes of interaction between metal complexes and the DNA macromolecule have been described: (i) electrostatic interactions with the negatively charged sugar-phosphate structure, (ii) minor or major groove binding through van der Waals forces, hydrogen bonds, electrostatic forces or hydrophobic interactions and (iii) intercalation between base pairs, a process stabilized by π-π* stacking interactions between the aromatic systems of the ligand and DNA bases.

Effective drug binding to DNA induces changes in its UV–Vis spectrum, such as hypo-, hyper-, hypso- (blue), or batho- (red) chromic shifts, which are well documented in the literature [61,81,82]. As a result, we looked at changes in the UV–Vis spectra of the complexes as CT-DNA was added stepwise (Figure 6 and Figure S11). Notably, the superior value corresponding to the free ligand, (4.30 ± 2.22) × 10^5^ L∙mol^−1^, suggests that complexation does not improve the affinity for DNA. Overall, the changes observed are similar for all tested compounds and the *K_b_* values indicate the presence of similar interactions of moderate intensity between the tested compounds and DNA. Although the *K_b_* values are rather high (Table 2), indicating the existence of interactions between the complexes and DNA, the hyperchromism and lack of blue or red shifts suggest that groove binding rather than intercalation is the mechanism for these interactions [70]. 

Competitive binding studies (Figure S13) with EB give insight into the manner of binding to the DNA macromolecule. EB is a fluorescent dye with an aromatic scaffold that can intercalate between two base pairs in the DNA structure and form a stable complex capable of emitting fluorescent light. Displacing EB from this complex results in a quenching effect [61,83]. The computed *K_SV_* values (Table 2) are consistent with those reported for modest intercalating agents [84,85], which supports our previous claim that the main mechanism of interaction is most likely groove binding. The free ligand is characterized by a *K_SV_* value close to the complexes, suggesting that complexation has little influence on the binding process. Furthermore, the *K_50_* constant characterizes the affinity of the compounds for the EB–DNA complex and the equilibrium of the interaction. The lower the *K_50_* value, the higher the compounds’s affinity for DNA and the higher its capacity to bind at lower concentrations [86]. According to the calculated *K_50_* values (Table 2), nalidixic acid displays the lowest affinity for the EB–DNA system, whereas the affinity of the systems in the 1:2 series follows the order Sm(nal)_2_ (31.35 ± 4.21 µM) > La(nal)_2_ > Gd(nal)_2_ > Tb(nal)_2_ > Eu(nal)_2_ (34.75 ± 3.69 µM). For the 1:3 series, *K_50_* values decrease in the order Eu(nal)_3_ (30.96 ± 10.85 µM) > Gd(nal)_3_ > Tb(nal)_3_ > La(nal)_3_ (36.45 ± 16.53 µM).

Lower LUMO energies are correlated with a greater propensity to accept electrons from the HOMO of the DNA base pairs with which it interacts. In turn, this affects the degree of π-stacking interactions between the electron acceptor molecule and the electron donor base pairs, which can determine the DNA binding affinity of the molecule ([37] and citations therein). However, nalidixic acid, with the highest LUMO energy (vide supra) has a higher binding constant than its complexes. This finding suggests that, for these compounds, the mechanism of binding to DNA does not rely on π-stacking interactions, possibly due to steric hindrance. Therefore, these complexes most likely bind to DNA minor/major grooves through hydrophobic interactions. This finding, together with the higher *K_b_* values calculated for the M(nal)_3_ series, with more hydrophobic methyl and ethyl groups than their M(nal)_2_ analogues, reinforce our assumption that these groups provide a hydrophobic recognition element that interacts with DNA more effectively.

The values calculated for our complexes (*K_b_* ~ 10^4^–10^5^ M^−1^) are generally in the same range of or even higher than other previously reported metal complexes of quinolones ([37] and citations therein, [87,88,89]). However, in some cases the cytotoxicity of the complexes with similar binding affinities, translated to lower IC_50_ values on breast cancer cells [37]. This finding, along with the fact that CT-DNA affinity does not correlate with the HOMO–LUMO energy gap or IC_50_ values suggests that, contrary to our first assumption, DNA is not the primary target of these compounds. Therefore, we tested the role that protein binding plays in the cytotoxic activity of the complexes. Two serum transport proteins were used to probe the interaction of these compounds with proteins.

### 3.5. HSA and Apo-Tf Binding Studies

The role of HSA and apo-Tf in the transport of metal complexes in the human body has sparked our interest in examining the interactions of the investigated complexes with HSA and apo-Tf. HSA is the most abundant protein in the human circulatory system, accounting for 80% of the osmotic pressure of colloid particles [90], and is the protein with the highest affinity for drugs, influencing their pharmacokinetic and pharmacodynamic profiles. Apo-Tf is involved in iron transport, as well as the transport of xenobiotics [91,92]. Due to the various unique receptors expressed on the surface of cancer cells, it is also being explored as a potential drug carrier for targeted delivery into these cells [93]. 

The nature of the interaction between metal complexes and proteins can be investigated by analyzing changes occurring in the fluorescence spectra of these proteins. HSA and apo-Tf present an intrinsic fluorescence due to the tryptophan residue in position 214 (Trp-214). In the fluorescence spectra of HSA and apo-Tf, a strong peak can be seen at 340 nm and 327 nm, when excited at 280 nm and 295 nm, respectively. The tryptophan residue is highly sensitive to variations in the polarity of the medium [61], which are accompanied by shifts in the aforementioned peaks. The quenching effect has been correlated with a decrease in peak areas (Figure 7, Figures S18 and S19); this decrease is more accentuated for the complexes in comparison with the free ligand and for HSA in comparison with apo-Tf. As a result, our observations point to a stronger affinity towards HSA, which has been previously observed for similar complexes [94].

The fluorescence quenching effect can be explained through a number of processes, such as molecular collision, energy transfer, excited-state reaction or ground-state complex formation. In the case of small molecules, three main processes are relevant: static quenching (involving the formation of a nonfluorescent complex between the quencher and the fluorophore in the ground state), dynamic quenching (the excited state of the fluorophore loses energy via a collisional process with the quencher) and combined static and dynamic quenching [95].

The Stern–Volmer plots (F_0_/F vs. [Q]) and modified Stern–Volmer plots (F_0_/(F_0_-F) vs. [Q]) (Figures S20 and S21) for the studied interactions provide further information. Information about the static or dynamic nature of the quenching mechanism is provided by the values of the Stern–Volmer quenching constant (K_SV_*) and the bimolecular quenching constant (*K_q_*) (Table 3, Figures S18 and S19). In the case of apo-Tf, the free ligand and complexes present similar values for K_SV_*, in the range of 10^5^. The *K_q_* values are analogous for both series, vary in the same manner as the K_SV_* constant, and are larger than those seen in the literature for quenchers of biopolymer fluorescence (2.0 × 10^10^ M^−1^s^−1^), implying a static quenching mechanism [96]. *K_a_* values (Table 3, Figures S22 and S23) indicate good protein binding, but a slightly higher affinity towards HSA. The lowest *K_a_* values for both proteins correspond to the free ligand, suggesting that metal coordination increases the affinity for serum proteins. Hence, we can speculate that the ability of the complexes to be transported through the human body is higher than that of nalidixic acid. The number of binding sites (n) fluctuates around 1 for all complexes.

The dissociation constant (*K_d_*) (Figures S22 and S23) characterizes the stability of the interaction between the studied complexes and the serum proteins. All of the obtained values are below 9 µM for both HSA and apo-Tf, which indicates an optimal interaction between the complexes and the proteins. This may result in efficient transportation throughout the human body; moreover, the values are not high enough to determine drug retention and the effects that derive from this, such as: altered plasmatic concentration, distribution, metabolism and efficacy. The bulk of calculated Hill coefficients are higher than one, indicating a positively cooperative binding [59].

The synchronous spectra (Figures S24–S27) give information on possible structural changes occurring due to interactions between proteins and the studied compounds. Changes in the vicinity of the tyrosine and tryptophan residues can be highlighted by using wavelength intervals of Δλ = 15 nm and Δλ = 60 nm_._ The shifts observed are linked to changes in the polarity of the medium surrounding the amino acids, with the tryptophan residue being particularly sensitive. Thus, a reduction in the exposure to the solvent is equivalent to a more hydrophobic environment and results in a blue shift, whereas increased exposure to the solvent creates a more polar environment and determines a red shift in the synchronous spectra [97]. Bathochromic shifts are observed for the compounds upon interaction with apo-Tf when using Δλ = 60 nm. This finding indicates that the protein adopts a different conformation as a consequence of its interaction with the complexes, one that increases the exposure of the tryptophan residue to the solvent, resulting in a more polar environment in its vicinity.

In a similar manner to the observation made for the DNA binding studies, despite being bulkier, M(nal)_3_ complexes displayed higher binding affinities for both serum proteins than their M(nal)_2_ analogues. This is likely due to the hydrophobic interactions between the methyl and ethyl groups and the proteins. The binding constants are consistent with moderate to high affinities toward transport proteins, in the same range as [38] or slightly lower than other nalidixic acid metal complexes [37,38,89].

**Figure 9:**
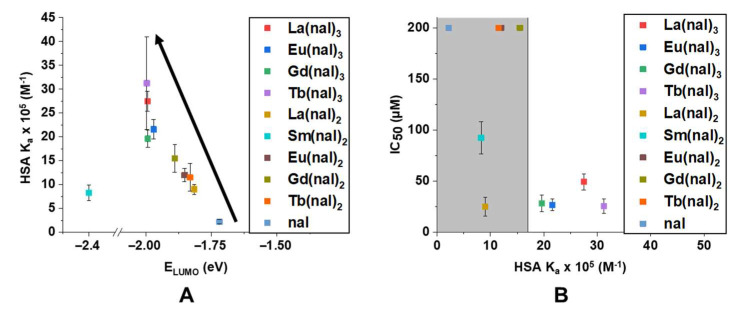
Graphical representations of (**A**) HSA *K_a_* × 10^5^ (M^−1^) vs. E_LUMO_ (eV); the black arrow indicates that generally lower LUMO energy values translate to higher HSA binding affinities (note that Sm(nal)_2_ and Gd(nal)_3_ represent the exceptions) and (**B**) IC_50_ (µM) vs. HSA *K_a_* × 10^5^ (M^−1^); IC_50_ values were calculated for the LoVo cell line, and results > 200 µM were attributed the value 200 on the graph; grey area shows compounds with low activity (note that La(nal)_2_ represents the exception).

Moreover, we plotted the values calculated for the HSA binding constants against the LUMO energy values of all compounds and we noticed that, generally, the lower the LUMO energy value, the higher the binding affinity (Figure 9A). This observation agreed with the previous finding that high LUMO values generally translate to low activity (Figure 8), hence we plotted the HSA binding constants against the IC_50_ values of the compounds (Figure 9B). In a similar manner, it can be observed that compounds with low cytotoxic activity displayed lower HSA binding affinities, with the exception of La(nal)_2_, the most active compound of the series. This suggests that interactions with proteins play a role in the activity of these compounds and that La(nal)_2_ has a different mechanism of action compared with the M(nal)_3_ series, which displayed similar binding affinities and IC_50_ values.

Therefore, future research will seek to further investigate what factors contribute to the observed link between the LUMO energy, HSA binding affinity and cytotoxic activity, and to identify the intracellular targets of these complexes. Such targets may include topoisomerases I/II, tyrosine kinases and growth factor receptors such as VEGFR and EGFR ([98] and citations therein).

## 4. Materials and Methods

All solvents and reagents were of analytical reagent grade and used without further purification. Nalidixic acid, LaCl_3_, EuCl_3_·6H_2_O, GdCl_3_·6H_2_O, SmCl_3_·6H_2_O, TbCl_3_·6H_2_O, double-stranded calf thymus DNA, human serum albumin and apotransferrin were purchased from Sigma-Aldrich Chemical Co. (Schnelldorf, Germany).

### 4.1. Synthesis

For the synthesis of the 1:2 molar ratio complexes, nalidixic acid (0.6 mmol; 0.1394 g) and solid NaOH (0.6 mmol; 0.0240 g) were dissolved in distilled water (15 mL) in a round-bottom flask under continuous stirring and heating (50 °C). The hot mixture was then added dropwise to the metal salt (0.3 mmol), while carefully maintaining the pH level at 5 by adding HCl (1 M) or NaOH (0.5 M) solutions dropwise. The pH of the final mixture was adjusted to 7 before it was refluxed for 4.5 h. The off-white precipitates were filtered and washed with distilled water and acetone and then dried in the desiccator over anhydrous calcium chloride. The 1:3 molar ratio complexes were obtained in a similar way, using 0.9 mmol (0.2090 g) of nalidixic acid, 0.9 mmol (0.0360 g) of solid NaOH and 0.3 mmol of metal salts. The purity was found to be over 90% for all complexes, according to the elemental analysis.

La(nal)_2_—La(C_12_H_11_N_2_O_3_)_2_(OH)(H_2_O)·2H_2_O, MW = 672.41 g/mol; Elemental analysis found (calculated): %C 42.35 (42.87), %H 4.45 (4.35), %N 8.09 (8.33); MS (ESI+): *m*/*z*: 601.06 ([La(nal)_2_]^+^), *m*/*z*: 679.08 ([La(nal)_2_(DMSO)]^+^), *m*/*z*: 757.09 ([La(nal)_2_(DMSO)_2_]^+^); UV–vis (nm): 1935, 1670, 335, 260; FT-IR (cm^−1^): 3725 (w, ν(O-H) coordinated water molecule), 3399 (w, (ν(O-H) lattice water), 3056 (w, ν(C-H) aromatic), 2974, 2925 (w, ν(CH_3_-CH_2_)), 1618 (s, ν(O-C-O)as), 1570 (s, ν(C=O)pyridone), 1524 (ν (C-N) pyridone), 1443 (s, ν(O-C-O)s), 578 (m, ν(M-O)).

Sm(nal)_2_—Sm(C_12_H_11_N_2_O_3_)_2_(OH)(H_2_O)·3H_2_O, MW = 701.88 g/mol; Elemental analysis found (calculated): %C 40.65 (41.07), %H 4.28 (4.45), %N 7.51 (7.98); MS (ESI+): *m*/*z*: 692.09 ([Sm(nal)_2_(DMSO)]^+^), *m*/*z*: 770.11 ([Sm(nal)_2_(DMSO)_2_]^+^); UV–vis (nm): 1940, 1500, 1395, 1245, 320, 250; FT-IR (cm^−1^): 3649 (w, ν(O-H) coordinated water molecule), 3420 (wb, (ν(O-H) lattice water), 1616 (s, ν(O-C-O)as), 1568 (s, ν(C=O)pyridone), 1523 (s, (C-N) pyridone)), 1443 (s, ν(O-C-O)s), 599 (m, ν(M-O)); Molar conductance (DMSO, Ω^−1^∙cm^2^∙mol^−1^): 3.0.

Eu(nal)_2_—Eu(C_12_H_11_N_2_O_3_)_2_(OH)(H_2_O)·3H_2_O, MW = 703.49 g/mol; Elemental analysis found (calculated): %C 40.47 (40.98), %H 4.51 (4.44), %N 7.45 (7.96); MS (ESI+): *m*/*z*: 693.09 ([Eu(nal)_2_(DMSO)]^+^), *m*/*z*: 771.10 ([Eu(nal)_2_(DMSO)_2_]^+^); UV–vis (nm): 1935, 340, 265; FT-IR (cm^−1^): 3395 (w, ν(O-H) coordinated water molecule), 3068 (w, ν(C-H) aromatic), 2981, 2931 (w, ν(CH_3_-CH_2_)), 1615 (s, ν(O-C-O)as), 1567 (s, ν(C=O)pyridone), 1523 (s, ν (C-N) pyridone), 1442 (s, ν(O-C-O)s), 559 (m, ν(M-O)); Molar conductance (DMSO, Ω^−1^∙cm^2^∙mol^−1^): 2.3.

Gd(nal)_2_—Gd(C_12_H_11_N_2_O_3_)_2_(OH)(H_2_O)·3H_2_O, MW = 708.77 g/mol; Elemental analysis found (calculated): %C 41.07 (40.67), %H 4.69 (4.41), %N 7.21 (7.90); MS (ESI+): *m*/*z*: 620.08 ([Gd(nal)_2_]_3_^3+^), *m*/*z*: 698.09 ([Gd(nal)_2_(DMSO)]_2_^2+^), *m*/*z*: 776.11 ([Gd(nal)_2_(DMSO)_2_]+); UV–vis (nm): 1935, 330, 255; FT-IR (cm^−1^): 3377 (wb, ν(O-H) coordinated water molecule), 2981, 2931 (w, ν(CH_3_-CH_2_)), 1614 (s, ν(O-C-O)as), 1567 (s, ν(C=O)pyridone), 1523 (s, ν (C-N) pyridone), 1441 (s, ν(O-C-O)s), 558 (m, ν(M-O)); Molar conductance (DMSO, Ω^−1^∙cm^2^∙mol^−1^): 2.5.

Tb(nal)_2_—Tb(C_12_H_11_N_2_O_3_)_2_(OH)(H_2_O)·1.5H_2_O, MW = 683.43 g/mol; Elemental analysis found (calculated): %C 42.78 (42.18), %H 4.78 (4.13), %N 7.57 (7.20); MS (ESI+): *m*/*z*: 621.08 ([Tb(nal)_2_]_3_^3+^), *m*/*z*: 699.09 ([Tb(nal)_2_(DMSO)]^+^), *m*/*z*: 777.11 ([Tb(nal)_2_(DMSO)_2_]^+^); UV–vis (nm): 1940, 345, 265; FT-IR (cm^−1^): 3397 (wb, ν(O-H) coordinated water molecule), 2981, 2932 (w, ν(CH_3_-CH_2_)), 1615 (s, ν(O-C-O)as), 1568 (s, ν(C=O)pyridone), 1523 7(s, ν (C-N) pyridone), 1442 (s, ν(O-C-O)s), 602 (w, ν(M-O)); Molar conductance (DMSO, Ω^−1^∙cm^2^∙mol^−1^): 2.2.

La(nal)_3_—La(C_12_H_11_N_2_O_3_)_3_(H_2_O)_2_·6H_2_O, MW = 976.71 g/mol; Elemental analysis found (calculated): %C 44.97 (44.27), %H 5.15 (5.06), %N 8.41 (8.60); MS (ESI+): *m*/*z*: 833.15 ([[La(nal)_3_]_2_+2H]^2+^); UV–vis (nm): 1945, 330, 255; FT-IR (cm^−1^): 3403 (w, ν(O-H) coordinated water molecule), 3060 (w, ν(C-H) aromatic), 2972 (w, ν(CH_3_-CH_2_)), 1614 (s, ν(O-C-O)as), 1557 (s, ν(C=O)pyridone), 1439 (s, ν(O-C-O)s), 558 (m, ν(M-O)); ^1^H NMR (500 MHz, DMSO-*d6*): δ/ppm = 8.83 (3H), 8.16 (3H), 7.28 (3H), 4.40 (6H), 2.57 (9H), 1.23 (9H); Molar conductance (DMSO, Ω^−1^∙cm^2^∙mol^−1^): 4.2.

Eu(nal)_3_—Eu(C_12_H_11_N_2_O_3_)_3_(H_2_O)_2_·2.5H_2_O, MW= 926.71 g/mol; Elemental analysis found (calculated): %C 47.08 (46.66), %H 4.55 (4.57), %N 9.48 (9.07); MS (ESI+): *m*/*z*: 693.09 ([Eu(nal)_2_(DMSO)]^+^), *m*/*z*: 771.10 ([Eu(nal)_2_(DMSO)_2_]^+^), *m*/*z*: 846.17 ([[Eu(nal)_3_]_2_+2H]^2+^); UV–vis (nm): 1945, 335, 260; FT-IR (cm^−1^): 3399 (wb, ν(O-H) coordinated water molecule), 3061 (w, ν(C-H) aromatic), 2971, 2929 (w, ν(CH_3_-CH_2_)), 1615 (s, ν(O-C-O)as), 1558 (s, ν(C=O)pyridone), 1441 (s, ν(O-C-O)s), 463 (w, ν(M-O)); Molar conductance (DMSO, Ω^−1^∙cm^2^∙mol^−1^): 3.5.

Gd(nal)_3_—Gd(C_12_H_11_N_2_O_3_)_3_(H_2_O)_2_·1.5H_2_O, MW = 913.99 g/mol; Elemental analysis found (calculated): %C 47.24 (47.31), %H 4.51 (4.41), %N 9.32 (9.19); MS (ESI+): *m*/*z*: 698.09 ([Gd(nal)_2_(DMSO)]^+^), *m*/*z*: 776.11 ([Gd(nal)_2_(DMSO)_2_]^+^), *m*/*z*: 852.16 ([[Gd(nal)_3_]+H]^+^); UV–vis (nm): 1950, 325, 255; FT-IR (cm^−1^): 3436 (wb, ν(O-H) coordinated water molecule), 2968 (w, ν(CH_3_-CH_2_)), 1615 (s, ν(O-C-O)as), 1559 (s, ν(C=O)pyridone), 1439 (s, ν(O-C-O)s), 599 (m, ν(M-O)); Molar conductance (DMSO, Ω^−1^∙cm^2^∙mol^−1^): 6.0.

Tb(nal)_3_—Tb(C_12_H_11_N_2_O_3_)_3_(H_2_O)_2_·1.5H_2_O, MW = 915.66 g/mol; Elemental analysis found (calculated): %C 47.57 (47.22), %H 4.68 (4.40), %N 9.73 (9.18); MS (ESI+): *m*/*z*: 621.08 ([Tb(nal)_2_]^+^), *m*/*z*: 699.09 ([Tb(nal)_2_(DMSO)]^+^), m/z: 777.11 ([Tb(nal)_2_(DMSO)_2_]^+^), m/z: 853.16 ([[Tb(nal)_3_]+H]^+^); UV–vis (nm): 1940, 1925, 355, 280, 245; FT-IR (cm^−1^): 3420 (wb, ν(O-H) coordinated water molecule), 3059 (w, ν(C-H) aromatic), 2970, 2932 (w, ν(CH_3_-CH_2_)), 1614 (s, ν(O-C-O)as), 1559 (s, ν(C=O)pyridone), 1439 (s, ν(O-C-O)s), 590 (w, ν(M-O)); Molar conductance (DMSO, Ω^−1^∙cm^2^∙mol^−1^): 6.5.

### 4.2. Physicochemical Characterization of the Complexes

A PE 2400 analyzer (Perkin Elmer, Billerica, MA, USA) was used for the elemental analysis of C, H, and N. On an FT-IR VERTEX 70 spectrometer (Bruker, Billerica, MA, USA), KBr pellets were used to record infrared spectra (Bruker). UV–visible–NIR spectra were recorded on solid probes, without dilution, on a V-670 spectrophotometer (Jasco, Tokyo, Japan), in the region of 200–2000 nm; spectralon was used as a reference sample. The fluorescence spectra were recorded using a Jasco FP 6500 spectrofluorometer (Jasco, Tokyo, Japan). The following parameters were used to investigate thermal behavior (TG and DTA) using a Labsys 1200 Setaram instrument (Setaram, Caluire, France): measurements were carried out in a synthetic air atmosphere with a flow rate of 16.66 cm^3^/min using an alumina crucible with an average mass of 12 mg, a temperature range of 20–1000 °C and a heating rate of 10 °C/min. For high-resolution mass spectrometry (HRMS), the complexes were dissolved in DMSO and 1:10 (V:V) dilutions in MeOH were injected and analyzed using an LTQ-Orbitrap Velos Pro (Thermo Fisher Scientific, Bremen, Germany). The ^1^H NMR spectra were acquired by a Bruker Advance Ultrashield Plus 500 spectrometer (Bruker AXS GmbH, Karlsruhe, Germany) with 500 MHz working frequency at 25 °C. Chemical shifts were measured in parts per million (ppm) relative to the internal standard TMS. Conductivity measurements were carried out with a Consort C830 (Turnhout, Belgium) conductometer with an SK10T platinum electrode embedded in glass (cell constant 1.0 cm^−1^).

### 4.3. Computational Studies

Using the semiempirical method PM7 in MOPAC version 22.0.1 (Stewart Computational Chemistry, available online at http://openmopac.net/MOPAC2016.html, accessed on 20 January 2021) [99], the optimized structures of nalidixic acid and its complexes were computed. Individual optimizations for different starting conformations were performed and the conformation with the lowest energy was chosen as the global minimum and used for further investigations. The optimized geometries were computed under no symmetry restrictions. In order to confirm that each structure corresponds to a true minimum, the absence of imaginary frequencies in the vibrational analysis was verified. ORCA 5.0.3 program suite [100] was further used for time-dependent density functional theory (TDDFT) calculations on the optimized geometries. The TDDFT computations were performed using the PBE0 functional and the conductor-like polarizable continuum model (CPCM) for solvation in DMSO. The RIJCOSX approximation (resolution-of-identity (RI-J) and the chain-of-spheres (COSX) approximations) was used to lower the computational cost. Other pieces of software used were Gauss View 6.0.16 [101] for data visualization, IboView v20211019-RevA program (source code available at http://www.iboview.org, accessed on 18 May 2022) orbital depictions, and Mercury 4.0 [102] for image capture.

### 4.4. Cytotoxicity Studies

LoVo, MDA-MB-231 and HUVEC human cell lines were purchased from American Type Culture Collection (ATCC). Cells were routinely maintained in culture either in culture flasks or seeded in plates, using Dulbecco’s modified Eagle medium/nutrient mixture F-12 (DMEM:F12) medium supplemented with 2 mL glutamine, 10% fetal calf serum (Sigma Aldrich, St. Louis, MO, USA), 100 units/mL penicillin, 100 μg/mL streptomycin and incubated at 37 °C in 5% CO_2_ humidified atmosphere.

Cytotoxicity studies were performed in 96-well microtiter plates with flat bottoms (Falcon, Teterboro, NJ, USA) using the CellTiter 96 Aqueous One Solution Cell Proliferation Assay (Promega, Madison, WI, USA), an MTS-based colorimetric assay. Metabolically active cells have the ability to reduce MTS [3-(4,5-dimethylthiazol-2-yl)-5-(3-carboxymethoxyphenyl)-2-(4-sulfophenyl)-2H-tetrazolium, inner salt], a yellow tetrazolium salt, to a colored formazan that is soluble in the culture medium. Briefly, 15 × 10^3^ cells per well were cultured in 100 µL culture medium. After 24 h, adherent cells were treated with different concentrations of the studied compounds, as well as adriamycin (Adr) and cisplatin (Cis-Pt), used as positive controls for 24 h and 48 h, respectively. Each well was then filled with 20 µL of reagent containing MTS and PES (phenazine ethosulfate, a cationic dye with excellent chemical stability that binds to MTS and produces a stable solution). The resulting formazan was determined using a Tecan GENios Spectrophotometer (Tecan US, Durham, NC, USA) by measuring the absorbance at λ = 492 nm after incubation for 4 h at 37 °C with moderate agitation every 15 min. The cytotoxicity tests were performed in triplicate, with results reported as mean values with standard deviations (SD). The cytotoxicity of DMSO was also investigated under the same conditions, and no harmful effect was found at doses less than 1%.

Data were expressed as cell viability by comparing with untreated cells considered 100% viable, using the following formula:

(1) $$\mathrm{Cell}\;\mathrm{viability}\;(\%)=\frac{\mathrm{absorbance}\;\mathrm{of}\;\mathrm{treated}\;\mathrm{cells}-\mathrm{absorbance}\;\mathrm{of}\;\mathrm{culture}\;\mathrm{medium}}{\mathrm{absorbance}\;\mathrm{of}\;\mathrm{untreated}\;\mathrm{cells}-\mathrm{absorbance}\;\mathrm{of}\;\mathrm{culture}\;\mathrm{medium}}$$

IC_50_ values were calculated in GraphPad Prism version 8.0.1 for Windows, GraphPad Software, San Diego, CA, USA, www.graphpad.com (accessed on 18 May 2022), using the built-in equation nonlinear regression (curve fit)–log(inhibitor) vs. normalized response variable slope y = 100/(1 + 10^((LogIC50-x) × HillSlope))^). For the calculations, concentrations were expressed in µM and transformed to the corresponding logarithmic values and the data were normalized against the smallest value of cell viability in this data set, corresponding to cisplatin.

### 4.5. Studies on DNA Binding

#### 4.5.1. Stability Studies

The stability of all tested compounds was tested in the same conditions as the samples containing CT-DNA. Solutions of the tested compounds (10^−3^ M) were prepared in DMSO; the solutions were further diluted using Tris-HCl buffer in order to obtain test solutions of 20 μM. In order to observe whether variations in absorbance or position of peaks occur, UV–Vis spectra were recorded at different time intervals: *t* = 0, 10, 20, 30, 40, 50, 60, 90 and 120 min. A blank sample containing only DMSO and buffer was used as reference.

#### 4.5.2. UV–Vis Spectra

The UV–Vis spectra of 1.8 mL samples containing 20 µM of the examined compounds and escalating volumes of DNA stock solution (0–40 µM with a 5 µM increment) were recorded for the DNA binding investigations. The samples had been prepared in Tris-HCl buffer (5 mM Tris-HCl/ 50 mM NaCl, pH 7.4 adjusted with a 0.1 M HCl solution).

The concentration of the DNA stock solution was calculated by measuring the absorption intensity at 260 nm and using a molecular extinction coefficient of 6600 M^−1^·cm^−1^. We established that the DNA stock solution was sufficiently free of protein (the ratio between the absorption at 260 nm and 280 nm, respectively, was found to be in the range 1.8–2) [84]. Using a Jasco 650 spectrophotometer (Jasco, Tokyo, Japan), these experiments were carried out in 1 cm quartz cells at room temperature.

Equation (2) was used to calculate the binding constant (*K_b_*).

(2) $$\frac{\left[\mathrm{DNA}\right]}{\varepsilon_{a}-\varepsilon_{f}}=\frac{\left[\mathrm{DNA}\right]}{\varepsilon_{b}-\varepsilon_{f}}+\frac{1}{K_{b}\left(\varepsilon_{b}-\varepsilon_{f}\right)}$$

where [DNA] is the nucleobase concentration of DNA in the sample, ε_a_, ε_b_, ε_f_ are the apparent absorption coefficient, the extinction coefficient for the completely bound complex, and the extinction coefficient for the free complex, respectively, and *K_b_* is the intrinsic binding constant. The slope (1/(ε_b_ − ε_f_)) to the intercept (1/*K_b_*(ε_b_ − ε_f_)) ratio was used to calculate *K_b_* [103].

#### 4.5.3. Competitive Binding Assay with Ethidium Bromide (EB) through Fluorescence Spectroscopy

The fluorescence spectra were recorded using 1.6 mL samples containing 10 µM CT-DNA, 2 µM EB, and increasing amounts (0–40 µM with an increment of 5 µM) of tested compounds; the samples were prepared by keeping the components in contact for 10 min at room temperature, under continuous stirring and in the dark. The excitation wavelength was 500 nm, and the spectra were recorded between the wavelengths of 520 and 800 nm. A Jasco FP 6500 fluorometer and quartz cells with a 1 cm route length were employed. The classical Stern–Volmer equation (Equation (3)) was used to interpret the obtained data [104]:

(3) $$\frac{F_{0}}{F}=1+K_{SV}\cdot \left[Q\right]$$

F_0_ and F are the fluorescence intensities of the EB–DNA samples in the absence and presence of the tested compounds, respectively; *K_SV_* is the Stern–Volmer constant, which was computed as the slope from the plot of F_0_/F vs. [Q], where [Q] is the chemical concentration.

The binding ratio was plotted against the logarithm of the concentration in comparison with a control sample exposed solely to DMSO.

(4) $$y=A_{1}+\frac{(A_{2}-A_{1})\times x^{n}}{K_{50}{}^{n}+x^{n}}$$

For fitting data points, the modified Hill function with offset (Origin^®^) was used: where y represents the binding ratio (F/F_0_), x represents the concentration, A_1_ represents the minimum of y values, A_2_ represents the maximum of y values, *K_50_* represents the concentration corresponding to 50% binding or the half maximum binding constant, and n represents the Hill coefficient [97].

### 4.6. HSA and Apo-Tf Binding Studies

#### 4.6.1. Studies on fluorescence Quenching Mechanism

Using a Jasco FP 6500 spectrofluorometer (Jasco, Tokyo, Japan) and quartz cells with a 1 cm path length, the fluorescence spectra of HSA and apo-Tf were recorded in the presence and absence of the examined substances. The 2.5 mL samples containing 2.5 µM HSA or 1 µM apo-Tf were titrated by adding 5 µL of the tested substance at a time, a volume that corresponded to a 1 µM increase in concentration. The concentration range used for these experiments varied from 0 to 8 µM. The relevant spectra were collected in the emission domains of 300–500 nm (HSA) and 305–500 nm (apo-Tf) after 3 min of continuous stirring at room temperature, using excitation wavelengths of 280 nm (HSA) and 295 nm (apo-Tf).

Experiments were conducted in a pH = 7.4 Tris-HCl buffer (5 mM Tris-HCl/50 mM NaCl for HSA and 5 mM Tris-HCl/50 mM NaCl/25 mM NaHCO_3_ for apo-Tf).

The following modified Stern–Volmer equation has been used to calculate the Stern–Volmer constant, which will be denoted as K_SV_* in order to differentiate it from the Stern–Volmer constant calculated for the DNA binding experiments:

(5) $$\frac{F_{0}}{F_{0}-F}=\frac{1}{f_{a}K_{sv}^{*}\left[Q\right]}+\frac{1}{f_{a}}$$

where F_0_ and F are the fluorescence intensities in the absence and presence of the tested compounds, fa is the fraction of fluorophore accessible to the quencher (the tested compounds), [Q] is the quencher concentration, and K_SV_* (M^−1^) is the Stern–Volmer constant; the plot of F_0_/(F_0_ − F) against 1/[Q] yields *K_SV_* as the slope and f_a_ as the y intercept. 

Subsequently, the bimolecular quenching rate constant was obtained by using Equation (6):

(6) $${K_{\mathrm{sv}}}^{*}=K_{q}\tau_{0}$$

*K_q_* (M^−1^·s^−1^) is the bimolecular quenching rate constant and τ_0_ is the fluorophore’s lifespan in the absence of the investigated chemicals (10^−8^ s) [105,106]. The value of *K_q_* can be used to assess whether the quenching phenomena have an underlying static or dynamic mechanism [83].

Using the following equation, the affinity constant (*K_a_*) and the number of binding sites (n) have been calculated:

(7) $$\mathrm{lg}\frac{F_{0}-F}{F}=\mathrm{lg}K_{a}+n\mathrm{lg}\left[Q\right]$$

The binding affinity, *K_d_*, can be calculated using the quadratic function below:

(8) $$\frac{F_{0}-F}{F_{0}-F_{b}}=\frac{{\left[P\right]}_{t}+\left[Q\right]+K_{d}-\sqrt{{\left({\left[P\right]}_{t}+\left[Q\right]+K_{d}\right)}^{2}-4{\left[P\right]}_{t}\left[Q\right]}}{2{\left[P\right]}_{t}}$$

where F and F_0_ are the fluorescence intensities of the protein in the presence and absence of the quencher, respectively, F_b_ is the fluorescence of a fully bound protein–quencher, [P]_t_ is the total concentration of the protein, and [Q] is the concentration of the added quencher. Each binding isotherm was plotted against the total quencher concentration in solution as a function of bound to free protein (F/F_0_).

The isotherms were then fitted using the nonlinear regression function below:

(9) $$\frac{F}{F_{0}}={\;F}_{1}+\left(F_{2}-F_{1}\right)\frac{K_{d}^{n}}{K_{d}^{n}+{\left[Q\right]}^{n}}$$

where n represents the Hill coefficient, F_1_ and F_2_ are the horizontal and vertical asymptotes, respectively. The *K_d_* in Equation (9) is derived from the quadratic binding function (Equation (8)) [97]. 

Data were collected in triplicate, averaged, and expressed as mean value ± standard deviation (SD). All graph fitting models were defined and built using OriginPro^®^ 2018.

#### 4.6.2. Studies on Conformational Changes of HSA and Apo-Tf Due to the Interaction with the Tested Compounds

Conformational changes were investigated by comparing the synchronous fluorescence spectra of the two proteins recorded in the presence of the studied compounds with those recorded in their absence; the spectra were recorded at Δλ = 60 nm and Δλ = 15 nm, respectively, in the wavelength interval of 250–400 nm.

## 5. Conclusions

Using nalidixic acid as the ligand, we synthesized and characterized nine homoleptic complexes with lanthanide (La(III), Sm(III), Eu(III), Gd(III), and Tb(III)) ions, with general formulas abbreviated as M(nal)_2_ and M(nal)_3_. Structural characterization has been accomplished by means of elemental analysis, spectroscopic studies (UV–VIS–NIR, FT-IR, high-resolution mass spectrometry) and thermal analysis. Geometry optimizations were carried out for the proposed structures using MOPAC/PM7 calculations and further TDDFT studies were used to predict absorption spectra and analyze frontier molecular orbitals. Overall, the results of the computational studies were consistent with the experimental findings.

The biological studies assessed the binding affinity of the complexes for DNA and serum proteins (apo-Tf and HSA), as well as their cytotoxic activities. DNA binding assay results indicate the presence of moderate contacts and possibly intercalative processes, with a groove binding type of interaction being more likely; a moderate ability to displace EB was also observed. Furthermore, based on the high binding constants, these complexes displayed considerable affinities for both proteins, particularly HSA, and further research revealed that they interact with the proteins via a static quenching mechanism. Notably, the 1:3 series generally displayed stronger binding affinity towards HSA or DNA than the corresponding 1:2 complexes.

The cytotoxic effect was tested on two cancer cell lines, MDA-MB-231 (human breast adenocarcinoma) and LoVo (human colorectal adenocarcinoma), as well as one normal cell line, HUVEC (human umbilical vein endothelial cells). The most active compounds on the LoVo cell line, with lower IC_50_ values than cisplatin, are La(nal)_2_ (IC_50_ = 25.08 ± 9.11 μM), Tb(nal)_3_ (IC_50_ = 25.55 ± 6.97 μM), Eu(nal)_3_ (IC_50_ = 26.88 ± 5.72 μM) and Gd(nal)_3_ (IC_50_ = 28.36 ± 7.95 μM). Moreover, most complexes displayed selective activity against LoVo, while being inactive against MDA-MB-231 and HUVEC. Notably, the 1:3 series generally displayed stronger cytotoxic activities, as well as binding affinities towards HSA or DNA than the 1:2 complexes. One notable exception is La(nal)_2_, which displayed more pronounced cytotoxic effects compared with La(nal)_3_. However, its binding affinity towards biomacromolecules was found to be lower.

Additionally, we noticed that the LUMO energy value is inversely linked to the HSA binding affinity and that compounds with low HSA binding affinities displayed low cytotoxic activities. To the latter observation, La(nal)_2_ constituted once again the exception. These findings suggest that the mechanism of action of these complexes, particularly La(nal)_2_, may target alternate pathways specific to cancer cells, in addition to DNA or serum transport proteins. Future studies will aim at identifying the intracellular targets of these complexes. We hope that our findings will inspire and aid research towards the development of new (or old) drugs with improved anticancer activity.

## Figures and Tables

**Table 1:** IC_50_ values (μM) for nalidixic acid, M(nal)_2_, M(nal)_3_ and cisplatin (Cis-Pt).

Compound	IC_50_ (μM)	
	LoVo	HUVEC
Nalidixic acid	>200	>200
La(nal)_2_	25.08 ± 9.11	113.5 ± 46.79
Sm(nal)_2_	92.39 ± 15.97	>200
Eu(nal)_2_	>200	>200
Gd(nal)_2_	>200	>200
Tb(nal)_2_	>200	>200
La(nal)_3_	49.83 ± 7.92	148.8 ± 17.93
Eu(nal)_3_	26.88 ± 5.72	>200
Gd(nal)_3_	28.36 ± 7.95	58.44 ± 29.32
Tb(nal)_3_	25.55 ± 6.97	96.17 ± 48.80
Cis-Pt	38.55 ± 4.63	78.48 ± 16.03

Cytotoxicity was assessed by MTS assay after 48 h of incubation with each compound for the LoVo cell line and 24 h for HUVEC. Data obtained are based on the average of three independent experiments and the reported errors are the corresponding standard deviations. IC_50_ values were calculated in GraphPad Prism 8.0.1 (GraphPad Software, San Diego, CA, USA) using the built-in equation nonlinear regression (curve fit)−log(inhibitor) vs. normalized response variable slope.

**Table 2:** Calculated values for the binding constants (*K_b_*), the Stern–Volmer quenching constants (*K_SV_*) and half maximum binding constants (*K_50_*) that characterize the interactions of the tested compounds with DNA.

Compound	*K_b_* (L∙mol^−1^) (×10^5^)	*K_SV_* (M^−1^) (×10^3^)	EB Displacement Assay	
			*K_50_* (µM)	n
**Nalidixic acid**	4.30 ± 2.22	9.87 ± 0.11	50.41 ± 9.60	2.18 ± 0.30
**La(nal)_2_**	0.61 ± 0.09	8.90 ± 0.17	31.39 ± 13.66	2.20 ± 0.81
**Sm(nal)_2_**	2.18 ± 0.76	10.24 ± 0.22	31.35 ± 4.21	2.96 ± 0.77
**Eu(nal)_2_**	0.49 ± 0.06	8.35 ± 0.13	34.75 ± 3.69	2.81 ± 0.38
**Gd(nal)_2_**	0.80 ± 0.09	10.47 ± 0.13	34.11 ± 8.90	2.33 ± 0.72
**Tb(nal)_2_**	0.37 ± 0.04	9.14 ± 0.16	34.69 ± 11.18	2.50 ± 0.96
**La(nal)_3_**	0.75 ± 0.17	2.48 ± 0.44	36.45 ± 16.53	2.17 ± 0.99
**Eu(nal)_3_**	3.84 ± 1.02	9.62 ± 0.10	30.96 ± 10.85	1.98 ± 0.95
**Gd(nal)_3_**	4.17 ± 1.72	9.86 ± 0.19	31.88 ± 7.73	3.33 ± 1.43
**Tb(nal)_3_**	2.37 ± 0.61	8.35 ± 0.11	31.96 ± 10.16	2.60 ± 1.21

**Table 3:** Binding constants and thermodynamic parameters calculated for the interaction of the complexes with HSA and apo-Tf at 298 K.

Complex	K_sv_* (M^−1^)	*K_q_* (M^−1^∙s^−1^)	*K_a_* (M^−1^)	n_1_ (Number of Binding Sites)	*K_d_* (μM)	n_2_ (Hill Coefficient)
Apo-Tf						
**Nal**	(1.02 ± 0.09) × 10^5^ (f_a_ = 0.48 ± 0.03)	1.02 × 10^13^	(0.31 ± 0.08) × 10^4^	0.79 ± 0.02	6.55 ± 2.11	1.55 ± 0.37
**La(nal)_2_**	(0.44 ± 0.23) × 10^5^ (f_a_ = 1.20 ± 0.49)	0.44 × 10^13^	(6.94 ± 2.32) × 10^4^	1.00 ± 0.03	5.43 ± 1.46	1.82 ± 0.55
**Sm(nal)_2_**	(1.16 ± 0.06) × 10^5^ (f_a_ = 0.79 ± 0.02)	1.16 × 10^13^	(10.17 ± 6.14) × 10^4^	1.01 ± 0.05	6.16 ± 1.27	1.71 ± 0.31
**Eu(nal)_2_**	(1.85 ± 0.06) × 10^5^ (f_a_ = 0.71 ± 0.01)	1.85 × 10^13^	(1.02 ± 0.13) × 10^4^	0.80 ± 0.01	7.07 ± 6.13	0.82 ± 0.41
**Gd(nal)_2_**	(0.78 ± 0.09) × 10^5^ (f_a_ = 0.92 ± 0.07)	0.78 × 10^13^	(3.46 ± 1.34) × 10^4^	0.94 ± 0.03	8.87 ± 6.20	1.20 ± 0.40
**Tb(nal)_2_**	(2.23 ± 0.15) × 10^5^ (f_a_ = 0.65 ± 0.02)	2.23 × 10^13^	(0.32 ± 0.08) × 10^4^	0.71 ± 0.02	8.01 ± 4.30	0.84 ± 0.22
**La(nal)_3_**	(1.04 ± 0.13) × 10^5^ (f_a_ = 0.80 ± 0.06)	1.04 × 10^13^	(1.51 ± 0.72) × 10^4^	0.86 ± 0.04	8.64 ± 2.22	1.04 ± 0.19
**Eu(nal)_3_**	(2.21 ± 0.13) × 10^5^ (f_a_ = 0.80 ± 0.02)	2.21 × 10^13^	(2.25 ± 1.01) × 10^4^	0.84 ± 0.03	3.85 ± 0.29	1.58 ± 0.23
**Gd(nal)_3_**	(1.78 ± 0.07) × 10^5^ (f_a_ = 0.82 ± 0.02)	1.78 × 10^13^	(2.63 ± 0.39) × 10^4^	0.87 ± 0.01	5.86 ± 0.72	1.22 ± 0.12
**Tb(nal)_3_**	(2.70 ± 0.11) × 10^5^ (f_a_ = 0.68 ± 0.01)	2.70 × 10^13^	(1.63 ± 0.42) × 10^4^	0.83 ± 0.02	5.76 ± 2.60	0.98 ± 0.34
**HSA**						
**Nal**	(1.25 ± 0.06) × 10^5^ (f_a_ = 1.06 ± 0.02)	1.25 × 10^13^	(2.19 ± 0.46) × 10^5^	1.03 ± 0.01	5.69 ± 1.44	1.33 ± 0.29
**La(nal)_2_**	(1.09 ± 0.02) × 10^5^ (f_a_ = 1.09 ± 0.02)	1.09 × 10^13^	(9.03 ± 1.04) × 10^5^	1.09 ± 0.01	3.35 ± 0.11	1.32 ± 0.11
**Sm(nal)_2_**	(2.50 ± 0.12) × 10^5^ (f_a_ = 1.05 ± 0.02)	2.50 × 10^13^	(8.25 ± 1.62) × 10^5^	1.08 ± 0.01	3.60 ± 0.11	1.34 ± 0.09
**Eu(nal)_2_**	(1.75 ± 0.11) × 10^5^ (f_a_ = 1.17 ± 0.04)	1.74 × 10^13^	(11.99 ± 1.38) × 10^5^	1.13 ± 0.01	4.63 ± 0.97	1.14 ± 0.27
**Gd(nal)_2_**	(1.51 ± 0.07) × 10^5^ (f_a_ = 1.25 ± 0.03)	1.51 × 10^13^	(15.52 ± 2.93) × 10^5^	1.15 ± 0.01	4.79 ± 0.84	1.20 ± 0.23
**Tb(nal)_2_**	(1.92 ± 0.08) × 10^5^ (f_a_ = 1.13 ± 0.02)	1.92 × 10^13^	(11.52 ± 2.87) × 10^5^	1.12 ± 0.02	3.99 ± 0.14	1.52 ± 0.09
**La(nal)_3_**	(2.75 ± 0.10) × 10^5^ (f_a_ = 1.12 ± 0.01)	2.75 × 10^13^	(27.48 ± 2.10) × 10^5^	1.16 ± 0.00	2.91 ± 0.07	1.18 ± 0.08
**Eu(nal)_3_**	(2.89 ± 0.11) × 10^5^ (f_a_ = 1.13 ± 0.01)	2.89 × 10^13^	(21.59 ± 2.04) × 10^5^	1.13 ± 0.00	2.67 ± 0.21	1.17 ± 0.27
**Gd(nal)_3_**	(2.69 ± 0.05) × 10^5^ (f_a_ = 1.10 ± 0.00)	2.69 × 10^13^	(19.61 ± 1.83) × 10^5^	1.14 ± 0.01	3.27 ± 0.11	1.35 ± 0.12
**Tb(nal)_3_**	(2.39 ± 0.10) × 10^5^ (f_a_ = 1.13 ± 0.02)	2.39 × 10^13^	(31.26 ± 9.66) × 10^5^	1.18 ± 0.02	4.61 ± 1.67	0.89 ± 0.35

## Data Availability

Data is contained within the article and supplementary material.

## Supplementary Materials

The following are available online at https://www.mdpi.com/article/10.3390/ph15081010/s1, Table S1: Wavelengths and absorbance (A) values observed in the UV–Vis–NIR spectra of nalidixic acid and complexes; Table S2: IR data (cm^−1^) and band assignments for nalidixic acid (Nal), sodium salt of nalidixic acid (Nal-Na^+^) and M(nal)_2_ compounds, M = La^3+^, Sm^3+^, Eu^3+^, Gd^3+^, Tb^3+^; Table S3: IR data (cm^−1^) and band assignments for nalidixic acid (Nal), sodium salt of nalidixic acid (Nal-Na^+^) and M(nal)_3_ compounds, M = La^3+^, Eu^3+^, Gd^3+^, Tb^3+^; Table S4: Thermal decomposition data (in air flow) for the M(nal)_2_ complexes, M = La^3+^, Sm^3+^, Eu^3+^, Gd^3+^, Tb^3+^; Table S5: Thermal decomposition data (in air flow) for the M(nal)_3_ complexes, M = La^3+^, Eu^3+^, Gd^3+^, Tb^3+^; Table S6: Geometric parameters: bond lengths, bond angles, dihedral angles, charge density and total energy of M(nal)_2_ and M(nal)_3_; Table S7: IR selected data (experimental vs. predicted) for the optimized structures of Eu(nal)_2_ and Eu(nal)_3_; Table S8: UV–Vis spectral data for nalidixic acid and complexes (experimental vs. predicted); Table S9: Absorbances of tested compounds in UV–Vis, in DMSO–TrisHCl buffer, [compound] = 20μM; Figure S1: Experimental and predicted vibrational spectra of nalidixic acid and its M(nal)_2_ and M(nal)_3_ metal complexes; Figure S2: Mass spectra for the synthesized complexes: (**A**) La(nal)_2_; (**B**) Sm(nal)_2_; (**C**) Eu(nal)_2_; (**D**) Gd(nal)_2_; (**E**) Tb(nal)_2_; (**F**) La(nal)_3_; (**G**) Eu(nal)_3_; (**H**) Gd(nal)_3_; (**I**) Tb(nal)_3_; Figure S3: TG, DTG and DTA curves for the M(nal)_2_ complexes, M = La^3+^, Sm^3+^, Eu^3+^, Gd^3+^, Tb^3+^; Figure S4: TG, DTG and DTA curves for the M(nal)_3_ complexes, M = La^3+^, Sm^3+^, Eu^3+^, Gd^3+^, Tb^3+^; Figure S5: ^1^H-NMR spectrum of nalidixic acid; Figure S6: ^1^H-NMR spectrum of La(nal)_3_; Figure S7: Experimental and predicted electronic spectra of nalidixic acid and its metal complexes; Figure S8: Cytotoxicity of M(nal)_2_ (M = La^3+^, Sm^3+^, Eu^3+^, Gd^3+^, Tb^3+^) and M(nal)_3_ (M = La^3+^, Eu^3+^, Gd^3+^, Tb^3+^) complexes tested on the following cell lines: (**A**) HUVEC-24h, (**B**) HUVEC-48h, (**C**) LoVo-24h, (**D**) LoVo-48h, (**E**) MDA-MB 231-24h and (**F**) MDA-MB 231-48h; Figure S9: Graphical representations of normalized cell viability vs. log(concentration). Concentrations were expressed in µM and transformed to the corresponding logarithmic values. Data were normalized against the smallest value of cell viability in this data set, corresponding to cisplatin, and the control corresponding to untreated cells. Results were plotted in GraphPad Prism 8.0.1 using the built-in equation nonlinear regression (curve fit) − log (inhibitor) vs. normalized response variable slope; Figure S10: Stability assay: UV–vis spectra of the complexes in DMSO–TrisHCl buffer mixture (5 mM Tris-HCl/ 50 mM NaCl, pH 7.4); Figure S11: Absorption spectra of the tested compounds in the absence and presence of increasing amounts of DNA; [compound] = 20 μM; [DNA] = 0, 5, 10, 15, 20, 25, 30, 35, 40 μM. The arrows show the absorption changes upon increasing the DNA concentration; Figure S12: Graphical representations of the DNA binding constants (*K_b_*) for systems containing [compound] = 20 μM; [DNA] = 5, 10, 15, 20, 25, 30, 35, 40 μM. *K_b_* values were calculated using Equation (2) in OriginPro^®^ 2018. Outliers are represented on the graphs as red data points; Figure S13: Fluorescence spectra of the EB–DNA system in the absence and presence of increasing amounts of the tested compounds. λ_ex_ = 500 nm, [EB] = 2 μM, [DNA] = 10 μM, [compound] = 0, 5, 10, 15, 20, 25, 30, 35, 40 μM. Arrows indicate the changes in fluorescence intensities upon increasing the concentrations of the tested compounds; Figure S14: Graphical representations of the *K_SV_* constants for systems containing [EB] = 2 μM, [DNA] = 10 μM, [compound] = 5, 10, 15, 20, 25, 30, 35, 40 μM. *K_SV_* values were calculated using Equation (3) in OriginPro^®^ 2018. Outliers are represented on the graphs as red data points; Figure S15: Graphical representation of the *K_50_* constants for systems containing [EB] = 2 μM, [DNA] = 10 μM, [compound] = 5, 10, 15, 20, 25, 30, 35, 40 μM. *K_50_* values were calculated using Equation 3 in OriginPro^®^ 2018. Outliers are represented on the graphs as red data points; Figure S16: Changes in fluorescence intensity of free HSA vs. compound–HSA systems; the black arrows indicate a decrease in the intensity upon the addition of increasing amounts of compound; [HSA] = 2.5 μM, [compound] = 0, 1, 2, 3, 4, 5, 6, 7, 8 μM; Figure S17: Changes in fluorescence intensity of free apo-Tf vs. compound–apo-Tf systems; the black arrows indicate a decrease in the intensity upon the addition of increasing amounts of compound; [apo-Tf] = 1 μM, [compound] = 0, 1, 2, 3, 4, 5, 6, 7, 8 μM; Figure S18: Variation of the HSA fluorescence peak area upon adding increasing amounts of the studied compounds. [HSA] = 2.5 μM, [compound] = 0, 1, 2, 3, 4, 5, 6, 7, 8 μM; Figure S19: Variation of the apo-Tf fluorescence peak area upon adding increasing amounts of the studied compounds; [apo-Tf] = 1 μM, [compound] = 0, 1, 2, 3, 4, 5, 6, 7, 8 μM; Figure S20: Classical (left) and modified (right) Stern–Volmer plots for each of the HSA interaction studies; Figure S21: Classical (left) and modified (right) Stern–Volmer plots for each of the apo-Tf interaction studies; Figure S22: Representation of *K_a_* and *K_d_* constants for each of the studied compound–HSA systems; Figure S23: Representation of *K_a_* and *K_d_* constants for each of the studied compound–apo-Tf systems; Figure S24: Synchronous spectra for the HSA interaction systems recorded at Δλ = 15 nm. [HSA] = 2.5 μM, [compound] = 0, 1, 2, 3, 4, 5, 6, 7, 8 μM; Figure S25: Synchronous spectra for the HSA interaction systems recorded at at Δλ = 60 nm; [HSA] = 2.5 μM, [compound] = 0, 1, 2, 3, 4, 5, 6, 7, 8 μM; Figure S26: Synchronous spectra of the tested compounds–apo-Tf systems recorded at Δλ =15 nm; [apo-Tf] = 1 μM, [compound] = 0, 1, 2, 3, 4, 5, 6, 7, 8 μM; Figure S27: Synchronous spectra of the tested compounds–apo-Tf interaction systems recorded at Δλ = 60 nm; [apo-Tf] = 1 μM, [compound] = 0, 1, 2, 3, 4, 5, 6, 7, 8 μM.
