# Supplementary material for: A Study on Repositioning Nalidixic Acid via Lanthanide Complexation: Synthesis, Characterization, Cytotoxicity and DNA/Protein Binding Studies
Source: Pharmaceuticals (Basel). 2022 Aug 17;15(8):1010. doi: 10.3390/ph15081010 (PMC9412414; doi:10.3390/ph15081010)
Supplement: Supplementary file 1 [file pharmaceuticals-15-01010-s001.zip › pharmaceuticals-1799753-supplementary.pdf]

A study on repositioning nalidixic acid *via* lanthanide complexation: Synthesis, characterization, cytotoxicity and DNA/protein binding studies

Ana-Madalina Maciuca[1], Alexandra-Cristina Munteanu[1*], Mirela Mihaila[2], Mihaela Badea[3], Rodica Olar[3], George Mihai Nitulescu[4], Cristian V. A. Munteanu[5], and Valentina Uivarosi[1*]

[1] Department of General and Inorganic Chemistry, Faculty of Pharmacy, Carol Davila University of Medicine and Pharmacy, 6 Traian Vuia St, Bucharest 020956, Romania;

[2] Center of Immunology, Stefan S. Nicolau Institute of Virology, 285 Mihai Bravu Ave, Bucharest 030304, Romania;

[3] Department of Inorganic Chemistry, Faculty of Chemistry, University of Bucharest, 90-92 Panduri Str, Bucharest 050663, Romania;

[4] Department of Pharmaceutical Chemistry, Faculty of Pharmacy, Carol Davila University of Medicine and Pharmacy, 6 Traian Vuia Str, Bucharest 020956, Romania;

[5] Romanian Academy-Institute of Biochemistry (IBAR), 296 Spl. Independenţei, 060031 Bucharest, Romania;

* Correspondence: alexandra.ticea@umfcd.ro; valentina.uivarosi@umfcd.ro
Tel.: +4-021-318-0742; Fax: +4-021-318-0750.

Abstract: "Drug repositioning" is a modern strategy used to uncover new applications for out-of-date drugs. In this context, nalidixic acid, the first member of the quinolone class with limited use today, has been selected to obtain nine new metal complexes with lanthanide cations (La^{3+}, Sm^{3+}, Eu^{3+}, Gd^{3+}, Tb^{3+}); the experimental data suggest that the quinolone acts as a bidentate ligand, binding to the metal ion *via* the keto and carboxylate oxygen atoms, findings that are supported by DFT calculations. The cytotoxic activity of the complexes has been studied using tumoral cell lines, MDA-MB-231 and LoVo, and a normal cell line, HUVEC. Their affinity for DNA and the manner of binding have been tested using UV-Vis spectroscopy and competitive binding studies; our results indicate that major and minor groove-binding play a significant role in these interactions. The affinity towards serum proteins has also been evaluated, the complexes displaying higher affinity towards albumin than apo-transferrin.

Keywords: drug repositioning; nalidixic acid; lanthanide ions; anticancer; DNA binding; serum proteins binding.

**Table S1.** Wavelengths and absorbance (A) values observed in the UV-Vis-NIR spectra of nalidixic acid and complexes.	4
**Table S2.** IR data (cm^{-1}) and band assignments for nalidixic acid (Nal), sodium salt of nalidixic acid ($Nal\text{-}Na^{+}$) and $M(nal)_2$ compounds, M= La^{3+}, Sm^{3+}, Eu^{3+}, Gd^{3+}, Tb^{3+}.	5
**Table S3.** IR data (cm^{-1}) and band assignments for nalidixic acid (Nal), sodium salt of nalidixic acid ($Nal\text{-}Na^{+}$) and $M(nal)_3$ compounds, M= La^{3+}, Eu^{3+}, Gd^{3+}, Tb^{3+}.	6
**Figure S1.** Experimental and predicted vibrational spectra of nalidixic acid and its $M(nal)_2$ and $M(nal)_3$ metal complexes.	7
**Figure S2.** Mass spectra for the synthesized complexes: **A.** $La(nal)_2$; **B**. $Sm(nal)_2$; **C.** $Eu(nal)_2$; **D.** $Gd(nal)_2$; **E.** $Tb(nal)_2$; **F.** $La(nal)_3$; **G.** $Eu(nal)_3$; **H.** $Gd(nal)_3$; **I.** $Tb(nal)_3$.	14
**Figure S3.** TG, DTG and DTA curves for the $M(nal)_2$ complexes, M= La^{3+}, Sm^{3+}, Eu^{3+}, Gd^{3+}, Tb^{3+}.	23
**Table S4.** Thermal decomposition data (in air flow) for the $M(nal)_2$ complexes, M= La^{3+}, Sm^{3+}, Eu^{3+}, Gd^{3+}, Tb^{3+}.	24
**Figure S4.** TG, DTG and DTA curves for the $M(nal)_3$ complexes, M= La^{3+}, Sm^{3+}, Eu^{3+}, Gd^{3+}, Tb^{3+}.	25
**Table S5.** Thermal decomposition data (in air flow) for the $M(nal)_3$ complexes, M= La^{3+}, Eu^{3+}, Gd^{3+}, Tb^{3+}.	25
**Figure S5.** ^{1}H-NMR spectrum of nalidixic acid.	26
**Figure S6.** ^{1}H-NMR spectrum of $La(nal)_3$.	26
**Table S6.** Geometric parameters: bond lengths, bond angles, dihedral angles, charge density, total energy of $M(nal)_2$ and $M(nal)_3$.	27
**Table S7.** IR selected data (experimental vs. predicted) for the optimized structures of $Eu(nal)_2$ and $Eu(nal)_3$.	32
**Figure S7.** Experimental and predicted electronic spectra of nalidixic acid and its metal complexes.	32
**Table S8.** UV-Vis spectral data for nalidixic acid and complexes (experimental vs. predicted)**.**	35
**Figure S8.** Citotoxicity of $M(nal)_2$ (M= La^{3+}, Sm^{3+}, Eu^{3+}, Gd^{3+}, Tb^{3+}) and $M(nal)_3$ (M= La^{3+}, Eu^{3+}, Gd^{3+}, Tb^{3+}) complexes tested on the following cell lines: **A.** HUVEC - 24h, **B.** HUVEC- 48h, **C**. LoVo – 24h, **D**. LoVo-48h, **E.** MDA-MB 231- 24h and **F**. MDA-MB 231- 48h.	36
**Figure S9.** Graphical representations of normalized cell viability *vs.* log(Concentration). Concentrations were expressed in μM and transformed to the corresponding logarithmic values. Data were normalized against the smallest value of cell viability in this data set, corresponding to cisplatin, and the control corresponding to untreated cells. Results were plotted in GraphPad Prism 8.0.1 using the built-in equation Nonlinear regression (curve fit) – log(inhibitor) *vs.* normalized response variable slope.	37

**Figure S10.** Stability assay: UV-vis spectra of the complexes in DMSO-TrisHCl buffer mixture (5 mM Tris-HCl/ 50 mM NaCl, pH 7.4).	40
**Table S9.** Absorbances of tested compounds in UV-Vis, in DMSO-TrisHCl buffer, [compound]= 20μM.	42
**Figure S11.** Absorption spectra of the tested compounds in the absence and presence of increasing amounts of DNA. [compound] = 20 μM; [DNA] = 0, 5, 10, 15, 20, 25, 30, 35, 40 μM. The arrows show the absorption changes upon increasing the DNA concentration.	43
**Figure S12.** Graphical representations of the DNA-binding constants (K_b) for systems containing [compound] = 20 μM; [DNA] = 5, 10, 15, 20, 25, 30, 35, 40 μM. K_b values were calculated using Equation 2 in OriginPro® 2018. Outliers are represented on the graphs as red data points.	45
**Figure S13.** Fluorescence spectra of the EB - DNA system in the absence and presence of increasing amounts of the tested compounds. λ_{ex} = 500 nm, [EB] = 2 μM, [DNA] = 10 μM, [compound] = 0, 5, 10, 15, 20, 25, 30, 35, 40 μM. Arrows indicate the changes in fluorescence intensities upon increasing the concentrations of the tested compounds.	47
**Figure S14.** Graphical representations of the K_{SV} constants for systems containing [EB] = 2 μM, [DNA] = 10 μM, [compound] = 5, 10, 15, 20, 25, 30, 35, 40 μM. K_{SV} values were calculated using Equation 3 in OriginPro® 2018. Outliers are represented on the graphs as red data points.	49
**Figure S15.** Graphical representation of the K_{50} constants for systems containing [EB] = 2 μM, [DNA] = 10 μM, [compound] = 5, 10, 15, 20, 25, 30, 35, 40 μM. K_{50} values were calculated using Equation 3 in OriginPro® 2018. Outliers are represented on the graphs as red data points.	51
**Figure S16.** Changes in fluorescence intensity of free HSA *vs.* compound-HSA systems; the black arrows indicate a decrease of the intensity upon the addition of increasing amounts of compound; [HSA] = 2.5 μM, [compound] = 0, 1, 2, 3, 4, 5, 6, 7, 8 μM.	53
**Figure S17.** Changes in fluorescence intensity of free apo-Tf *vs.* compound-apo-Tf systems; the black arrows indicate a decrease of the intensity upon the addition of increasing amounts of compound. [apo-Tf] = 1 μM, [compound] = 0, 1, 2, 3, 4, 5, 6, 7, 8 μM.	55
**Figure S18.** Variation of the HSA fluorescence peak area upon adding increasing amounts of the studied compounds. [HSA] = 2.5 μM, [compound] = 0, 1, 2, 3, 4, 5, 6, 7, 8 μM.	57
**Figure S19.** Variation of the apo-Tf fluorescence peak area upon adding increasing amounts of the studied compounds. [apo-Tf] = 1 μM, [compound] = 0, 1, 2, 3, 4, 5, 6, 7, 8 μM.	59
**Figure S20.** Classical (left) and modified (right) Stern-Volmer plots for each of the HSA interaction studies.	61
**Figure S21.** Classical (left) and modified (right) Stern-Volmer plots for the apo-Tf interaction assay	65
**Figure S22.** Representation of Ka and Kd constants for each of the studied compound-HSA systems.	69
**Figure S23.** Representation of Ka and Kd constants for each of the studied compound-apo-Tf systems.	73
**Figure S24.** Synchronous spectra for the HSA interaction systems recorded at $\Delta\lambda$= 15 nm. [HSA] = 2.5 μM, [compound] = 0, 1, 2, 3, 4, 5, 6, 7, 8 μM.	77
**Figure S25.** Synchronous spectra for the HSA interaction systems recorded at at $\Delta\lambda$= 60 nm. [HSA] = 2.5 μM, [compound] = 0, 1, 2, 3, 4, 5, 6, 7, 8 μM.	79
**Figure S26.** Synchronous spectra of the tested compounds - apo-Tf systems recorded at $\Delta\lambda$=15 nm. [apo-Tf] = 1 μM, [compound] = 0, 1, 2, 3, 4, 5, 6, 7, 8 μM.	80
**Figure S27.** Synchronous spectra of the tested compounds - apo-Tf interaction systems recorded at $\Delta\lambda$=60 nm. [apo-Tf] = 1 μM, [compound] = 0, 1, 2, 3, 4, 5, 6, 7, 8 μM.	83

Table S1. Wavelengths and absorbance (A) values observed in the UV-Vis-NIR spectra of nalidixic acid and complexes.

**Compus**	**Nalidixic acid**									
**λ(nm)**	255	-	315	-	-	-	1385	1670	-	-
**A**	0.757	-	0.829	-	-	-	0.089	0.1846	-	-
	**La(nal)$_2$**									
**λ(nm)**	260	-	335	-	-	-	-	1670	1935	-
**A**	0.924	-	0.982	-	-	-	-	0.114	0.198	-
	**Sm(nal)$_2$**									
**λ(nm)**	250	-	320	685	1090	1245	1395	1500	1940	-
**A**	1,009	-	1.054	0.164	0.153	0.181	0.187	0.187	0.260	-
	**Eu(nal)$_2$**									
**λ(nm)**	265	-	340	-	-	-	1455	1675	1935	-
**A**	0.425	-	0.558	-	-	-	0.132	0.150	0.272	-
	**Gd(nal)$_2$**									
**λ(nm)**	255	-	330	685	-	-	1455	1675	1935	-
**A**	0.907	-	1.010	0.134	-	-	0.128	0.145	0.247	-
	**Tb(nal)$_2$**									
**λ(nm)**	265	-	345	685	-	-	1455	1680	1940	-
**A**	0.626	-	0.769	0.275	-	-	0.238	0.253	0.385	-
	**La(nal)$_3$**									
**λ(nm)**	255	-	330	-	-	-	1455	1670	1945	-
**A**	0.948	-	1.023	-	-	-	0.121	0.135	0.269	-
	**Eu(nal)$_3$**									
**λ(nm)**	260	-	335	-	-	-	1445	1670	1945	-
**A**	0.304	-	0.460	-	-	-	0.116	0.153	0.275	-
	**Gd(nal)$_3$**									
**λ(nm)**	255	-	325	685	-	-	-	1670	1925	1950
**A**	0.993	-	1.049	0.279	-	-	-	0.275	0.367	0.363
	**Tb(nal)$_3$**									
**λ(nm)**	245	280	355	685	-	-	1440	1670	1925	1940
**A**	0.751	0.829	0.922	0.118	-	-	0.106	0.147	0.264	0.261

Table S2. IR data (cm^{-1}) and band assignments for nalidixic acid (Nal) , sodium salt of nalidixic acid (Nal-Na^+) and M(nal)$_2$ compounds, M= La^{3+}, Sm^{3+}, Eu^{3+}, Gd^{3+}, Tb^{3+}.

Nal	Nal-Na^+	La(nal)$_2$	Sm(nal)$_2$	Eu(nal)$_2$	Gd(nal)$_2$	Tb(nal)$_2$	Asignments
-	3376 wb	3725 vw<br>3399 w	3649 w<br>3420 wb	3395 wn	3377 wb	3397 wb	ν(O–H); COOH, H_2O
3044 w	-	3056 w	-	3068 w	-	-	Aromatic ν(C–H)
2984 w<br>2946 w	2923 w	2974 w<br>2925 w	-	2981 w<br>2931 w	2981 w<br>2931 w	2981 w<br>2932 w	ν (CH_3-CH_2)
1707 s	-	-	-	-	-	-	ν(C=O); COOH
-	1632 vs	1618	1616	1615	1614	1615	ν as (COO^-)
1613 s	1606	1570	1568 s	1567 s	1567 s	1568 s	Pyridonic ν(C=O)
1561	1535 m	1524	1523 s	1523 s	1523s	1523 s	Pyridonic ν (C-N)
1517	1496 m	1499 m	1500m	1499 m	1499 m	1500 m	ν (C=C)
1469 s<br>1442 w	1391 s	1348 m	1347 m	1346 s	1346 s	1347 s	ν (–CH); δ (–CH_3)
-	1438 vs	1443 s	1443 s	1442 s	1441 s	1442 s	ν s(COO^-)
-	194	175	173	173	173	173	**Δ=** ν as (COO^-)- ν s (COO^-)
1151 w	1130 w	1173 w	1131 m	1130 m	1130 m	1131 m	ν (C–N)
1033 w<br>1006	1035 w	1034 w<br>1009	1092 w<br>1050 w	1092 w<br>1049 w	1092 w<br>1050 w	1092 w<br>1050 w	δ (–CH_2)
803 s<br>777 w<br>743w<br>705 m	771 w<br>749 s<br>702 w	758 m	781 w<br>757 m<br>704 w	781 w<br>756 m<br>703 w	780 w<br>756 s<br>704 w	782 w<br>757 m<br>704 w	δ (COO^-)
656 m<br>634 m<br>539 m<br>504 w<br>485 m<br>455 m<br>424 m	649 m<br>630 m<br>560 m<br>535 m<br>484 m<br>446 m<br>432 m<br>407 m	656 m<br>635 w<br>540 m<br>502 m<br>453	657 mw<br>635 w<br>541 m<br>458 m<br>422 w	657 m<br>635 w<br>541 m<br>469 m	656 m<br>634 w<br>539 m<br>490 m<br>457 w	658 m<br>635 w<br>542 m<br>492 s<br>446 w	Ring deformation
		578 m<br>492 m<br>477 w<br>410 s	599 m<br>559 m<br>492 s<br>409 s	559 m<br>491 s	558 m<br>408 s	602 w<br>559 m<br>469 s<br>407 s	ν (M–O)

Table S3. IR data (cm^{-1}) and band assignments for nalidixic acid (Nal), sodium salt of nalidixic acid (Nal-Na^+) and M(nal)$_3$ compounds, M= La^{3+}, Eu^{3+}, Gd^{3+}, Tb^{3+}.

Nal	Nal-Na^+	La(nal)$_3$	Eu(nal)$_3$	Gd(nal)$_3$	Tb(nal)$_3$	Asignments
-	3376 wb	3403 w	3399 wb	3436 wb	3420 wb	ν(O–H); COOH, H_2O
3044 w	-	3060 w	3061 w	-	3059 w	Aromatic ν(C–H)
2984 w<br>2946 w	2923 w	2972 w	2971 w<br>2929 w	2968 w	2970 w<br>2932 w	ν (CH_3-CH_2)
1707 s	-	-	-	-	-	ν(C=O); COOH
-	1632 vs	1614 s	1615 s	1615 s	1614 s	ν as(COO^-)
1613 s	1606	1557 s	1558 s	1559 s	1559 s	Pyridonic ν(C=O)
1517	1496 m	1499 m	1499 m	1499 m	1499 m	ν (C=C)
1469 s	1391 s	1344 m	1345 s	1345 s	1345 s	ν (–CH); δ (–CH_3)
1384 m	1362 m	-	1380 w	1378 w	1378 w	Methyl δ(C-H)
-	1438 vs	1439 s	1441 s	1439 s	1439 s	ν s(COO^-)
-	194	175	174	176	175	**Δ=** ν as (COO^-)- ν s(COO^-)
1293 m	1298 m	1284 w	1286 m	1284 m	1284 m	δr(–CH_2)
1151 w	1130 w	1157 w<br>1129 m	1159 m	1129 m	1128 m	ν (C–N)
1033 w<br>1006 w	1035 w	1049 w	1092 w<br>1049 w	1102 w<br>1047 w	1092 w<br>1049 w	δ (–CH_2)
803 s<br>777 w<br>743 w<br>705 m	771 w<br>749 s<br>702	786 w<br>754 s<br>704 w	782 w<br>757 m<br>703 w	782 w<br>758 s<br>705 w	783 w<br>758 m<br>704 w	δ (COO^-)
656 m<br>634 m<br>539 m<br>504 m<br>485 s<br>455 m<br>424 w	649 m<br>630 m<br>560 m<br>535 m<br>484 m<br>446 m<br>432 m<br>407 m	656 m<br>634 w<br>541 m<br>489 s<br>457 w	657 m<br>635 w<br>541 m<br>488 s<br>419m	657 m<br>634 w<br>542 m<br>489 m<br>451 w	657 m<br>634 w<br>542 m<br>488 s<br>477 s<br>431 w	Ring deformation
		558 m<br>425 m	463 w<br>433 w<br>402 s	599 m<br>558<br>405 s	590 w<br>558 m<br>403 s	ν (M–O)

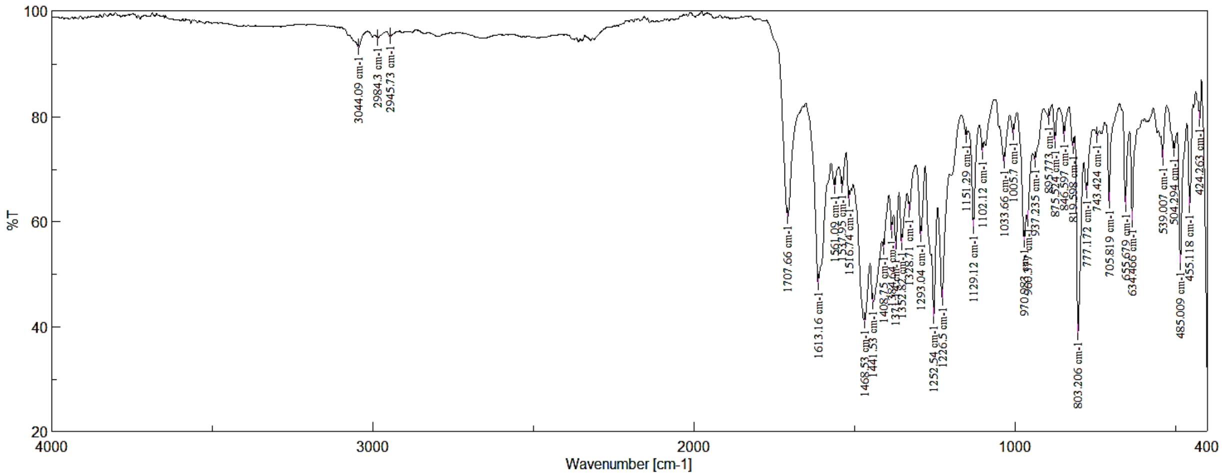

FT-IR spectrum of nalidixic acid

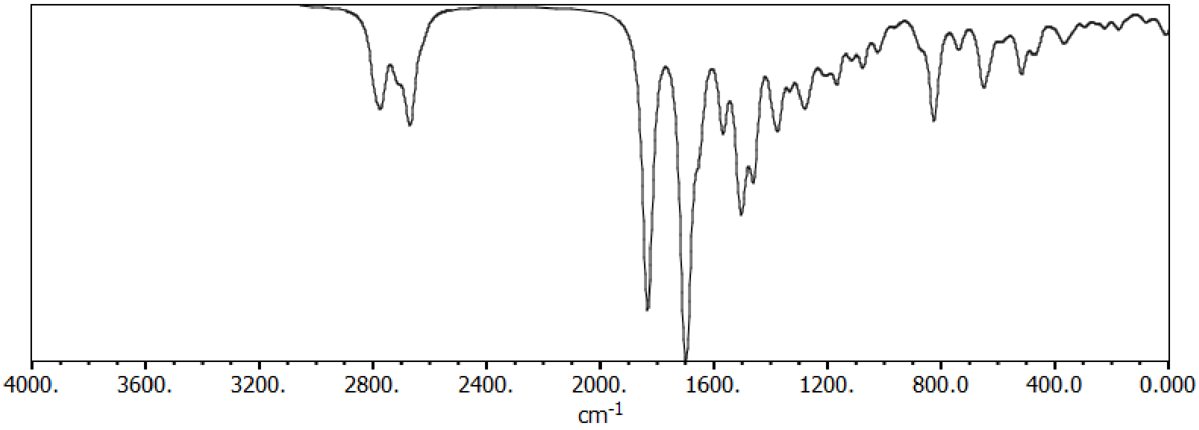

Predicted spectrum of nalidixic acid

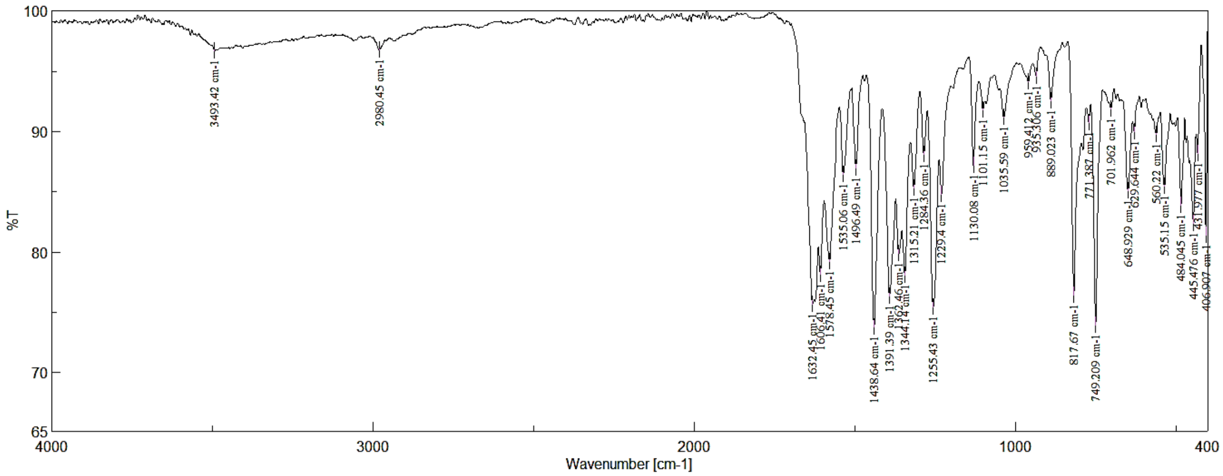

FT-IR spectrum of nalidixic acid sodium salt ($Nal^{-}Na^{+}$)

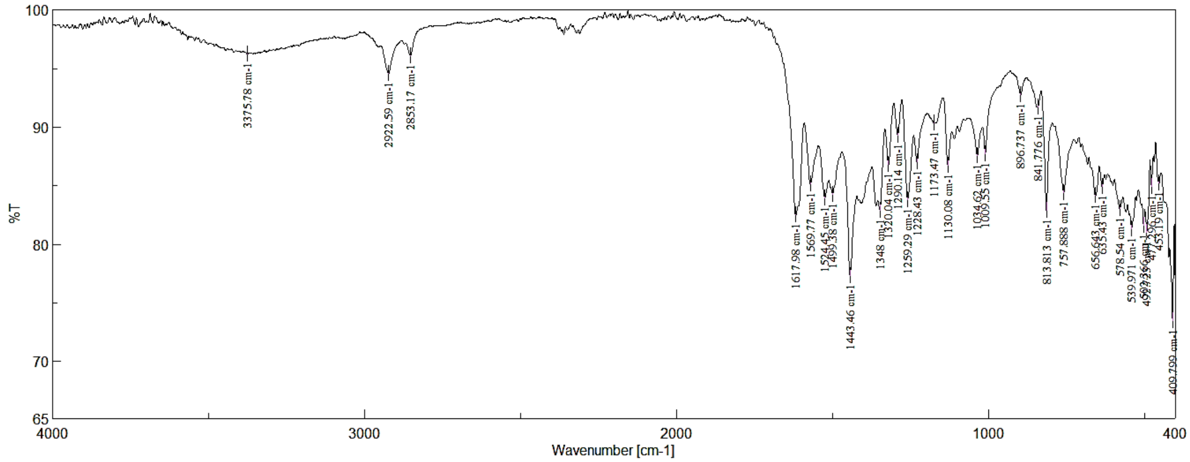

FT-IR spectrum of La(nal)$_2$

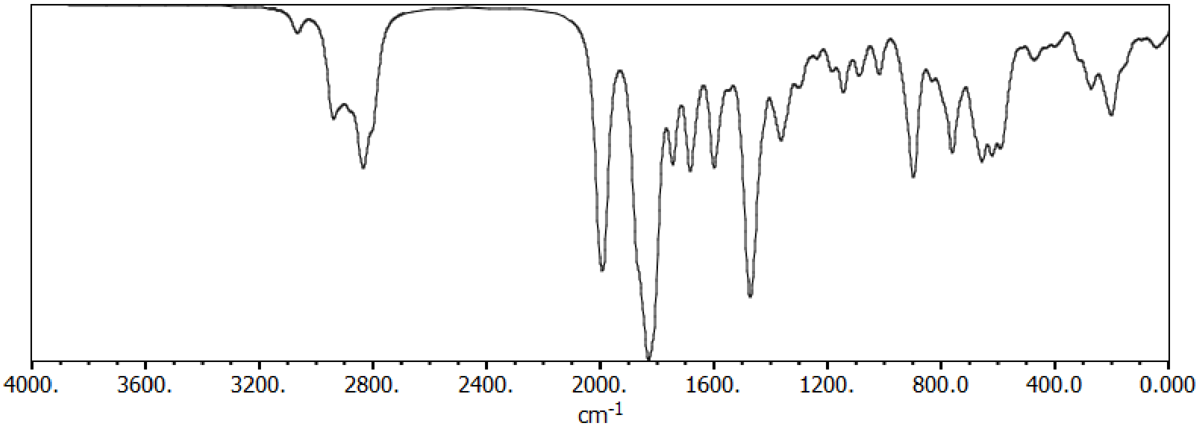

Predicted spectrum of La(nal)$_2$

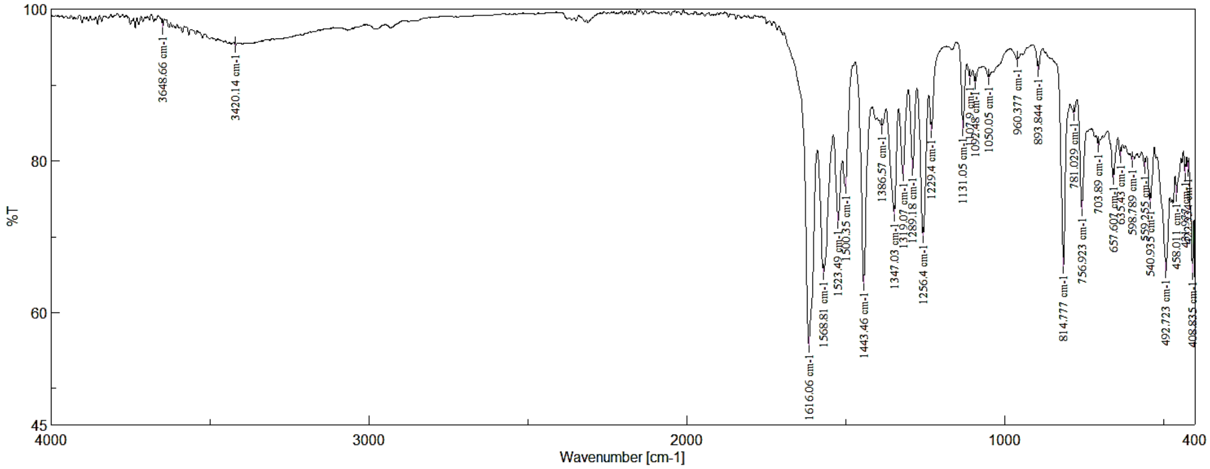

FT-IR spectrum of Sm(nal)$_2$

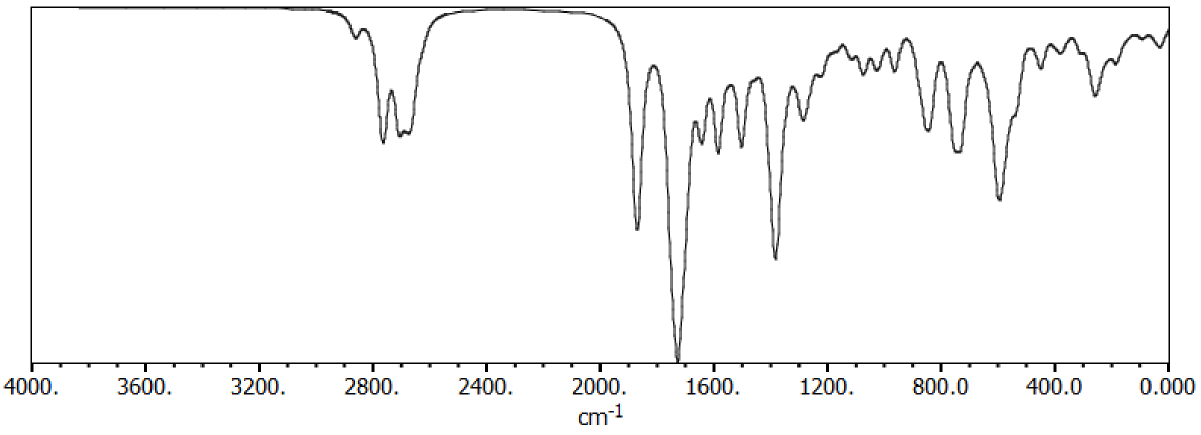

Predicted spectrum of Sm(nal)$_2$

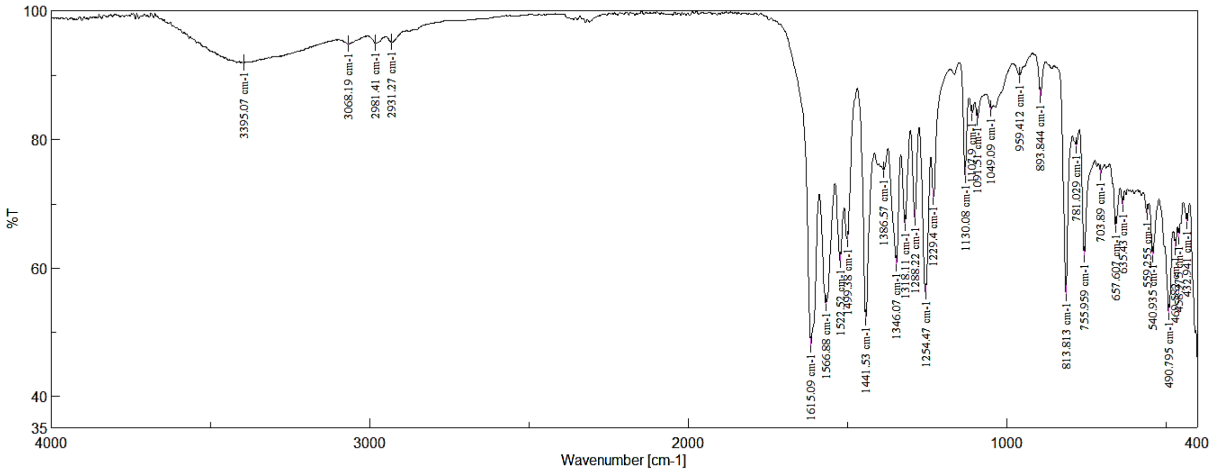

FT-IR spectrum of Eu(nal)$_2$

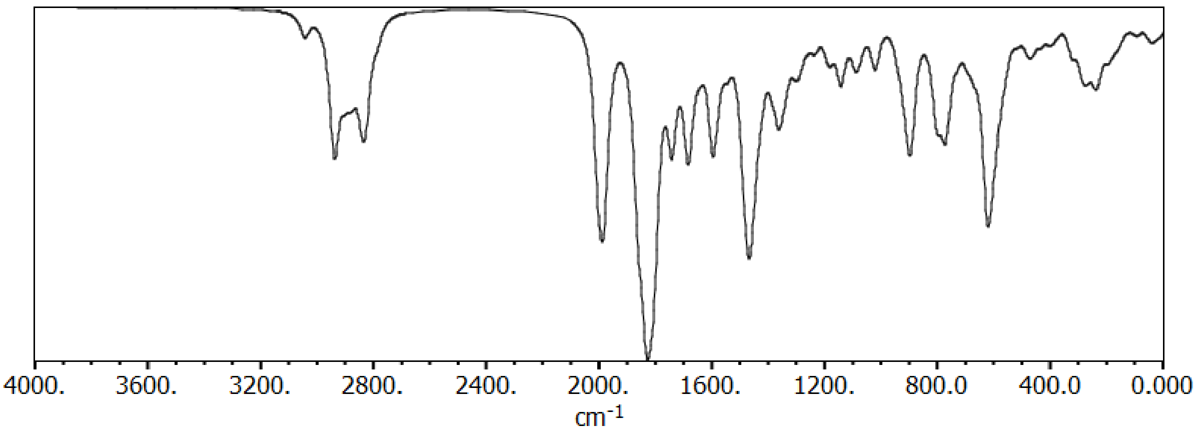

Predicted spectrum of Eu(nal)$_2$

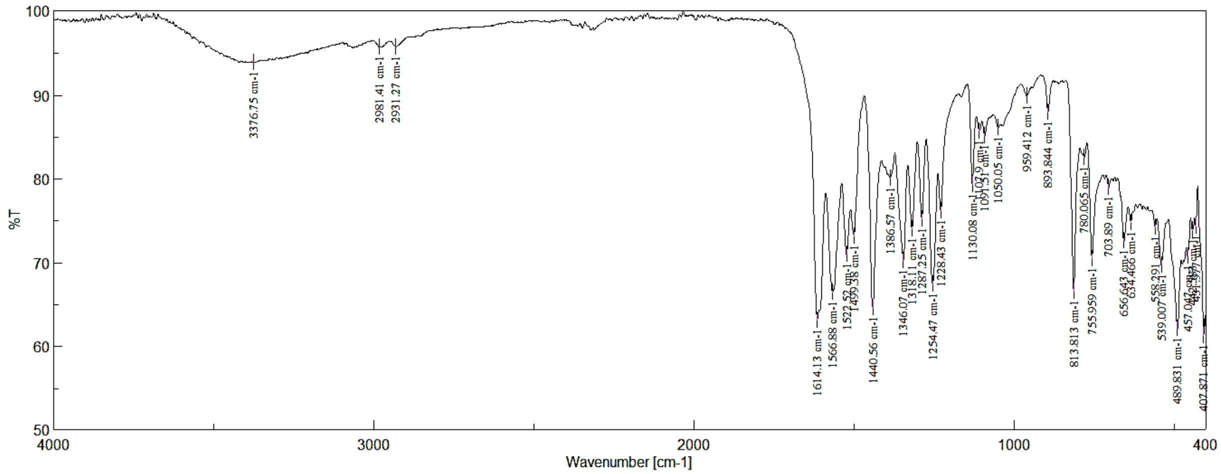

FT-IR spectrum of $Gd(nal)_2$

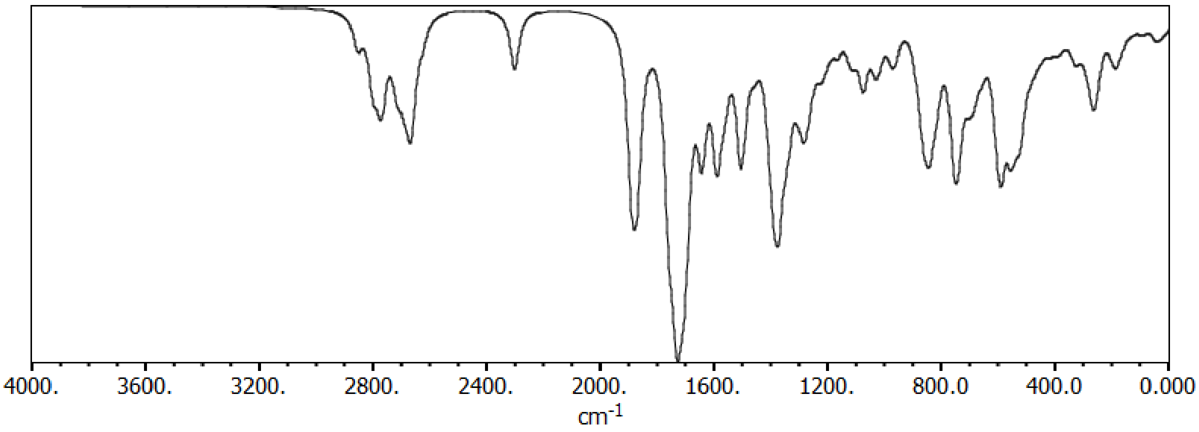

Predicted spectrum of $Gd(nal)_2$

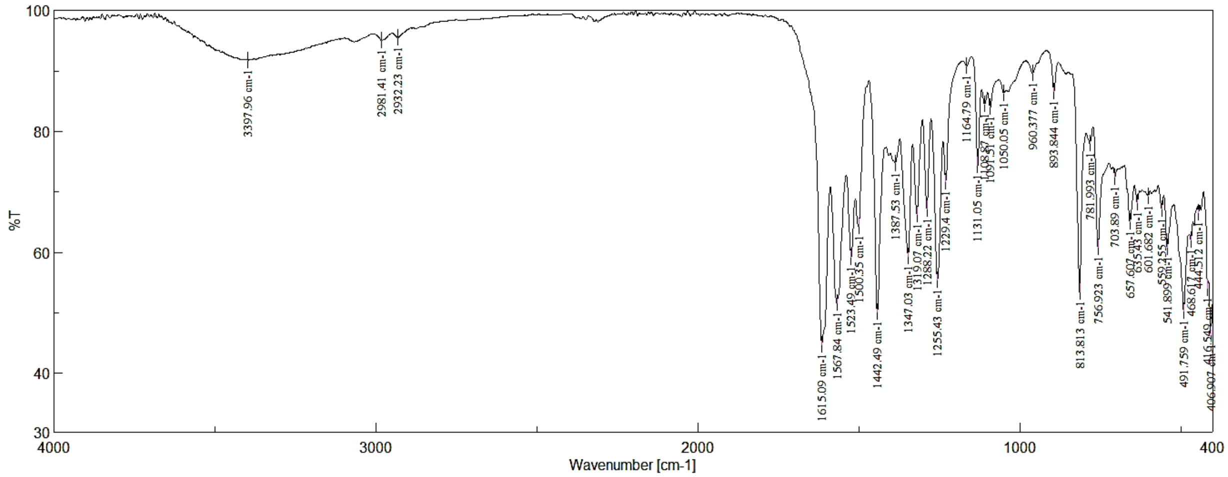

FT-IR spectrum of $Tb(nal)_2$

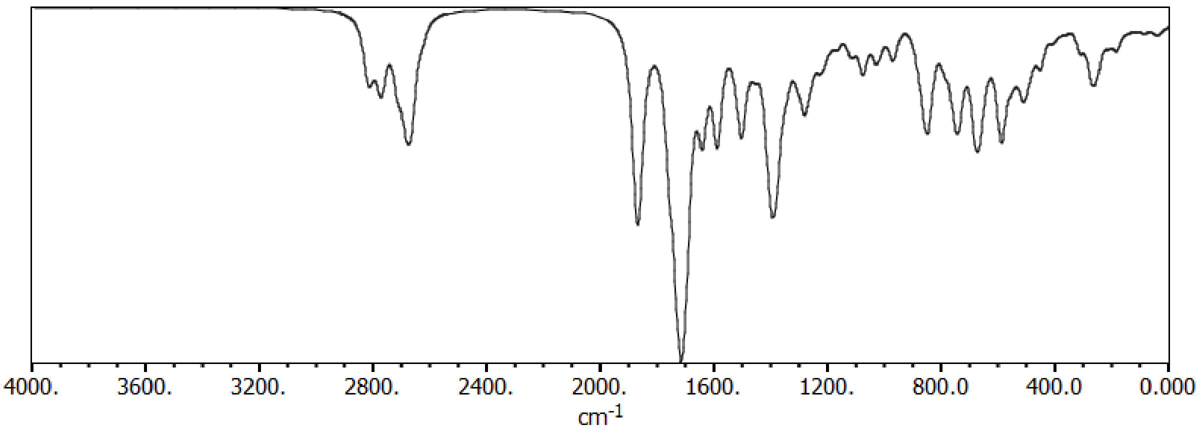

Predicted spectrum of Tb(nal)$_2$

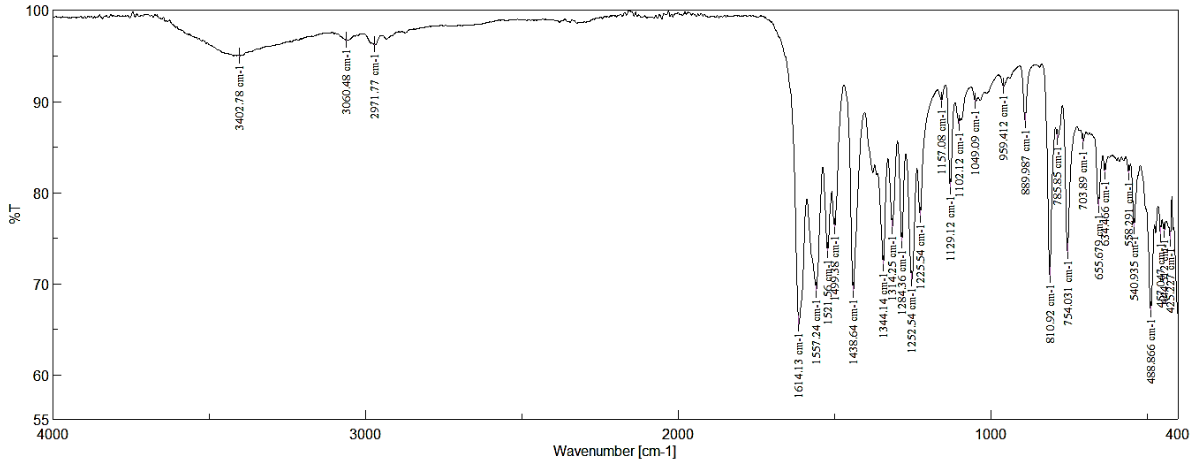

FT-IR spectrum of La(nal)$_3$

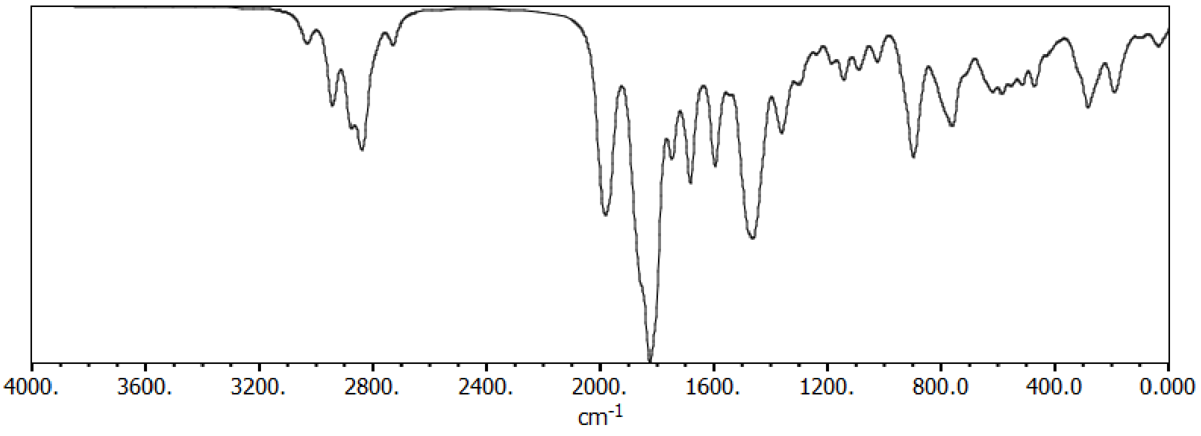

Predicted spectrum of La(nal)$_3$

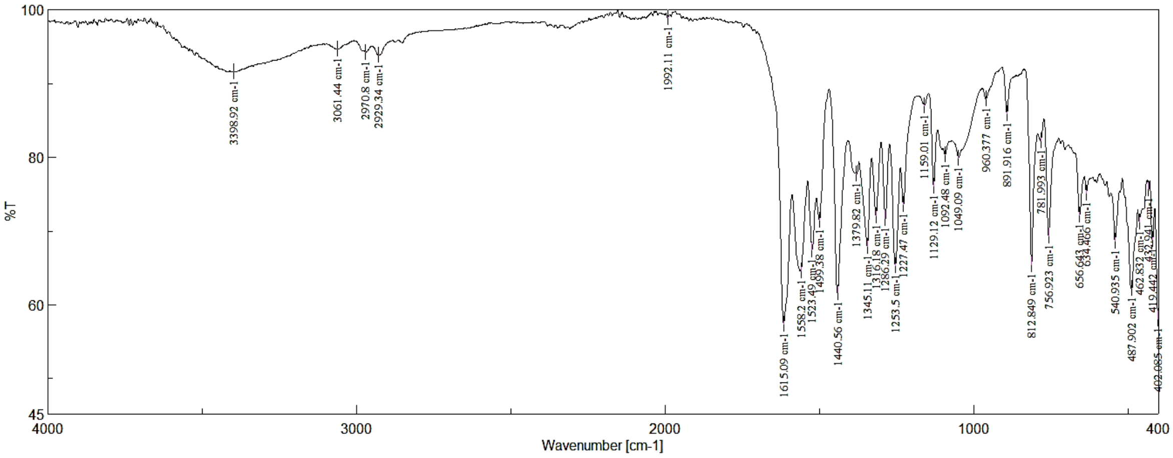

FT-IR spectrum of Eu(nal)$_3$

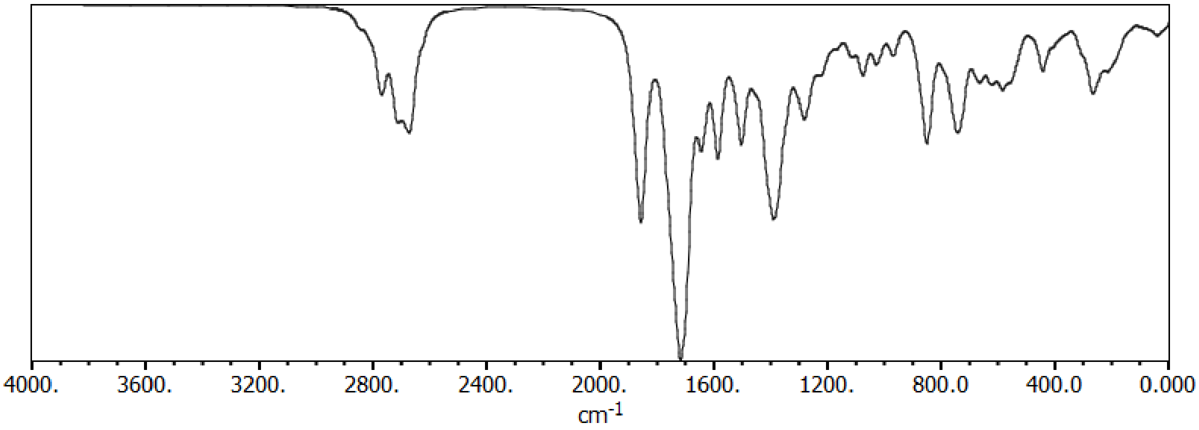

Predicted spectrum of Eu(nal)$_3$

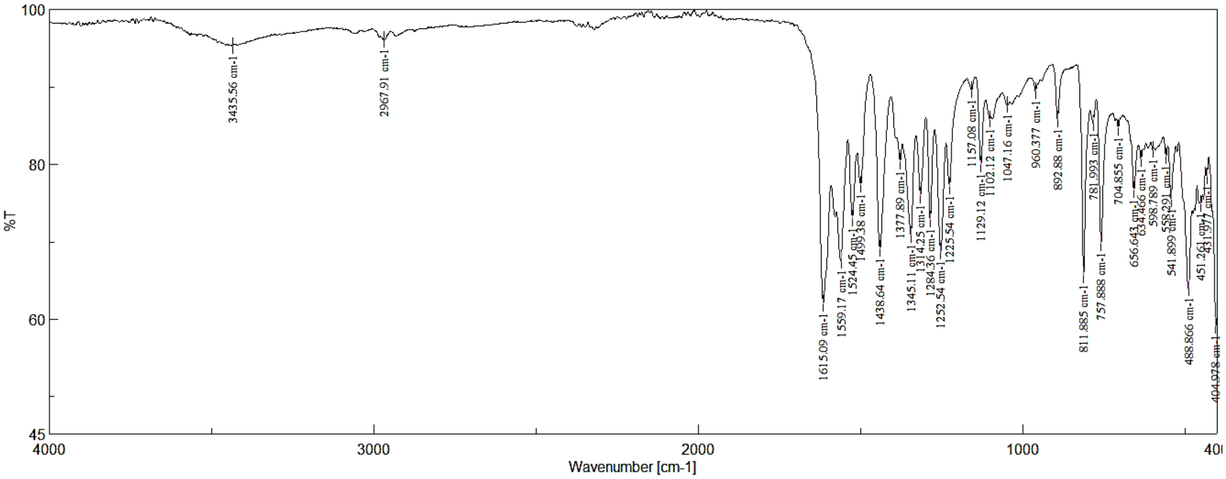

FT-IR spectrum of Gd(nal)$_3$

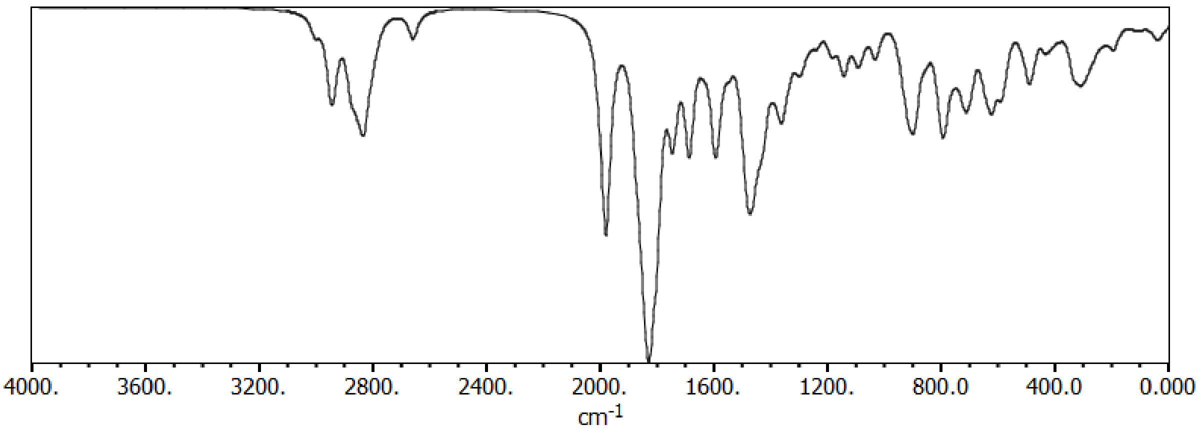

Predicted spectrum of Gd(nal)₃

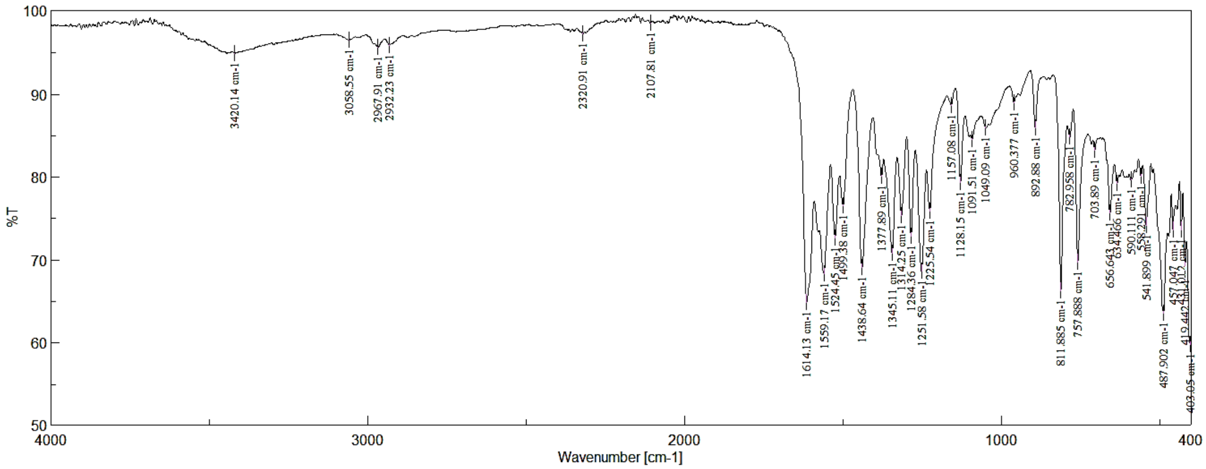

FT-IR spectrum of Tb(nal)₃

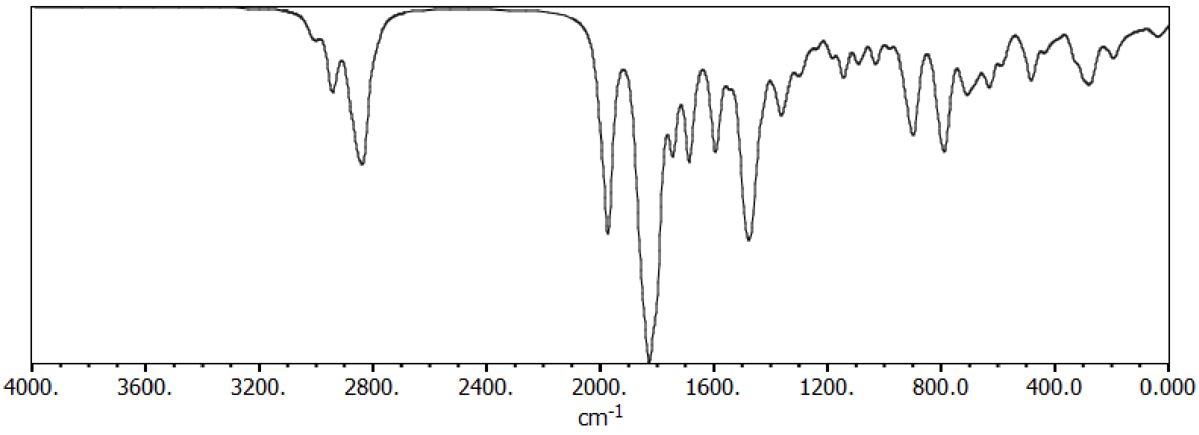

Predicted spectrum of Tb(nal)₃

Figure S1. Experimental and predicted vibrational spectra of nalidixic acid and its M(nal)₂ and M(nal)₃ metal complexes.

A.

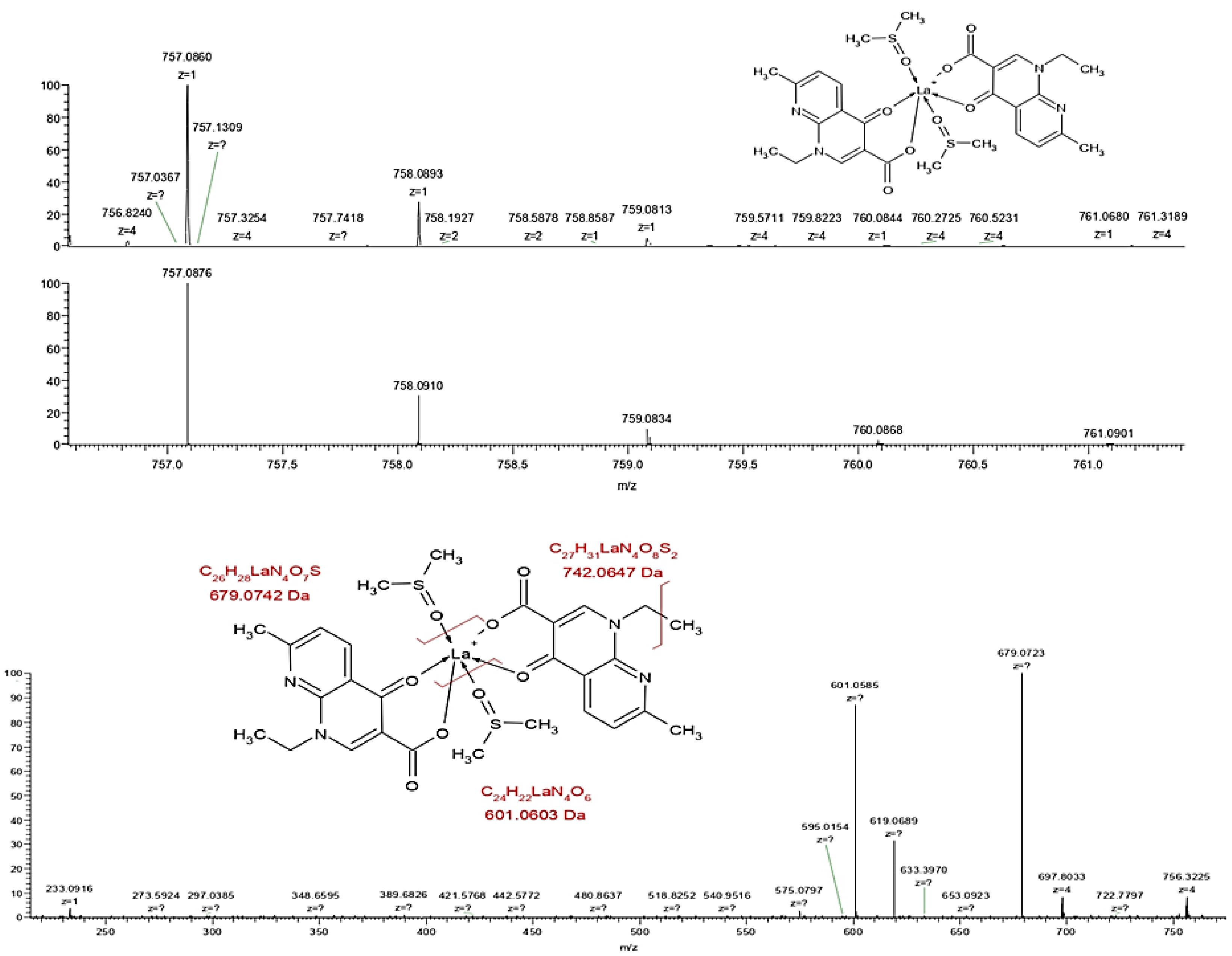

757.0860 z=1
757.1309 z=?
757.0367 z=?
756.8240 z=4
757.3254 z=4
757.7418 z=?
758.0893 z=1
758.1927 z=2
758.5878 z=2
758.8587 z=1
759.0813 z=1
759.5711 z=4
759.8223 z=4
760.0844 z=1
760.2725 z=4
760.5231 z=4
761.0680 z=1
761.3189 z=4
757.0876
758.0910
759.0834
760.0868
761.0901
m/z
$C_{26}H_{28}LaN_4O_7S$
679.0742 Da
$C_{27}H_{31}LaN_4O_8S_2$
742.0647 Da
$C_{24}H_{22}LaN_4O_6$
601.0603 Da
233.0916 z=1
273.5924 z=?
297.0385 z=?
348.6595 z=?
389.6826 z=?
421.5768 z=?
442.5772 z=?
480.8637 z=?
518.8252 z=?
540.9516 z=?
575.0797 z=?
595.0154 z=?
601.0585 z=?
619.0689 z=?
633.3970 z=?
653.0923 z=?
679.0723 z=?
697.8033 z=4
722.7797 z=?
756.3225 z=4
m/z

B.

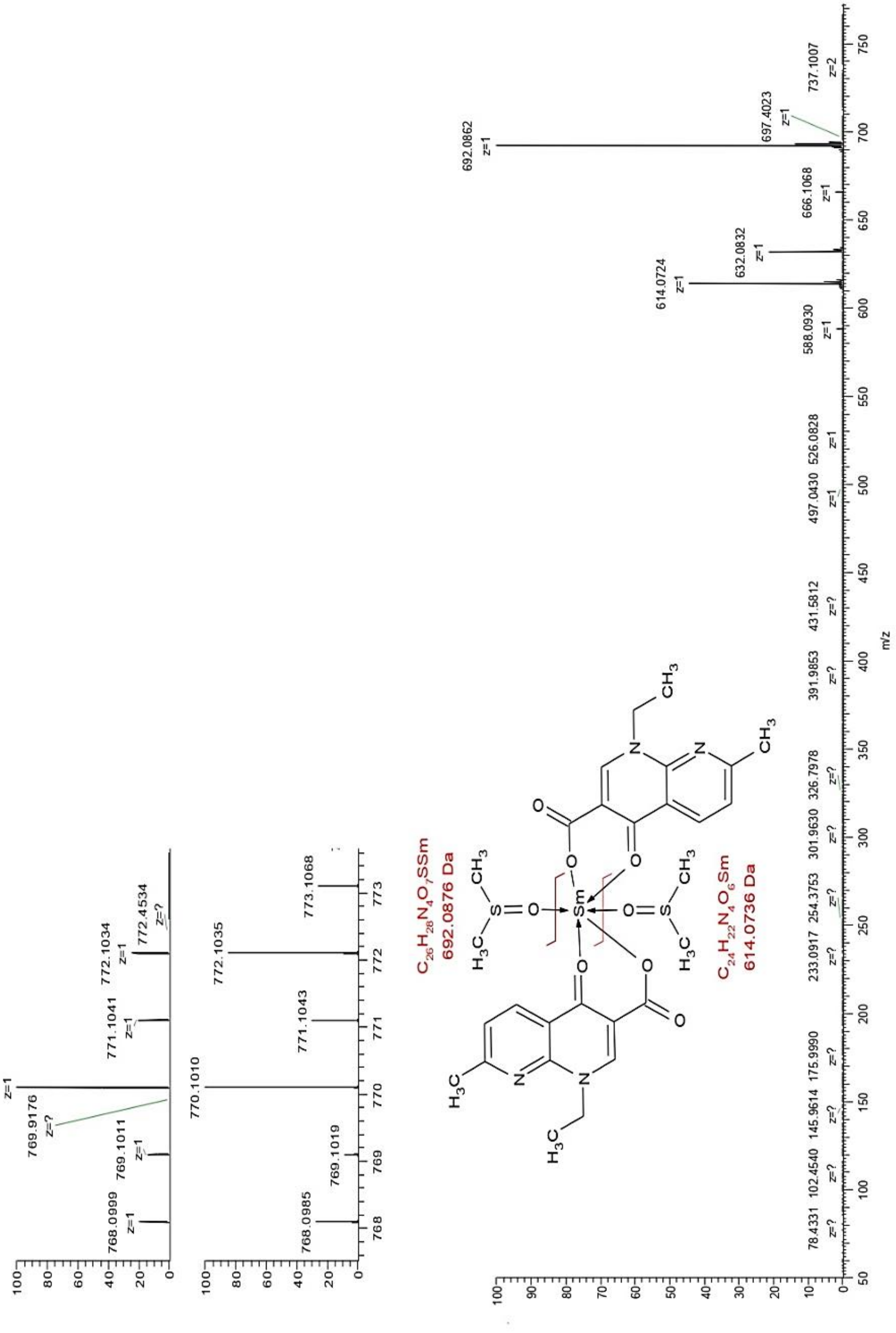

$C_{26}H_{28}N_4O_7SSm$
692.0876 Da
$C_{24}H_{22}N_4O_6Sm$
614.0736 Da
692.0862
614.0724
632.0832
770.1011
772.1034
m/z

C.

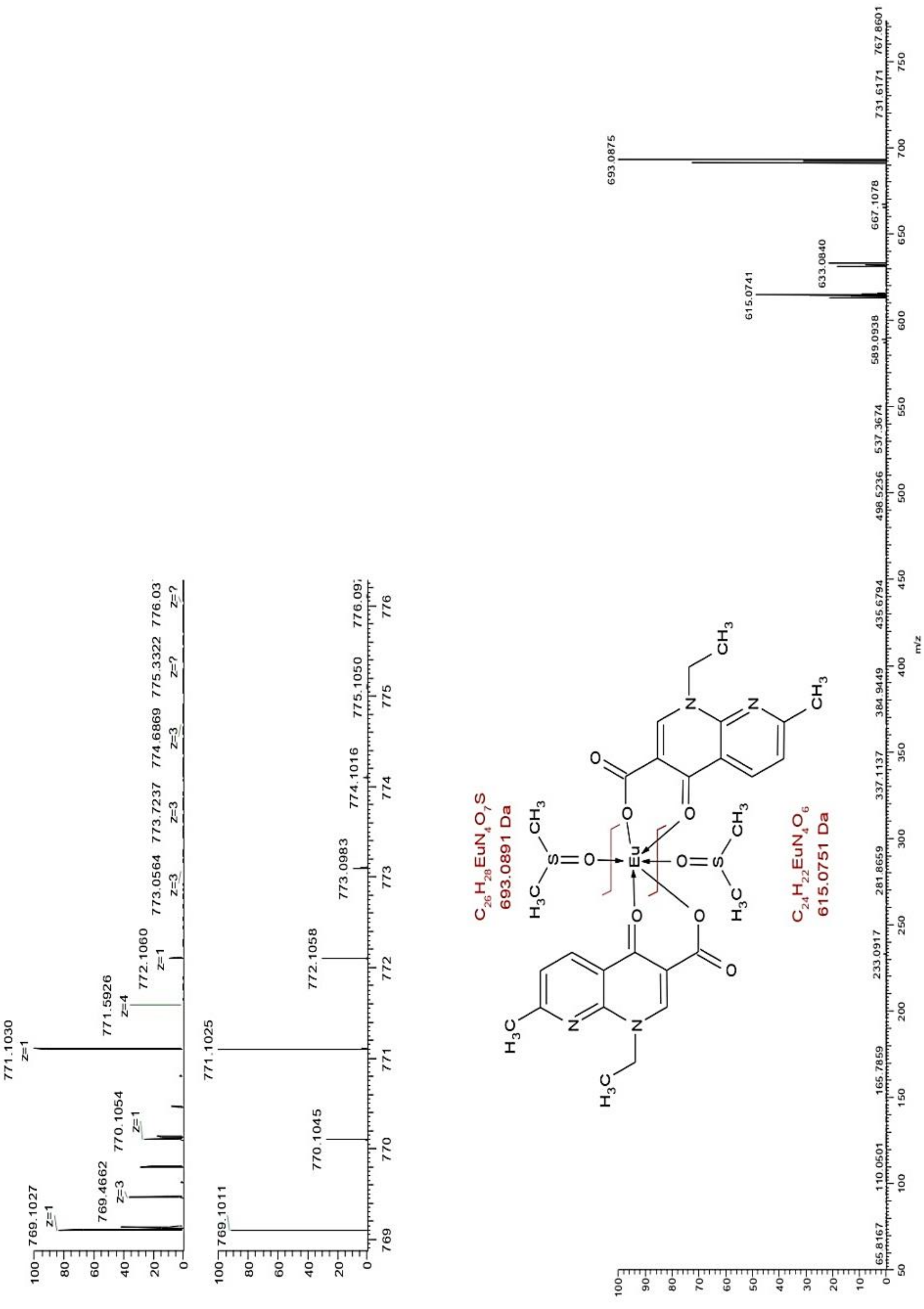

693.0875
615.0741
633.0840
667.1078
589.0938
731.6171
767.8601
537.3674
498.5236
435.6794
384.9449
337.1137
281.8659
233.0917
165.7859
110.0501
65.8167
m/z
C26H28EuN4O7S
693.0891 Da
C24H22EuN4O6
615.0751 Da
769.1027
769.4662
770.1054
771.1030
771.5926
772.1060
773.0564
773.7237
774.6869
775.3322
769.1011
770.1045
771.1025
772.1058
773.0983
774.1016
775.1050

D.

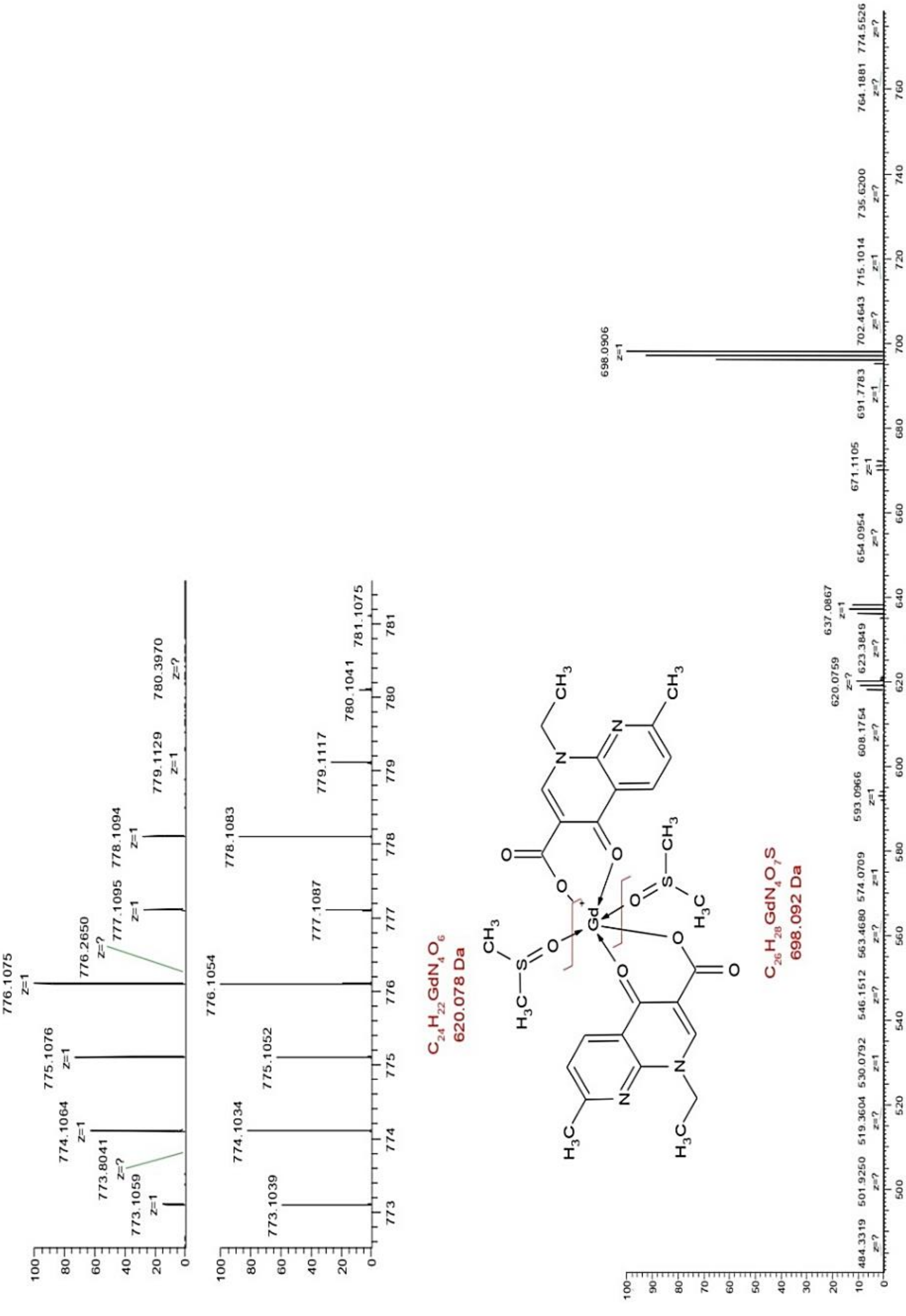

E.

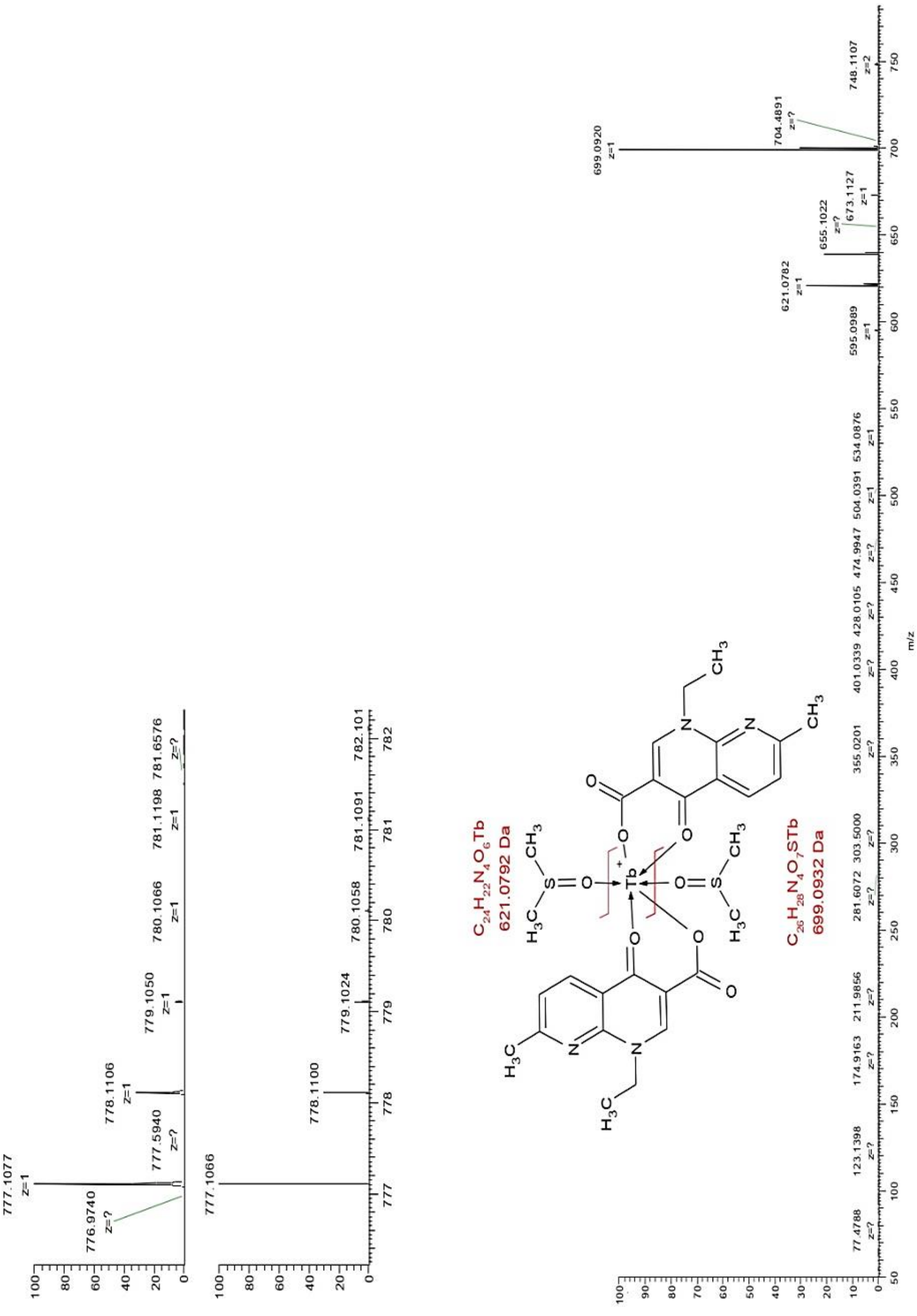

F.

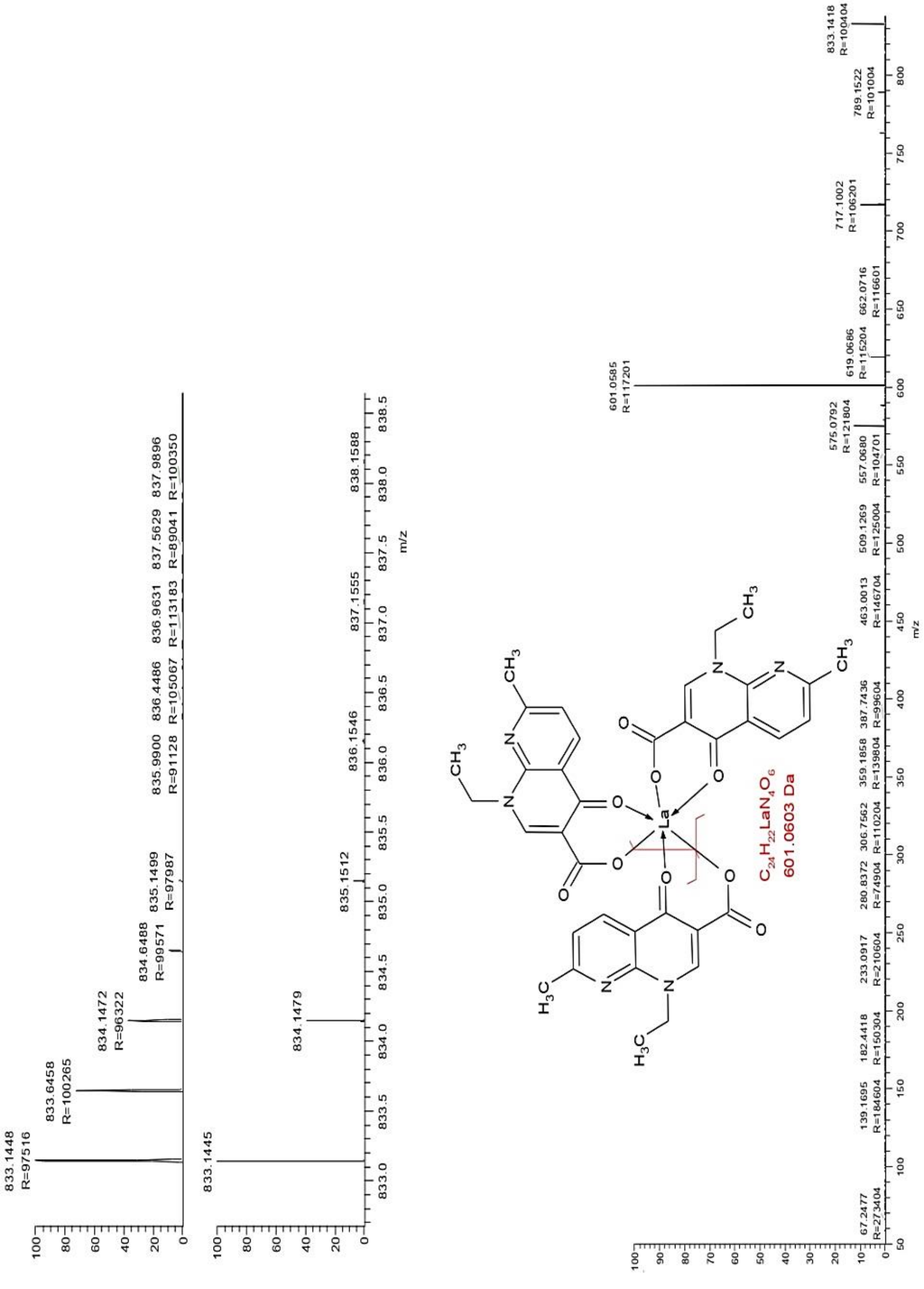

833.1448
R=97516
833.6458
R=100265
834.1472
R=96322
834.6488
R=99571
835.1499
R=97987
835.9900
R=91128
836.4486
R=105067
836.9631
R=113183
837.5629
R=89041
837.9896
R=100350
833.1445
834.1479
835.1512
836.1546
837.1555
838.1588
m/z
601.0585
R=117201
833.1418
R=100404
C24H22LaN4O6
601.0603 Da

G.

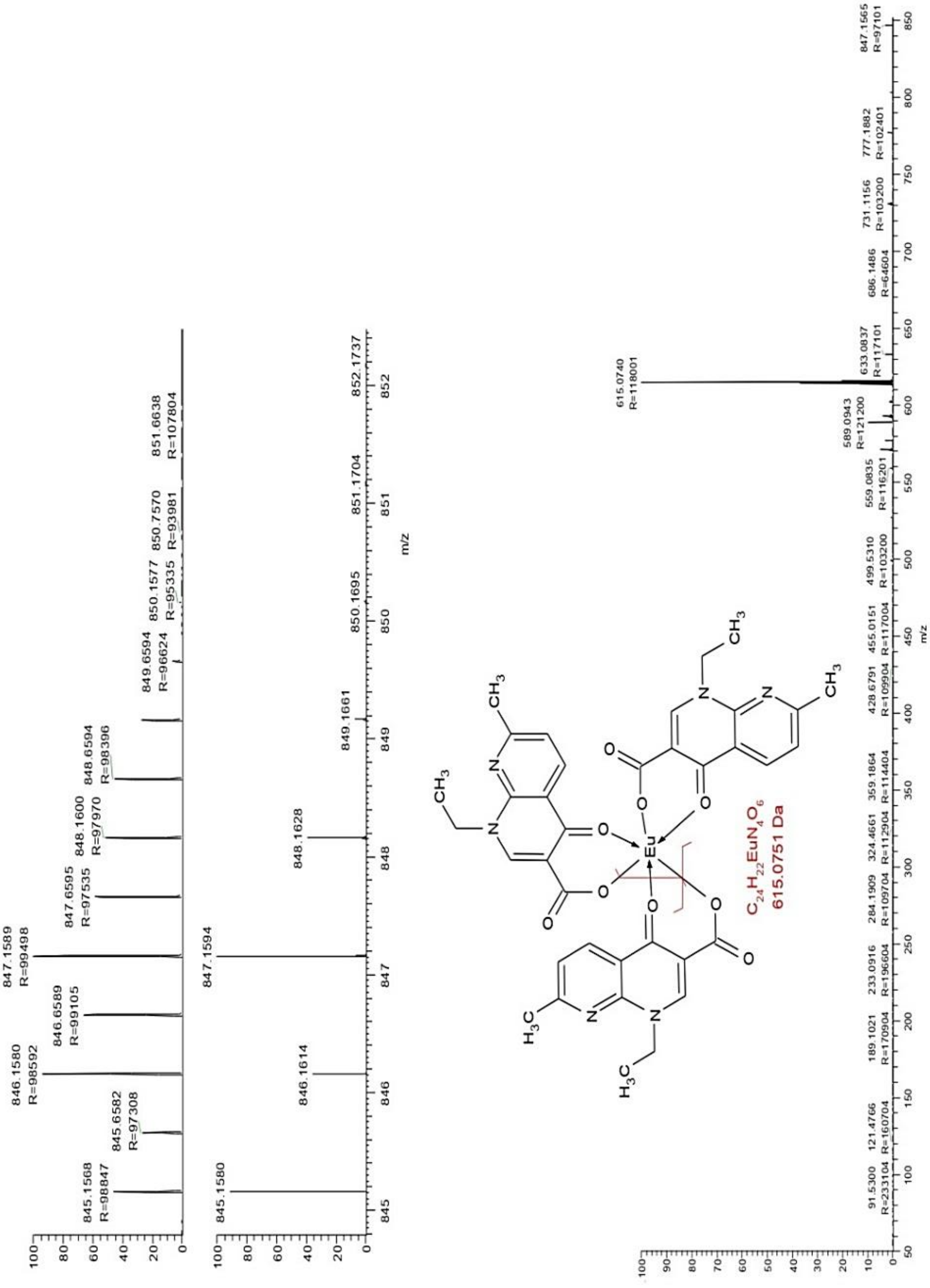

H.

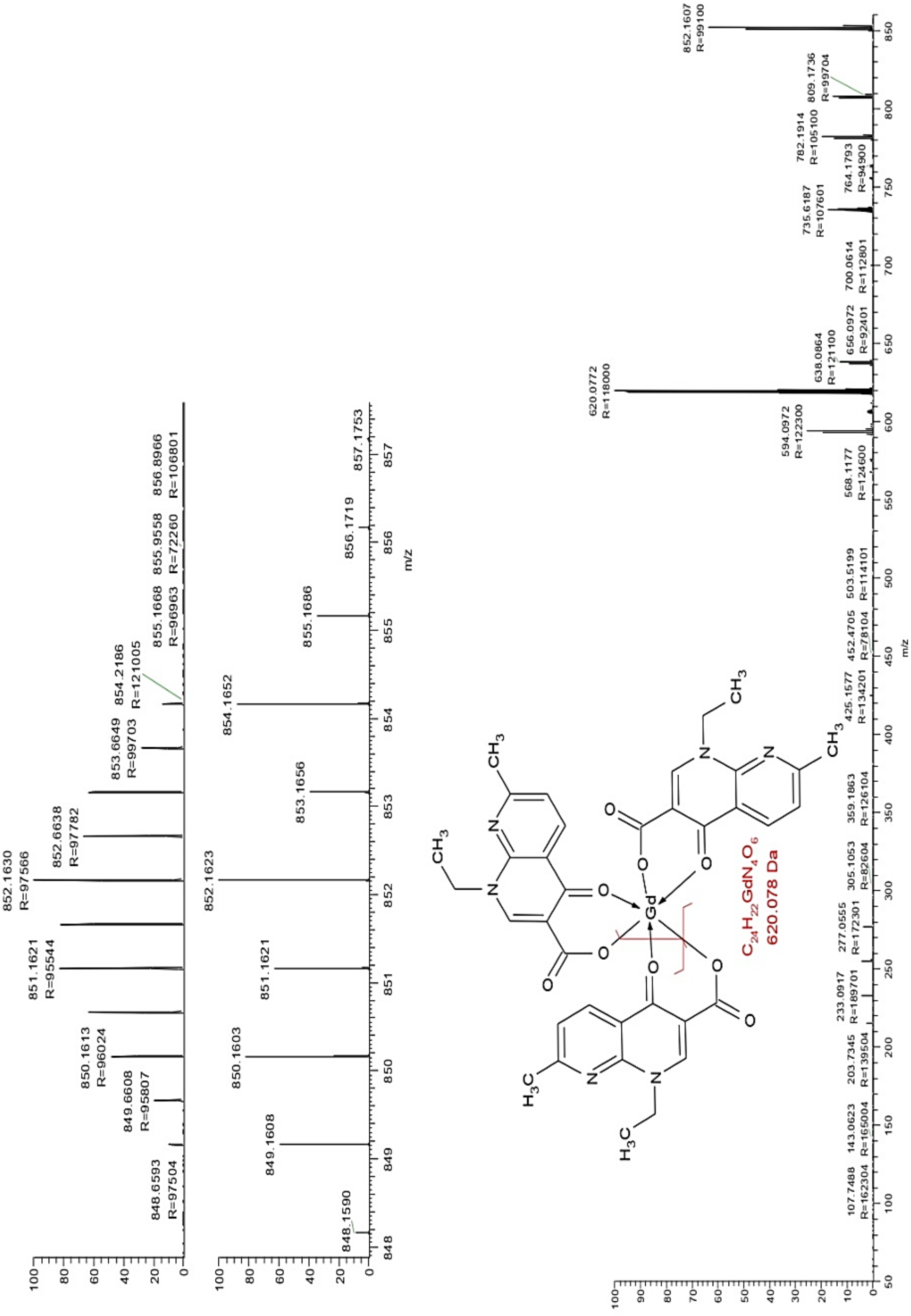

I.

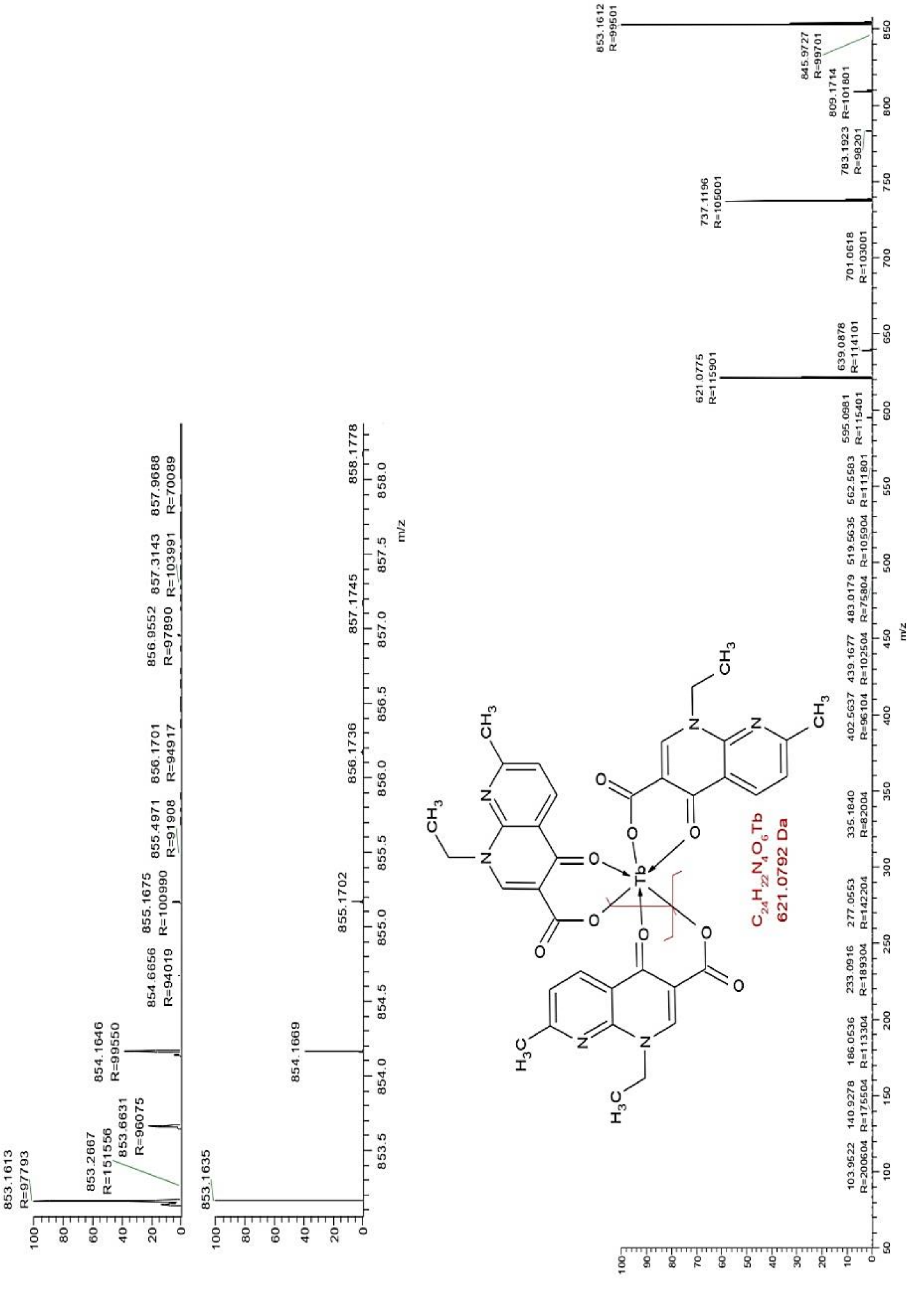

Figure S2. Mass spectra for the synthesized complexes: **A.** La(nal)$_2$; **B.** Sm(nal)$_2$; **C.** Eu(nal)$_2$; **D.** Gd(nal)$_2$; **E.** Tb(nal)$_2$; **F.** La(nal)$_3$; **G.** Eu(nal)$_3$; **H.** Gd(nal)$_3$; **I.** Tb(nal)$_3$.

**La(nal)₂**	**Sm(nal)₂**

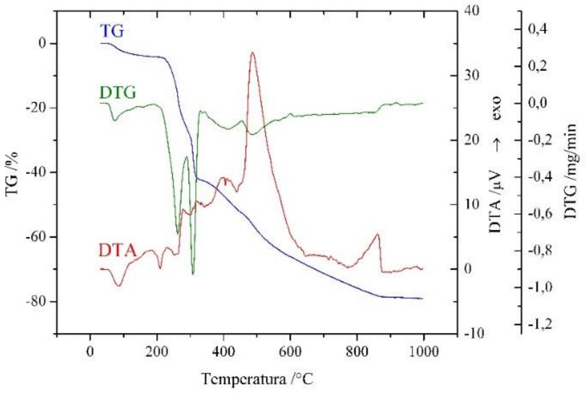

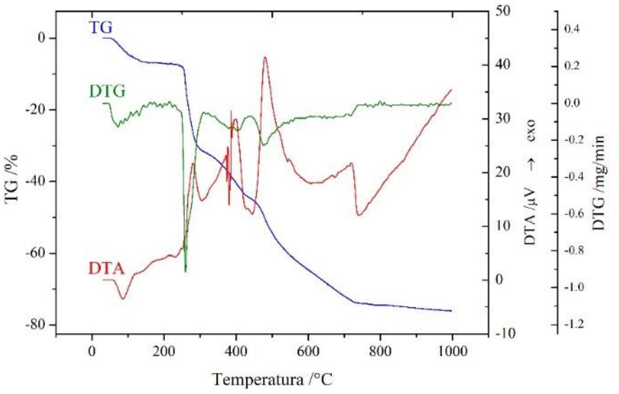

**Eu(nal)₂**	**Gd(nal)₂**

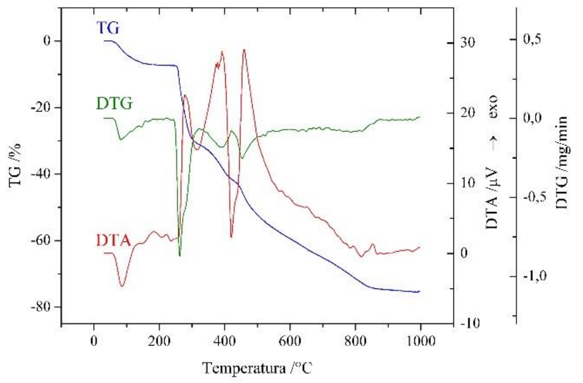

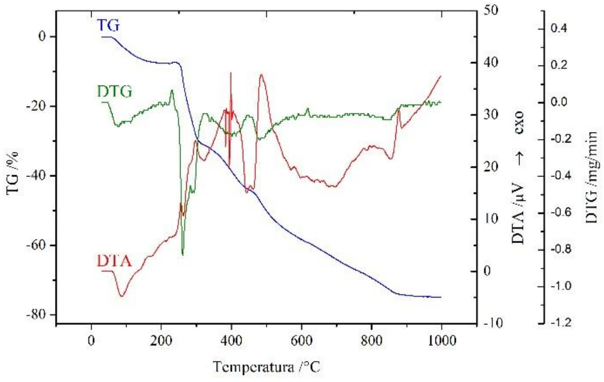

Tb(nal)₂

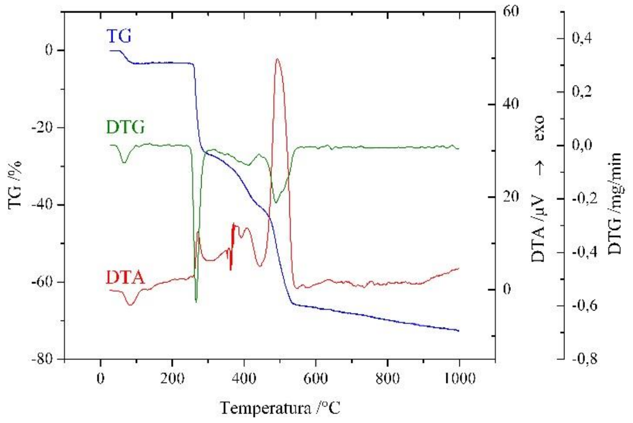

Figure S3. TG, DTG and DTA curves for the M(nal)$_2$ complexes, M= La^{3+}, Sm^{3+}, Eu^{3+}, Gd^{3+}, Tb^{3+}.

Table S4. Thermal decomposition data (in air flow) for the M(nal)$_2$ complexes, M= La^{3+}, Sm^{3+}, Eu^{3+}, Gd^{3+}, Tb^{3+}.

**Complex**	**Step**	**Thermal effect**	**Temperature range / ºC**	Δm_{exp} /%	Δm_{calc} /%
[La(nal)$_2$(OH)(H_2O)]·2H_2O	1	endothermic	55-150	4.3	4.7
	2	exothermic	190-285	23.2	22.9
	3	exothermic	285-340	15.3	15.0
	4	exothermic	340-900	35.9	36.0
		**Residue (1/2 La_2O_3)**		21.3	21.4
[Sm(nal)$_2$(OH)(H_2O)]·3H_2O	1	endothermic	50-220	7.7	7.3
	2	exothermic	220-325	24.9	25.2
	3	exothermic	325-950	42.5	42.7
		**Residue (1/2 Sm_2O_3)**		24.9	24.8
[Eu(nal)$_2$(OH)(H_2O)]·3H_2O	1	endothermic	55-215	7.4	7.7
	2	exothermic	215-335	25.0	24.8
	3	exothermic	335-900	42.8	42.5
		**Residue (1/2 Eu_2O_3)**		24.8	25.0
[Gd(nal)$_2$(OH)(H_2O)]·3H_2O	1	endothermic	50-210	7.5	7.6
	2	exothermic	210-335	24.5	24.7
	3	exothermic	335-950	42.6	42.1
		**Residue (1/2 Gd_2O_3)**		25.4	25.6
[Tb(nal)$_2$(OH)(H_2O)]·1.5H_2O	1	endothermic	50-105	3.6	3.9
	2	exothermic	240-340	25.3	25.6
	3	exothermic	340-950	43.6	43.2
		**Residue (1/4 Tb_4O_7)**		27.5	27.3

La(nal)$_3$	Eu(nal)$_3$
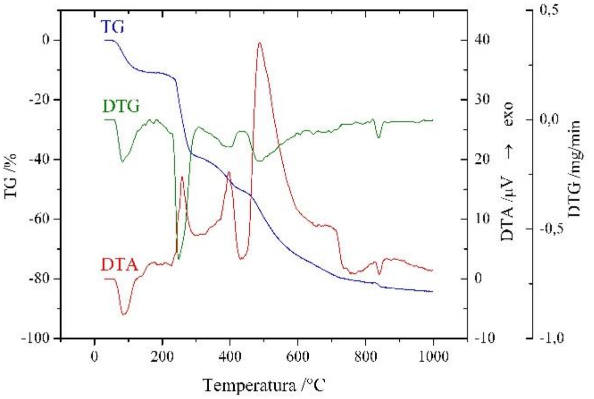	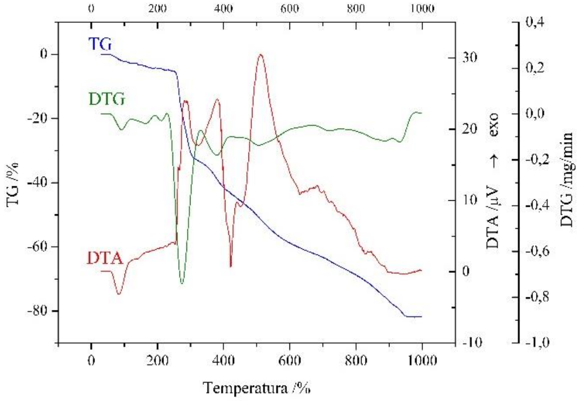

Gd(nal)$_3$	Tb(nal)$_3$
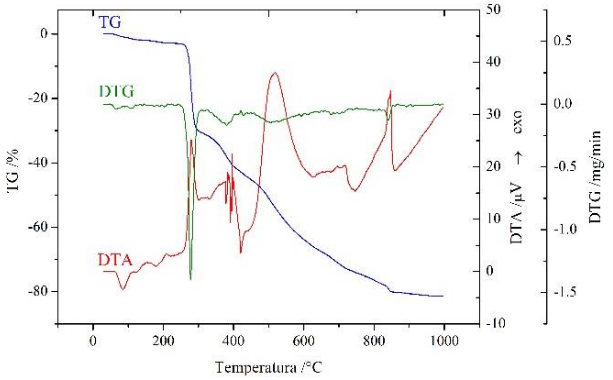	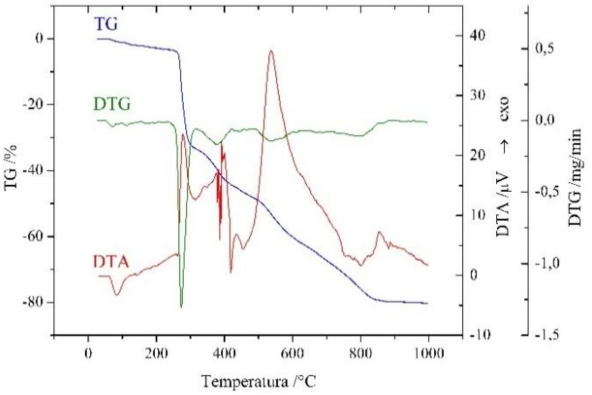

Figure S4. TG, DTG and DTA curves for the M(nal)$_3$ complexes, M= La^{3+}, Eu^{3+}, Gd^{3+}, Tb^{3+}.

Table S5. Thermal decomposition data (in air flow) for the M(nal)$_3$ complexes, M= La^{3+}, Eu^{3+}, Gd^{3+}, Tb^{3+}

Complex	Step	Thermal effect	Temperature range / ºC	Δm_{exp} /%	Δm_{calc} /%
**[La(nal)$_3$(H_2O)$_2$]·6H_2O**	1	endothermic	50-175	10.9	11.1
	2	exothermic	175-305	28.3	28.5
	3	exothermic	305-950	44.0	43.7
		**Residue (1/2 La_2O_3)**		16.8	16.7
**[Eu(nal)$_3$(H_2O)$_2$]·2.5H_2O**	1	endothermic	50-225	4.7	4.9
	2	exothermic	225-330	28.1	28.2
	3	exothermic	330-960	48.0	47.9
		**Residue (1/2 Eu_2O_3)**		19.2	19.0
**[Gd(nal)$_3$(H_2O)$_2$]·1.5H_2O**	1	endothermic	55-220	2.9	2.9
	2	exothermic	220-325	28.6	28.6
	3	exothermic	325-980	48.6	48.7
		**Residue (1/2 Gd_2O_3)**		19.9	19.8
**[Tb(nal)$_3$(H_2O)$_2$]·1.5H_2O**	1	endothermic	55-235	3.0	2.9
	2	exothermic	235-335	29.3	29.5
	3	exothermic	335-900	47.7	47.8
		**Residue (1/4 Tb_4O_7)**		20.0	19.8

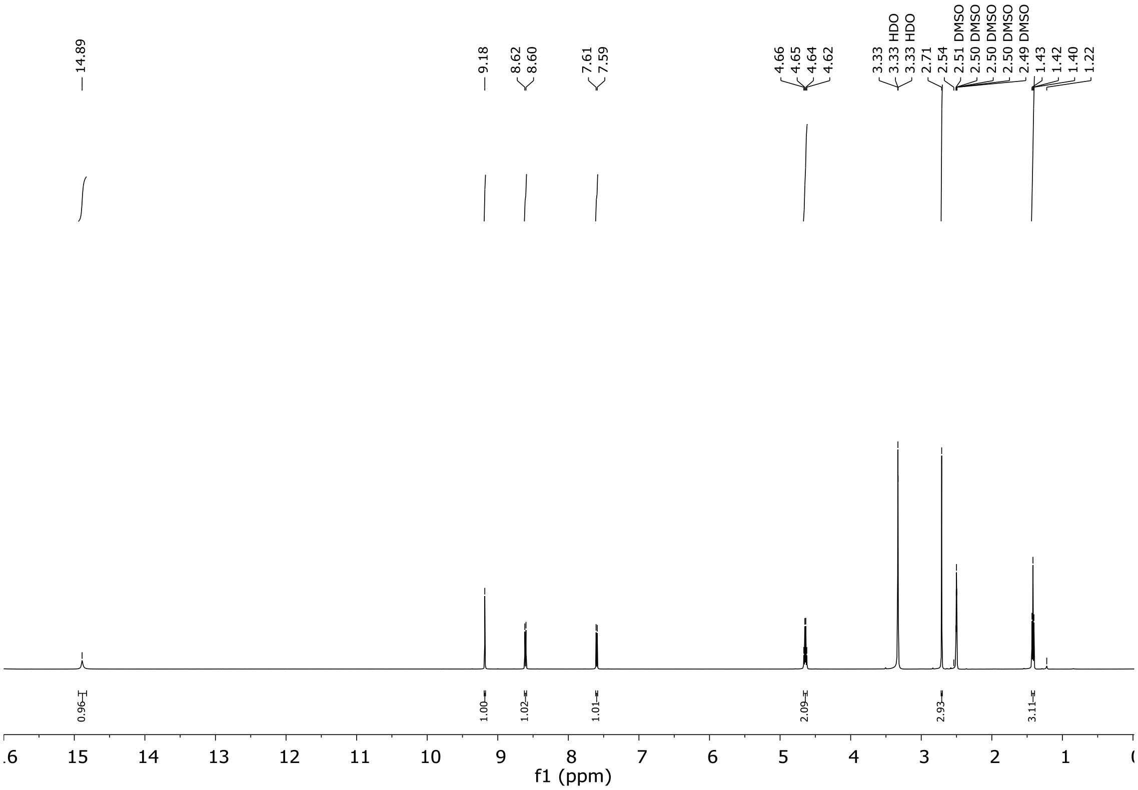

Figure S5. ^{1}H-NMR spectrum of nalidixic acid.

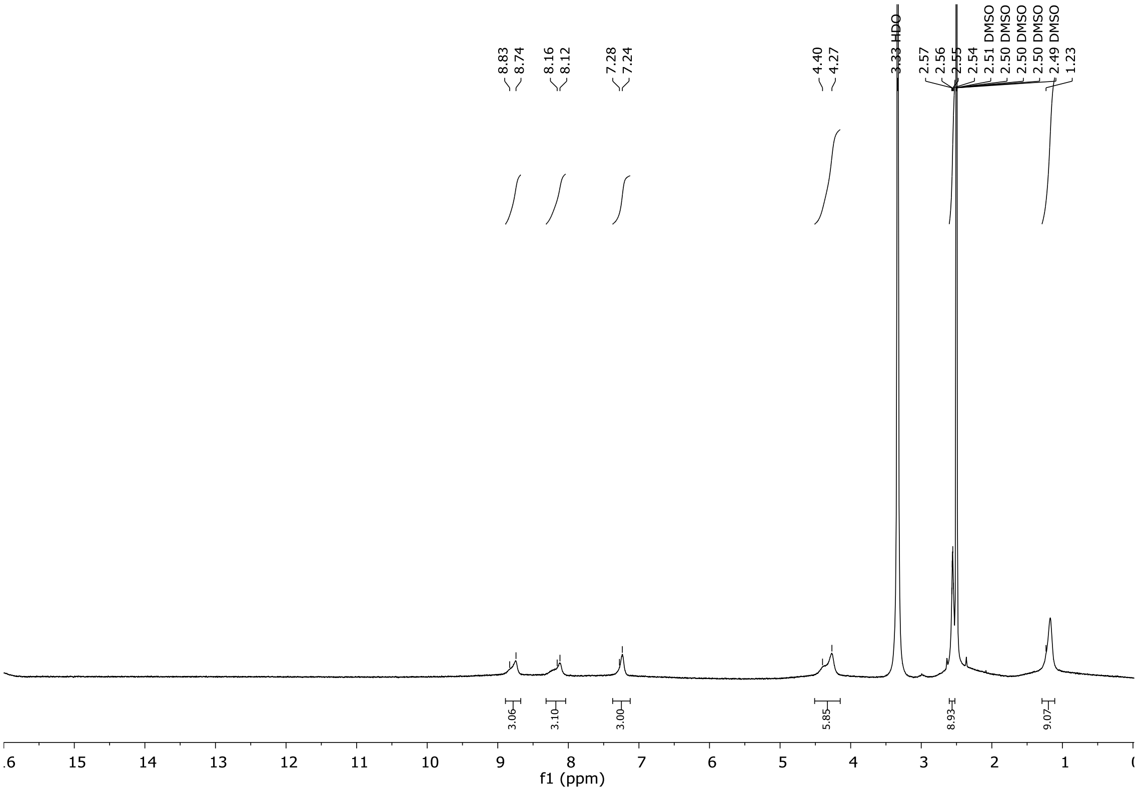

Figure S6. ^{1}H-NMR spectrum of La(nal)$_3$.

Table S6. Geometric parameters: bond lengths, bond angles, dihedral angles, charge density, total energy of M(nal)$_2$ and M(nal)$_3$.

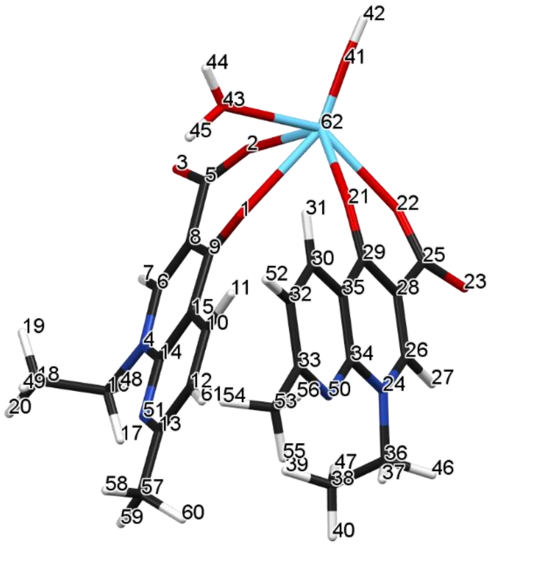 **La(nal)$_2$**	**Bond length (Å)**		**Mulliken atomic charge**	
	La-O1	2.565	La62	0.679
	La-O2	2.412	O1	-0.342
	La-O21	2.564	O2	-0.458
	La-O22	2.411	O21	-0.354
	La-O41	1.493	O22	-0.452
	La-O43	2.625	O41	0.112
			O43	-0.307
			N4	-0.304
			N24	-0.301
			N50	-0.225
			N51	-0.227
**Total energy (a.u.): -1778.62**	**Bond angle (°)**		**Torsion angle**	
	O1-La-O2	62.35	O41-La-O22-C25	126.31
	O21-La-O22	62.73	O43-La-O2-C5	-70.32
	O41-La-O43	92.38	O43-La-O21-C29	128.66
	O41-La-O22	117.29	O41-La-O1-C9	125.93
	O2-La-O43	86.62	La-O22-C25-O23	176.10
	O1-La-O21	66.04	La-O2-C5-O3	176.66
	La-O22-C25	152.10	C8-C14-C28-C34	141.95
	La-O2-C5	153.33		
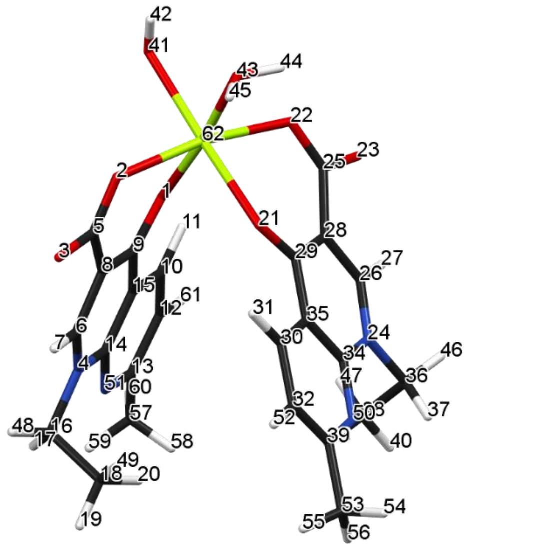 **Sm(nal)$_2$**	**Bond length (Å)**		**Mulliken atomic charge**	
	Sm-O1	2.303	Sm62	0.871
	Sm-O2	2.299	O1	-0.455
	Sm-O21	2.301	O2	-0.442
	Sm-O22	2.296	O21	-0.458
	Sm-O41	2.320	O22	-0.441
	Sm-O43	2.320	O41	-0.610
			O43	-0.245
			N4	-0.231
			N24	-0.232
			N50	-0.226
			N51	-0.223

	**Bond angle (°)**		**Torsion angle**	
			O41-Sm-O22-C25	-146.83
	O1-Sm-O2	84.12	O43-Sm-O2-C5	-151.54
	O21-Sm-O22	84.42	O43-Sm-O21-C29	-130.98
	O41-Sm-O43	84.21	O41-Sm-O1-C9	-127.69
**Total energy (a.u.): -2405.35**	O41-Sm-O22	95.80	Sm-O22-C25-O23	156.65
	O2-Sm-O43	94.11	Sm-O2-C5-O3	158.94
	O1-Sm-O21	92.67	C8-C14-C28-C34	106.51
	Sm-O22-C25	120.84		
	Sm-O2-C5	121.80		
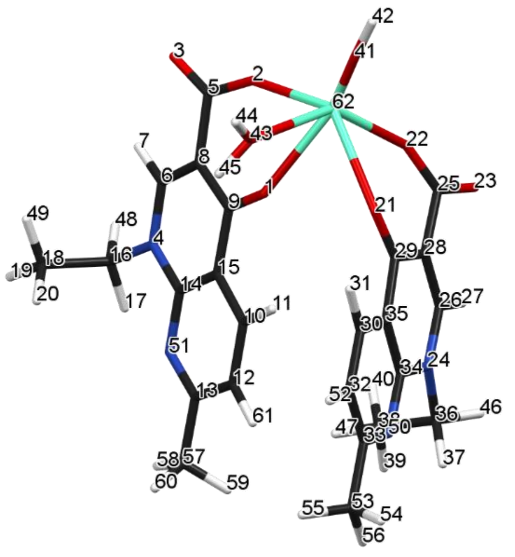	**Bond length (Å)**		**Mulliken atomic charge**	
	Eu-O1	2.432	Eu62	0.973
	Eu-O2	2.313	O1	-0.334
	Eu-O21	2.431	O2	-0.452
	Eu-O22	2.310	O21	-0.351
	Eu-O41	1.503	O22	-0.447
	Eu-O43	2.484	O41	-0.236
			O43	-0.297
			N4	-0.302
			N24	-0.299
			N50	-0.226
			N51	-0.226
**Eu(nal)$_2$**				
	**Bond angle (°)**		**Torsion angle**	
			O41-Eu-O22-C25	123.70
	O1-Eu-O2	64.48	O43-Eu-O2-C5	-73.01
	O21-Eu-O22	65.11	O43-Eu-O21-C29	131.90
**Total energy (a.u.): -2457.44**	O41-Eu-O43	90.62	O41-Eu-O1-C9	122.66
	O41-Eu-O22	116.03	Eu-O22-C25-O23	176.70
	O2-Eu-O43	87.53	Eu-O2-C5-O3	177.79
	O1-Eu-O21	67.02	C8-C14-C28-C34	146.86
	Eu-O22-C25	151.51		
	Eu-O2-C5	152.87		

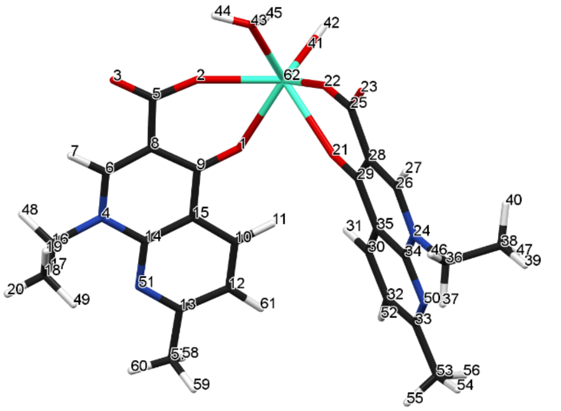<br>**Gd(nal)$_2$**	**Bond length (Å)**<br>Gd-O1<br>Gd-O2<br>Gd-O21<br>Gd-O22<br>Gd-O41<br>Gd-O43	 <br>2.389<br>2.309<br>2.388<br>2.310<br>1.511<br>2.443	**Mulliken atomic charge**<br>Gd62<br>O1<br>O2<br>O21<br>O22<br>O41<br>O43<br>N4<br>N24<br>N50<br>N51	 <br>1.016<br>-0.373<br>-0.462<br>-0.374<br>-0.463<br>-0.150<br>-0.317<br>-0.299<br>-0.300<br>-0.226<br>-0.226
**Total energy (a.u.): -2512.80**	**Bond angle (°)**<br>O1-Gd-O2<br>O21-Gd-O22<br>O41-Gd-O43<br>O41-Gd-O22<br>O2-Gd-O43<br>O1-Gd-O21<br>Gd-O22-C25<br>Gd-O2-C5	 <br>63.51<br>63.51<br>110.04<br>118.52<br>64.29<br>72.96<br>154.71<br>154.76	**Torsion angle**<br>O41-Gd-O22-C25<br>O43-Gd-O2-C5<br>O43-Gd-O21-C29<br>O41-Gd-O1-C9<br>Gd-O22-C25-O23<br>Gd-O2-C5-O3<br>C8-C14-C28-C34	 -105.40<br>-157.74<br>-15.28<br>-121.31<br>-175.93<br>176.26<br>178.80
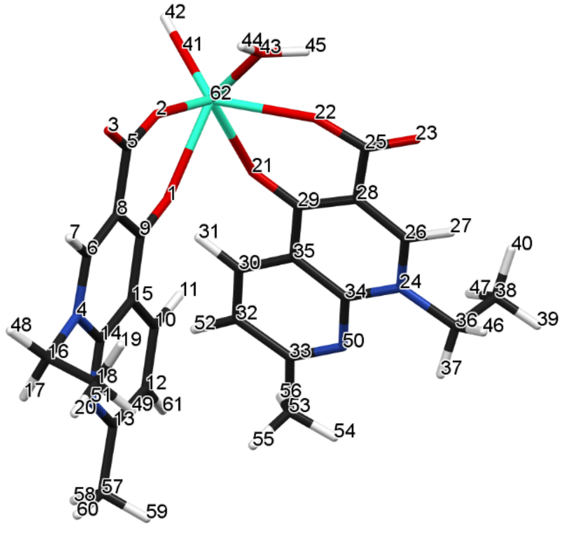<br>**Tb(nal)$_2$**	**Bond length (Å)**<br>Tb-O1<br>Tb-O2<br>Tb-O21<br>Tb-O22<br>Tb-O41<br>Tb-O43	 <br>2.365<br>2.294<br>2.375<br>2.290<br>1.509<br>2.425	**Mulliken atomic charge**<br>Tb62<br>O1<br>O2<br>O21<br>O22<br>O41<br>O43<br>N4<br>N24<br>N50<br>N51	 <br>1.001<br>-0.367<br>-0.469<br>-0.370<br>-0.467<br>-0.159<br>-0.311<br>-0.300<br>-0.301<br>-0.225<br>-0.226
**Total energy (a.u.): -2570.98**	**Bond angle (°)**<br>O1-Tb-O2<br>O21-Tb-O22<br>O41-Tb-O43<br>O41-Tb-O22<br>O2-Tb-O43<br>O1-Tb-O21<br>Tb-O22-C25<br>Tb-O2-C5	 <br>64.46<br>64.75<br>111.05<br>124.58<br>66.13<br>68.52<br>153.20<br>153.60	**Torsion angle**<br>O41-Tb-O22-C25<br>O43-Tb-O2-C5<br>O43-Tb-O21-C29<br>O41-Tb-O1-C9<br>Tb-O22-C25-O23<br>Tb-O2-C5-O3<br>C8-C14-C28-C34	 -98.79<br>-131.52<br>8.75<br>-110.54<br>-176.44<br>173.14<br>-158.87

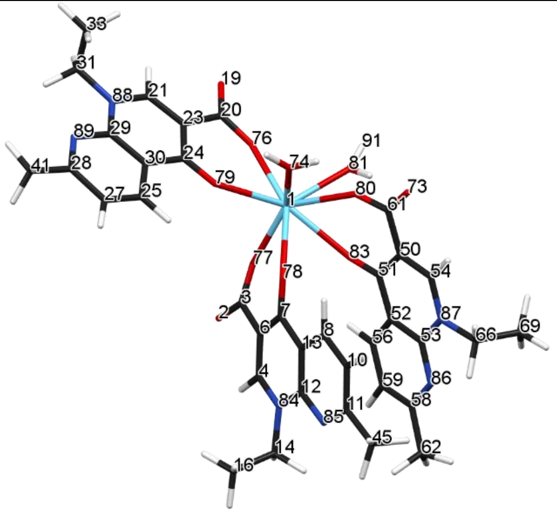	**Bond length (Å)**		**Mulliken atomic charge**	
	La1-O74	2.615	La1	1.183
	La1-O81	2.524	O74	-0.304
	La1-O80	2.487	O81	-0.308
	La1-O83	2.563	O80	-0.504
	La1-O77	2.374	O83	-0.362
	La1-O78	2.557	O77	-0.467
	La1-O76	2.338	O78	-0.376
	La1-O79	2.548	O76	-0.470
			O79	-0.379
			N84	-0.299
			N85	-0.228
			N86	-0.227
			N87	-0.300
**La(nal)$_3$**			N88	-0.298
			N89	-0.224
	**Bond angle (°)**		**Torsion angle**	
			O74-La-O80-C61	111.35
	O74-La-O81	101.13	O74-La-O83-C51	12.16
	O81-La-O80	60.36	O74-La-O78-C7	-179.90
	O80-La-O83	59.77	O74-La-O77-C3	-50.01
	O83-La-O78	63.06	O74-La-O79-C24	113.87
**Total energy (a.u.): -2577.37**	O78-La-O77	62.95	O74-La-O76-C20	-59.61
	O77-La-O79	91.23	O74-La-O81-H91	-90.16
	O79-La-O76	62.79	La-O76-C20-O19	175.68
	O76-La-O74	89.32	La-O80-C61-O73	-148.64
	La-O80-C61	148.86	La-O77-C3-O2	174.01
	La-O79-C24	140.94	N87-C51-C6-C12	2.06
			N88-C24-N87-C51	-90.47
			N88-C24-N84-C7	-174.19
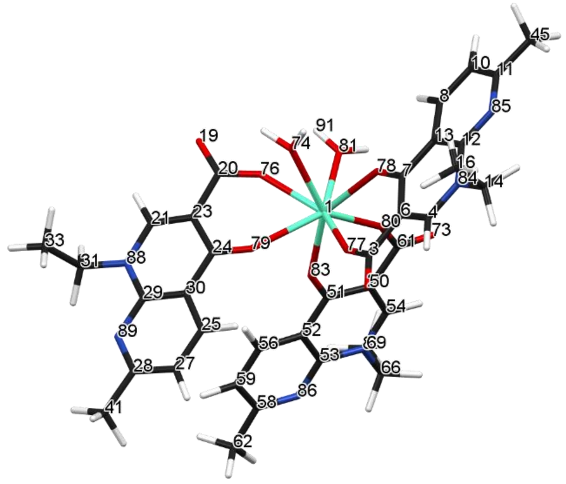	**Bond length (Å)**		**Mulliken atomic charge**	
	Eu1-O74	2.475	Eu1	1.069
	Eu1-O81	2.474	O74	-0.287
	Eu1-O80	2.321	O81	-0.283
	Eu1-O83	2.415	O80	-0.441
	Eu1-O77	2.285	O83	-0.347
	Eu1-O78	2.419	O77	-0.442
	Eu1-O76	2.357	O78	-0.361
	Eu1-O79	2.418	O76	-0.438
			O79	-0.354
			N84	-0.298
			N85	-0.225
			N86	-0.226
			N87	-0.300
**Eu(nal)$_3$**			N88	-0.301
			N89	-0.226

	**Bond angle**		**Torsion angle**	
	(°)		O74-Eu-O80-C61	-143.82
	O74-Eu-O81	83.57	O74-Eu-O83-C51	146.47
	O81-Eu-O80	63.57	O74-Eu-O78-C7	114.44
	O80-Eu-O83	64.02	O74-Eu-O77-C3	-61.42
	O80-Eu-O78	90.78	O74-Eu-O79-C24	90.12
**Total energy (a.u.): -3256.03**	O78-Eu-O77	64.38	O74-Eu-O76-C20	-115.25
	O77-Eu-O79	86.48	O74-Eu-O81-H91	-58.39
	O79-Eu-O76	63.70	Eu-O76-C20-O19	-176.32
	O76-Eu-O74	62.86	Eu-O80-C61-O73	165.00
	Eu-O80-C61	153.45	Eu-O77-C3-O2	177.21
	Eu-O79-C24	141.19	N87-C51-C6-C12	70.13
			N88-C24-N87-C51	165.01
			N88-C24-N84-C7	-99.73
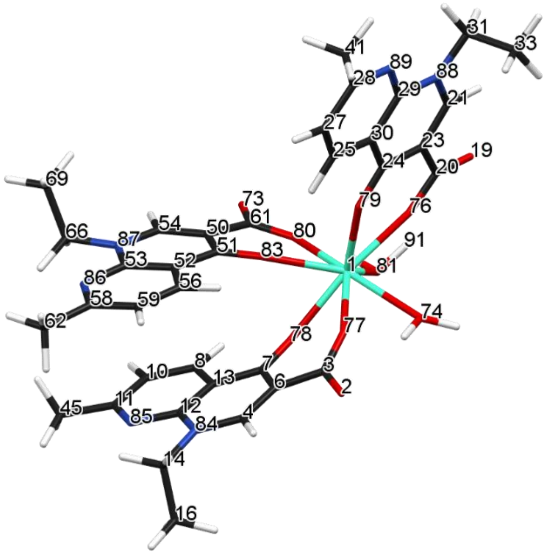	**Bond length (Å)**		**Mulliken atomic charge**	
	Gd1-O74	2.358	Gd1	1.220
	Gd1-O81	2.256	O74	-0.338
	Gd1-O80	2.314	O81	-0.300
	Gd1-O83	2.398	O80	-0.497
	Gd1-O77	2.323	O83	-0.370
	Gd1-O78	2.401	O77	-0.521
	Gd1-O76	2.305	O78	-0.371
	Gd1-O79	2.377	O76	-0.498
			O79	-0.389
			N84	-0.301
			N85	-0.225
			N86	-0.227
			N87	-0.301
			N88	-0.298
**Gd(nal)$_3$**			N89	-0.225
	**Bond angle**		**Torsion angle**	
	(°)		O74-Gd-O80-C61	147.25
	O74-Gd-O81	84.27	O74-Gd-O83-C51	-135.32
	O81-Gd-O80	64.73	O74-Gd-O78-C7	84.73
	O80-Gd-O83	63.45	O74-Gd-O77-C3	-129.86
	O83-Gd-O78	64.39	O74-Gd-O79-C24	76.41
	O78-Gd-O77	62.69	O74-Gd-O76-C20	-104.58
**Total energy (a.u.): -3311.35**	O77-Gd-O79	90.79	O74-Gd-O81-H91	-75.58
	O79-Gd-O76	63.57	Gd-O76-C20-O19	179.30
	O76-Gd-O74	78.61	Gd-O80-C61-O73	-178.69
	Gd-O80-C61	154.03	Gd-O77-C3-O2	174.74
	Gd-O79-C24	145.37	N87-C51-C6-C12	3.81
			N88-C24-N87-C51	-175.45
			N88-C24-N84-C7	179.78

	**Bond length (Å)**		**Mulliken atomic charge**	
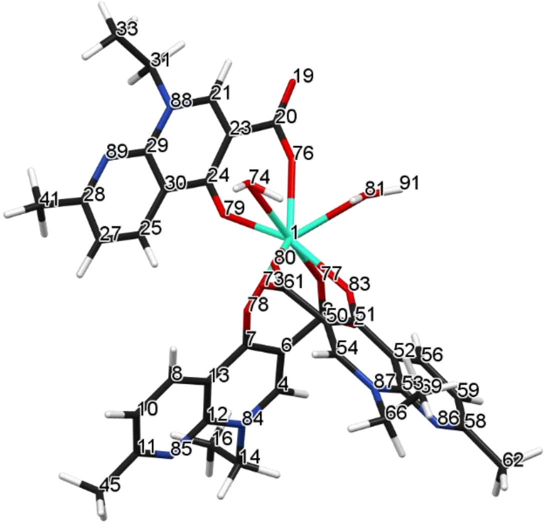	Tb1-O74	2.425	Tb1	1.116
	Tb1-O81	2.421	O74	-0.350
	Tb1-O80	2.296	O81	-0.261
	Tb1-O83	2.368	O80	-0.493
	Tb1-O77	2.279	O83	-0.377
	Tb1-O78	2.366	O77	-0.464
	Tb1-O76	2.300	O78	-0.385
	Tb1-O79	2.364	O76	-0.453
			O79	-0.383
			N84	-0.301
			N85	-0.226
			N86	-0.224
			N87	-0.300
			N88	-0.299
**Tb(nal)$_3$**			N89	-0.225
	**Bond angle (°)**		**Torsion angle**	
			O74-Tb-O80-C61	-147.22
	O74-Tb-O81	89.90	O74-Tb-O83-C51	53.72
	O81-Tb-O80	99.70	O74-Tb-O78-C7	164.65
	O80-Tb-O83	64.04	O74-Tb-O77-C3	-108.00
	O83-Tb-O78	86.84	O74-Tb-O79-C24	80.06
**Total energy (a.u.): -3369.41**	O78-Tb-O77	65.24	O74-Tb-O76-C20	-92.29
	O77-Tb-O79	88.89	O74-Tb-O81-H91	158.09
	O79-Tb-O76	64.71	Tb-O76-C20-O19	178.23
	O76-Tb-O74	66.91	Tb-O80-C61-O73	171.76
	Tb-O80-C61	153.53	Tb-O77-C3-O2	-168.07
	Tb-O79-C24	144.10	N87-C51-C6-C12	36.88
			N88-C24-N87-C51	-47.12
			N88-C24-N84-C7	-176.51

Table S7. IR selected data (experimental vs. predicted) for the optimized structures of $Eu(nal)_2$ and $Eu(nal)_3$.

Assignment	$Eu(nal)_2$		$Eu(nal)_3$	
	Experimental	Predicted	Experimental	Predicted
ν(O–H); COOH, H_2O	3395 wb	2871 w<br>2739 w	3399 wb	2850 w<br>2815 w<br>2724 m
ν (CH_3-CH_2)	2981 w<br>2931 w	2780 m<br>2779 m	2971 w<br>2921 w	2780 m<br>2775 m
ν as (COO^-)	1615 s	1888 s<br>1871 s	1615 s	1882 s<br>1862 s
Pyridonic ν(C=O)	1567 s	1757 s	1558 s	1768 s<br>1742 s
Pyridonic ν (C-N)	1523 s	1590 m	1523 s	1588 m
ν s (COO^-)	1442 s	1388 s<br>1365 w	1441 s	1381 s<br>1372 s
δ (COO^-)	781 w<br>756 m<br>703 w	850 w<br>758 w	782 w<br>757 m<br>703 w	759 w<br>745 w<br>721 w
Ring deformation	657 m<br>635 w<br>541 m<br>469 m	Numerous peaks of weak intensity in the 800-400 cm^{-1} range.	657 m<br>635 w<br>541 m	Numerous peaks of weak intensity in the 800-400 cm^{-1} range.
ν (M–O)	559 m<br>491 s	633 m<br>596 w<br>562 w<br>276 w	463 m<br>433 m	627 w<br>450 w<br>276 w

w = weak, m = medium, s = sharp, b = broad

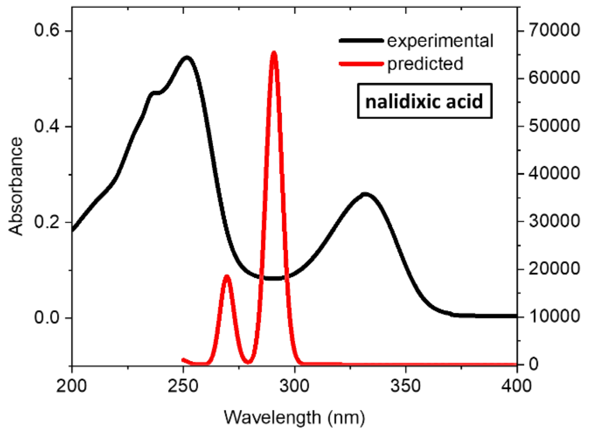

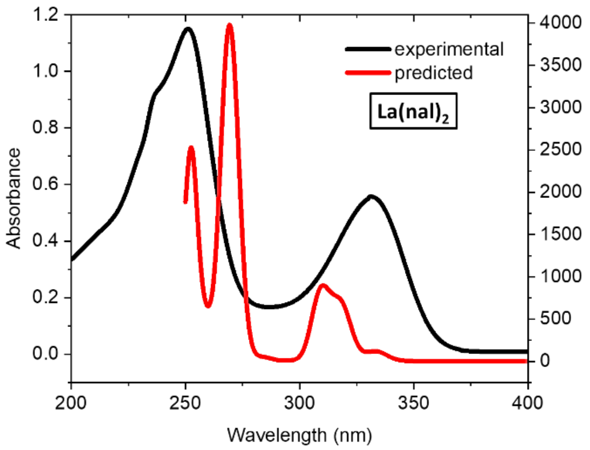

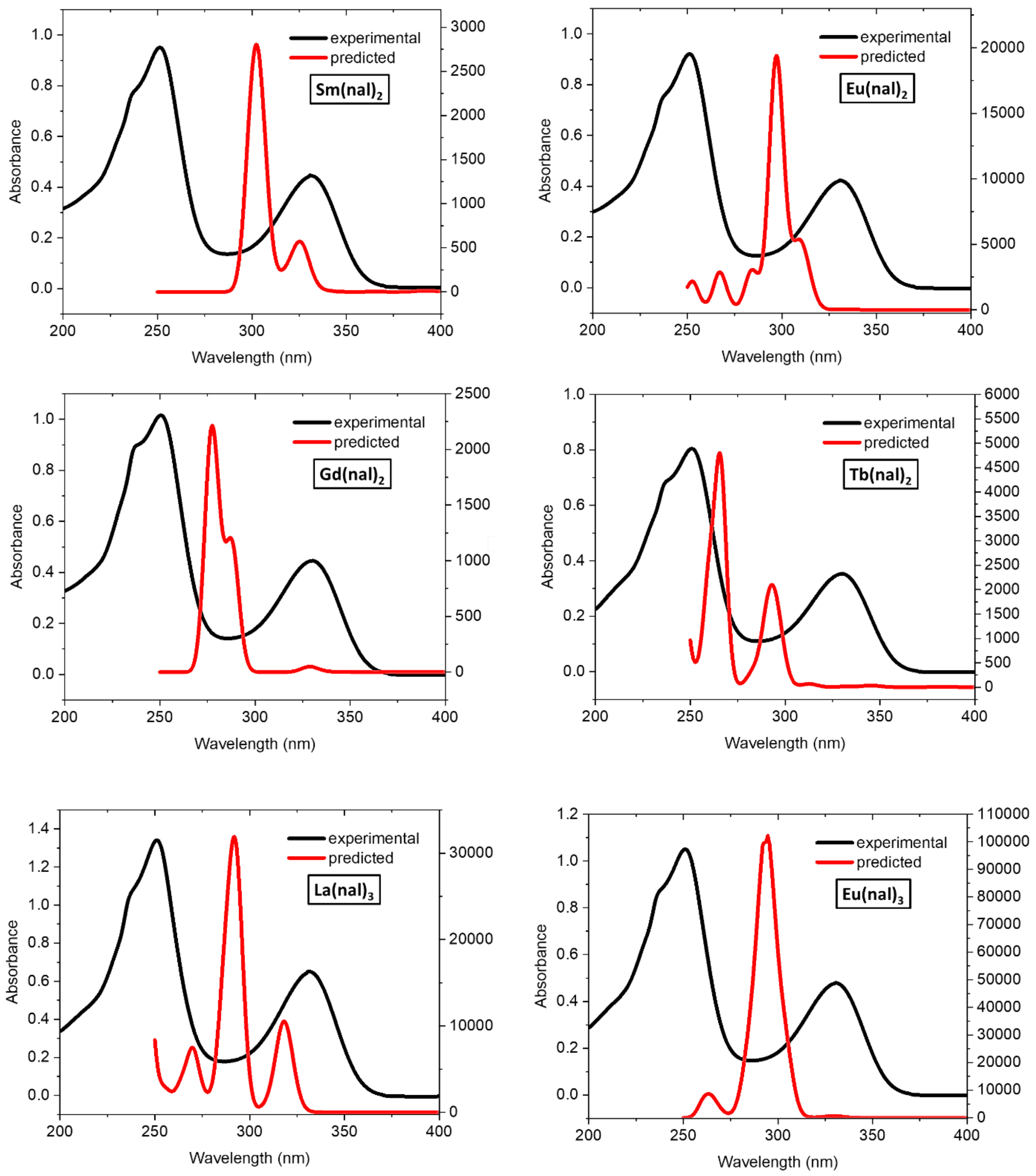

Sm(nal)2
Eu(nal)2
Gd(nal)2
Tb(nal)2
La(nal)3
Eu(nal)3
experimental
predicted
Absorbance
Wavelength (nm)

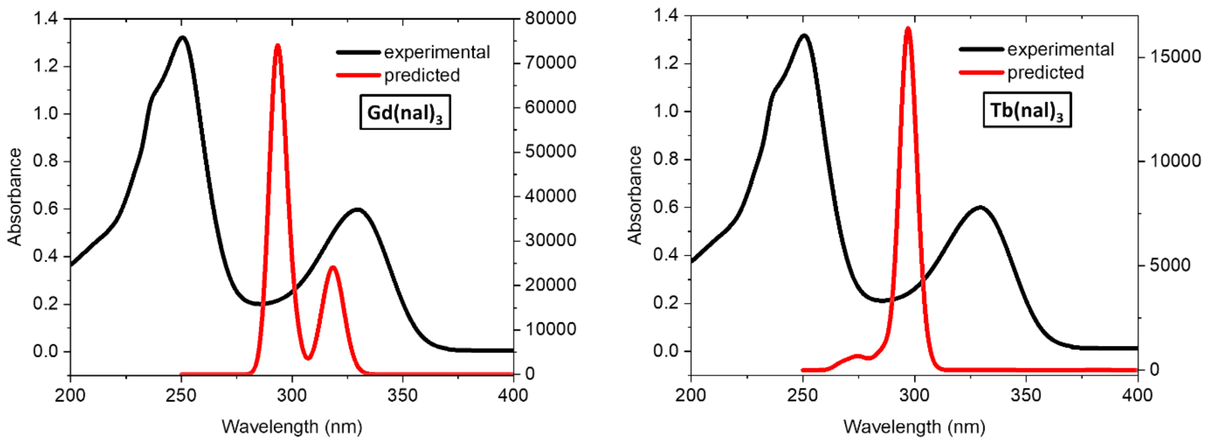

Figure S7. Experimental and predicted electronic spectra of nalidixic acid and its metal complexes.

Table S8. UV-Vis spectral data for nalidixic acid and complexes (experimental vs. predicted).

Compound	Band I (nm)		Band II (nm)	
	Experimental	Predicted	Experimental	Predicted
Nalidixic acid	253 (shoulder at 238)	270	335	291
La(nal)2	252 (shoulder at 236)	270<br>253	332	311 (shoulder at 318)
Sm(nal)2	253 (shoulder at 238)	302	334	326
Eu(nal)2	253 (shoulder at 237)	297 (shoulder at 284)<br>268<br>253	333	310
Gd(nal)2	251 (shoulder at 236)	292	333	303<br>351
Tb(nal)2	251 (shoulder at 236)	265	332	294
La(nal)3	251 (shoulder at 237)	292<br>270	333	319
Eu(nal)3	252 (shoulder at 237)	295<br>264	334	328
Gd(nal)3	252 (shoulder at 237)	294	331	320
Tb(nal)3	252 (shoulder at 236)	273	333	297

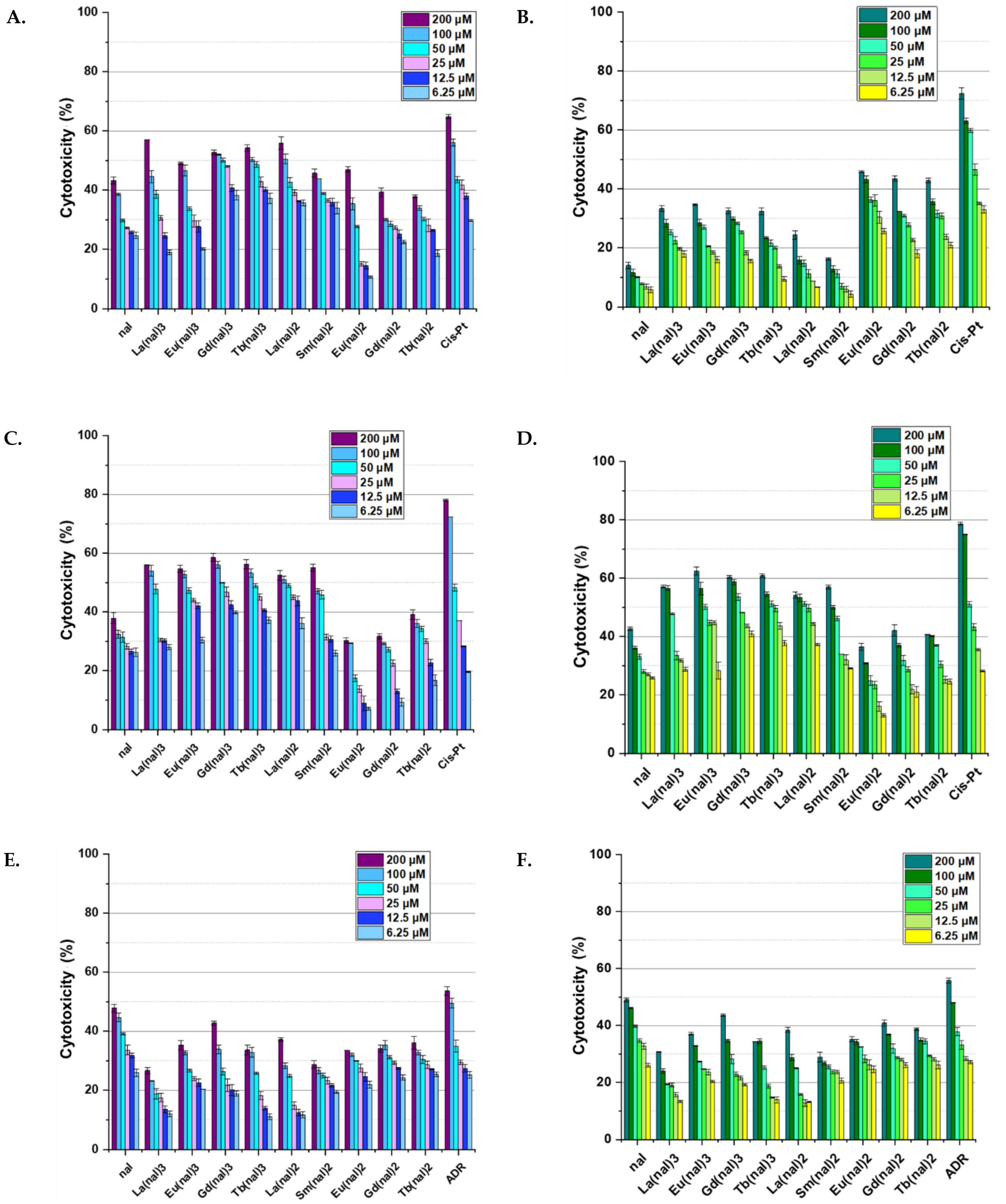

Figure S8. Citotoxicity of **M(nal)₂** (M= La^{3+}, Sm^{3+}, Eu^{3+}, Gd^{3+}, Tb^{3+}) and **M(nal)₃** (M= La^{3+}, Eu^{3+}, Gd^{3+}, Tb^{3+}) complexes tested on the following cell lines: **A.** HUVEC - 24h, **B.** HUVEC- 48h, **C**. LoVo – 24h, **D**. LoVo-48h, **E.** MDA-MB 231- 24h and **F**. MDA-MB 231- 48h.

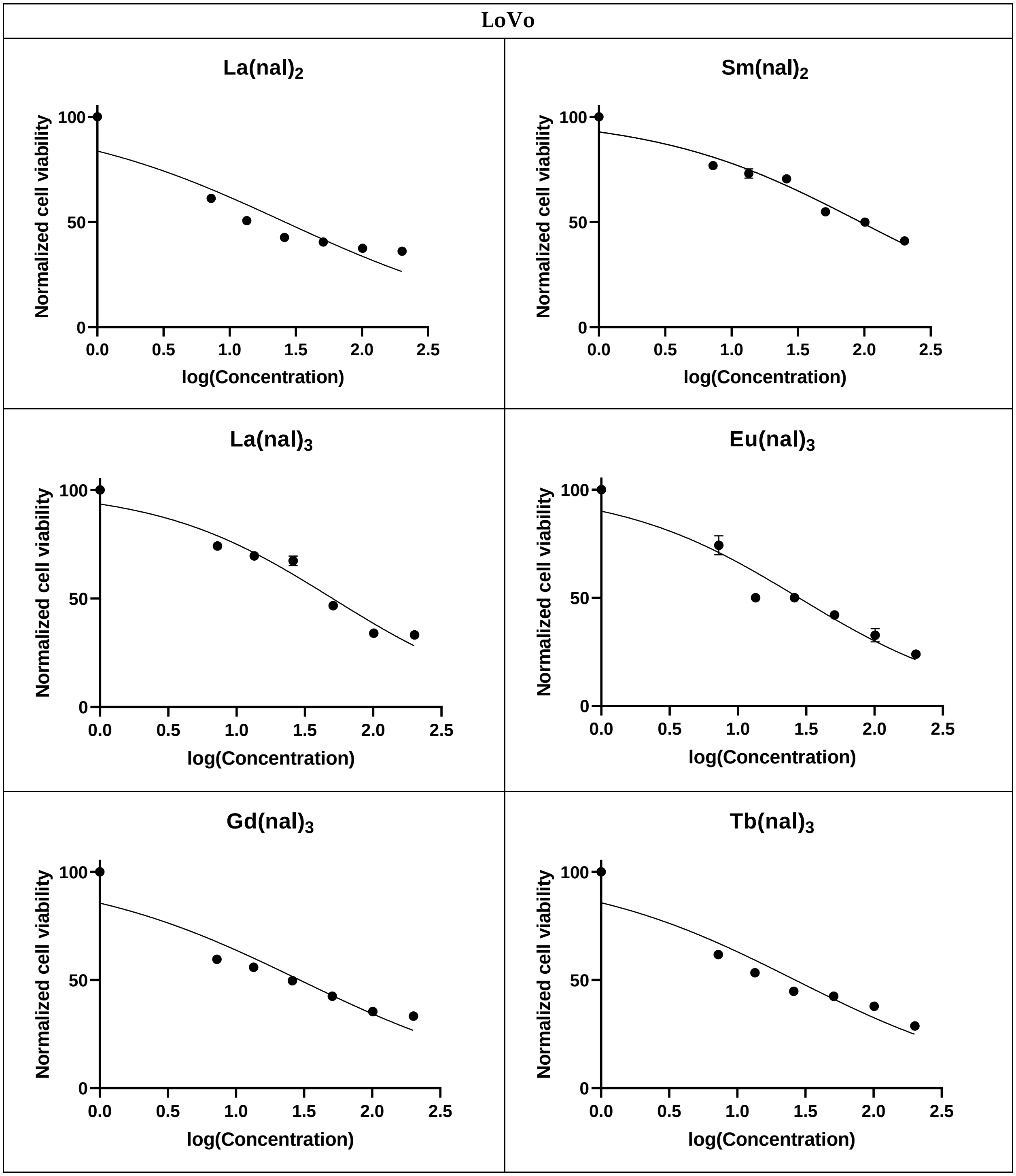
LoVo
La(nal)2
Sm(nal)2
La(nal)3
Eu(nal)3
Gd(nal)3
Tb(nal)3
Normalized cell viability
log(Concentration)
0
50
100
0.0
0.5
1.0
1.5
2.0
2.5

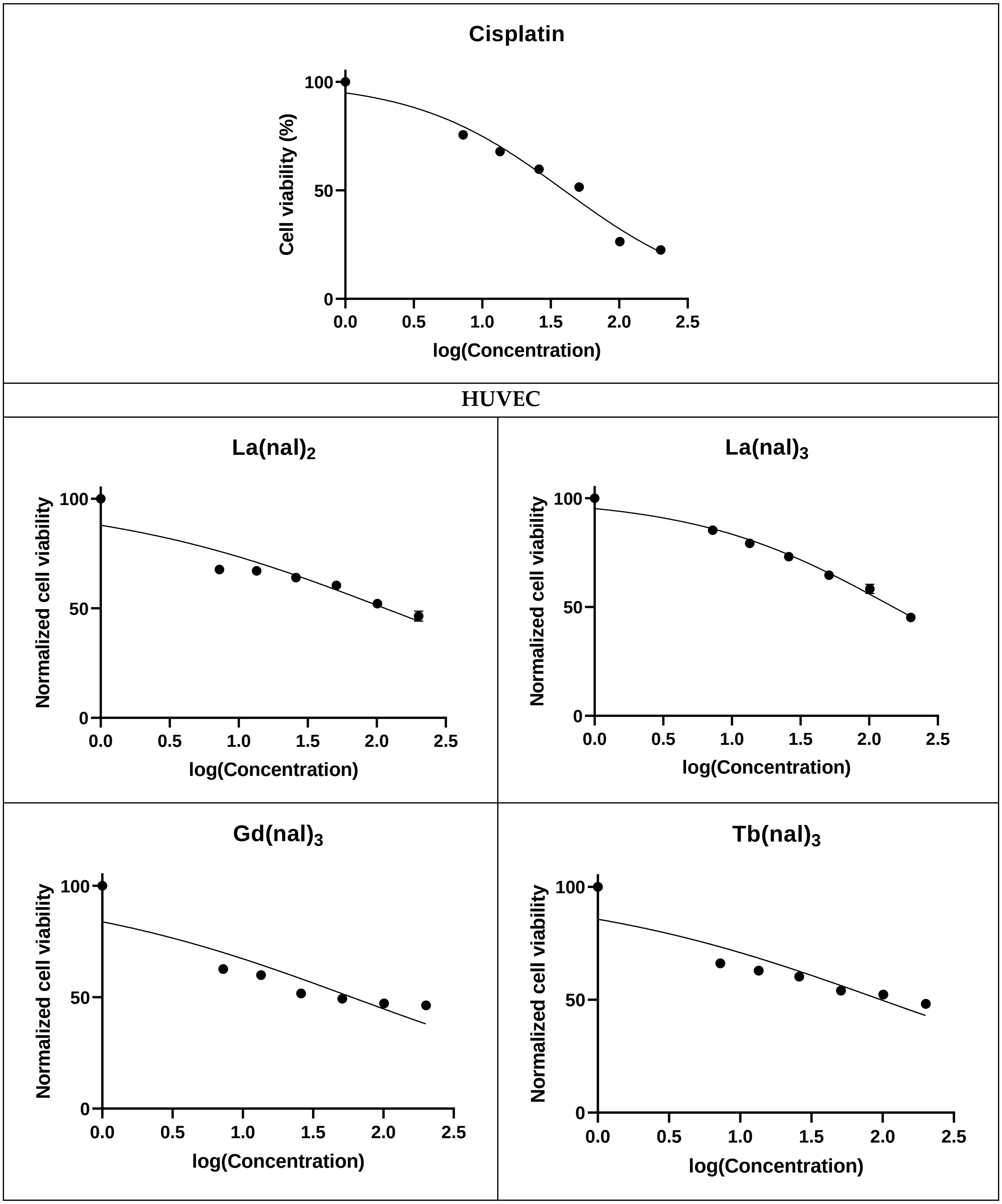

Cisplatin
Cell viability (%)
log(Concentration)
HUVEC
La(nal)2
La(nal)3
Gd(nal)3
Tb(nal)3
Normalized cell viability
log(Concentration)

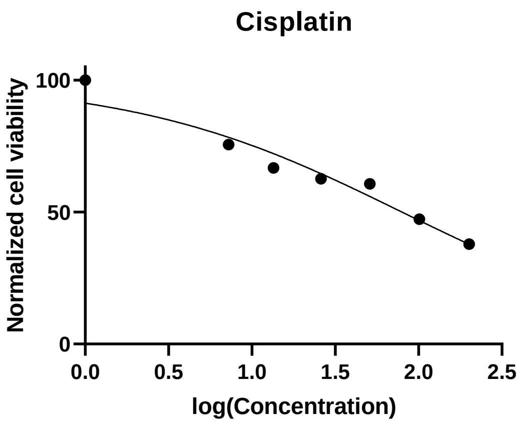

Figure S9. Graphical representations of normalized cell viability *vs.* log(Concentration). Concentrations were expressed in μM and transformed to the corresponding logarithmic values. Data were normalized against the smallest value of cell viability in this data set, corresponding to cisplatin. Results were plotted in GraphPad Prism 8.0.1 using the built-in equation Nonlinear regression (curve fit) – log(inhibitor) *vs.* normalized response variable slope.

$La(nal)_2$	$Sm(nal)_2$

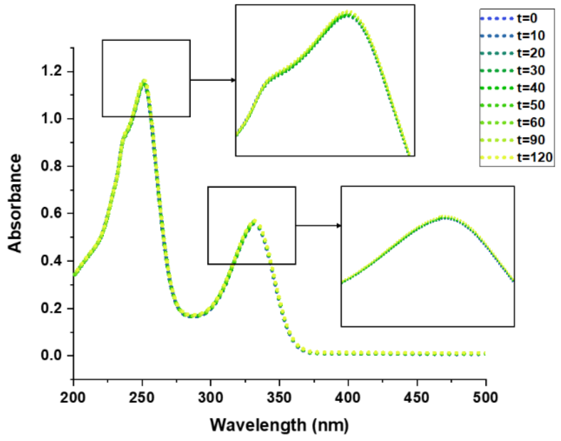

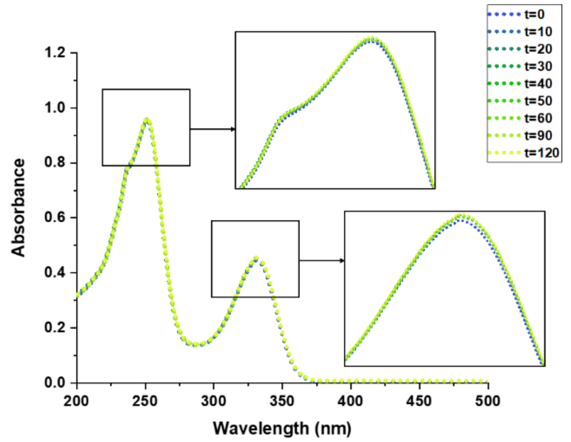

$Eu(nal)_2$	$Gd(nal)_2$

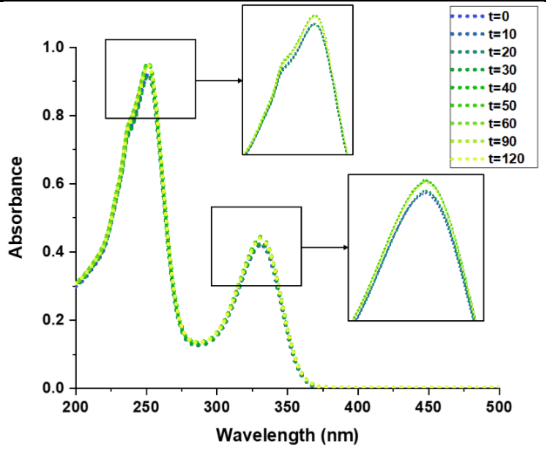

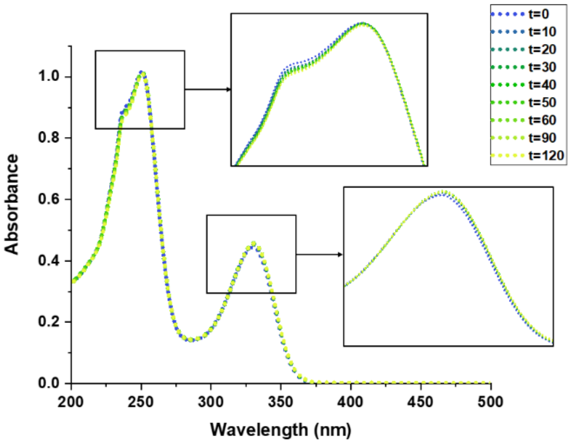

$Tb(nal)_2$	$La(nal)_3$

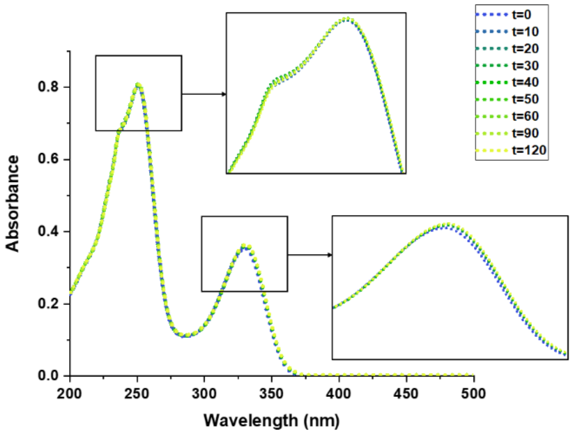

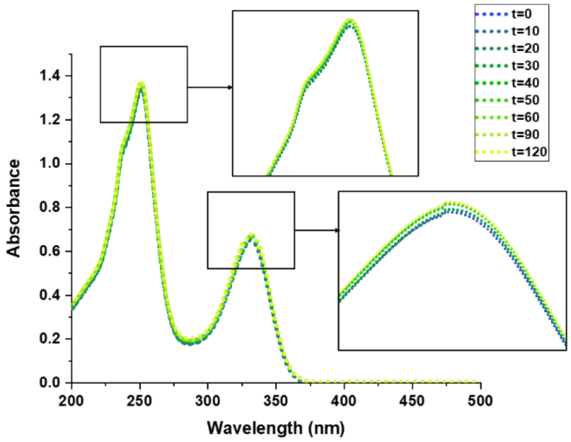

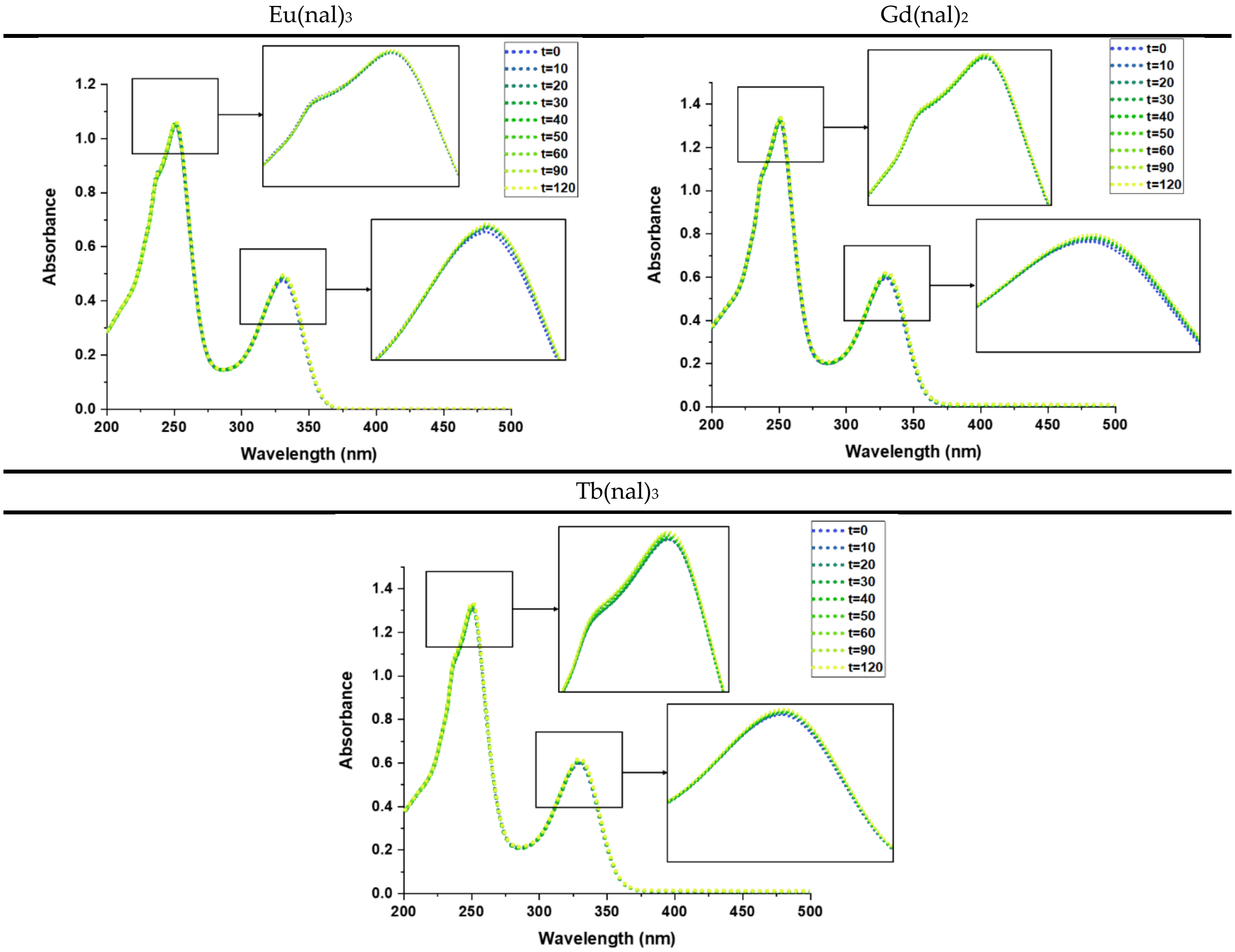

Figure S10. Stability assay: UV-vis spectra of the complexes in DMSO-TrisHCl buffer mixture (5 mM Tris-HCl/ 50 mM NaCl, pH 7.4).

Table S9. Absorbances of tested compounds in UV-Vis, in DMSO-TrisHCl buffer, [compound]= 20μM.

t (min)	λ (nm)	0	10	20	30	60	90	120
	**251**	1.150	1.147	1.147	1.149	1.152	1.155	1.158
**La(nal)2**	**331**	0.557	0.557	0.558	0.560	0.561	0.565	0.566
	**251.5**	0.951	0.957	0.959	0.958	0.959	1.100	0.956
**Sm(nal)2**	**331**	0.446	0.453	0.455	0.455	0.456	0.482	0.452
	**251**	0.921	0.920	0.920	0.919	0.954	0.954	0.952
**Eu(nal)2**	**330.5**	0.423	0.425	0.426	0.427	0.444	0.445	0.443
	**251.5**	1.015	1.013	1.014	1.013	1.013	1.015	1.012
**Gd(nal)2**	**330.5**	0.446	0.452	0.454	0.456	0.457	0.459	0.458
	**251**	0.804	0.804	0.805	0.807	0.807	0.809	0.809
**Tb(nal)2**	**330.5**	0.354	0.360	0.362	0.362	0.364	0.366	0.366
	**251**	1.341	1.338	1.336	1.339	1.358	1.363	1.369
**La(nal)3**	**331.5**	0.651	0.657	0.657	0.659	0.671	0.673	0.676
	**251**	1.050	1.049	1.052	1.053	1.055	1.055	1.057
**Eu(nal)3**	**331**	0.479	0.479	0.479	0.479	0.479	0.479	0.479
	**250.5**	1.321	1.318	1.320	1.323	1.325	1.331	1.335
**Gd(nal)3**	**330.5**	0.5974	0.605	0.609	0.612	0.614	0.622	0.623
	**250.5**	1.317	1.314	1.313	1.318	1.321	1.327	1.331
**Tb(nal)3**	**330.5**	0.598	0.605	0.605	0.609	0.610	0.614	0.617

Nalidixic acid	$La(nal)_2$

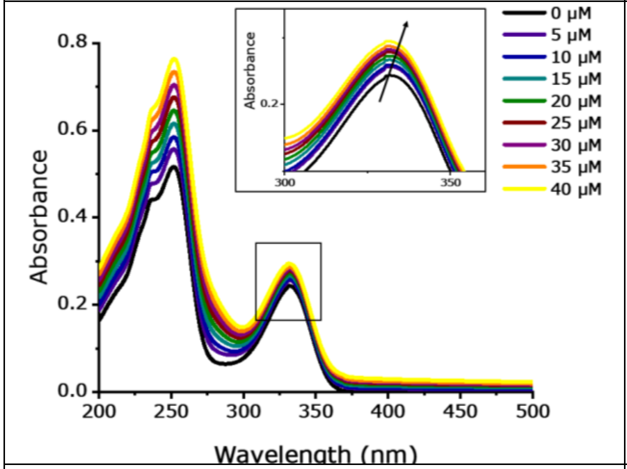

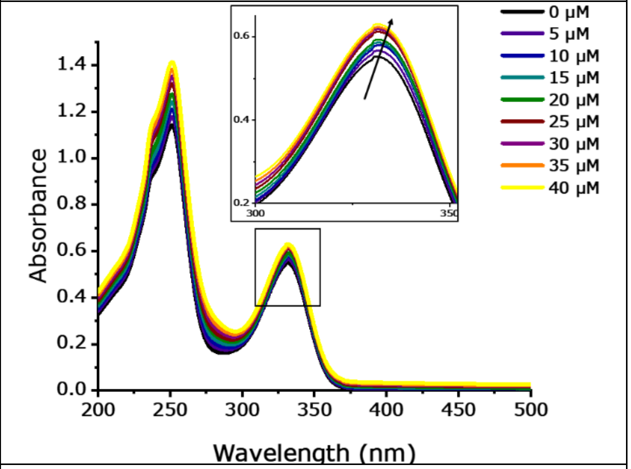

$Sm(nal)_2$	$Eu(nal)_2$

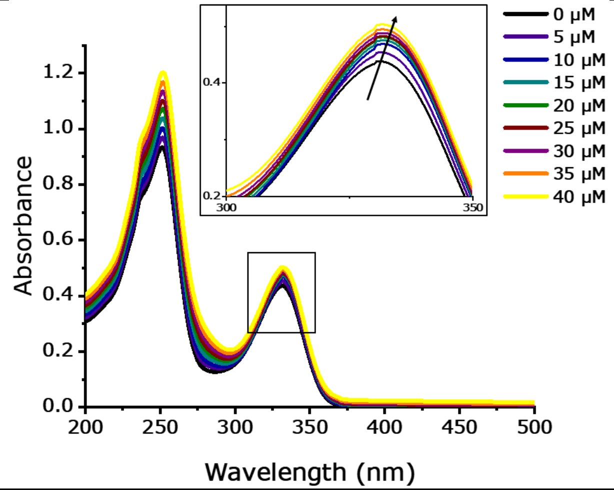

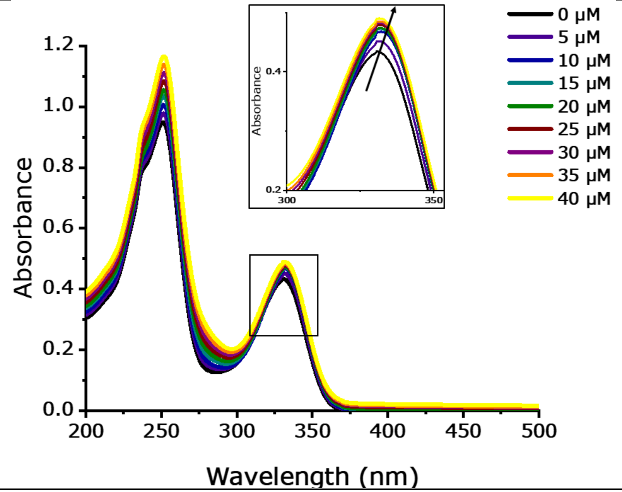

$Gd(nal)_2$	$Tb(nal)_2$

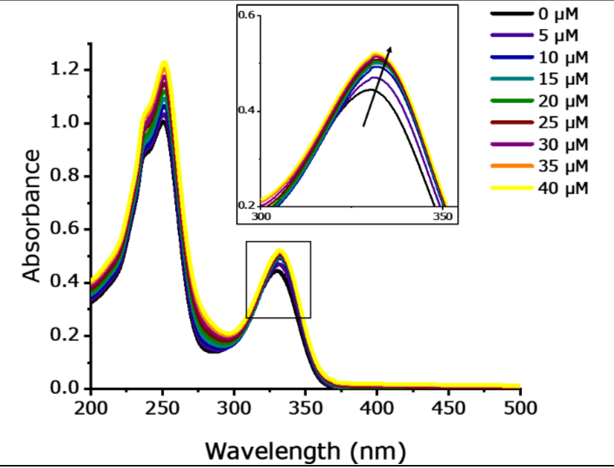

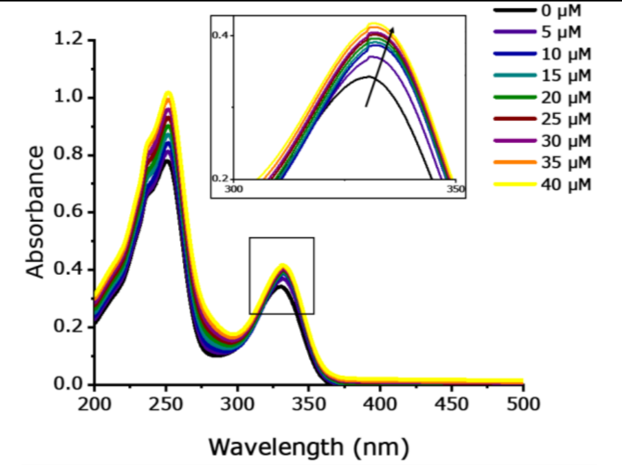

$La(nal)_3$	$Eu(nal)_3$

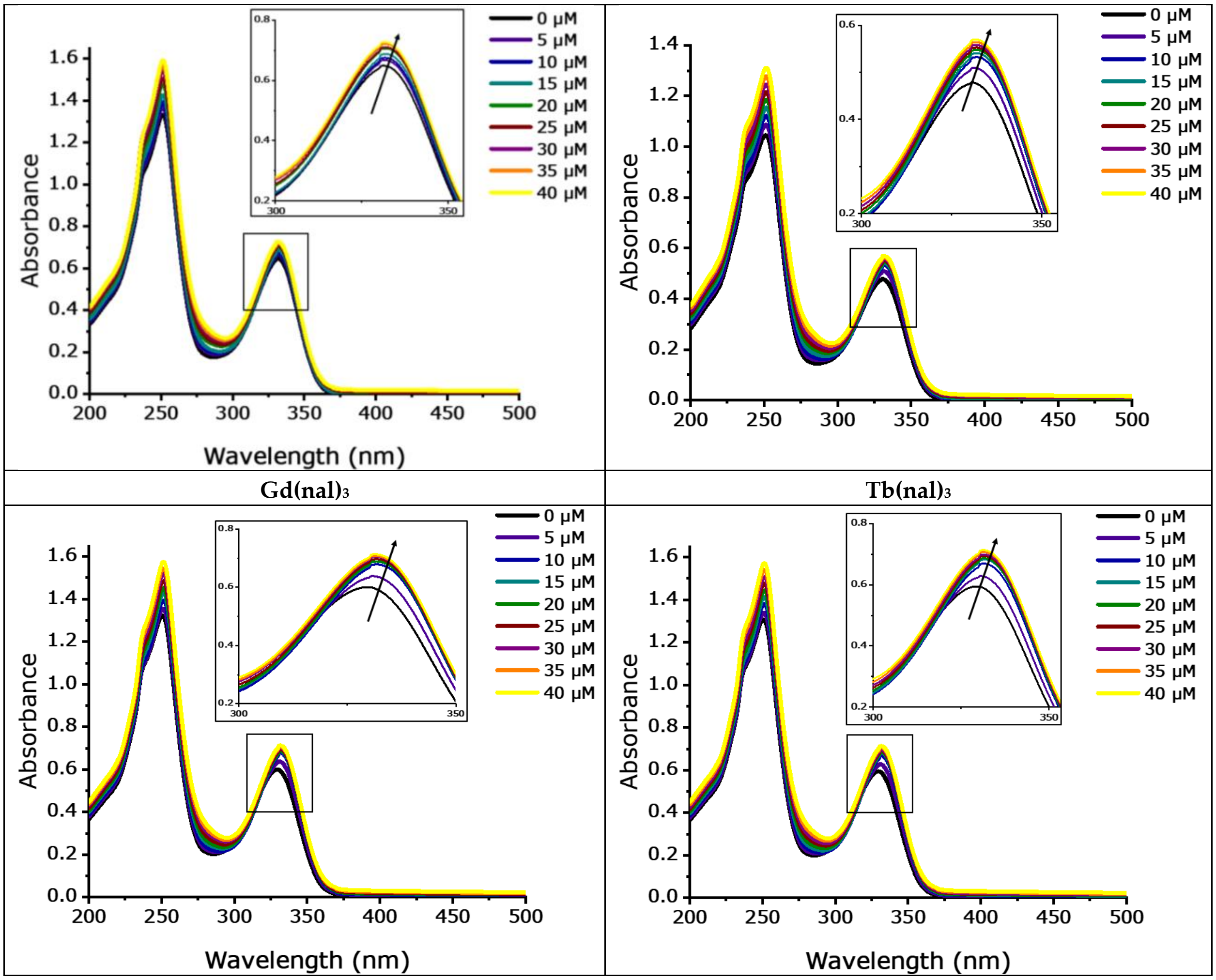

Figure S11. Absorption spectra of the tested compounds in the absence and presence of increasing amounts of DNA. [compound] = 20 μM; [DNA] = 0, 5, 10, 15, 20, 25, 30, 35, 40 μM. The arrows show the absorption changes upon increasing the DNA concentration.

Nalidixic acid

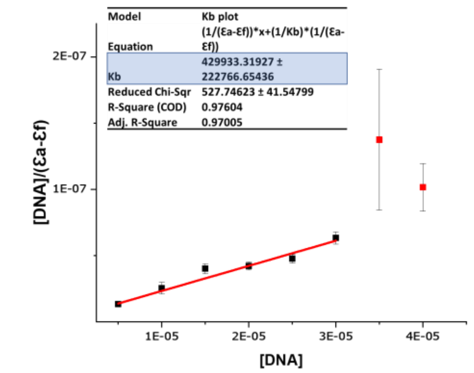

La(nal)2

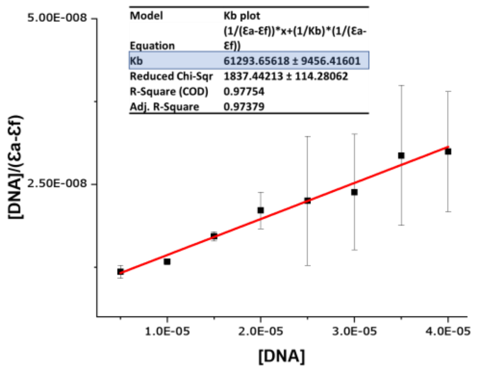

Sm(nal)2

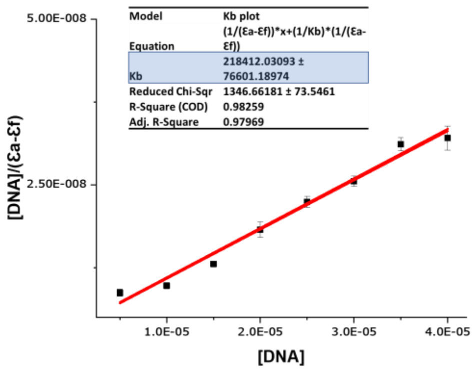

Eu(nal)2

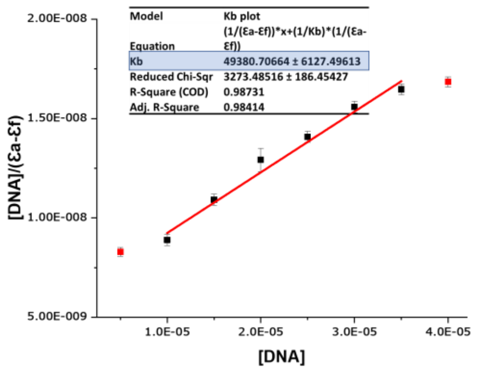

Gd(nal)2

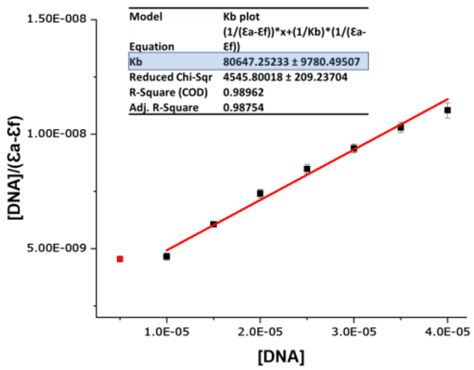

Tb(nal)2

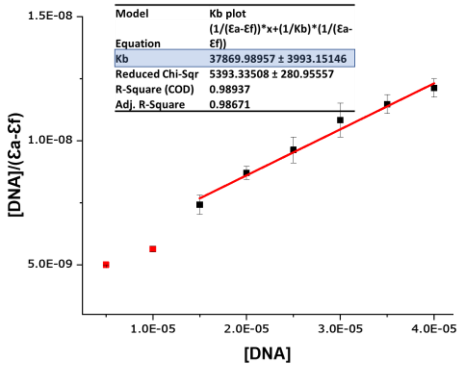

La(nal)$_3$	Eu(nal)$_3$
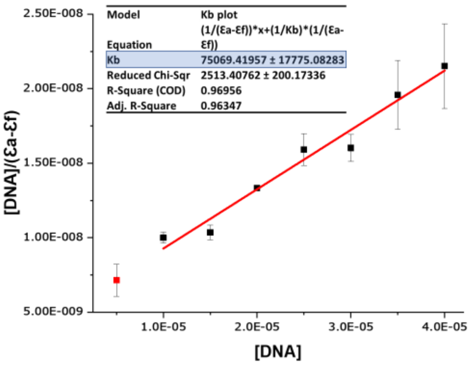	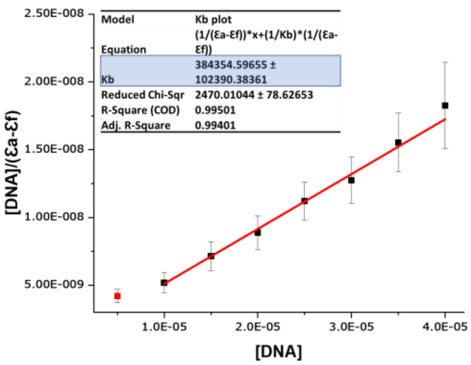
**Gd(nal)$_3$**	**Tb(nal)$_3$**
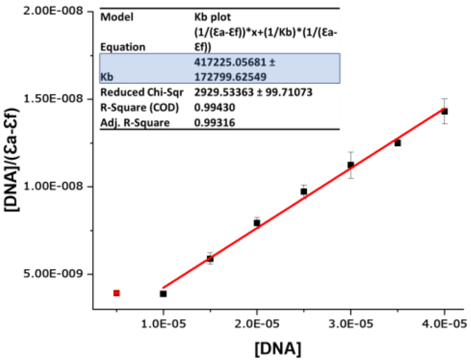	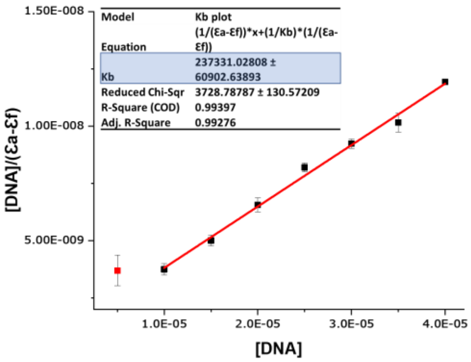

Figure S12. Graphical representations of the DNA-binding constants (K_b) for systems containing [compound] = 20 μM; [DNA] = 5, 10, 15, 20, 25, 30, 35, 40 μM. K_b values were calculated using Equation 2 in OriginPro® 2018. Outliers are represented on the graphs as red data points.

Nalidixic acid	$La(nal)_2$
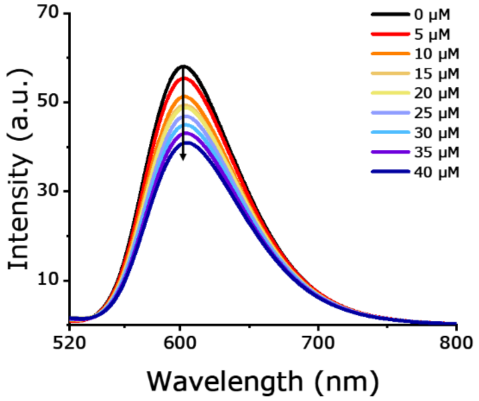	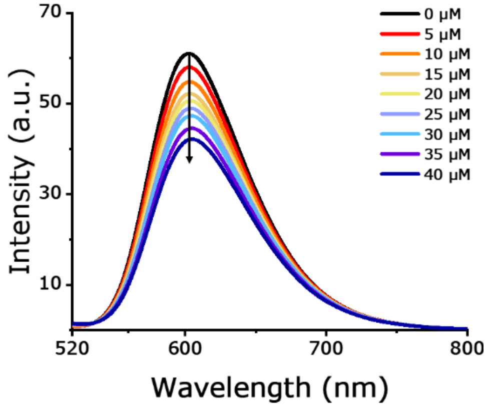

$Sm(nal)_2$	$Eu(nal)_2$
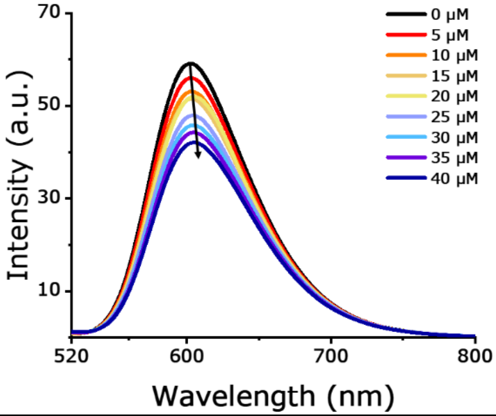	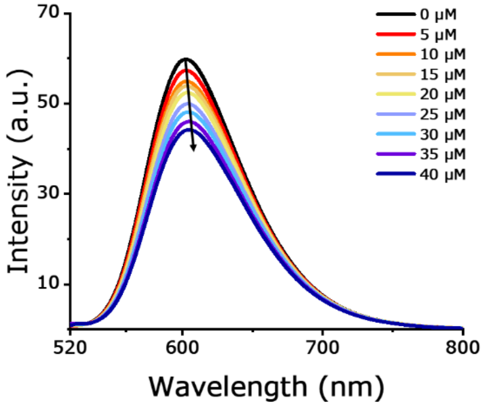

$Gd(nal)_2$	$Tb(nal)_2$
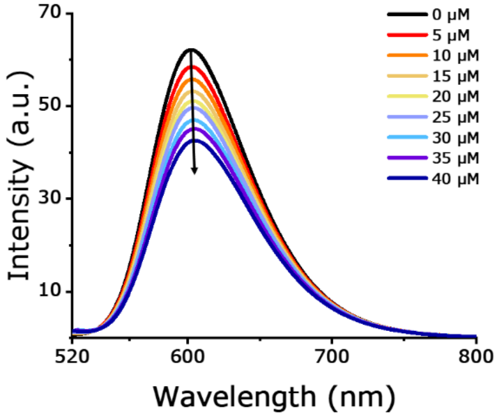	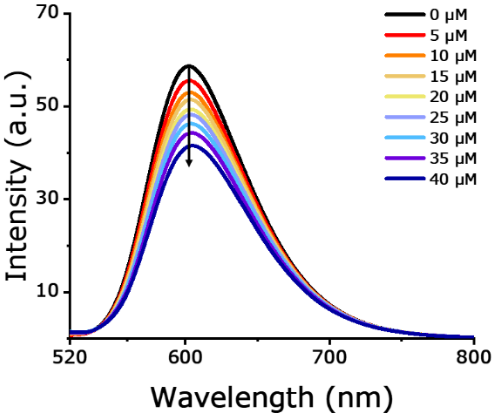

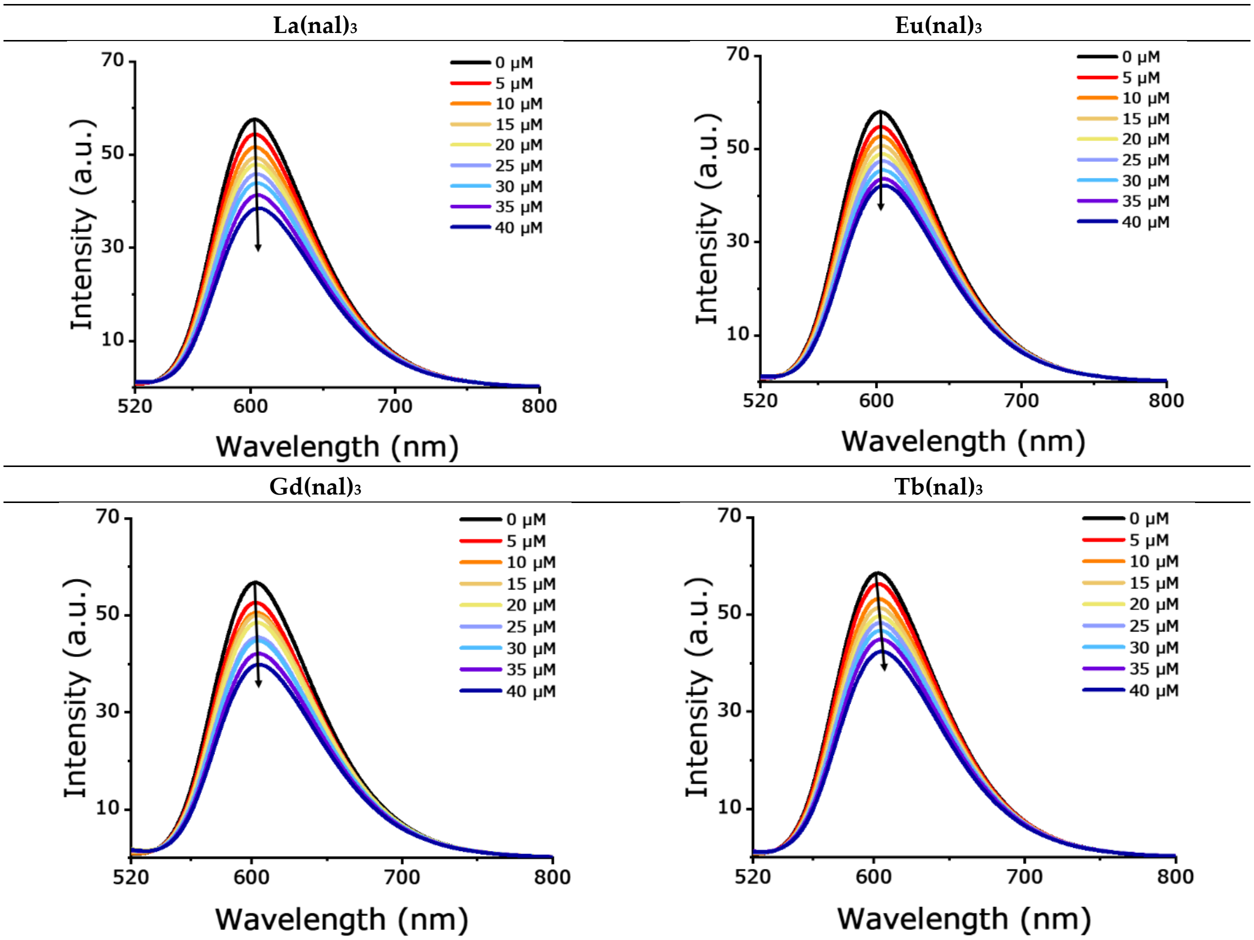

Figure S13. Fluorescence spectra of the EB - DNA system in the absence and presence of increasing amounts of the tested compounds. λ_{ex} = 500 nm, [EB] = 2 µM, [DNA] = 10 µM, [compound] = 0, 5, 10, 15, 20, 25, 30, 35, 40 µM. Arrows indicate the changes in fluorescence intensities upon increasing the concentrations of the tested compounds.

Nalidixic acid

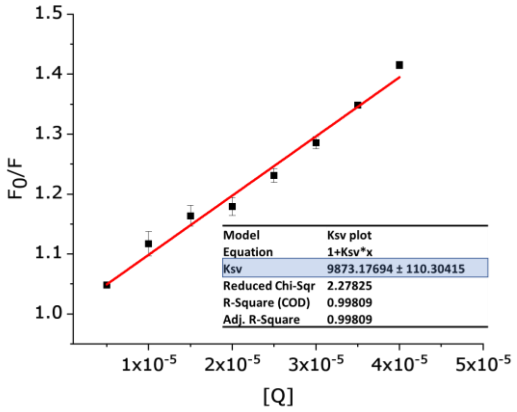

La(nal)2

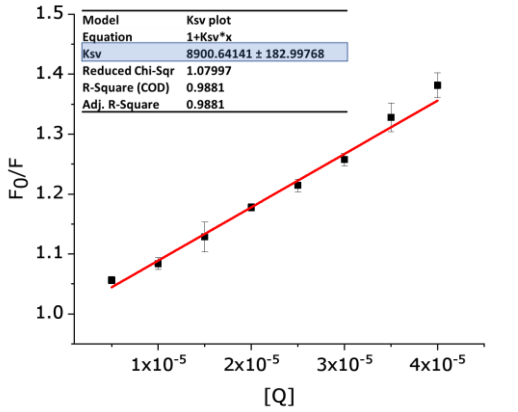

Sm(nal)2

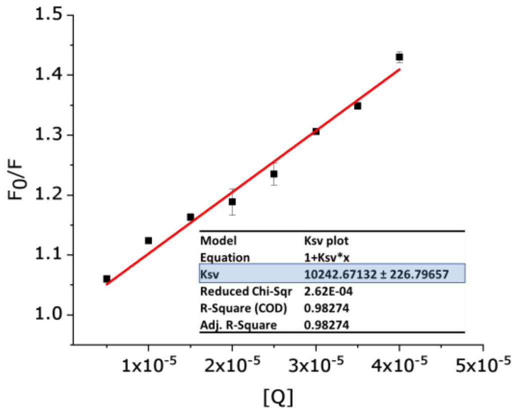

Eu(nal)2

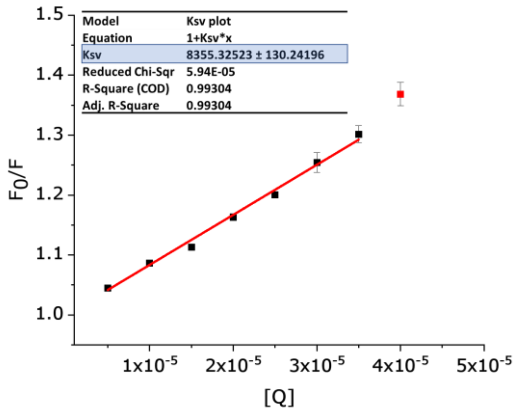

Gd(nal)2

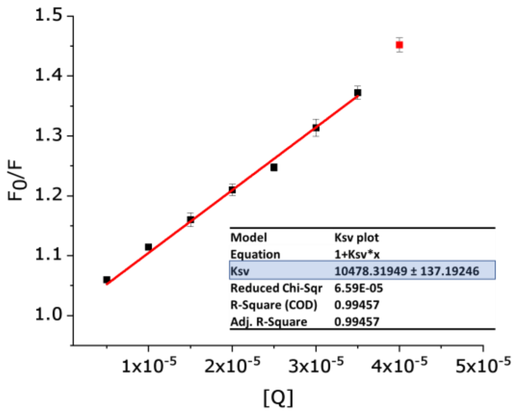

Tb(nal)2

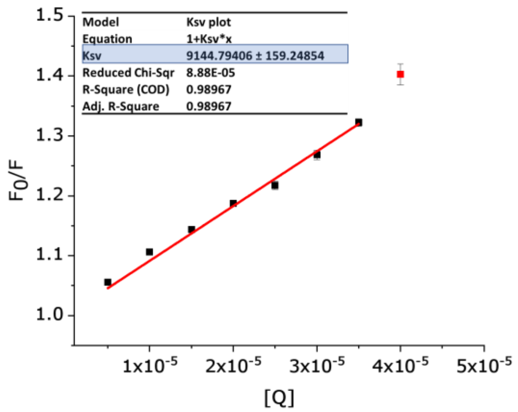

La(nal)$_3$

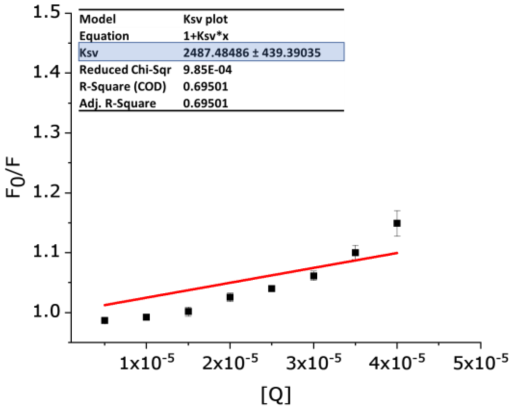

Eu(nal)$_3$

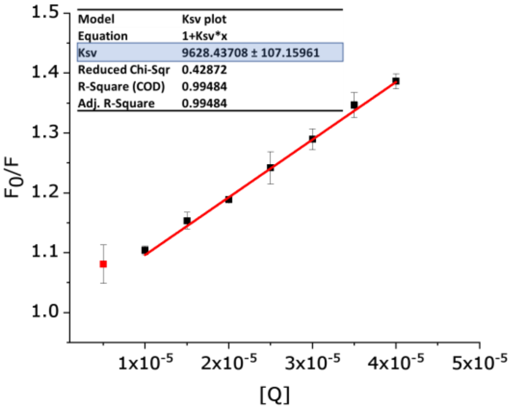

Gd(nal)$_3$

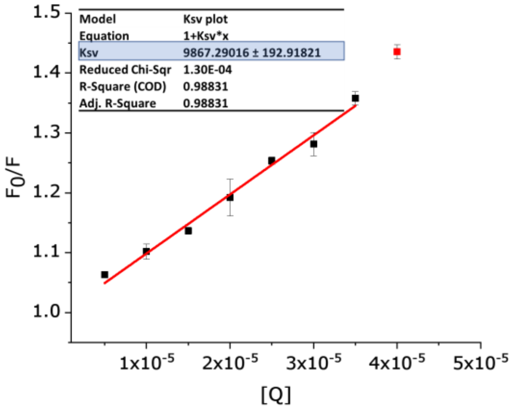

Tb(nal)$_3$

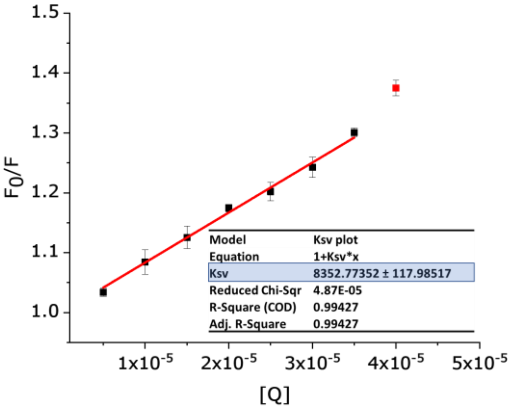

Figure S14. Graphical representations of the K_{SV} constants for systems containing [EB] = 2 μM, [DNA] = 10 μM, [compound] = 5, 10, 15, 20, 25, 30, 35, 40 μM. K_{SV} values were calculated using Equation 3 in OriginPro® 2018. Outliers are represented on the graphs as red data points.

Nalidixic acid

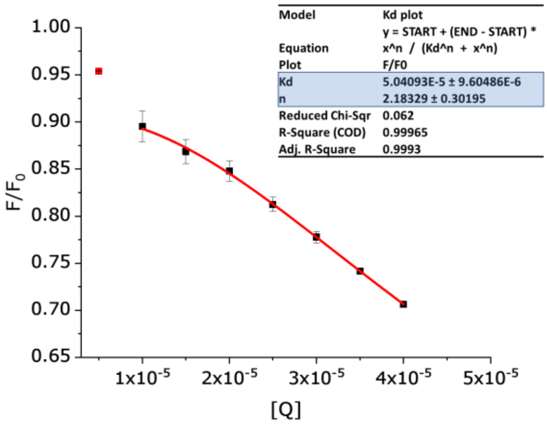

La(nal)$_2$

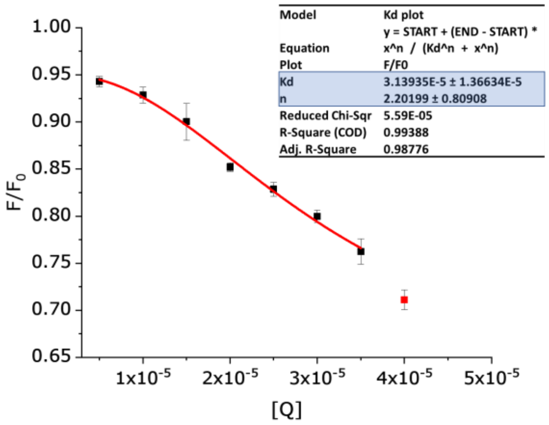

Sm(nal)$_2$

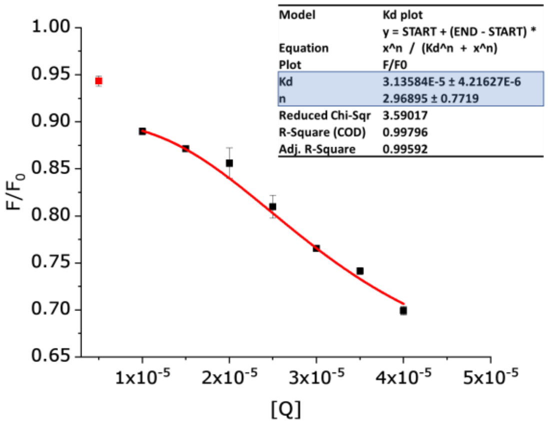

Eu(nal)$_2$

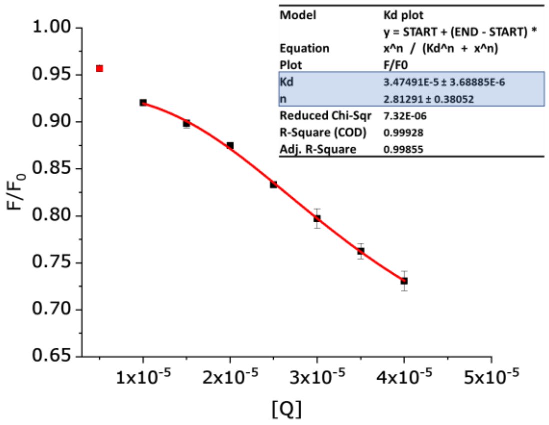

Gd(nal)$_2$

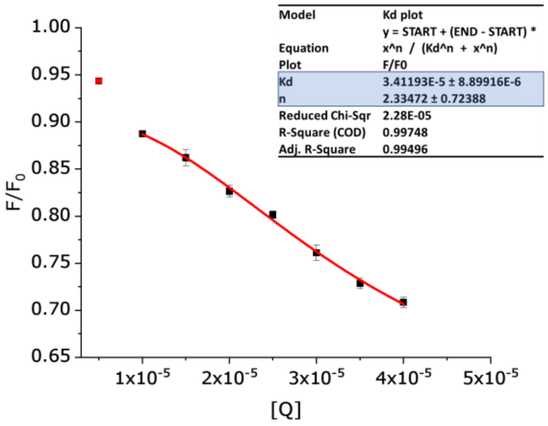

Tb(nal)$_2$

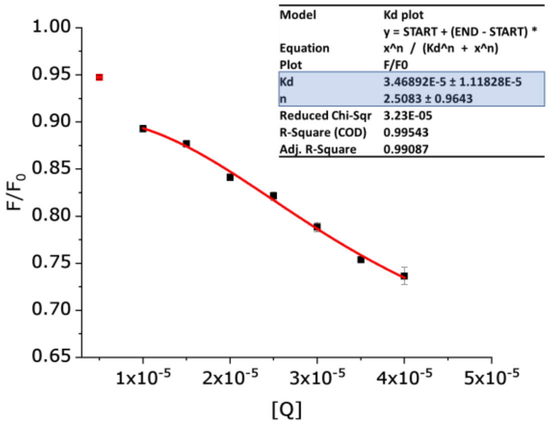

La(nal)$_3$ | **Eu(nal)$_3$**

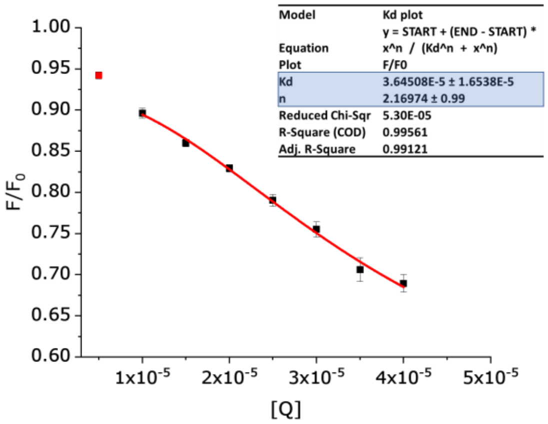

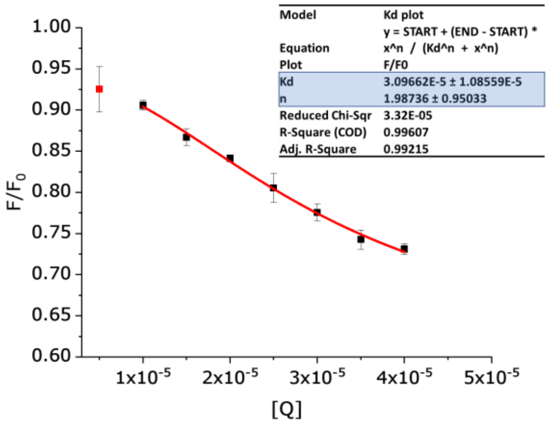

Gd(nal)$_3$ | **Tb(nal)$_3$**

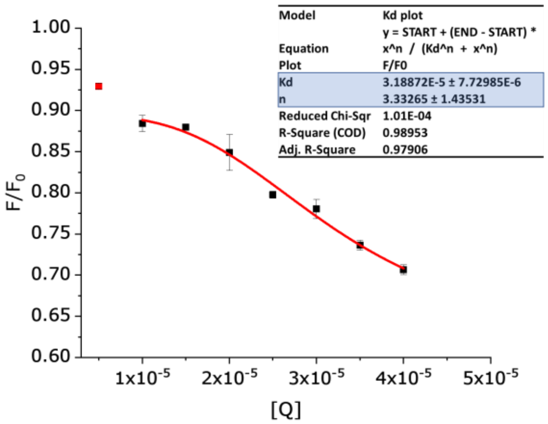

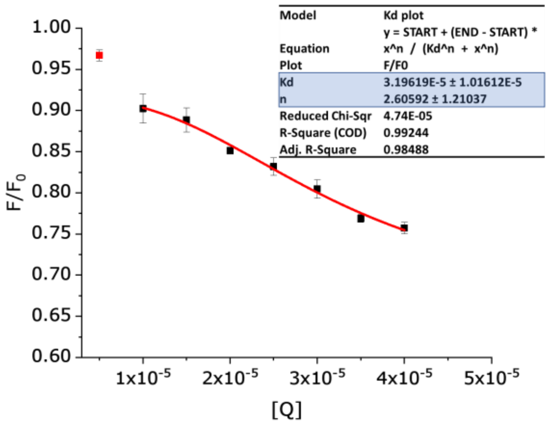

Figure S15. Graphical representation of the K_{50} constants for systems containing [EB] = 2 μM, [DNA] = 10 μM, [compound] = 5, 10, 15, 20, 25, 30, 35, 40 μM. K_{50} values were calculated using Equation 3 in OriginPro® 2018. Outliers are represented on the graphs as red data points.

Nalidixic acid

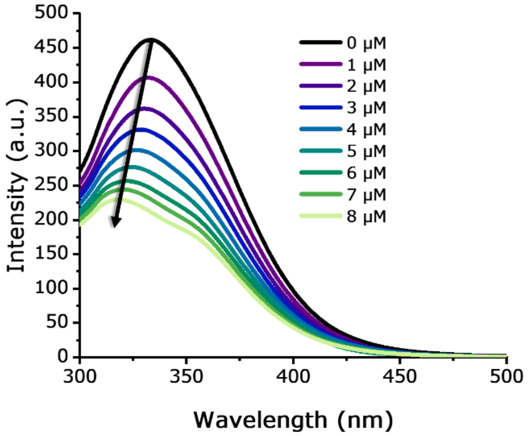

La(nal)$_2$

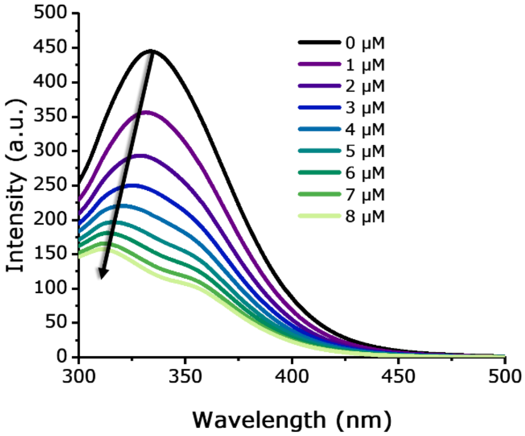

Sm(nal)$_2$

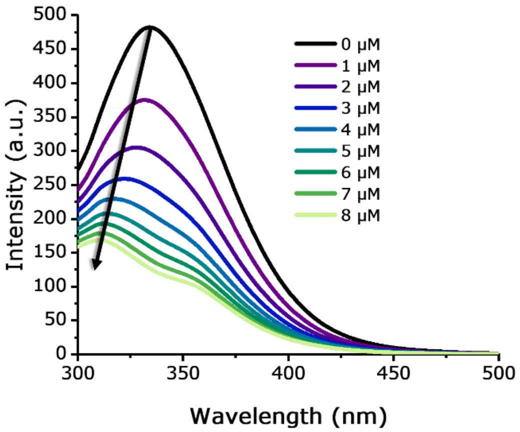

Eu(nal)$_2$

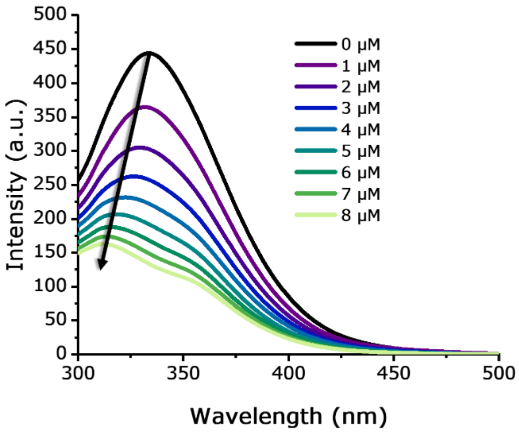

Gd(nal)$_2$

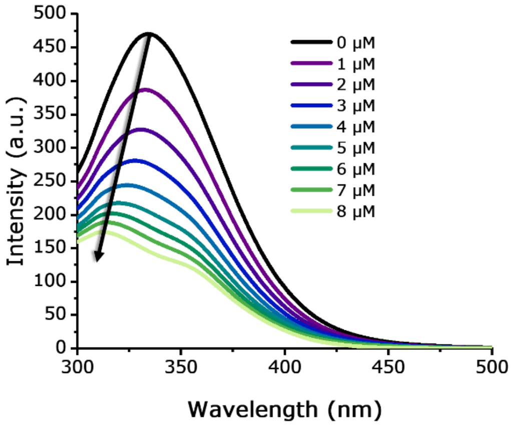

Tb(nal)$_2$

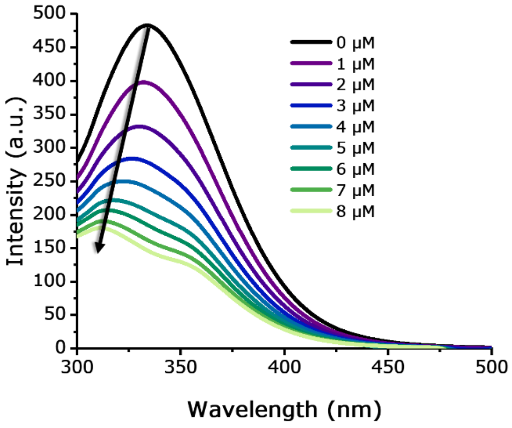

La(nal)$_3$	Eu(nal)$_3$
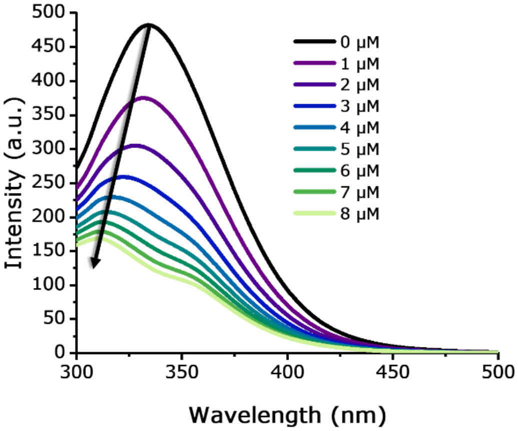	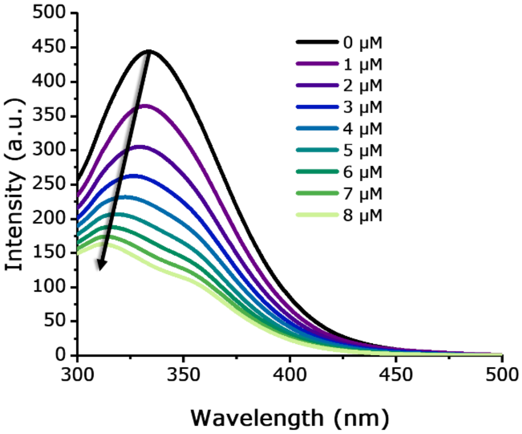
**Gd(nal)$_3$**	**Tb(nal)$_3$**
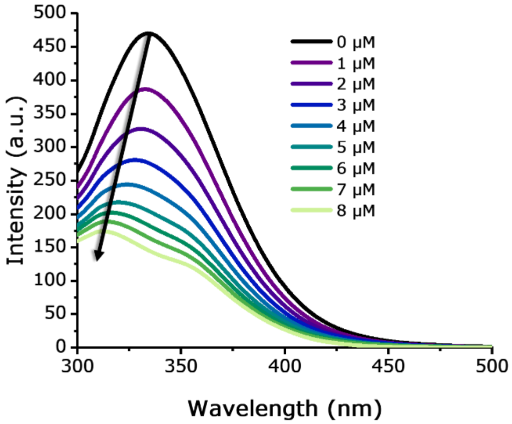	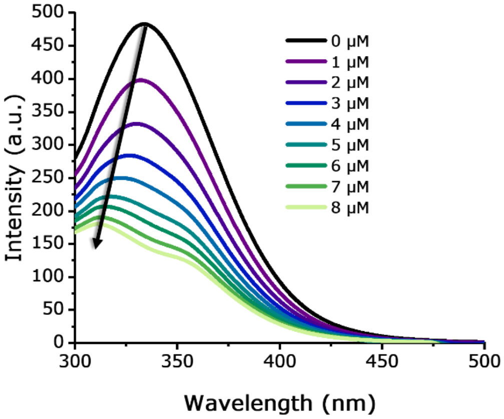

Figure S16. Changes in fluorescence intensity of free HSA *vs.* compound-HSA systems; the black arrows indicate a decrease of the intensity upon the addition of increasing amounts of compound; [HSA] = 2.5 μM, [compound] = 0, 1, 2, 3, 4, 5, 6, 7, 8 μM.

Nalidixic acid

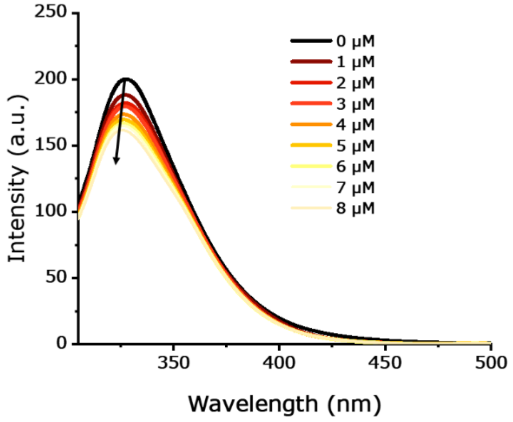

La(nal)$_2$

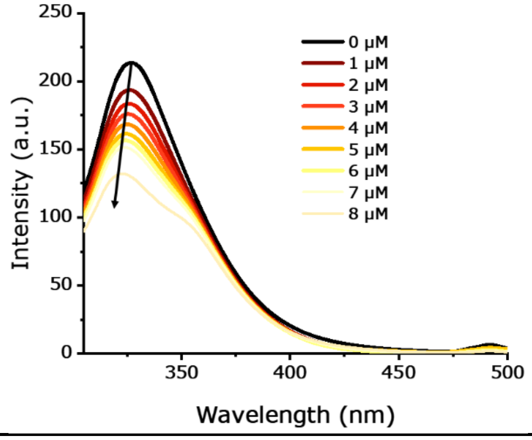

Sm(nal)$_2$

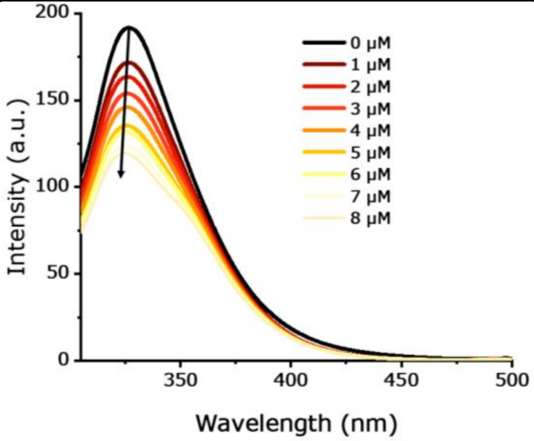

Eu(nal)$_2$

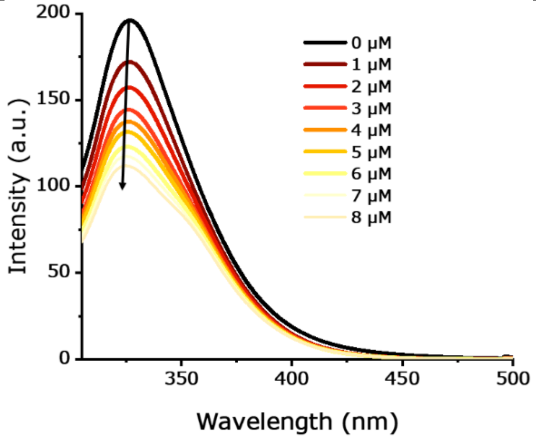

Gd(nal)$_2$

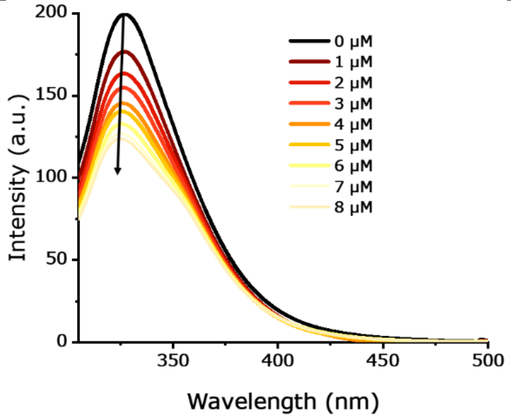

Tb(nal)$_2$

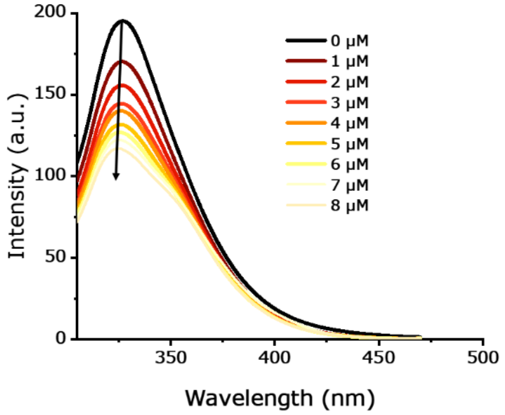

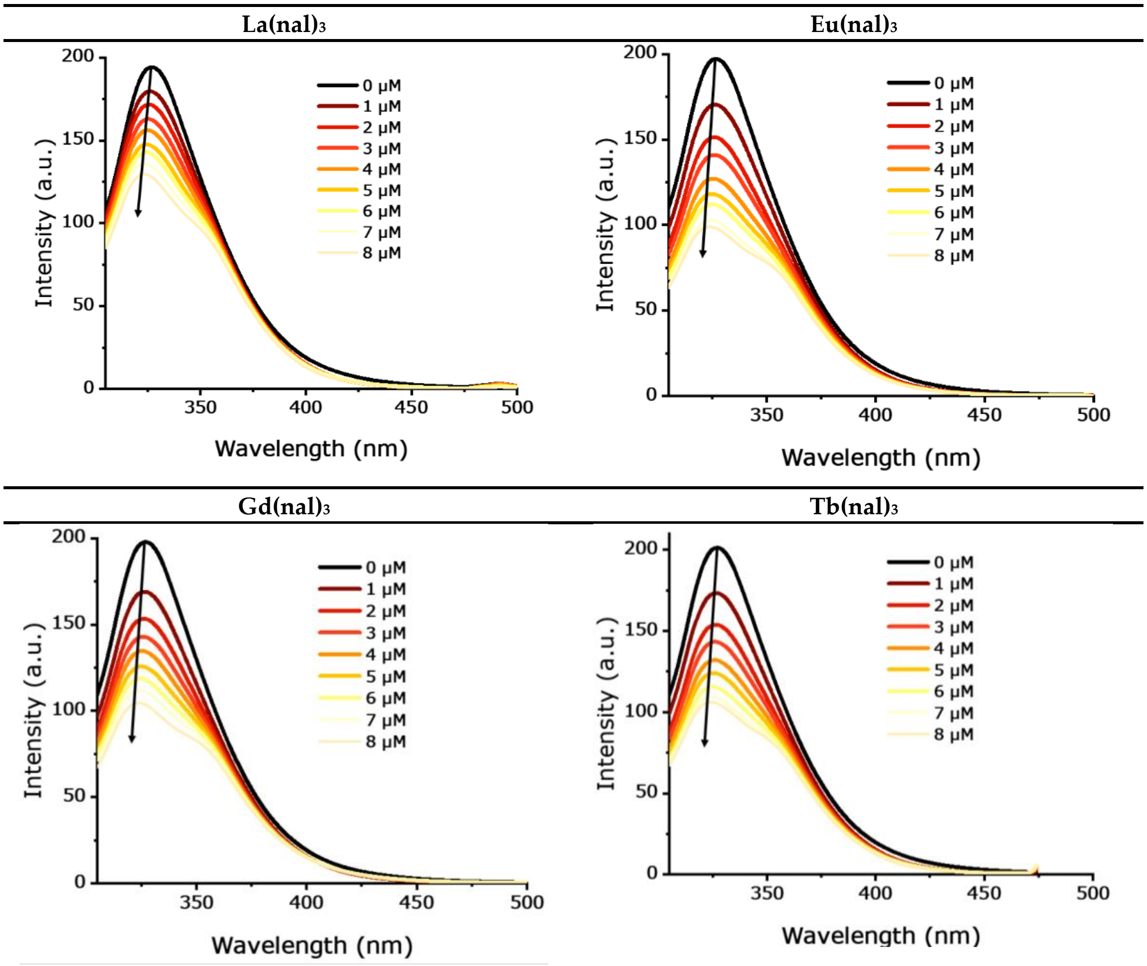

Figure S17. Changes in fluorescence intensity of free apo Tf *vs.* compound-apo Tf systems; the black arrows indicate a decrease of the intensity upon the addition of increasing amounts of compound. [apo-Tf] = 1 μM, [compound] = 0, 1, 2, 3, 4, 5, 6, 7, 8 μM.

Nalidixic acid

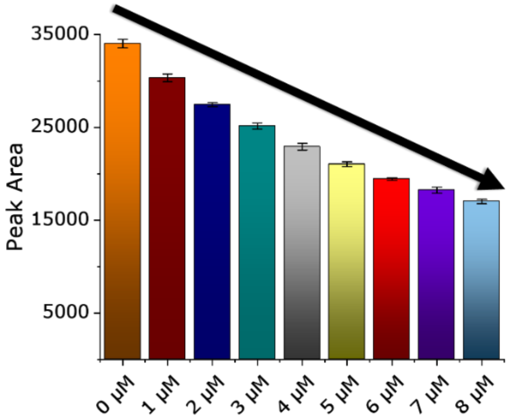

La(nal)2

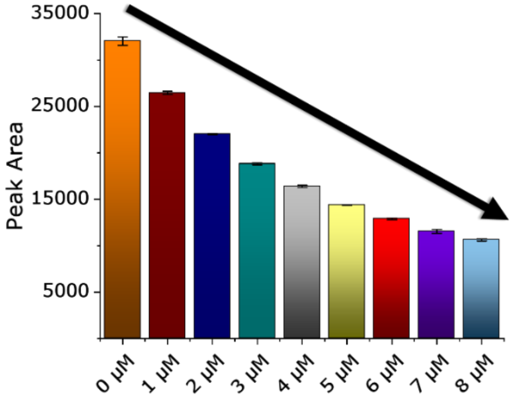

Sm(nal)2

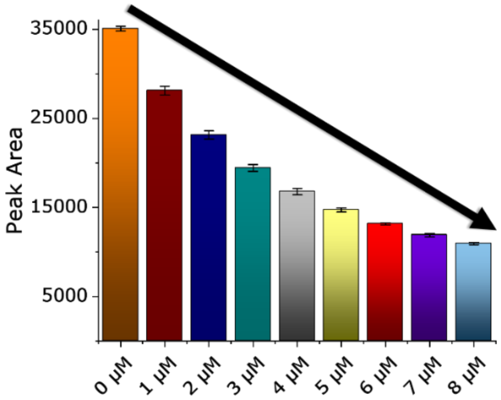

Eu(nal)2

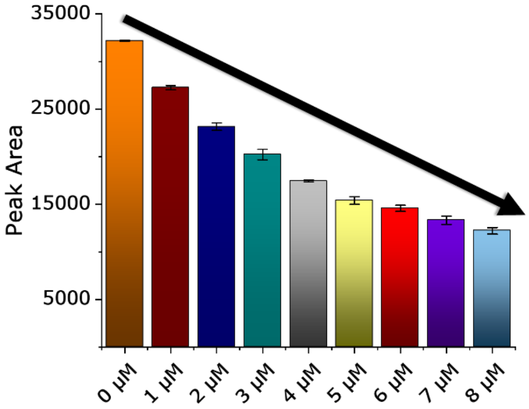

Gd(nal)2

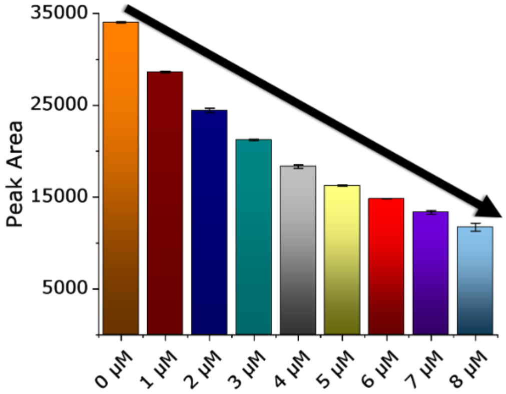

Tb(nal)2

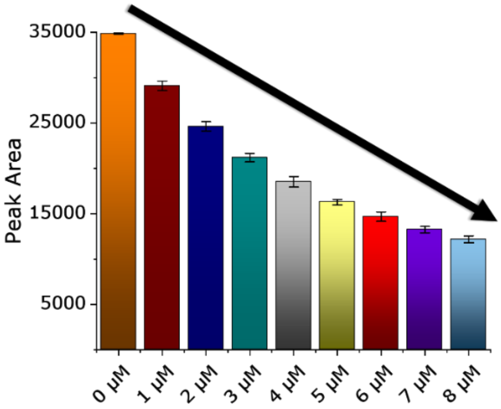

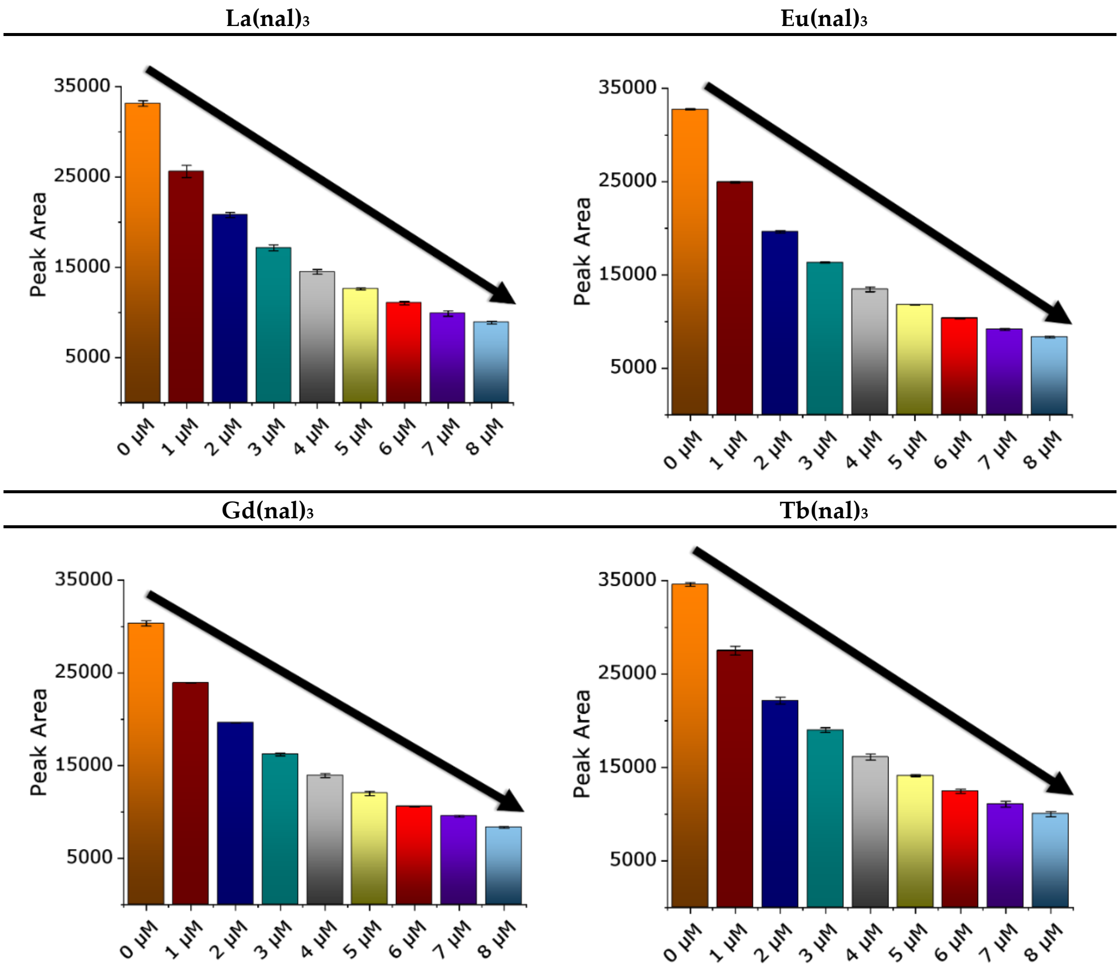

Figure S18. Variation of the HSA fluorescence peak area upon adding increasing amounts of the studied compounds. [HSA] = 2.5 μM, [compound] = 0, 1, 2, 3, 4, 5, 6, 7, 8 μM.

Nalidixic acid

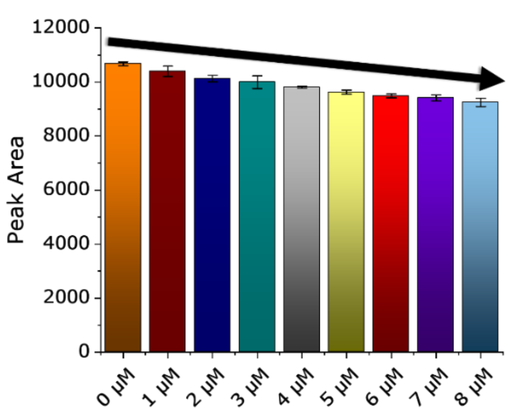

La(nal)$_2$

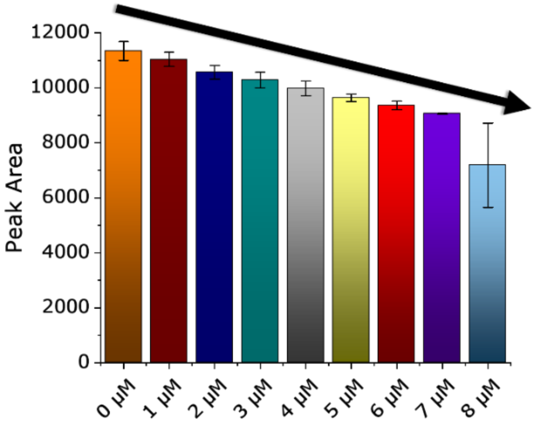

Sm(nal)$_2$

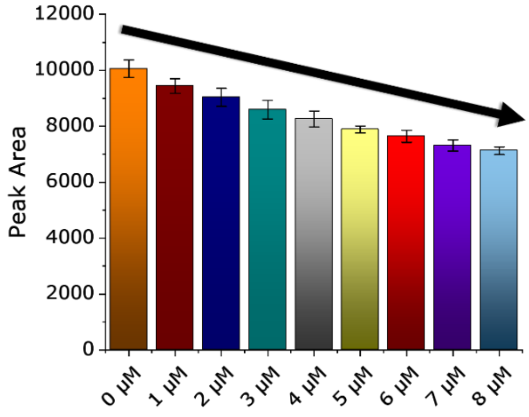

Eu(nal)$_2$

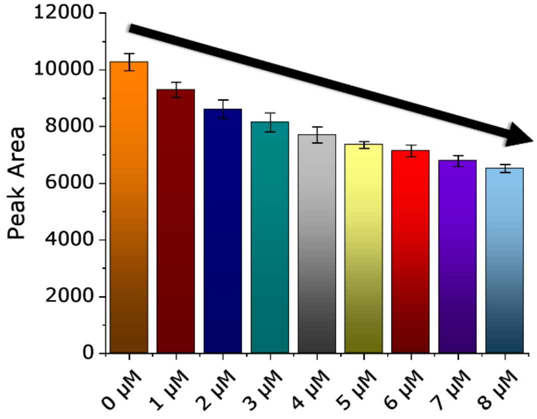

Gd(nal)$_2$

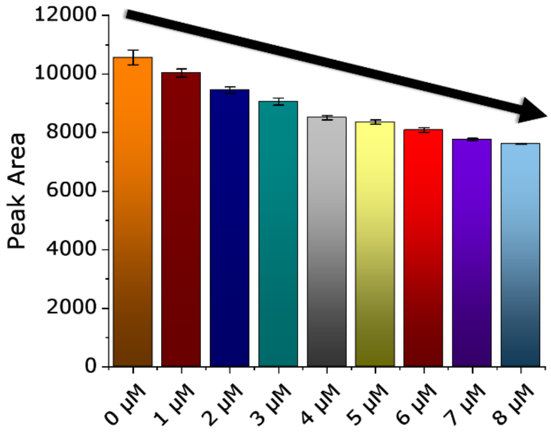

Tb(nal)$_2$

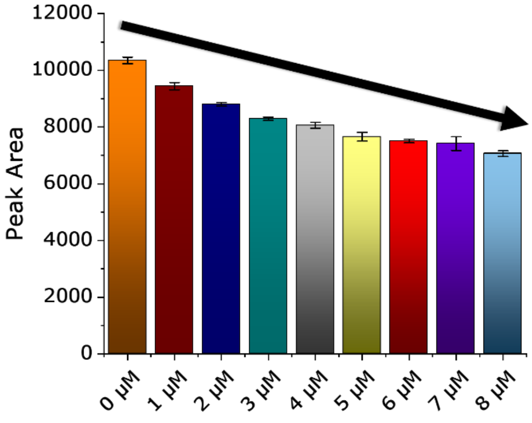

$La(nal)_3$	$Eu(nal)_3$
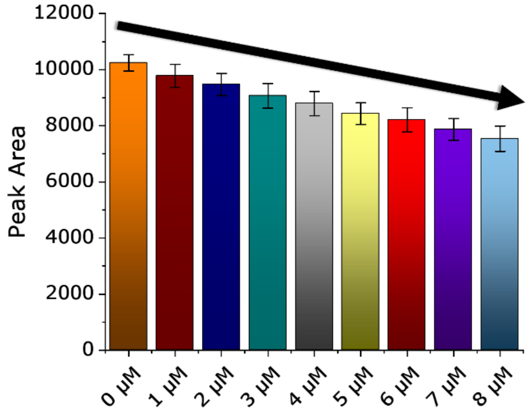	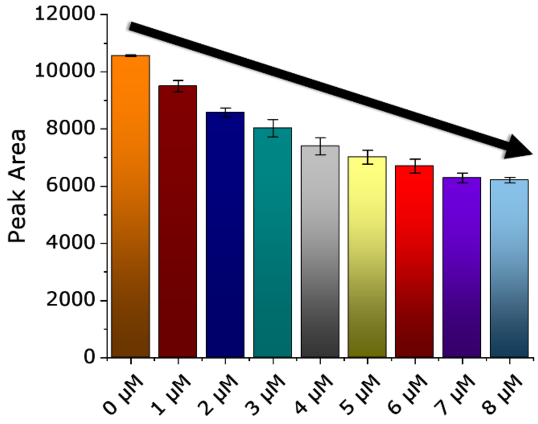

$Gd(nal)_3$	$Tb(nal)_3$
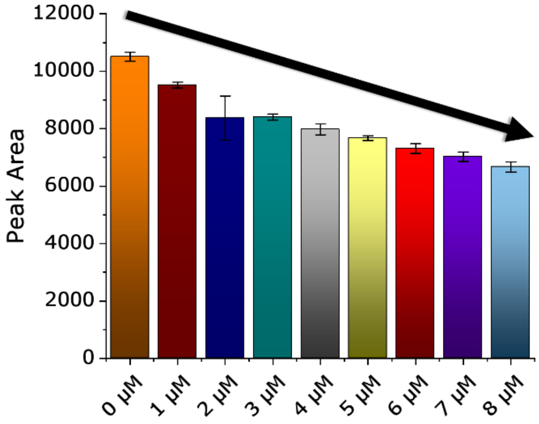	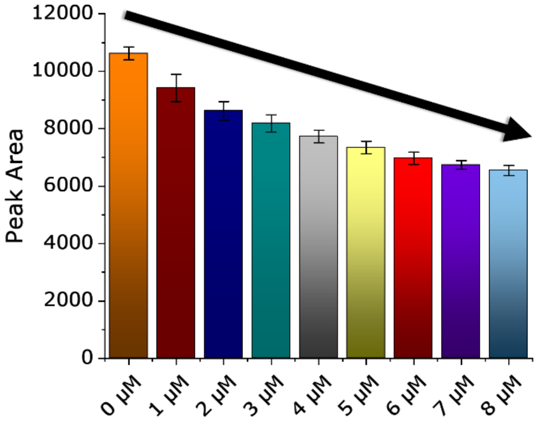

Figure S19. Variation of the apo-Tf fluorescence peak area upon adding increasing amounts of the studied compounds. [apo-Tf] = 1 μM, [compound] = 0, 1, 2, 3, 4, 5, 6, 7, 8 μM.

Classical Stern-Volmer	Modified Stern-Volmer

Nalidixic acid

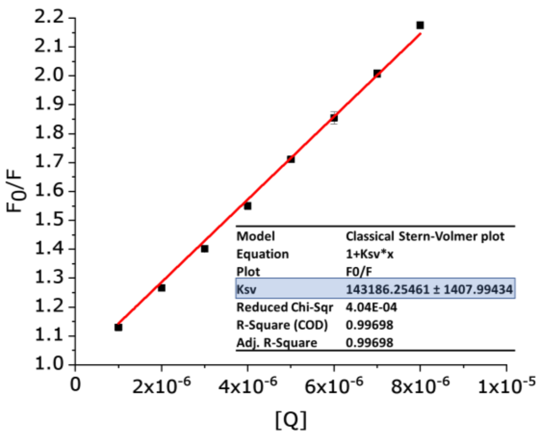

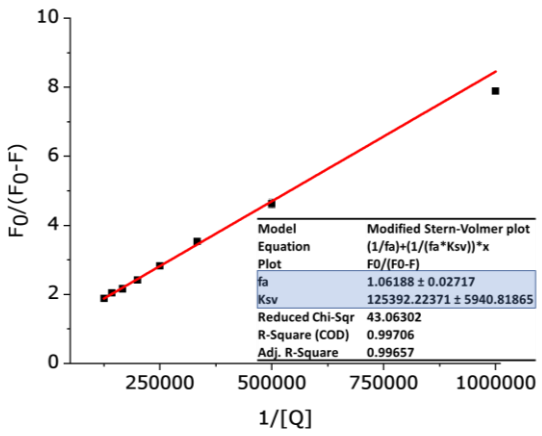

La(nal)2

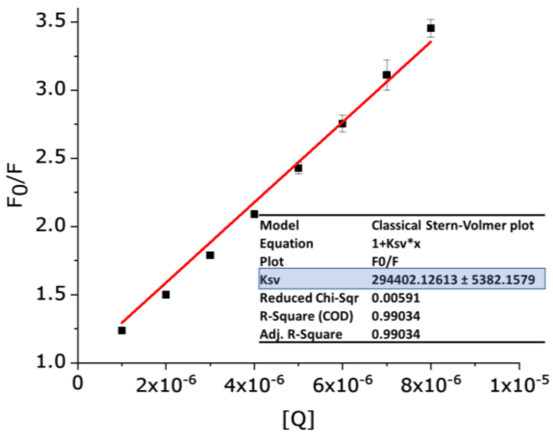

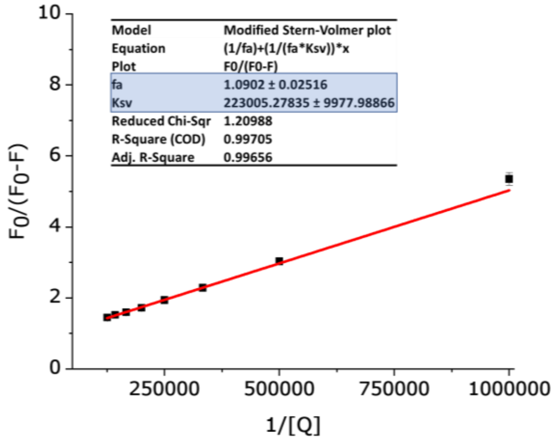

Sm(nal)2

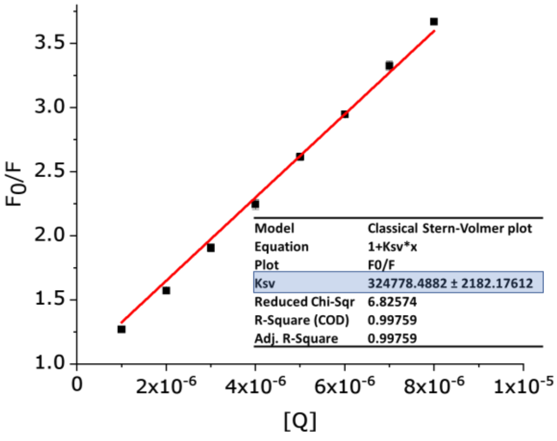

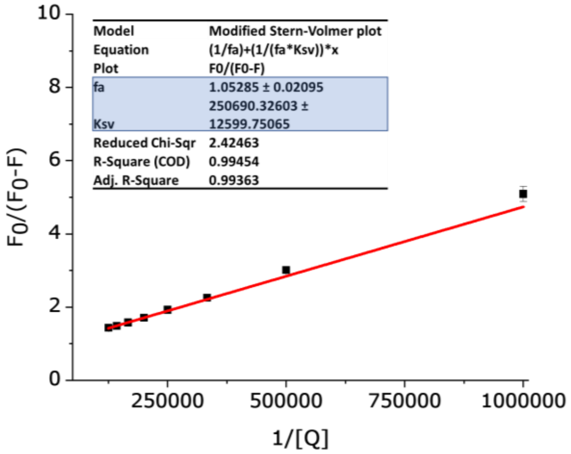

Classical Stern-Volmer	Modified Stern-Volmer
**$Eu(nal)_2$**	
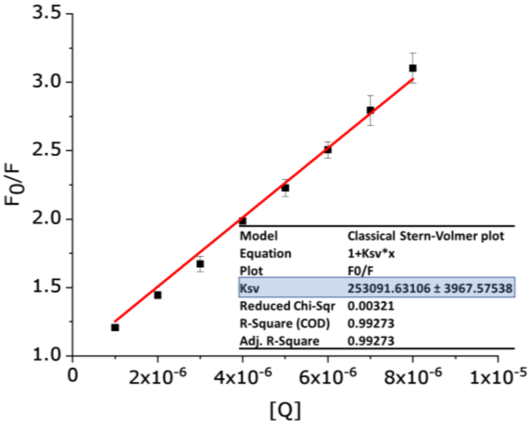	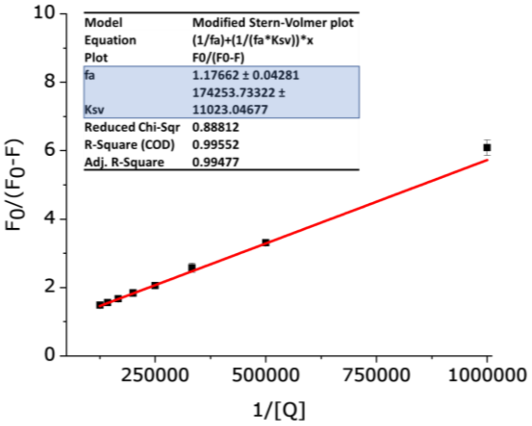
**$Gd(nal)_2$**	
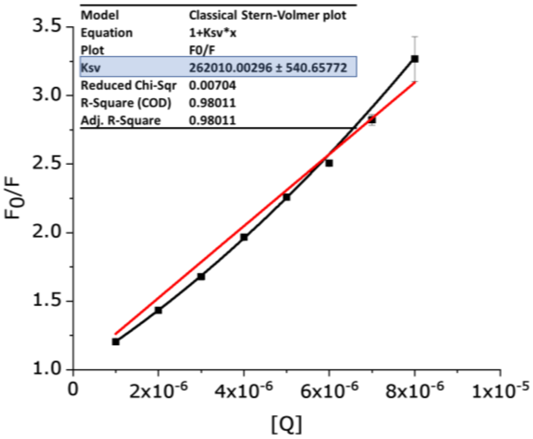	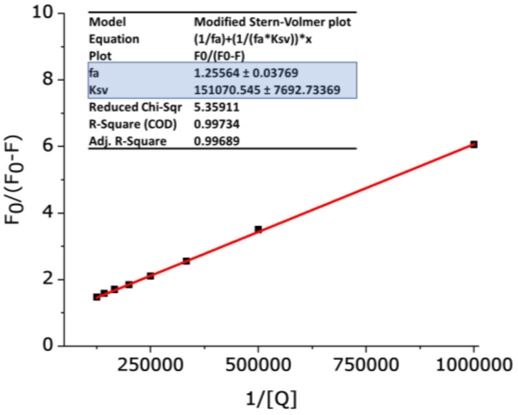
**$Tb(nal)_2$**	
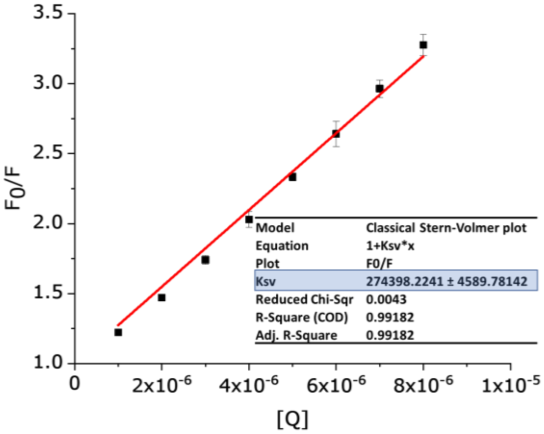	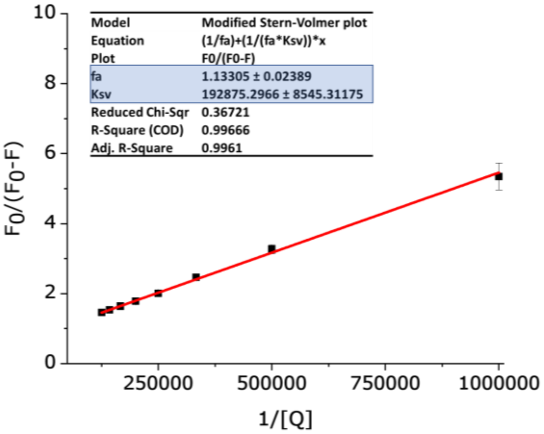

Classical Stern-Volmer	Modified Stern-Volmer

La(nal)$_3$

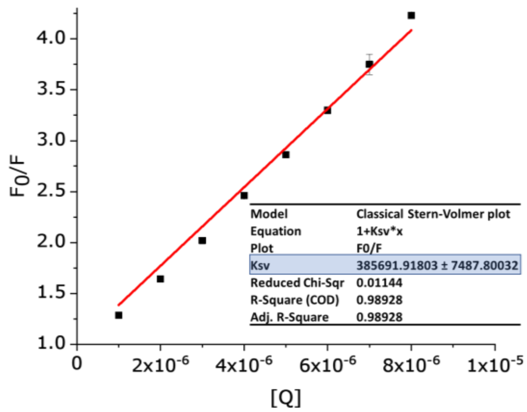

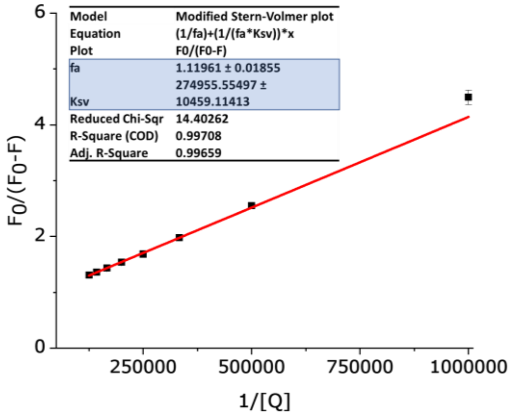

Eu(nal)$_3$

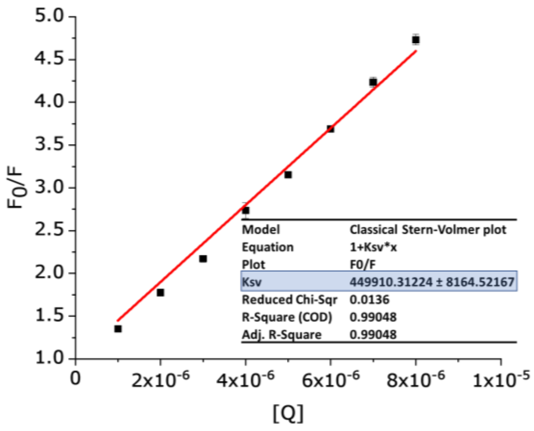

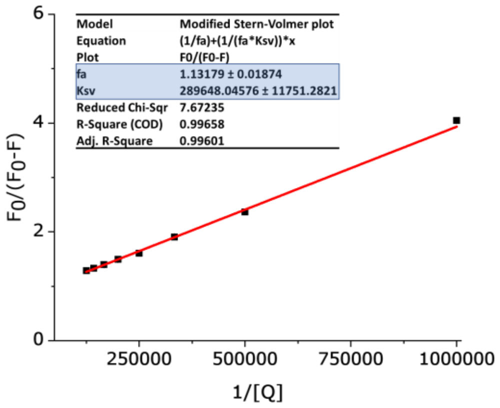

Gd(nal)$_3$

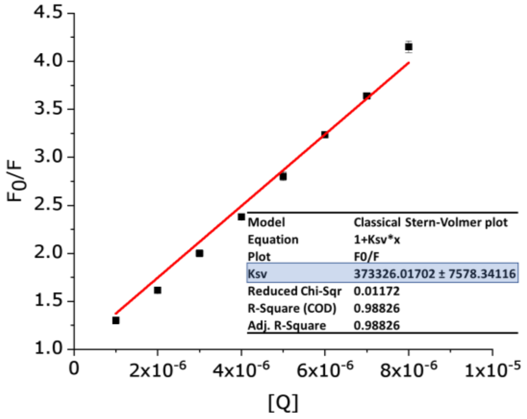

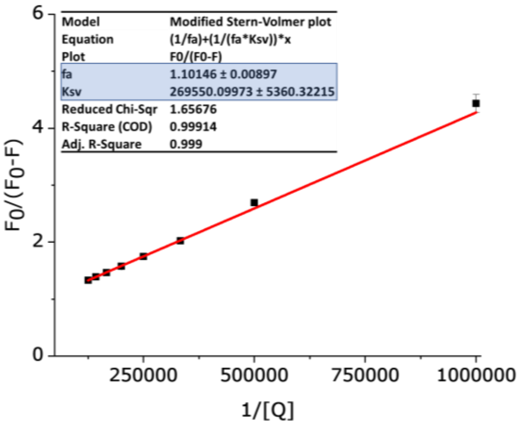

Classical Stern-Volmer	Modified Stern-Volmer
**Tb(nal)$_3$**	
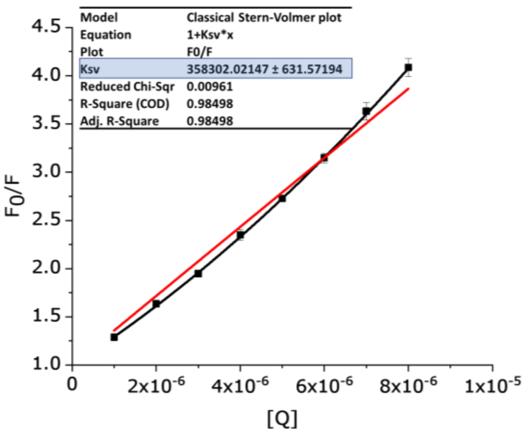	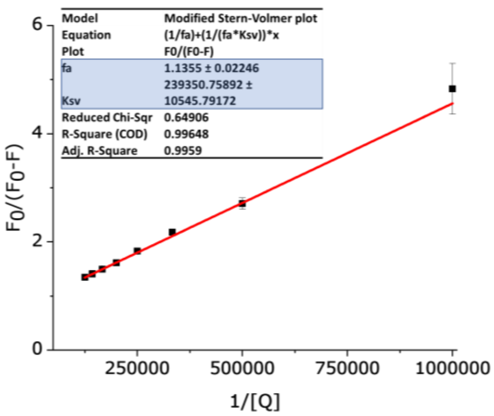

Figure S20. Classical (left) and modified (right) Stern-Volmer plots for each of the HSA interaction studies.

Classical Stern-Volmer	Modified Stern-Volmer

Nalidixic acid

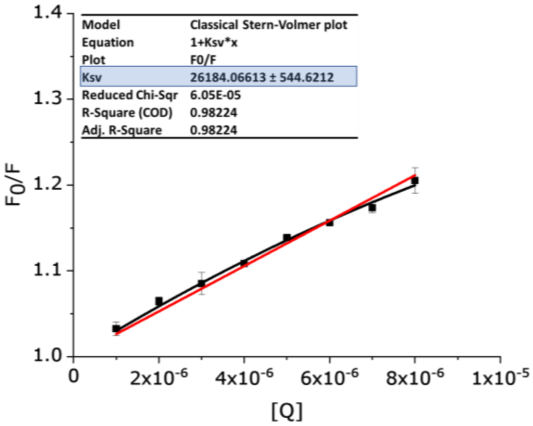

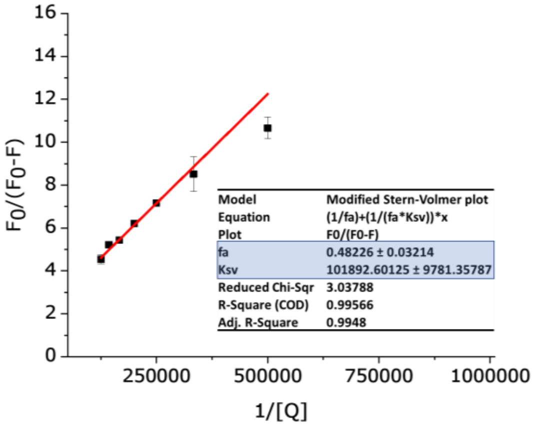

La(nal)2

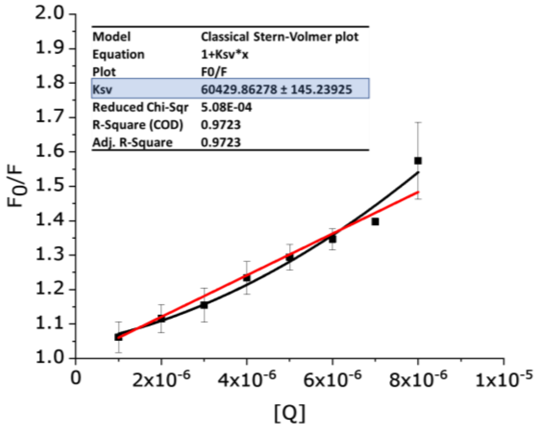

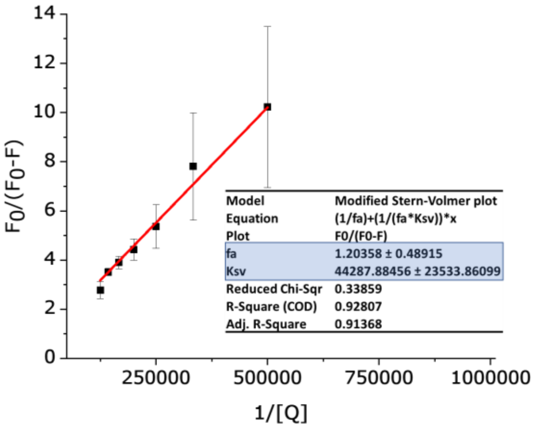

Sm(nal)2

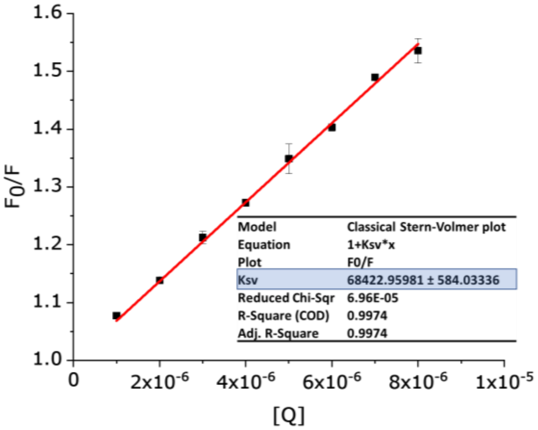

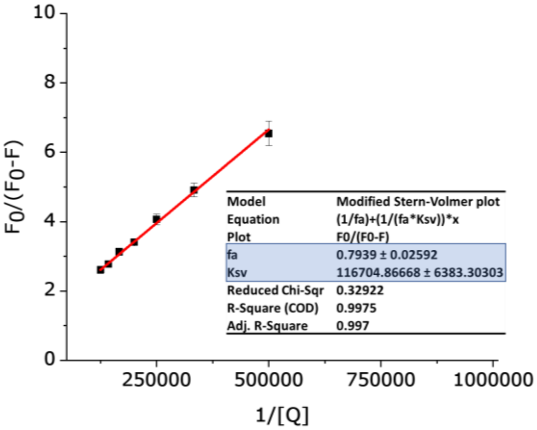

Classical Stern-Volmer	Modified Stern-Volmer

$Eu(nal)_2$

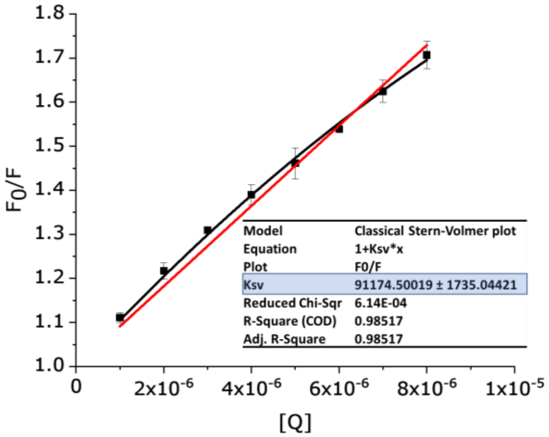

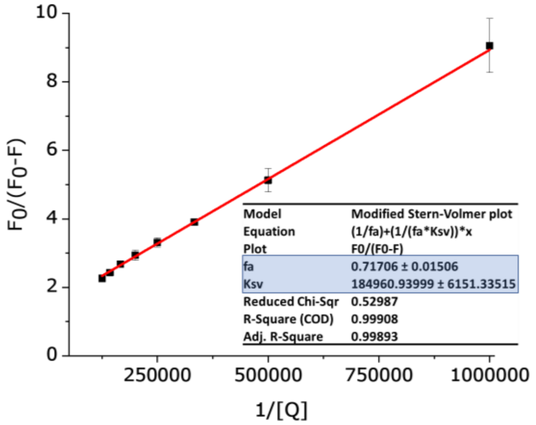

$Gd(nal)_2$

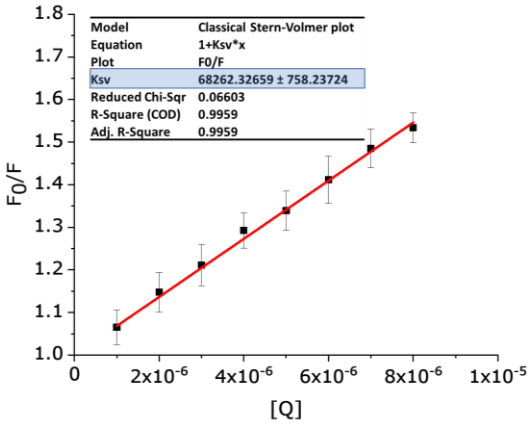

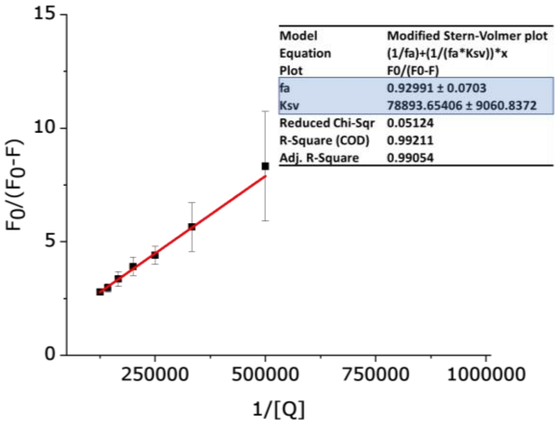

$Tb(nal)_2$

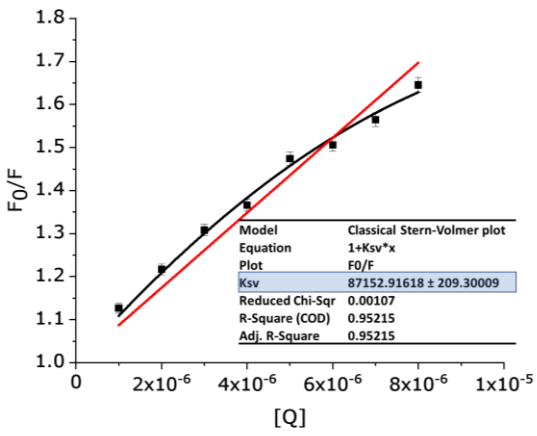

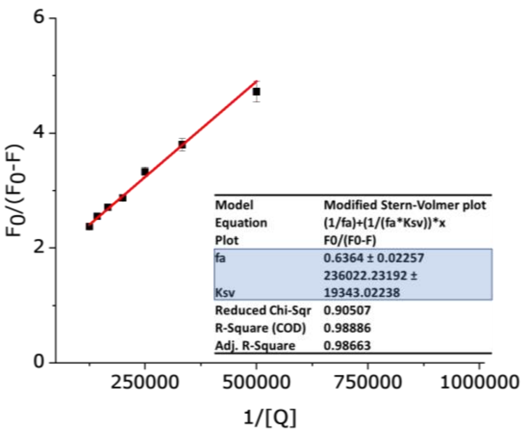

La(nal)3

Classical Stern-Volmer	Modified Stern-Volmer

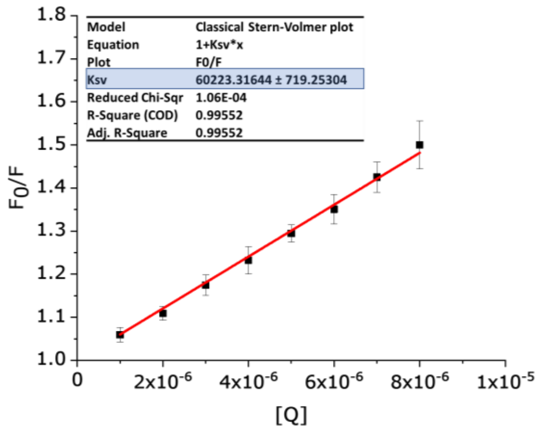

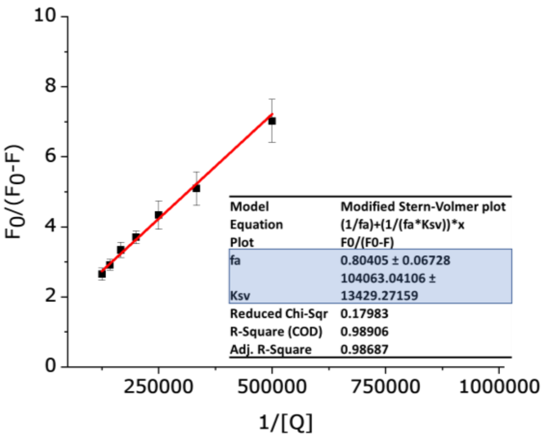

Eu(nal)3

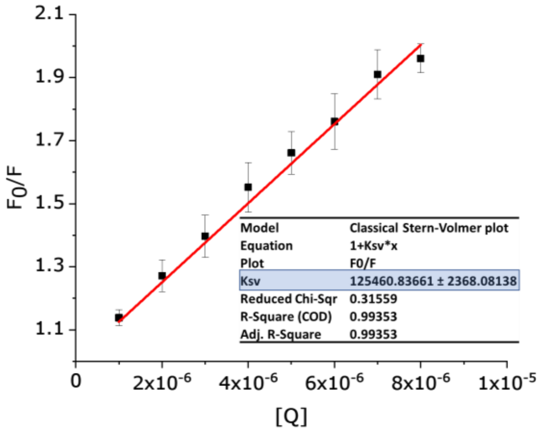

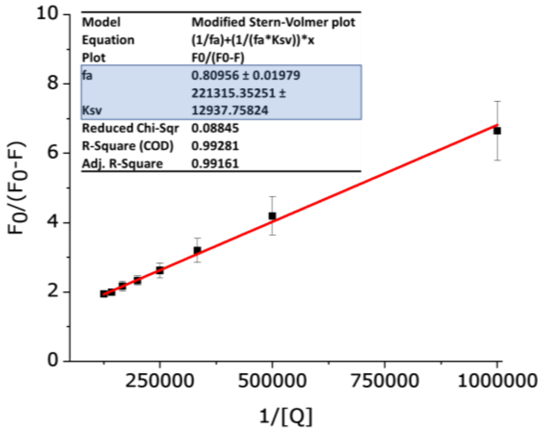

Gd(nal)3

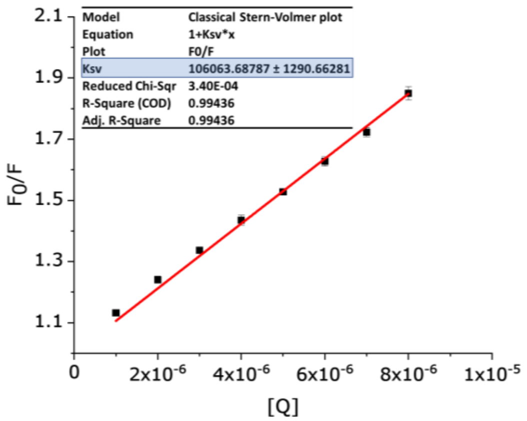

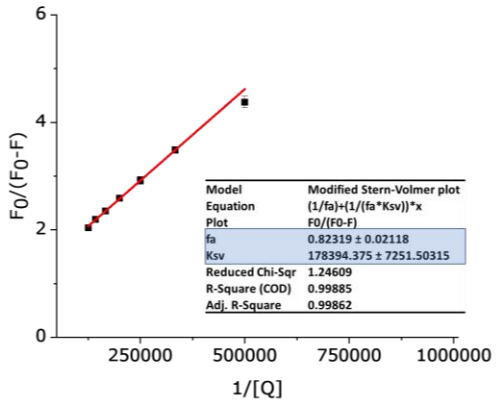

Tb(nal)$_3$	
**Classical Stern-Volmer**	**Modified Stern-Volmer**

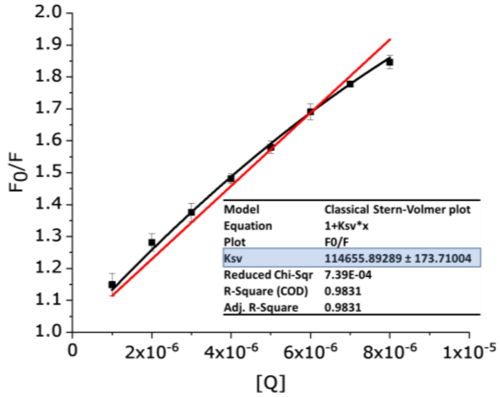

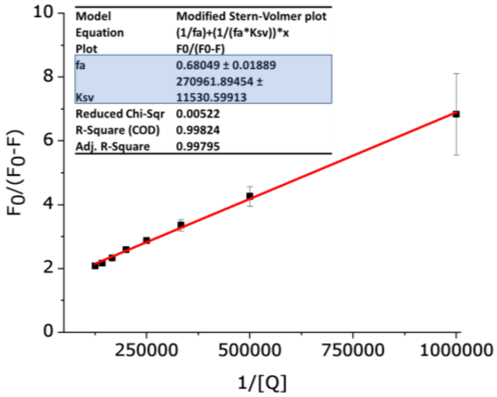

Figure S21. Classical (left) and modified (right) Stern-Volmer plots for the apo-Tf interaction assay.

Nalidixic acid

Ka | **Kd**

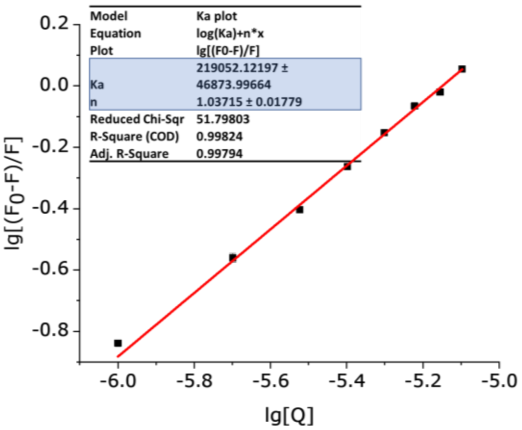

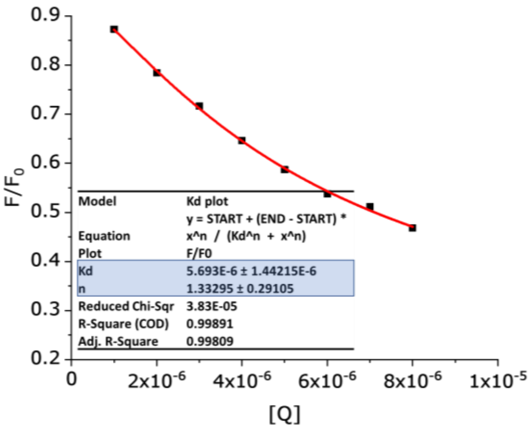

La(nal)2

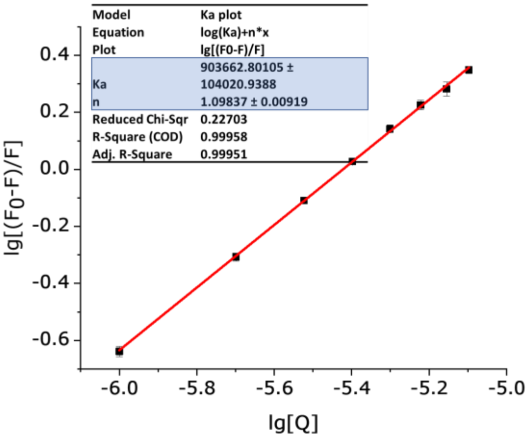

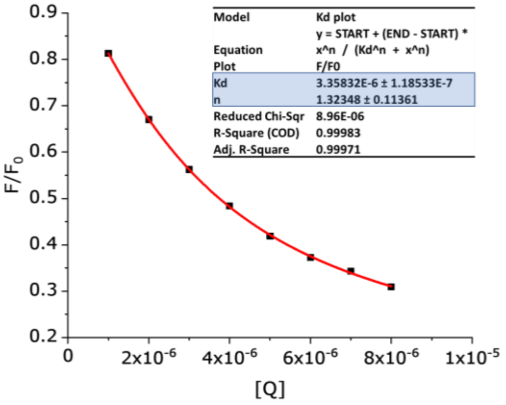

Sm(nal)2

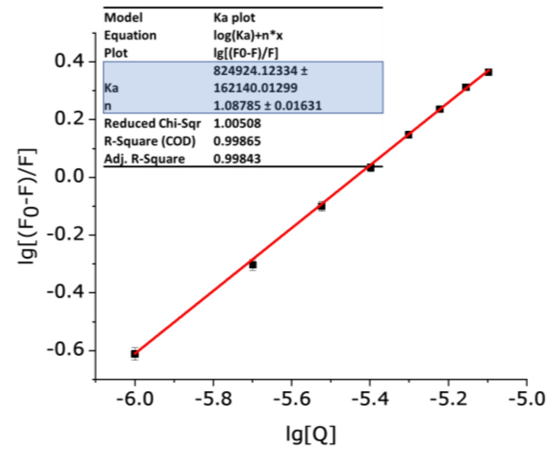

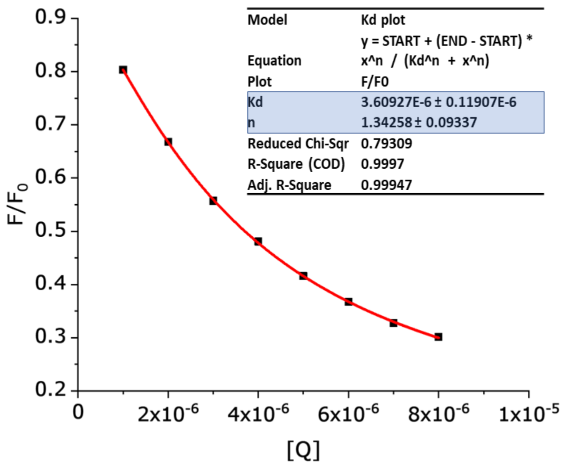

Eu(nal)2

Ka	Kd

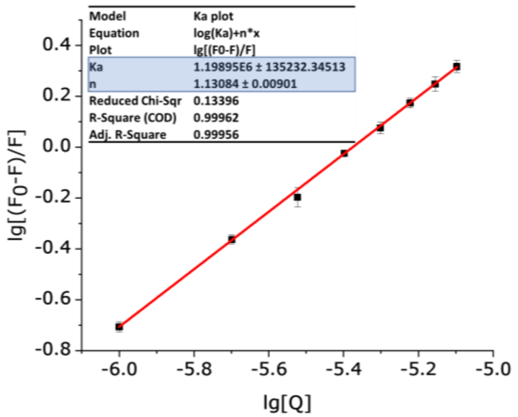

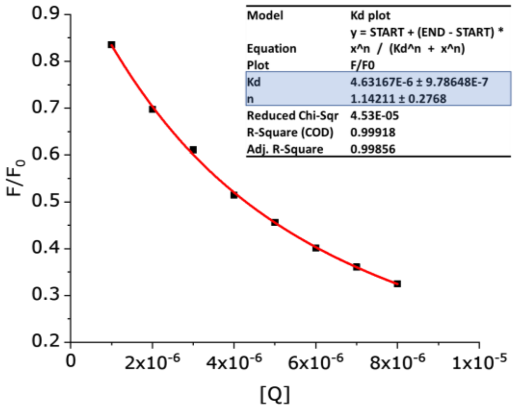

Gd(nal)2

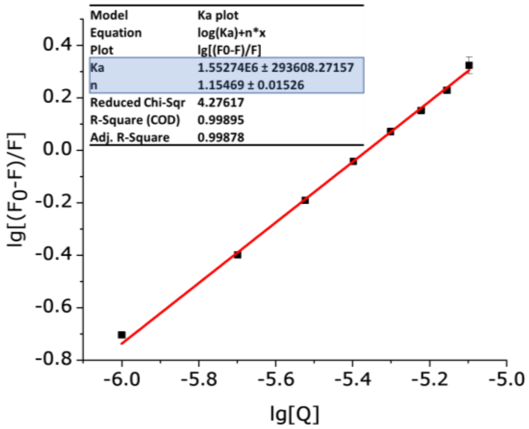

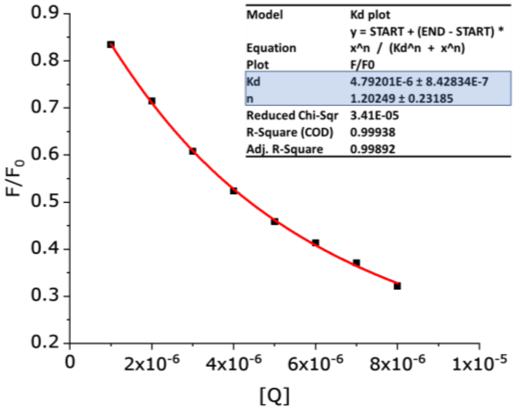

Tb(nal)2

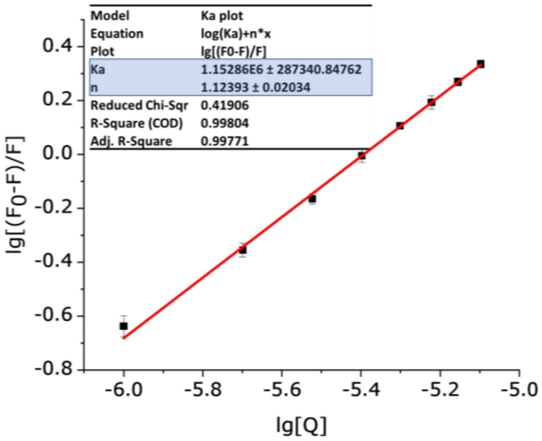

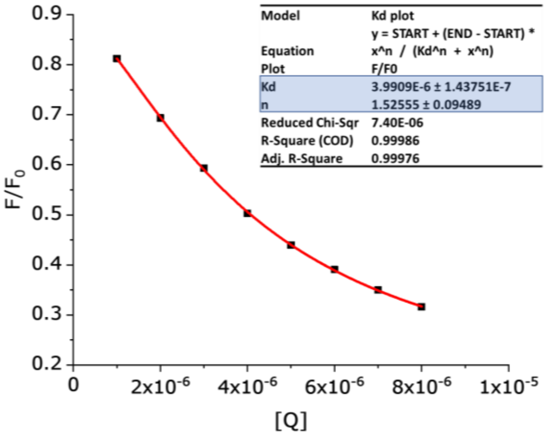

La(nal)3

Ka	Kd

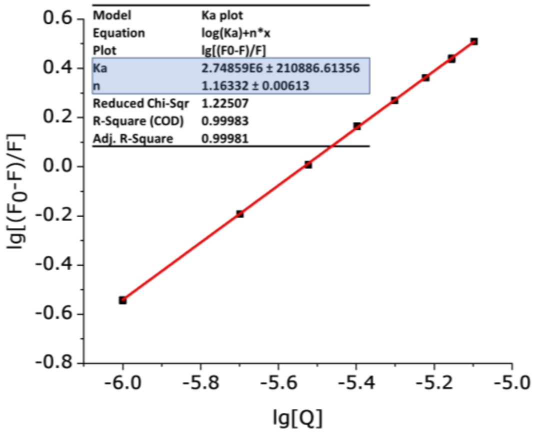

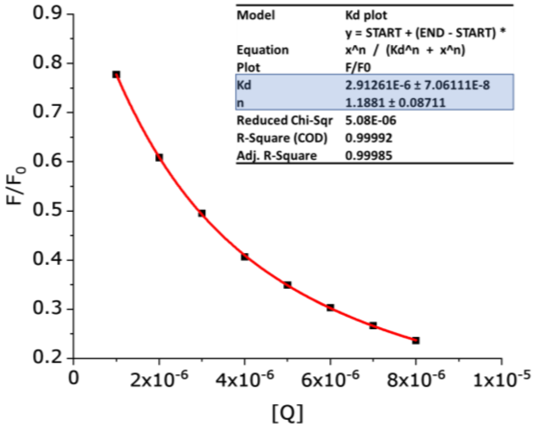

Eu(nal)3

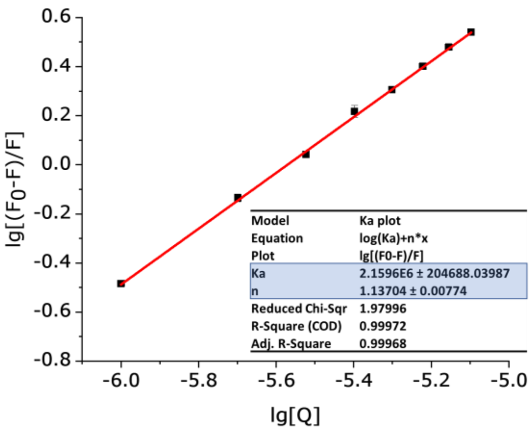

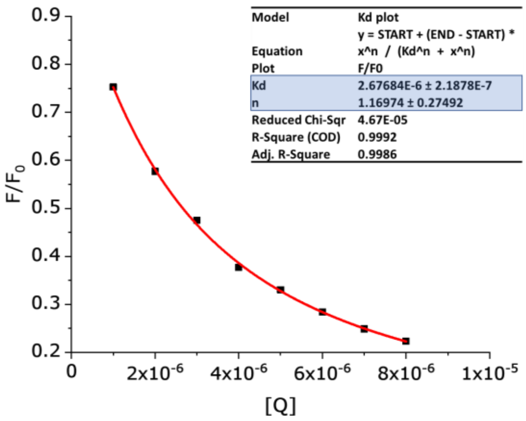

Gd(nal)3

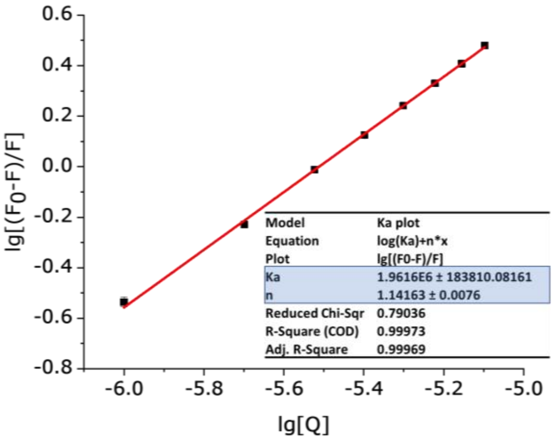

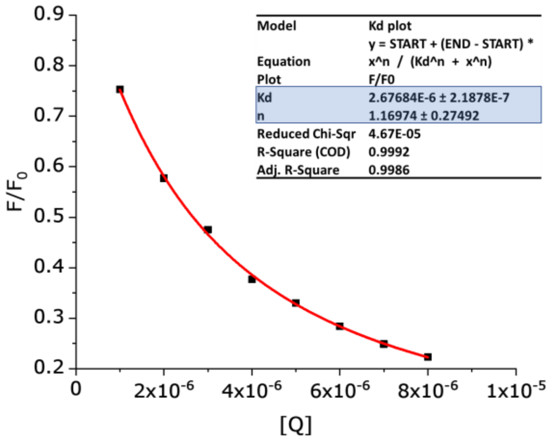

Tb(nal)3	
Ka	Kd

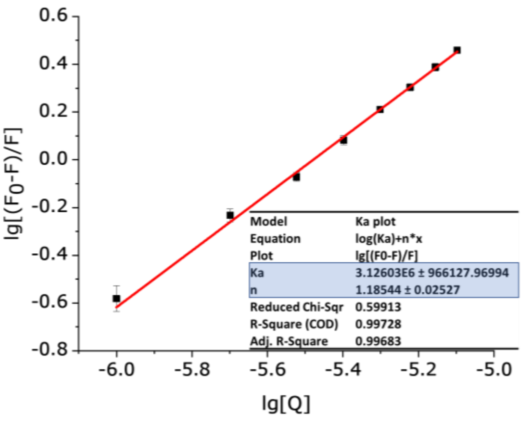

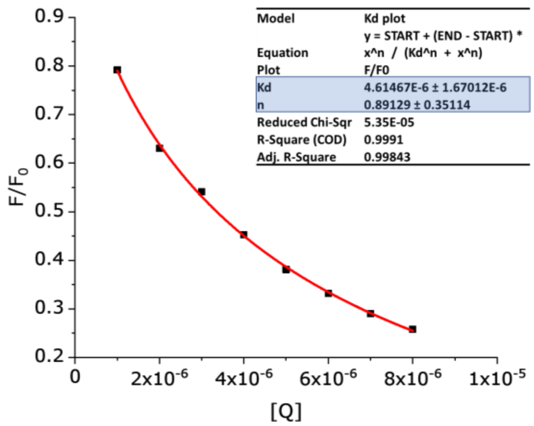

Figure S22. Representation of Ka and Kd constants for each of the studied compound-HSA systems..

Nalidixic acid

Ka

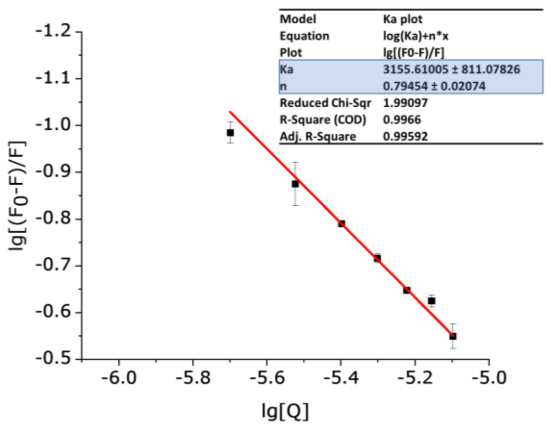

Kd

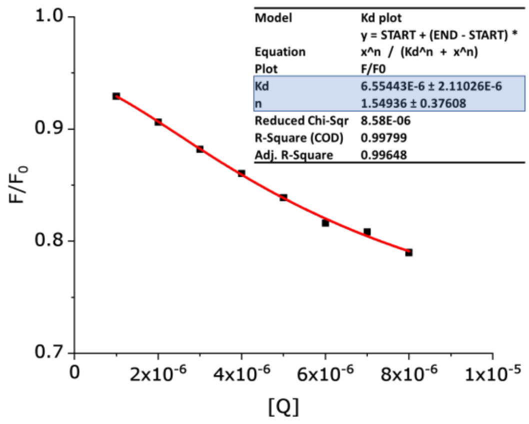

La(nal)2

Ka

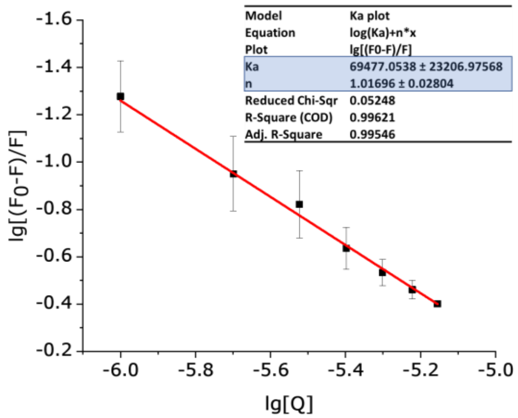

Kd

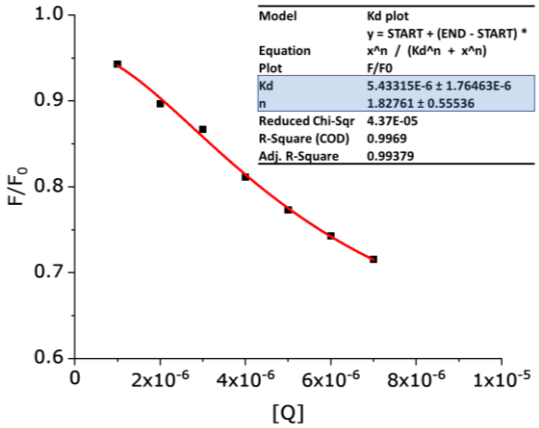

Sm(nal)2

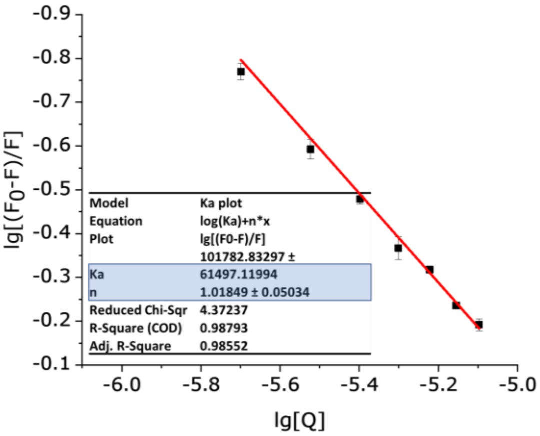

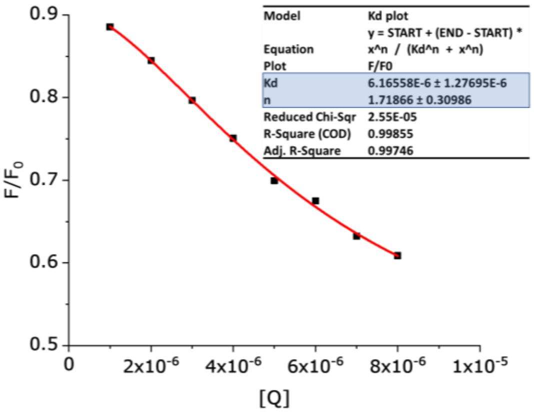

Eu(nal)$_2$

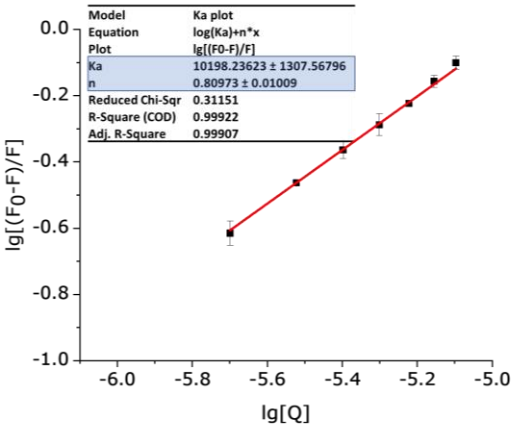

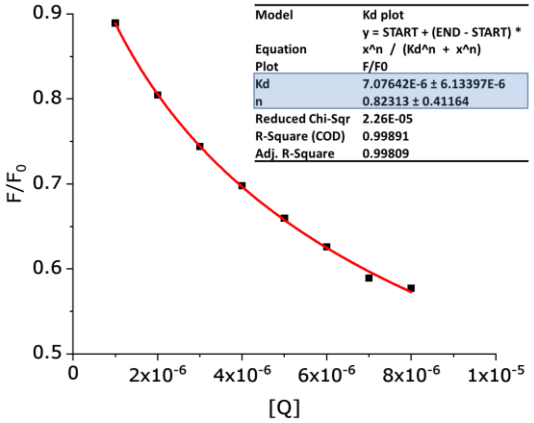

Gd(nal)$_2$

**Ka**	**Kd**

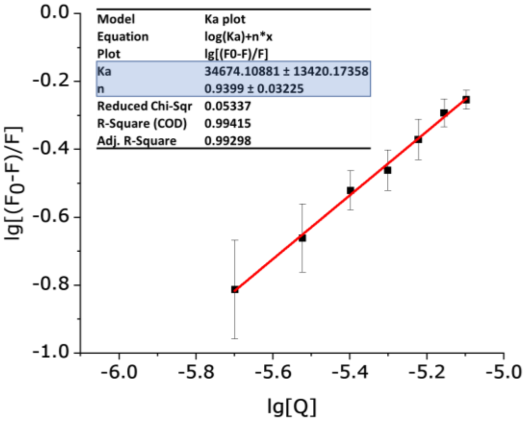

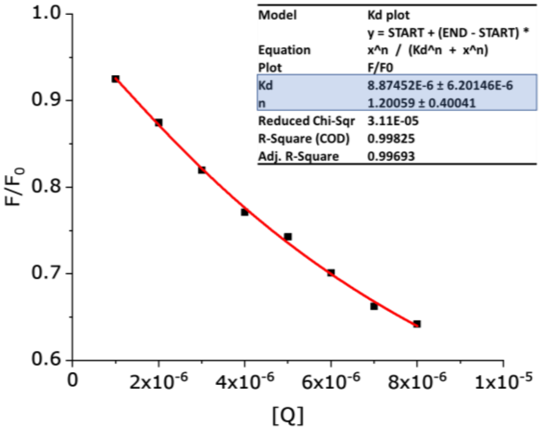

Tb(nal)$_2$

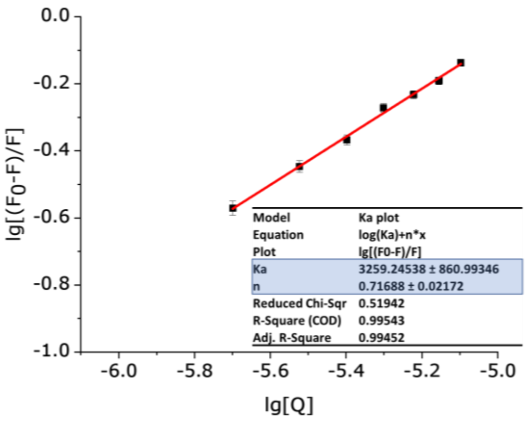

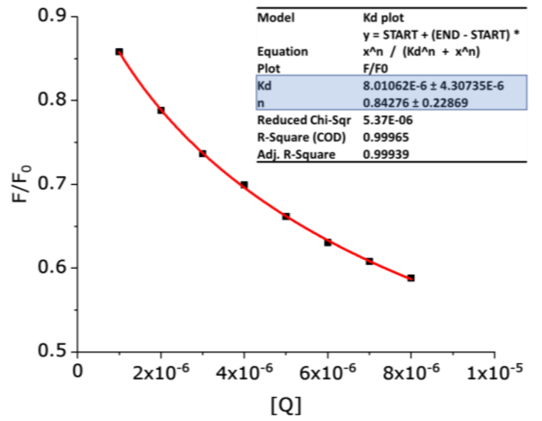

La(nal)$_3$

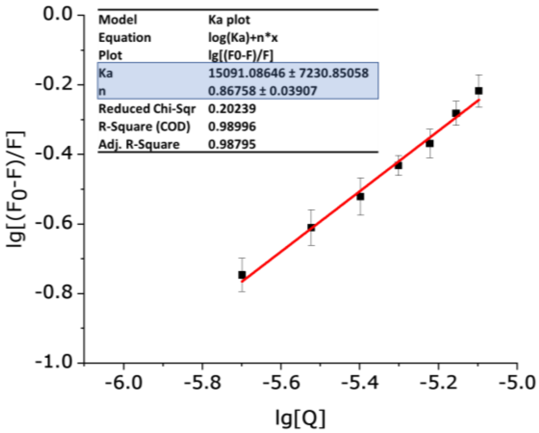

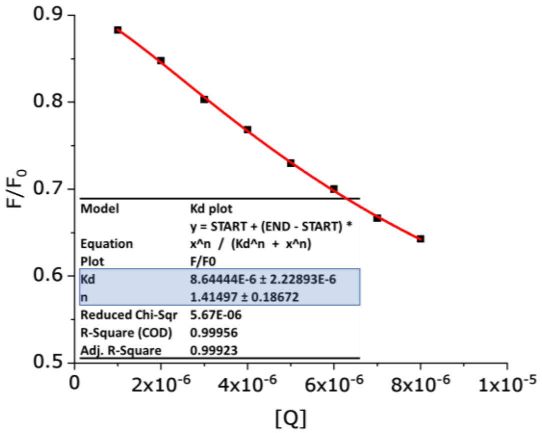

Eu(nal)$_3$

**Ka**	**Kd**

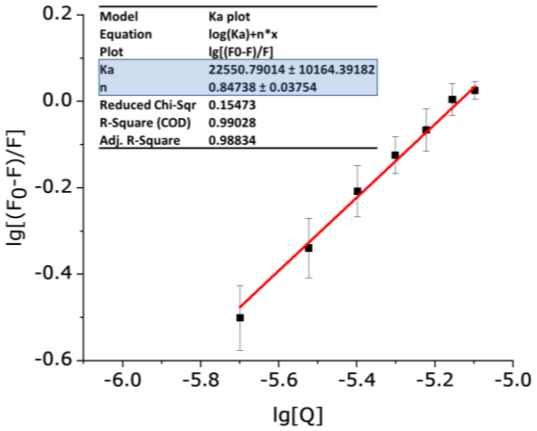

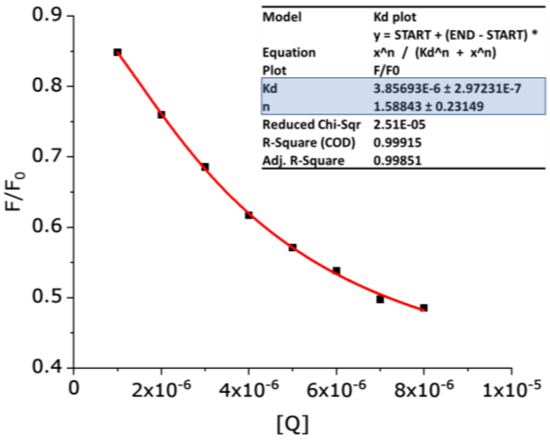

Gd(nal)$_3$

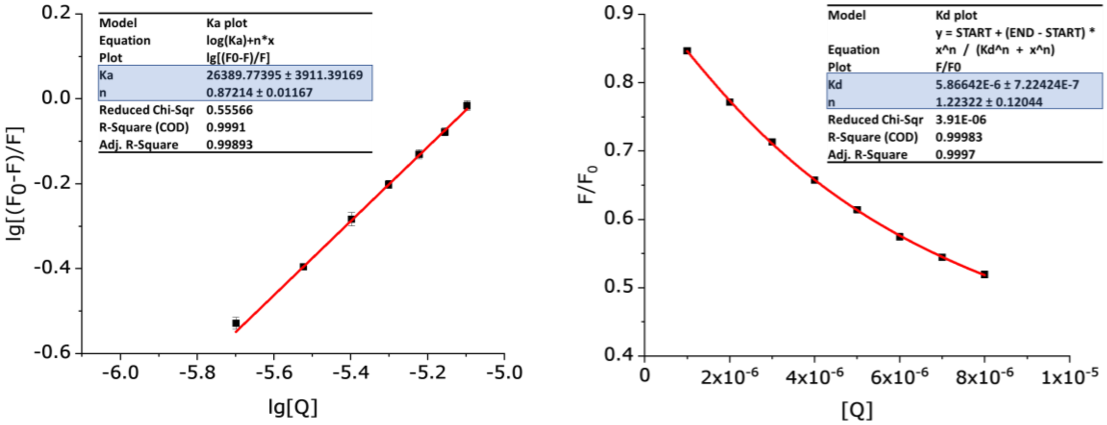

Tb(nal)$_3$

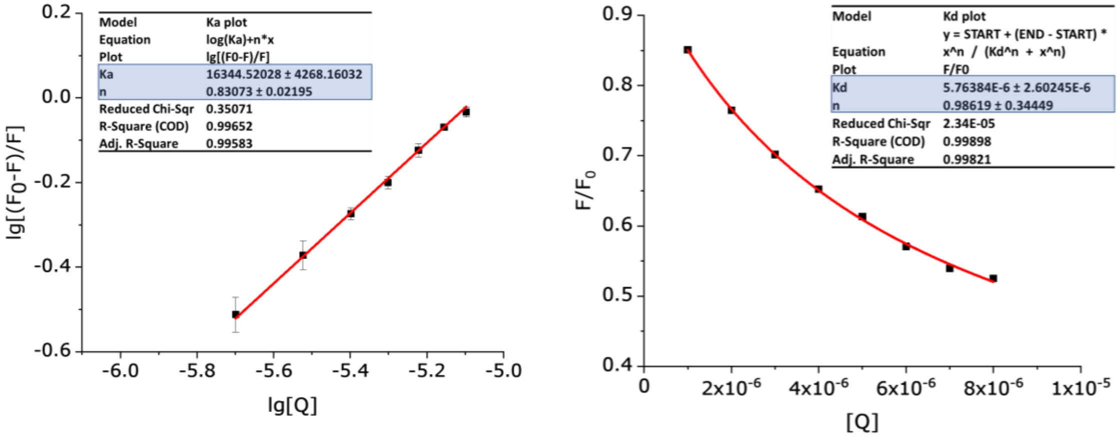

Figure S23. Representation of Ka and Kd constants for each of the studied compound-apo-Tf systems.

Nalidixic acid	$La(nal)_2$

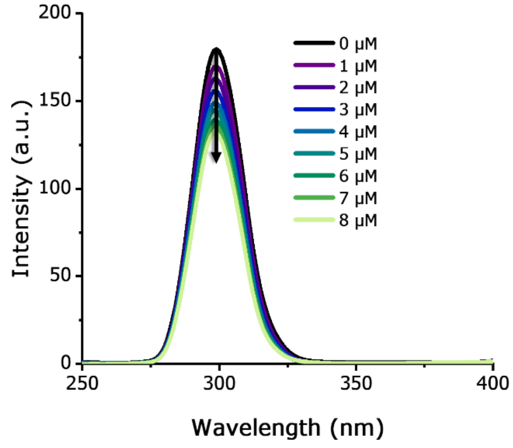

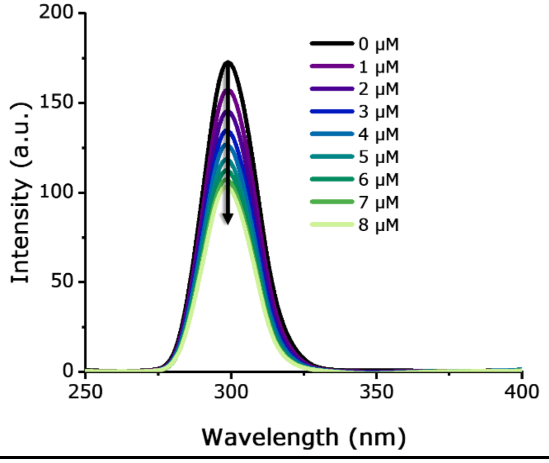

$Sm(nal)_2$	$Eu(nal)_2$

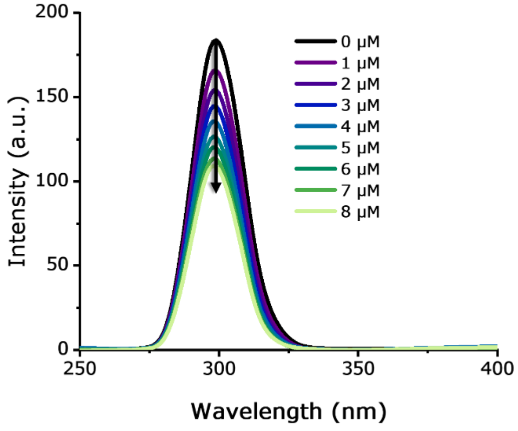

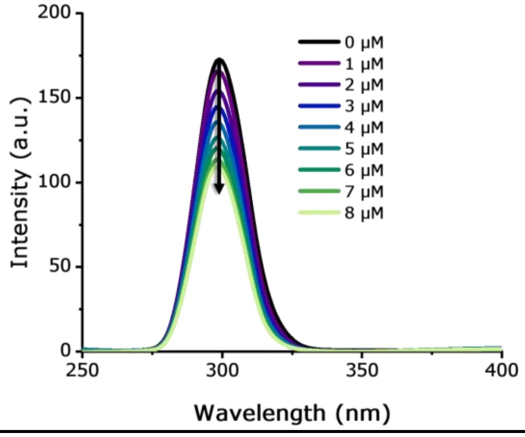

$Gd(nal)_2$	$Tb(nal)_2$

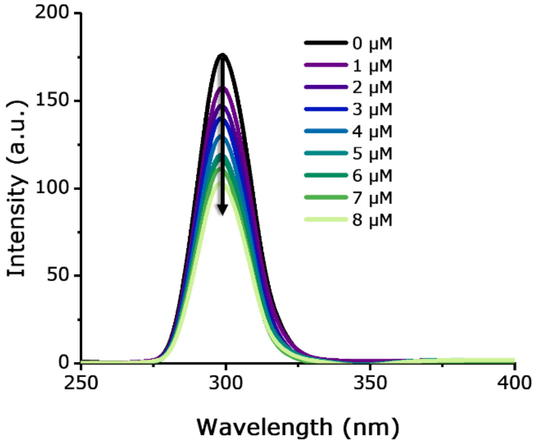

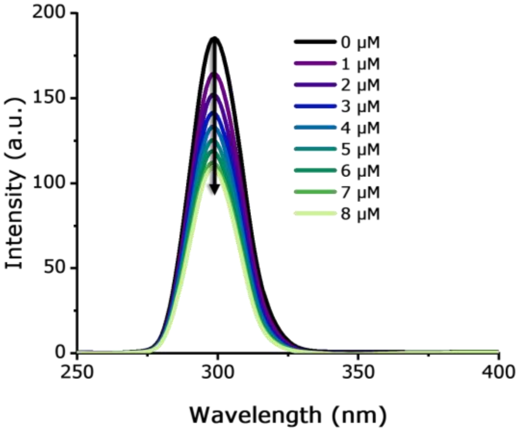

La(nal)$_3$	Eu(nal)$_3$
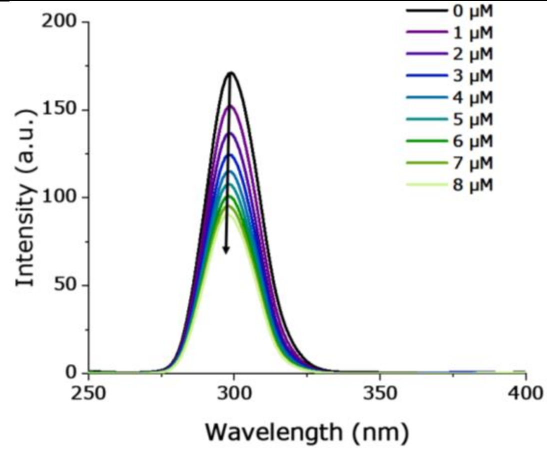	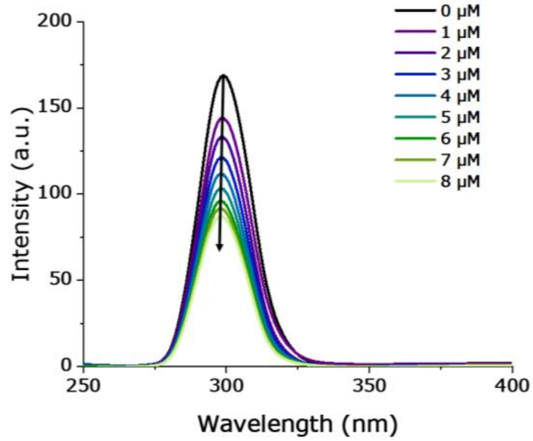

Gd(nal)$_3$	Tb(nal)$_3$
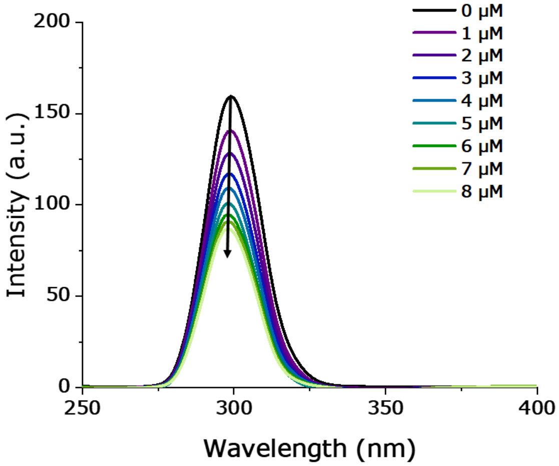	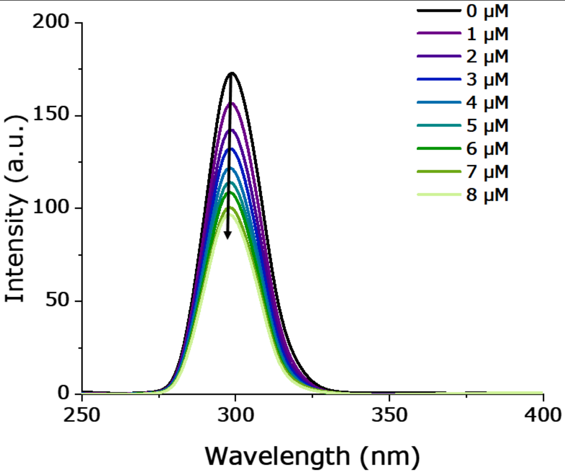

Figure S24. Synchronous spectra for the HSA interaction systems recorded at $\Delta\lambda$= 15 nm. [HSA] = 2.5 μM, [compound] = 0, 1, 2, 3, 4, 5, 6, 7, 8 μM.

Nalidixic acid

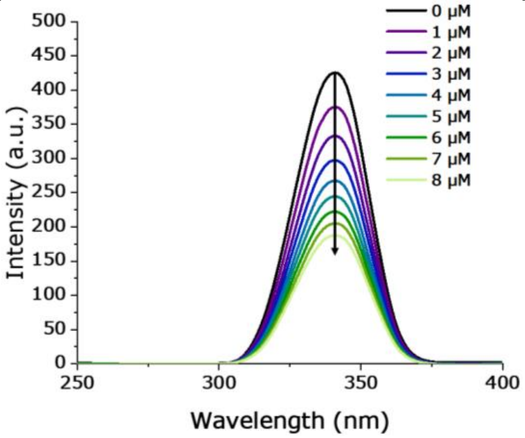

La(nal)$_2$

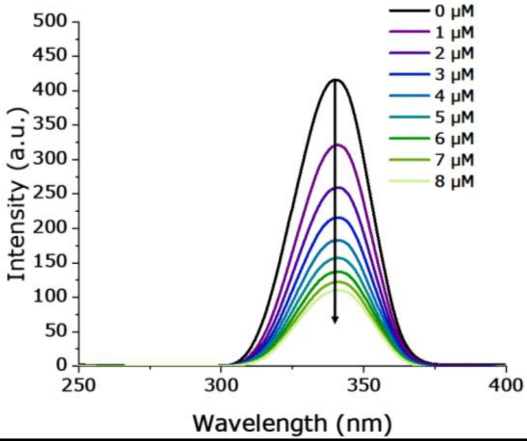

Sm(nal)$_2$

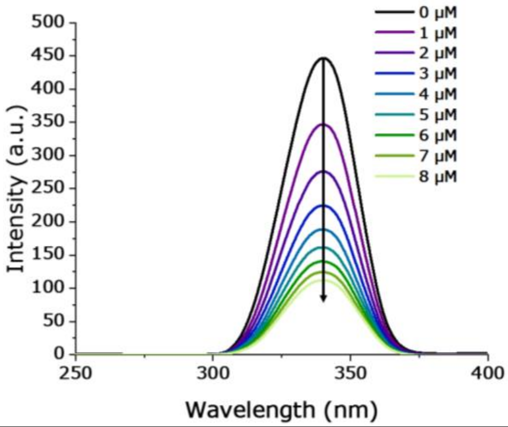

Eu(nal)$_2$

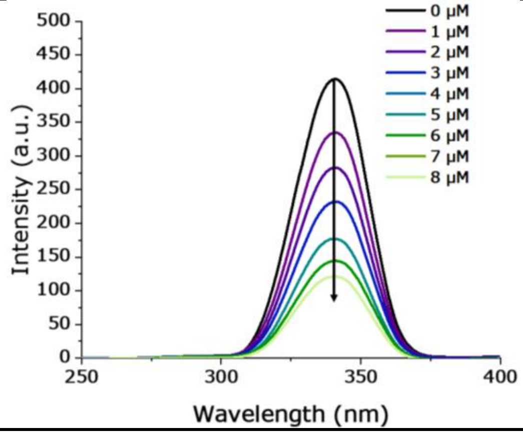

Gd(nal)$_2$

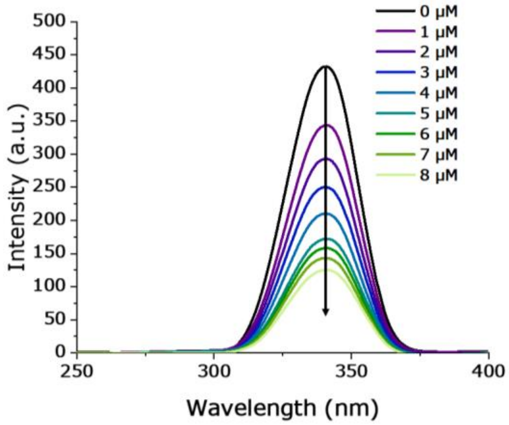

Tb(nal)$_2$

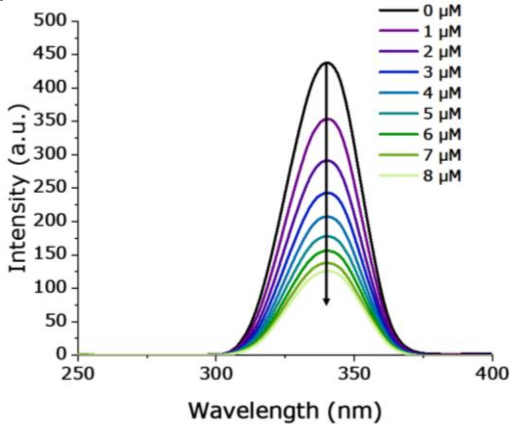

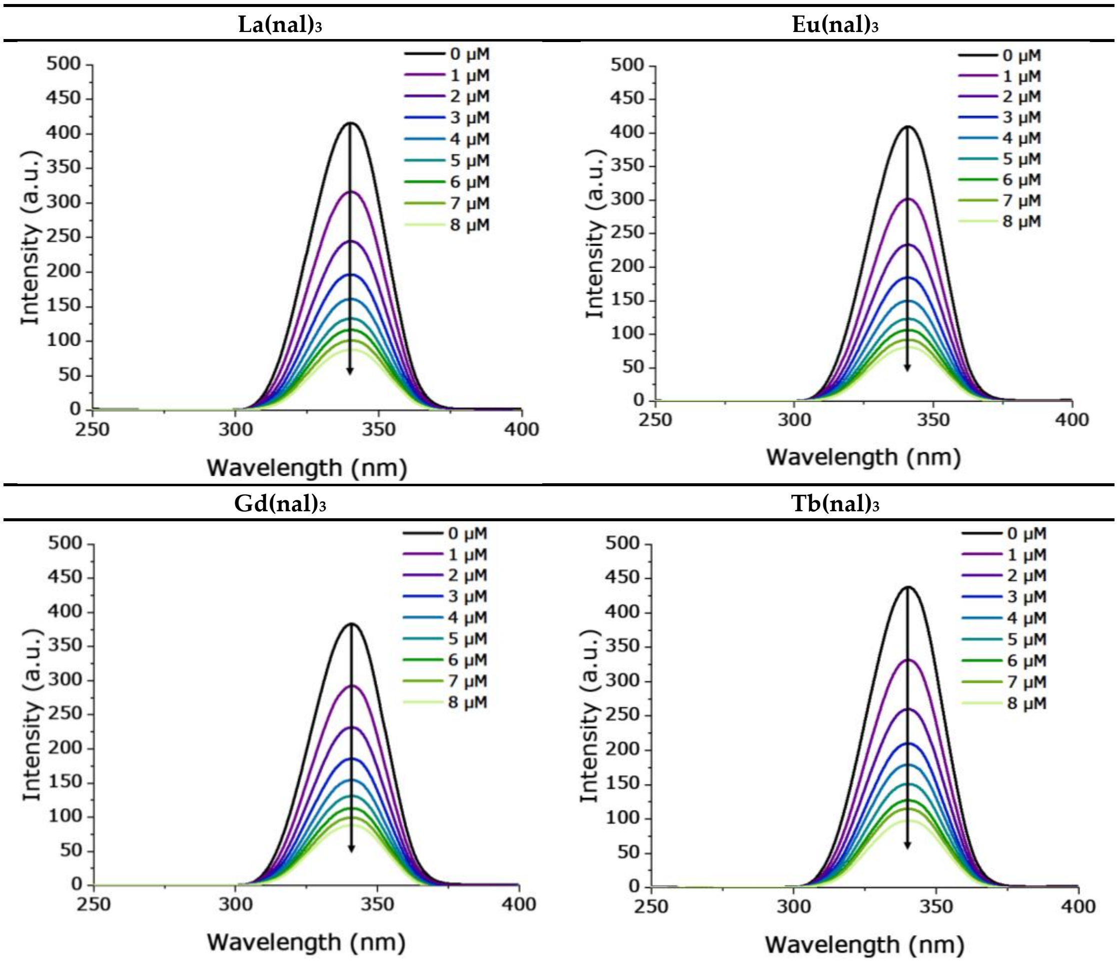

Figure S25. Synchronous spectra for the HSA interaction systems recorded at at $\Delta\lambda$= 60 nm. [HSA] = 2.5 μM, [compound] = 0, 1, 2, 3, 4, 5, 6, 7, 8 μM.

<table>
<tr><th>Nalidixic acid</th><th>La(nal)$_2$</th></tr>
<tr><td>

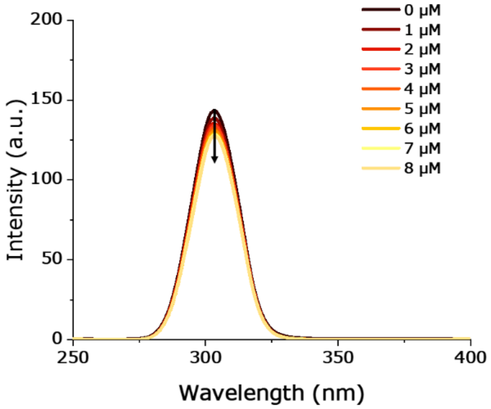

</td><td>

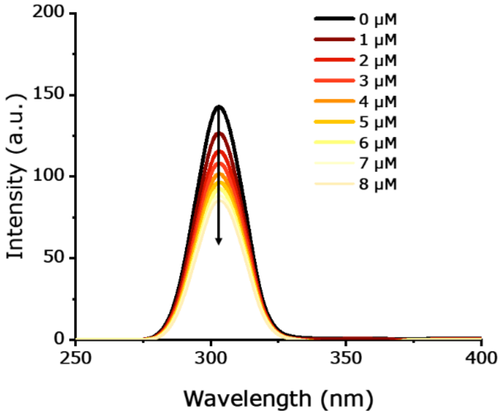

</td></tr>
<tr><th>Sm(nal)$_2$</th><th>Eu(nal)$_2$</th></tr>
<tr><td>

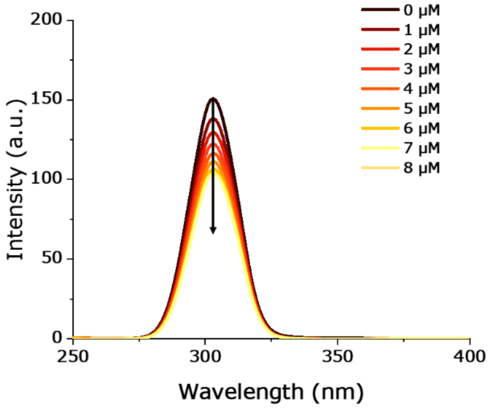

</td><td>

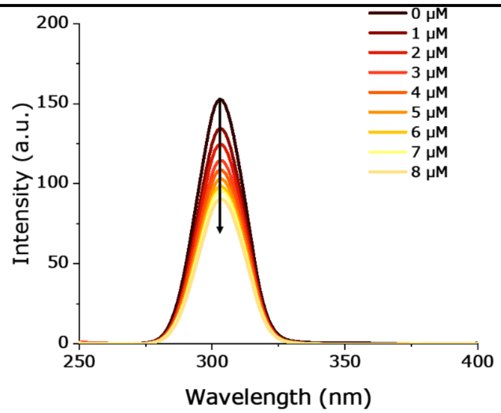

</td></tr>
<tr><th>Gd(nal)$_2$</th><th>Tb(nal)$_2$</th></tr>
<tr><td>

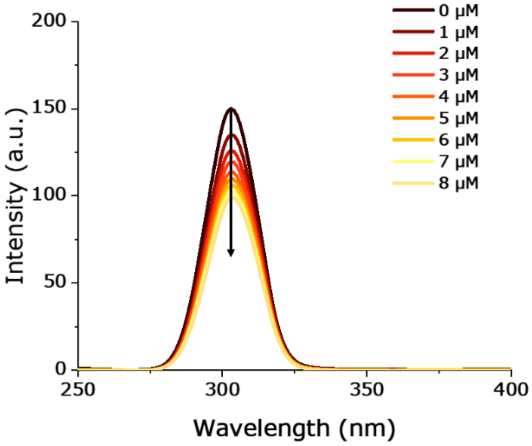

</td><td>

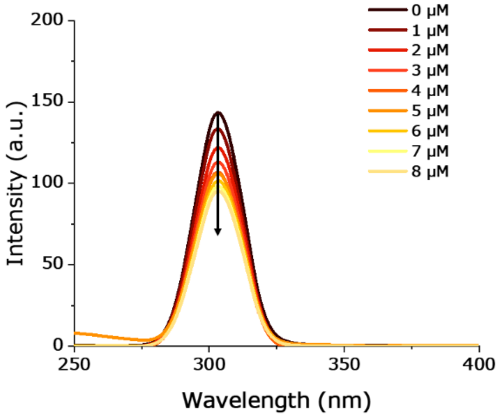

</td></tr>
</table>

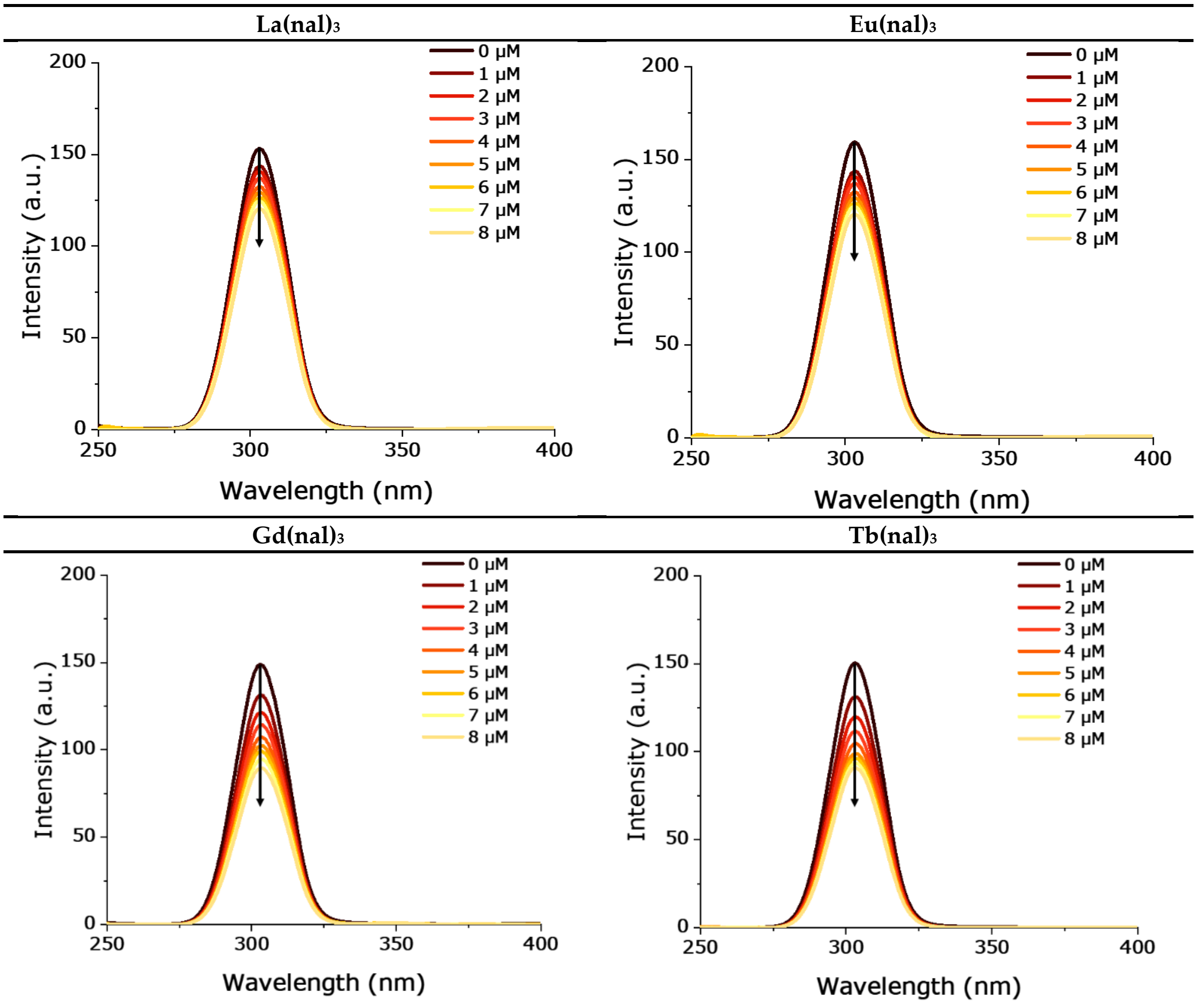

Figure S26. Synchronous spectra of the tested compounds - apo-Tf systems recorded at Δλ=15 nm. [apo-Tf] = 1 μM, [compound] = 0, 1, 2, 3, 4, 5, 6, 7, 8 μM.

Nalidixic acid

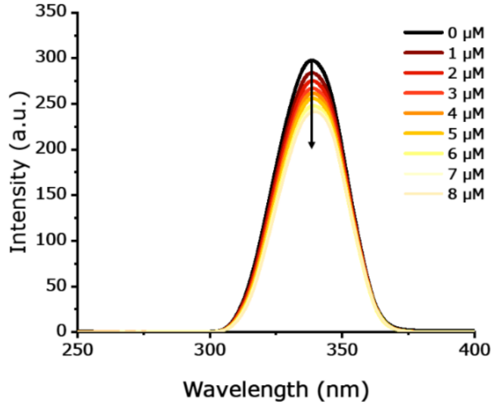

La(nal)$_2$

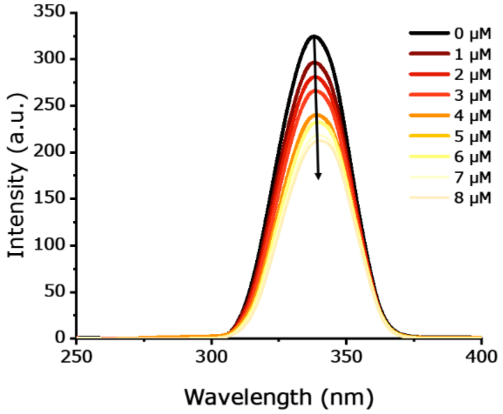

Sm(nal)$_2$

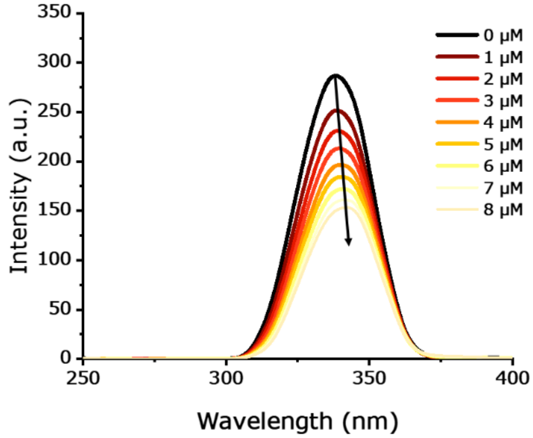

Eu(nal)$_2$

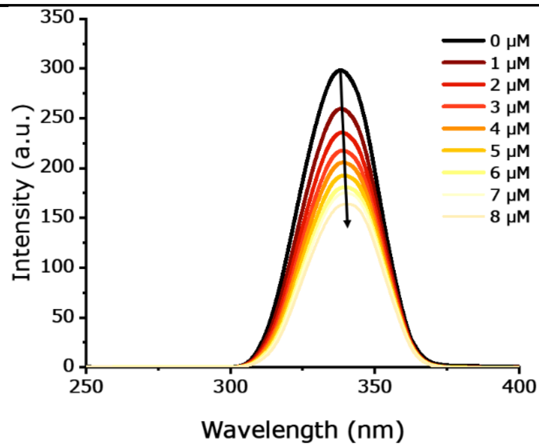

Gd(nal)$_2$

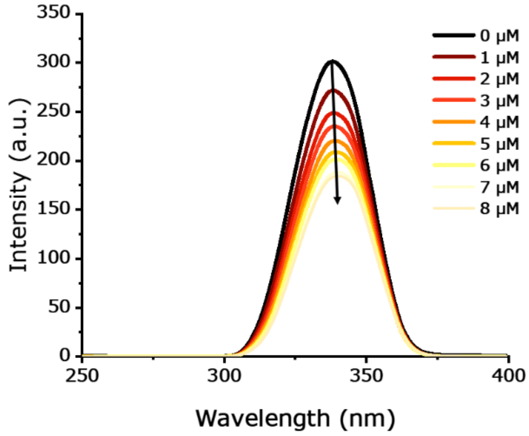

Tb(nal)$_2$

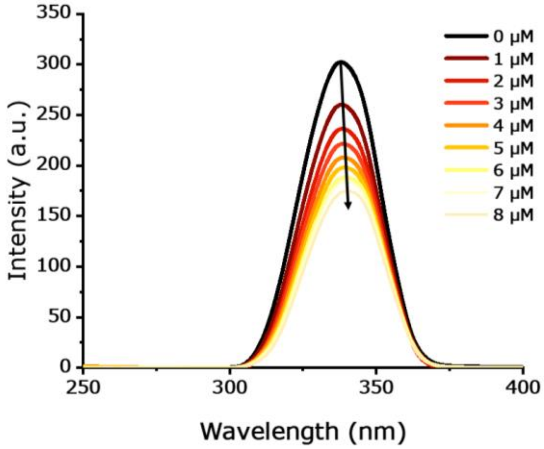

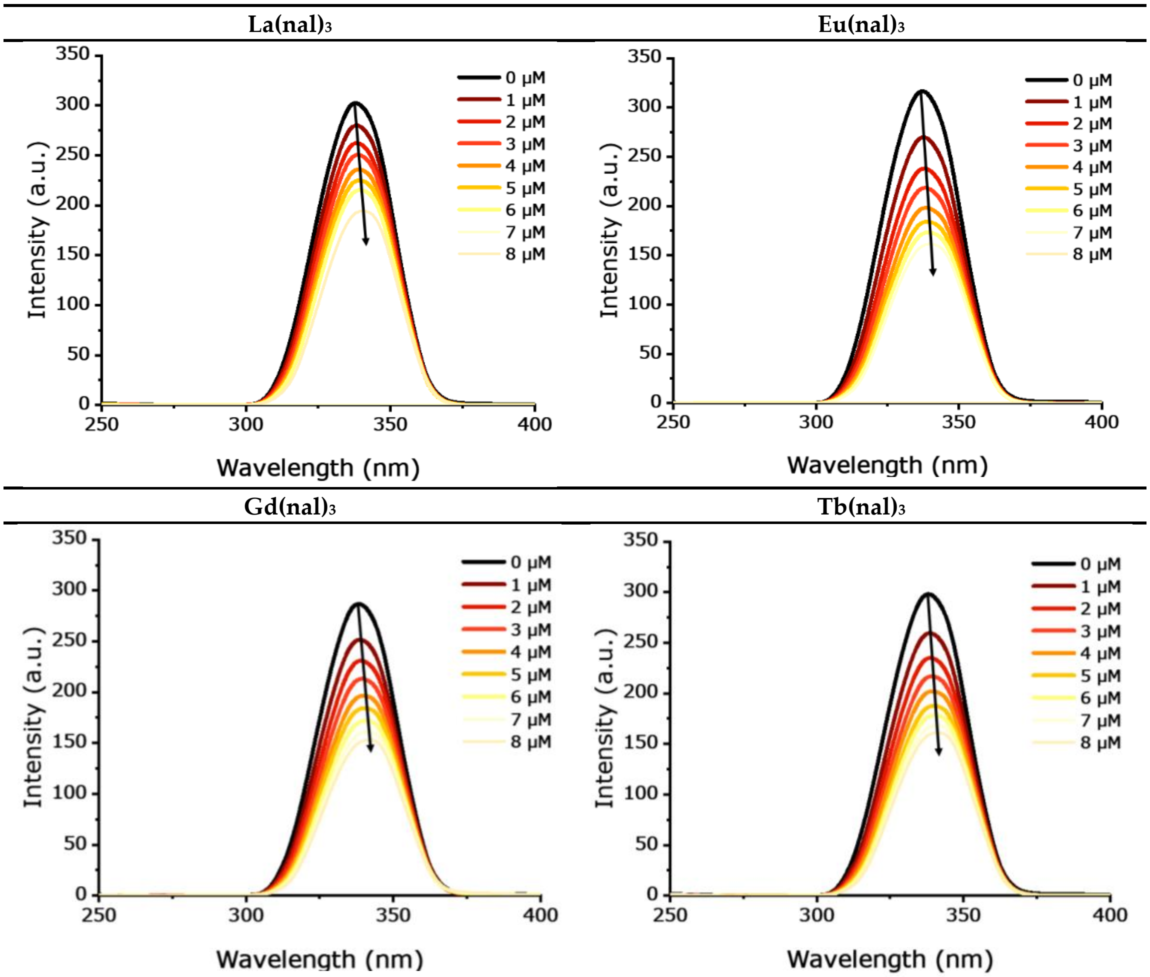

Figure S27. Synchronous spectra of the tested compounds - apo-Tf interaction systems recorded at $\Delta\lambda$=60 nm. [apo-Tf] = 1 μM, [compound] = 0, 1, 2, 3, 4, 5, 6, 7, 8 μM.
